# Trade Specialisation and Changing Patterns of Comparative Advantages in Manufactured Goods

**DOI:** 10.1007/s40797-022-00185-4

**Published:** 2022-03-16

**Authors:** Bernardina Algieri, Antonio Aquino, Marianna Succurro

**Affiliations:** 1 Department of Economics, Statistics and Finance, University of Calabria, Ponte P. Bucci Cubo 0, IT-87036 Arcavacata di Rende, Cosenza Italy; 2 Zentrum für Entwicklungsforschung, Rheinische Friedrich-Wilhelms-Universität Bonn, Genscherallee 3, D-53113 Bonn, Germany

**Keywords:** Comparative advantages, Manufacturing, Human capital, F11, F14, P52, Z32

## Abstract

Starting from the late 1950s, mainly as a reaction to Leontief’s paradoxical results, a large amount of research has underlined the great importance of technology and human capital in explaining international trade in manufactures. This study examines the patterns of comparative advantages for 42 countries and 91 manufactured classes of final and intermediate products and their changes between 2001 and 2019. The dynamics and the effects of the international fragmentation of production processes are also considered. Comparative advantages in each class of products are related to three different measures of a country’s human capital or technology endowment: the cost of labour, the level of formal education and the number of patents per capita. An indicator of home market size enters the model as a control variable. The econometric analysis reveals that in 2019, human capital or technology endowments explain comparative advantages in 70 out of 91 products. In particular, 31 products are positively and significantly associated with at least 1 of the 3 human capital or technology indicators, 34 products are negatively and significantly linked to at least 1 indicator, 5 products show contrasting results, 21 products are not related to any human capital or technology indicator. Between 2001 and 2019, comparative advantages for 15 classes of products shifted towards lower-technology countries and for 18 productions towards higher-technology countries. There were no shifts for 51 products. From a policy perspective, the distinction between high and low-technology productions could have some interesting implications. Since high-technology productions are characterised by a higher learning-by-doing rate, governments should foster domestic firms to upgrade the human capital or technology intensity of their productions.

**Supplementary Information:**

The online version contains supplementary material available at 10.1007/s40797-022-00185-4.

## Introduction

Despite the “Great Trade Collapse” of 2008 and the “Greater Trade Collapse” of 2020, global trade has experienced substantial changes through the integration of emerging economies into the global market, the international fragmentation of production processes and off-shoring pressures (Cigna et al. 2022; Baldwin and Evenett 2020; Tajoli and Felice 2018; Hanson 2012; Baldwin 2011; Baldone et al. 2001, 2002, 2007; Arndt and Kierzkowski 2001). These trends have affected the international flows of goods and services and the demand for skills and relative wages, bringing benefits and creating new policy challenges. It is well-known that trade can boost economic growth and foster aggregate welfare in several ways. It prompts a more efficient allocation of resources across sectors and enterprises, promotes competition, reduces *x* inefficiencies and stimulates innovation (e.g. OECD 2019; Aghion and Howitt 1998). Trade facilitates access to larger international markets, generating economies of scale and technological spillovers with positive implications for growth (e.g. OECD 2021, 2014; Dalum et al. 1999; Rivera-Batiz and Romer 1991). Trade enables countries to specialise in goods in which they have a comparative advantage and labour to cluster in a narrow range of tasks leading to higher productivity growth, for learning and scale effects (e.g. Andrews et al. 2018; Krugman 1980). However, the benefits of trade are not equally distributed across and within countries. For instance, countries that specialise in more dynamic and innovative production activities can more easily achieve sustained economic growth than others (Hausmann et al. 2007; Amable 2000; Grossman and Helpman 1991). The differences in relative factor endowments and technological capabilities of countries and the relative factor intensities and technological characteristics of production activities would shape international specialisation (Hanson 2012; OECD 2011; Feenstra 2010; Krugman 1998; Bowen et al. 1987; Hufbauer 1970; Ohlin 1933; Heckscher 1919). Besides, larger countries have a tendency to specialise in more differentiated products due to stronger home-market effects (Hanson and Xiang 2004; Davis and Weinstein 2003; Weder 2003; Drèze 1960). Likewise, countries with better institutions tend to have a comparative advantage in the activities whose costs are more sensitive to the quality of institutions (Nunn and Trefler 2014; Costinot 2009; Acemoglu et al. 2007; Levchenko 2007; Nunn 2007).

The competitive pressure of low-wage countries has eroded the comparative advantages of high-wage countries in some productions, and the international fragmentation of the production processes has generated large trade-flows in intermediate and unfinished goods (e.g. World Bank 2020; Timmer et al. 2013; Yi 2003; Baldone et al. 2001, 2002, 2007; Venables 1999). The shifts of productive activities towards lower-wage countries can have significant negative impacts on employment in high-wage countries. Some of these negative effects could shrink if changes in the patterns of comparative advantages could be anticipated to some extent.

In this context, the present study aims at analysing the changes in comparative advantages for a group of high-wage, mid-wage and low-wage countries, gaining a better understanding of trade developments and identifying similarities and differences across countries. At the same time, we try to account for the fragmentation of production across borders to encompass new developments in international trade (e.g. Limão and Xu 2021; Saygılı 2020; Tajoli and Felice 2018; Kimura et al. 2007; Baldone et al. 2007; Arndt and Kierzkowski 2001). In particular, we focus on a set of 42 countries and 91 classes of manufactured products for the years 2001 and 2019. The examination of final goods is complemented by an analysis regarding a group of parts and components (intermediates) that represent an increasingly large fraction of international trade (Johnson and Noguera 2017, 2012).

The topic is relevant given that comparative advantages provide an indication of where a Global Value Chain^1^ (GVC) activity should be located when several countries can handle the whole or part of the production process (Borin et al. 2021; Sopranzetti 2018; Lu and Karpova 2011). A country can enhance its equilibrium level of wages by upgrading its comparative advantages towards more innovative activities.

Changes in specialisation patterns can also have implications for inequality by shifting the demand from one factor of production to another, leading to changes in relative wages across and within skill categories. As a result, public policies could influence the wage gap between skilled and unskilled workers at home and abroad. For instance, policies that heighten access to higher education for a wider group of individuals could mitigate rising wage gaps in an environment characterised by increasing demand for high-skilled workers. Such policies could also generate spillovers to foreign countries by affecting relative wages of skilled to unskilled and trade patterns across countries.

In what follows, we introduce a new index to measure trade specialisation and a novel methodology for detecting shifts in the patterns of comparative advantages by exploiting information on trade flows and human capital endowments of different countries. In a first step, we compute a set of specialisation indices for each product and then estimate the linkage between comparative advantages and three different measures of human capital or technology endowments: education attainment, per-capita patents and labour costs of different countries. A measure of the size of a country’s internal market is also included, primarily as a control variable. The theoretical framework combines different strands of trade literature encompassing the orthodox version of the product cycle model, the new trade theory and the fragmentation literature. In a second step, we recombine the obtained cross-sectional estimates into a “comparative advantages shift matrix” that consistently accounts for changes in the patterns of comparative advantages. The novel “comparative advantage shift matrix” allows us to single out movements in the structure of comparative advantages across low-wage, mid-wage and high-wage countries. We think that the possibility of distinguishing between high and low-technology products could have some interesting policy implications. Since higher rates of learning-by-doing externalities characterise high-technology products, policy measures should facilitate improvements in the human capital or technology endowment of a country.

The remainder of the study is organised as follows. Section 2 reviews the economic literature on comparative advantages and their shifts over time. Section 3 presents the analysis on comparative advantages and computes the indices entering the empirical exercise. Section 4 outlines the theoretical framework, estimates the econometric model and proposes a classification of the manufactured products. Section 5 concludes.

## Literature Review

Robert Torrens (1815) was the first to enunciate, already in its ‘paradoxical’ version, the theory of ‘comparative advantage’. In his *Essay on the External Corn Trade*, Torrens pointed out that England could benefit from trade by importing corn from Poland in exchange for manufactures, even if corn was produced more efficiently in England than in Poland. Two years later, David Ricardo (1817) explained the same principle in *the Principles of Political Economy and Taxation,* Chapter VII. In Ricardo’s famous example, Portugal had an absolute advantage over England in cloth and wine but had a comparative advantage in wine and a comparative disadvantage in cloth. England had an absolute disadvantage in both productions, but a comparative advantage in cloth and a comparative disadvantage in wine. Torrens and Ricardo argued that in such a situation, trade could benefit both countries. The two economists demonstrated that the differences in relative (or comparative) costs between countries trigger the gains from trade. Nevertheless, they did not explain the possible determinants of inter-country differences in comparative costs.

More than one century later, Eli Heckscher (1919), while trying to assess the influence of international trade on the prices of the factors of production, presented a fundamental intuition of the sources of comparative advantages. This intuition anticipated the ‘skill version’ explanation of comparative advantages developed about fifty years later by Keesing (1966), Hufbauer (1970) and other researchers to clarify the Leontief paradox:

“Our primary purpose … is to discover the influence of foreign trade upon the prices of the factors of production.…this question leads to some fundamental assumptions in the theory of foreign trade. The first of these assumptions concerns the reasons for differences in comparative costs among countries.… With the same relative scarcity of factors of production … Both countries will have the same comparative costs for all goods… A difference in the relative scarcity of the factors of production between one country and another is thus a necessary condition for a difference in comparative costs ….A further indispensable condition is that the proportions in which the factors of production are combined shall not be the same for one commodity as for another … It must be stressed that the term “factor of production” does not refer simply to the broad categories of land, capital, and labour, but to the different qualities of each of these. International trade may be attributable to particularly fertile soil in one country, compared with another, or to a particularly skilled population, as well as to a disproportionate distribution of land or workers in general” (Heckscher 1919, in American Economic Association, 1950, pp. 278–279).

Heckscher’s argument was developed by his pupil Bertil Ohlin (1933). Ohlin analysed geographic districts or regions rather than nations, highlighting the importance of factor endowments, skills and scale economies on comparative advantages, so anticipating the analyses by “the new trade theories”:

“What are the causes of division of labour in general?…First of all, some individuals have a greater ability for certain tasks than others… Second, even if all individuals have the same natural abilities, it would still be advantageous to have specialization in one or a small number of occupations. In this way much greater skill can be acquired than if everyone produced everything for himself … This is one aspect of what is generally called the “economics of large scale production” … Turning from individuals to regions, one finds that the latter, like the former, is very differently endowed with facilities for the production of various articles. One reason is that they are differently supplied with productive factors. … each region is best equipped to produce the goods that require large proportions of the factors relatively abundant there … (pp. 6–7)…Countries with a large supply of labour having high technical skills make for superiority in industries requiring plenty of educated labour” (p. 50).

In the fifties, Wassily Leontief tested the prediction of the factor proportions theory for the United States with quite disappointing results: the capital/labour ratio turned out to be greater for the US imports than the US exports! Leontief’s results stimulated a remarkable number of works, mainly empirical, but some theoretical too. From the theoretical point of view, Linder (1961) and Drèze (1960) provided very stimulating contributions. Linder argued that a representative domestic demand for goods could fuel comparative advantages. Drèze suggested that countries with a larger home market have a comparative advantage in differentiated products, while smaller countries have a comparative advantage in homogeneous products. Rosenberg (1970) pointed to the role of technology in shaping international specialisation and the positive externalities generated by higher technology productions:

“…we accord a more prominent role to the effects of a dynamic technology comparative advantage …no longer based upon cost differences which are rooted in immutable forces of climate or geology. Rather it is the continually changing result of human ingenuity and inventiveness, reflecting the differential capacity of different countries to develop techniques … certain kinds of economic activities are more successful than others in contributing to the development of inventive abilities…Technological change is not, after all, something that has emerged in a random way from all sectors of the economy. It is the result of a problem-solving skill which, historically, has been heavily concentrated in specific sectors of the economy. In the early stages of industrialization, these skills were heavily concentrated in machine tools and engineering; later … this focus shifted to chemistry-based and more recently to electronic-based industries. … If one productive process involves a learning activity which leads to new techniques or products …these are externalities of the greatest importance” (in Vernon ed. 1970, pp. 70–72).

Feenstra and Rose (2000) showed a strong relationship between advanced export structures, high productivity levels and fast growth rates. Bensidoun et al. (2001) found that the growth effects of trade depend on the type of products in which countries specialise. Cuaresma and Wörz (2005) tested whether exports in technology-intensive industries have a higher potential for positive externalities, showing a significant effect in the case of developing countries.

The dynamics of factors requirements, and hence of comparative advantages, as products or industries grow older, was elucidated by Ronald Jones (1970, pp. 84–87):

“Vernon argues that advanced countries tend to have a comparative advantage in producing those commodities that are newly being developed. …Vernon suggests that high labour costs at home may nonetheless be outweighed by the advantage of having a location in which a variety of special kills is readily accessible. ….the input mix at the early stages of the development of the product is different from the input mix later, when production becomes standardized. …this suggests a three factors model: capital, “ordinary” labour, and a third factor that comprises a host of special skills on the part of labour… Advanced countries such as the United States, have a relative abundance of this third factor and hence a comparative advantage in producing new commodities at early stages of production. …It is at the early stages, where new products are being introduced, that relatively heavy use is made of resources in R&D”.

Specialisation patterns can be expected to change over time, mainly because of changes in relative factor intensities of products and relative factor endowments of countries, the introduction of new products, and the integration of new countries in international markets. Most studies examined some examples of changes in trade specialisation occurring for particular products and countries. Aquino (1981) proposed a simple methodology to investigate the degree, speed and timing of the shifts in comparative advantages for 25 manufactured products over 1962–1974. Balassa and Noland (1989) econometrically examined the changes in comparative advantages of Japan and the United States across 167 manufactured product categories between 1967 and 1983. In particular, comparative advantages have been related to inter-industry differences in factor intensities. Japan significantly changed its specialisation patterns, shifting from unskilled-labour intensive goods to human capital and R&D intensive products. The United States maintained their specialisation in physical capital, human capital and R&D intensive goods. Dalum et al. (1998) analysed the degree of stability in the export specialisation structure of 20 OECD countries from 1965 to 1992. Their results showed that the national specialisation patterns had been rather sticky, with a tendency to de-specialisation in the medium to long term. Hiley (1999) investigated the changes in revealed comparative advantages of Asian countries. The author unveiled a profound change in the manufacturing structure, involving the transition from simple consumer goods and resource‐based processing activities towards more sophisticated productions. Brasili et al. (2000) analysed the dynamics of trade patterns in six large industrialised countries and eight fast-growing Asian economies. The authors found that advanced countries had highly persistent trade patterns, whereas emerging countries experienced rapid changes. Despite the higher specialisation of emerging countries, both country groups showed a tendency towards a reduced polarization and a more symmetric distribution in their specialisation. De Benedictis (2005) examined the Italian export composition and its evolution over three decades starting from the 1970s. Using a non-parametric approach, he found that several sectors characterised by comparative disadvantages in the 1980s presented a comparative advantage by the end of the 1990s. The sectoral composition of Italian exports, examined at a high disaggregation level, was found not so similar to that of the newly industrialised countries exporting labour-intensive products. Hanson (2012) explored the changes in trade specialisation associated with the integration of low- and mid-income nations into the global economy. The author showed that specialisation takes place according to comparative advantages of resource-based or technology-based type. In addition, specialisation in low- and mid-income countries can change rapidly over time. Kim and Kim (2015) investigated the shifts in comparative advantages for the major ASEAN countries in the period 2000–2010: the export pattern of South Korea changed faster than that of Vietnam, Singapore, the Philippines, Thailand and Malaysia; the export structure of Indonesia remained instead quite stable. Whang (2017) documented that revealed comparative advantages in manufacturing were positively associated with the rate of decline in the labour share for agriculture. Quantitative experiments indicated that a slight difference in a country’s comparative advantage accounts for a large variation in the structural transformation of open economies. Stellian and Danna-Buitrago (2019) calculated comparative-advantage sustainability and comparative-disadvantage recurrences for Colombia in the Pacific Alliance for 2013–2017 and obtained results valuable for designing effective public policies. More recently, Liu et al. (2020) demonstrated that the development of financial and business services improved the revealed comparative advantage of manufacturing sectors that use these services intensively, but not that of other manufacturing sectors. The authors also proved that a country might partially overcome the drawback of underdeveloped domestic services by increasing imported services inputs. Hence, lower services trade barriers could promote manufacturing exports in developing countries.

A more recent branch of the literature focused on the increasing international fragmentation of production across borders that changed the nature of international competition and trade specialisation. In this context, several authors questioned whether traditional modelling of the determinants of international trade was still adequate to explain comparative advantages and international specialisation (Timmer et al. 2021; Sposi et al. 2021; Saygili 2020; Hamid and Aslam 2017; Cingolani et al. 2017; Timmer et al. 2013; Baldone et al. 2007; Kimura et al. 2007; De Simone 2008; Arndt 1997; Athukorala and Yamashita 2006; Jones et al. 2005; Amiti 2005; Grossman and Helpman 2005; Yi 2003; Jones 2000; Venables 1999; Jones and Kierzkowski 1990, 2001). These researchers contributed to the debate by developing new theoretical models or proposing new measures of competitiveness to take into account international production fragmentation, an important feature of the deepening structural interdependence of the globalised world economy.

It is worth mentioning that GVCs have existed for centuries. Up to the 1990s, they were characterised by exports of primary commodities from resource-rich countries to developed countries that produced final goods to be exported to third countries. GVCs flourished rapidly from 1990 to 2007—from about 40 to 52% of global trade (World Bank 2020, p. 2)—as technological advances in transportation, information, and communications, as well as lower trade barriers, encouraged manufacturers to expand production processes outside national borders. In that phase, GVCs consisted of worldwide fragmented productive processes involving high-technology products, characterised by some stages of labour-intensive productions. The more labour-intensive phases of high-technology products designed in technology-rich countries were outsourced to countries with a great abundance of very cheap labour, particularly East Asia (e.g. China, Vietnam, Cambodia) and Eastern Europe (e.g. Poland, Czech Republic, Slovakia, Romania). The fragmentation allowed developing and developed countries to benefit from specialisation in niche tasks. Most countries in East Asia, North America and Western Europe participated in complex GVCs, producing advanced manufactures (e.g. machinery and electronics) and services (e.g. transportation), and engaging in innovative activities. By contrast, several countries in Africa, Latin America, and Central Asia produced commodities for further processing in other countries.^2^ After 2007, however, trade and GVC growth decelerated. One reason for this declining trend has been the drop in overall economic growth. Another reason has been the reversal in trade reforms and liberalisation initiatives. Furthermore, the fragmentation of production in the most dynamic regions and sectors has matured. China, in particular, has been upgrading and integrating at home more and more production stages, becoming the hub for numerous Asian smaller countries (“Factory Asia”). In the United States, a booming shale sector reduced oil imports by one-fourth between 2010 and 2015 and slightly reduced the incentives to outsource manufacturing production (World Bank 2020, p. 2). Other causes of the slowdown in GVCs have been the reductions of international differences in labour cost, and the development of automation, 3D printing, internet of things, and other characteristics of “Industry 4.0”, which stimulated the “re-shoring” in high-wage countries of some labour-intensive stages of production.

Some studies have questioned whether the increasing trade in parts and components reduces the reliability of the traditional indicators of competitiveness based on gross exports. By linking production processes across borders, trading in intermediate inputs would create two distinct measurement problems. First, with fragmentation, trade data are double-counted because goods in process cross multiple national borders before getting embodied in the final product. This “double-counting” problem means that conventional data overstate the domestic (value-added) content of exports. Second, trade-shares calculated using reported data can lead to wrong inferences due to the relative importance of trade with the ‘region’ and the rest of the world (Athukorala and Yamashita 2006). The main “double-counting” and multi-country production chains would imply a hidden trade structure in value-added underlying gross trade flows. However, we believe that these problems are relevant when absolute values of exports and imports are used for calculating bilateral trade balances. They are negligible if we consider an index of comparative advantages equal to the difference between the normalised share of exports and the normalised share of imports as presented in Sect. 3. In addition, we consider a set of product categories that can securely be supposed to contain only intermediate inputs (reflected in the word “Parts of” or “Components” in the Standard International Trade Classification, SITC). In this way, comparative advantages can emerge not only in different sectors but also in different stages of production within sectors. By fragmenting complex products, GVCs allow countries to specialise in specific parts or tasks of production, avoiding domestic supply and demand constraints.

Baldone et al. (2001, 2002, 2007), De Benedictis and Tamberi (2000, 2004), De Benedictis and Tajoli (2004, 2007a, b, 2008), De Benedictis et al. (2009) and Finger and Kreinin (1979) devoted particular attention to inter-country comparisons of trade patterns. De Benedictis and Tajoli (2004, 2006, 2007a) tested four indexes of trade similarity: the Pearson coefficient of linear correlation, the Spearman coefficient of rank correlation, the Euclidean distance index and the Bray–Curtis metric, obtained as a standardisation of the Manhattan distance.^3^ Based on the Bray–Curtis metric, considered the most appropriate by the authors, they analysed the similarity of the export structures toward the EU market between Poland, Hungary, Romania and Bulgaria and the pre-2004 European Union members, from 1989 to 2001. They showed that processed trade was crucial in explaining changes in the overall structure of exports of these four countries and greater economic integration in terms of trade flows and processing trade did not always lead to more export similarity between these countries and the EU15. Their main result was that similarity in export composition had a positive, significant and nonlinear impact on catching-up and seemed to be driven by the growth of the main export markets more than by other factors. For the Central-Eastern European Countries and the EU, a correlation between convergence in export patterns and income levels per capita were quite robust since 2004 (De Benedictis and Tajoli 2004). Moving from that result, De Benedictis and Tajoli (2007a, b, 2008) explored the mechanisms that might have given rise to it.

## Trade Specialisation

To contribute to the existing literature and assess the changing patterns of comparative advantages, we first present and compute different specialisation indices and then we use them in the econometric analysis.

### Measuring Comparative Advantages and Disadvantages

Balassa (1965) proposed a simple, but ingenious, measure of comparative advantages as “revealed” by trade flows: the ratio of a country's share in the world exports of a particular product to its share in the world exports of all goods, usually multiplied by 100. The index ranges from 0 to + ∞ with a demarcation value at 100 (or 1). The greater the value of the index (above 100), the stronger is the comparative advantage; the smaller the value (below 100), the stronger is the comparative disadvantage. Formally, the Balassa index (B) is given by:

1 $$ B_{ij} = \left( {{\raise0.7ex\hbox{${\frac{{\exp_{ij} }}{{\sum\nolimits_{i = 1}^{N} {\exp_{ij} } }}}$} \!\mathord{\left/ {\vphantom {{\frac{{\exp_{ij} }}{{\sum\nolimits_{i = 1}^{N} {\exp_{ij} } }}} {\frac{{\sum\nolimits_{j = 1}^{M} {\exp_{ij} } }}{{\sum\nolimits_{i = 1}^{N} {\sum\nolimits_{j = 1}^{M} {\exp_{ij} } } }}}}}\right.\kern-\nulldelimiterspace} \!\lower0.7ex\hbox{${\frac{{\sum\nolimits_{j = 1}^{M} {\exp_{ij} } }}{{\sum\nolimits_{i = 1}^{N} {\sum\nolimits_{j = 1}^{M} {\exp_{ij} } } }}}$}}} \right)*100 $$

where e*xp*_*ji*_ stands for country *j*’s exports of commodity *i*, with *j* = 1…M countries and *i* = 1… N products.

Due to its simplicity and intuitive interpretation, the Balassa index is widely used in the literature, despite some shortcomings. However, this index has an asymmetric distribution and, including only export flows, overlooks the international fragmentation of production processes (e.g. Yeats 1985; Bowen 1983). For these reasons, we have computed three further indices: the Revealed Symmetric Comparative Advantage (RSCA) index proposed by Dalum et al. (1998)^4^ that adjusts for asymmetry, the Lafay index (1992) that corrects for international fragmentation of production stages and a new proposed index—called the Revealed Symmetric Augmented Comparative Advantage (RSACA)—that accounts for asymmetry, export and import flows^5^ and thus fragmentation.

The Symmetric Balassa index proposed by Dalum et al. (1998) is specified as:

2 $$ RSCA_{ij} = \frac{{B_{ij} - 1}}{{B_{ij} + 1}} = \frac{{\left( {{{\frac{{\exp_{ij} }}{{\sum\nolimits_{i = 1}^{N} {\exp_{ij} } }}} \mathord{\left/ {\vphantom {{\frac{{\exp_{ij} }}{{\sum\nolimits_{i = 1}^{N} {\exp_{ij} } }}} {\frac{{\sum\nolimits_{j = 1}^{M} {\exp_{ij} } }}{{\sum\nolimits_{i = 1}^{N} {\sum\nolimits_{j = 1}^{M} {\exp_{ij} } } }}}}} \right. \kern-\nulldelimiterspace} {\frac{{\sum\nolimits_{j = 1}^{M} {\exp_{ij} } }}{{\sum\nolimits_{i = 1}^{N} {\sum\nolimits_{j = 1}^{M} {\exp_{ij} } } }}}}} \right) - 1}}{{\left( {{{\frac{{\exp_{ij} }}{{\sum\nolimits_{i = 1}^{N} {\exp_{ij} } }}} \mathord{\left/ {\vphantom {{\frac{{\exp_{ij} }}{{\sum\nolimits_{i = 1}^{N} {\exp_{ij} } }}} {\frac{{\sum\nolimits_{j = 1}^{M} {\exp_{ij} } }}{{\sum\nolimits_{i = 1}^{N} {\sum\nolimits_{j = 1}^{M} {\exp_{ij} } } }}}}} \right. \kern-\nulldelimiterspace} {\frac{{\sum\nolimits_{j = 1}^{M} {\exp_{ij} } }}{{\sum\nolimits_{i = 1}^{N} {\sum\nolimits_{j = 1}^{M} {\exp_{ij} } } }}}}} \right) + 1}} $$

Its value ranges from − 1 to  1 (with a demarcation value at 0). A value larger than zero indicates that country *j* has a revealed comparative advantage in *i*. A value less than zero points to a comparative disadvantage in product *i* for country *j*.

The Lafay index (LFI), enclosing both exports and imports for a specific country, allows one to control for intra-industry trade and re-export flows generated by the processes of international fragmentation of production. For a given country, *j*, and for any given product, *i*, the Lafay index is defined as:

3 $$ LFI_{ij} = 100 \cdot \left( {\frac{{\exp_{ij} - imp_{ij} }}{{\exp_{ij} + imp_{ij} }} - \frac{{\sum\nolimits_{i = 1}^{N} {\left( {\exp_{ij} - imp_{ij} } \right)} }}{{\sum\nolimits_{i = 1}^{N} {\left( {\exp_{ij} + imp_{ij} } \right)} }}} \right) \cdot \frac{{\exp_{ij} + imp_{ij} }}{{\sum\nolimits_{i = 1}^{N} {\left( {\exp_{ij} + imp_{ij} } \right)} }} $$

The Lafay index weights each product’s contribution according to the respective importance in trade. A positive value of the Lafay index designates the existence of comparative advantages in a given product *i*. The larger is the value, the higher is the comparative advantage. Conversely, a negative value points to despecialisation. By construction, the following relation holds: $\sum\nolimits_{i = 1}^{N} {LFI_{ij} } = 0$.

The proposed Revealed Symmetric Augmented Comparative Advantage (RSACA) has been computed as:

4 $$ \begin{aligned} RSACA_{ij} &= \frac{{B_{ij}^{\exp ort} - 1}}{{B_{ij}^{\exp ort} + 1}} - \frac{{B_{ij}^{import} - 1}}{{B_{ij}^{import} + 1}}\\ & = \frac{{\left( {{{\frac{{\exp_{ij} }}{{\sum\nolimits_{i = 1}^{N} {\exp_{ij} } }}} \mathord{\left/ {\vphantom {{\frac{{\exp_{ij} }}{{\sum\nolimits_{i = 1}^{N} {\exp_{ij} } }}} {\frac{{\sum\nolimits_{j = 1}^{M} {\exp_{ij} } }}{{\sum\nolimits_{i = 1}^{N} {\sum\nolimits_{j = 1}^{M} {\exp_{ij} } } }}}}} \right. \kern-\nulldelimiterspace} {\frac{{\sum\nolimits_{j = 1}^{M} {\exp_{ij} } }}{{\sum\nolimits_{i = 1}^{N} {\sum\nolimits_{j = 1}^{M} {\exp_{ij} } } }}}}} \right) - 1}}{{\left( {{{\frac{{\exp_{ij} }}{{\sum\nolimits_{i = 1}^{N} {\exp_{ij} } }}} \mathord{\left/ {\vphantom {{\frac{{\exp_{ij} }}{{\sum\nolimits_{i = 1}^{N} {\exp_{ij} } }}} {\frac{{\sum\nolimits_{j = 1}^{M} {\exp_{ij} } }}{{\sum\nolimits_{i = 1}^{N} {\sum\nolimits_{j = 1}^{M} {\exp_{ij} } } }}}}} \right. \kern-\nulldelimiterspace} {\frac{{\sum\nolimits_{j = 1}^{M} {\exp_{ij} } }}{{\sum\nolimits_{i = 1}^{N} {\sum\nolimits_{j = 1}^{M} {\exp_{ij} } } }}}}} \right) + 1}} - \frac{{\left( {{{\frac{{imp_{ij} }}{{\sum\nolimits_{i = 1}^{N} {imp_{ij} } }}} \mathord{\left/ {\vphantom {{\frac{{imp_{ij} }}{{\sum\nolimits_{i = 1}^{N} {imp_{ij} } }}} {\frac{{\sum\nolimits_{j = 1}^{M} {imp_{ij} } }}{{\sum\nolimits_{i = 1}^{N} {\sum\nolimits_{j = 1}^{M} {imp_{ij} } } }}}}} \right. \kern-\nulldelimiterspace} {\frac{{\sum\nolimits_{j = 1}^{M} {imp_{ij} } }}{{\sum\nolimits_{i = 1}^{N} {\sum\nolimits_{j = 1}^{M} {imp_{ij} } } }}}}} \right) - 1}}{{\left( {{{\frac{{imp_{ij} }}{{\sum\limits_{i = 1}^{N} {imp_{ij} } }}} \mathord{\left/ {\vphantom {{\frac{{imp_{ij} }}{{\sum\limits_{i = 1}^{N} {imp_{ij} } }}} {\frac{{\sum\limits_{j = 1}^{M} {imp_{ij} } }}{{\sum\limits_{i = 1}^{N} {\sum\limits_{j = 1}^{M} {imp_{ij} } } }}}}} \right. \kern-\nulldelimiterspace} {\frac{{\sum\nolimits_{j = 1}^{M} {imp_{ij} } }}{{\sum\nolimits_{i = 1}^{N} {\sum\nolimits_{j = 1}^{M} {imp_{ij} } } }}}}} \right) + 1}}\end{aligned} $$

This index is the difference between the symmetric Balassa index calculated for exports and the symmetric Balassa index for imports. It ranges between − 2 and 2 with a demarcation value at 0 and ostensibly provides an unbiased measure of the intensity in comparative advantages and, as mentioned in the literature review, would allow us to cope with the “double-counting” problem.^6^ Similarly to the Lafay index, RSACA takes into account both exports and imports, and captures intra-trade and re-export flows. Differently from Lafay, it includes trade flows at the world level and, hence, can be considered as a “true” comparative advantage index. Moreover, the Lafay index appears to be excessively influenced by the industry size; and thus it would be more appropriate for a country-level analysis, than for a cross-section study.^7^

Because of the international fragmentation of production, it would be more correct to obtain the indices of comparative advantages based on value-added trade flows rather than in terms of gross exports and imports. Unfortunately, the necessary data are not yet available for a sufficiently high number of products and recent years. However, for the significance of our analysis, this does not seem to be a major problem, since we found that for almost all the classes of manufactured products for which the OECD (2018) has estimated both gross exports and the value-added content of gross exports in 2015, the correlation between the Balassa index calculated considering the value-added content of gross exports and the values of the Balassa index calculated on the basis of gross exports is very high, as it appears from Table 1.

**Table 1:** Linear correlation (RQ) coefficient between the values of the Balassa index of comparative advantage in domestic value-added content of gross exports and the Balassa index of comparative advantage in gross exports, 2015; Source: Own elaborations on data extracted on 30 09 2021 from: https://stats.oecd.org/Index.aspx?DataSetCode=TIVA_2018_C1

	RQ between Balassa VA and Balassa GR EXP (1)	Total domestic value added content of gross exports, 2015 (2)		Total gross exports, 2015 (3)		(2) / (3) %
		Abs. values	%	Abs. values	%	
D26: Computer, electronic and optical products	0.96	613,103	14.6	740,509	15.5	83
D13T15: Textiles, wearing apparel, leather prods	0.99	366,586	8.7	397,336	8.3	92
D27: Electrical equipment	0.98	258,407	6.2	296,549	6.2	87
D28: Machinery and equipment, nec	0.96	395,266	9.4	437,589	9.2	90
D29: Motor vehicles, trailers and semi-trailers	0.99	283,966	6.8	317,012	6.6	90
D30: Other transport equipment	0.98	175,256	4.2	195,392	4.1	90
DINFO: Information industries	0.96	819,687	19.5	958,932	20.1	85
D10T12: Food products, beverages and tobacco	0.98	264,103	6.3	284,265	6.0	93
D17T18: Paper products and printing	0.10	67,356	1.6	72,711	1.5	93
D20T21: Chemicals and pharmaceutical products	0.99	411,080	9.8	455,184	9.5	90
D22: Rubber and plastic products	0.97	126,693	3.0	142,305	3.0	89
D24: Basic metals	0.97	270,228	6.4	307,005	6.4	88
D25: Fabricated metal products	0.98	143,760	3.4	159,790	3.3	90
TOTAL		4,195,491	100	4,764,579	100	88

### Specialisation Indices Results

Based on the aforementioned indices (2), (3) and (4), we have computed the comparative advantages and disadvantages of 42 countries in 91 groups of intermediate^8^ and final products for the years 2001 and 2019. The list of countries is reported in Table A1 in the Appendix. Detailed results on RSACA are presented in Tables S1–S2 in the Online Supplementary Material.

The values of the Revealed Symmetric Augmented Comparative Advantage (Tables S1–S2) reveal that China has registered significant changes in comparative advantages from 2001 to 2019. Declines in China’s comparative advantages have especially been recorded for lead and articles thereof (from 1.05 to − 0.48), articles of leather, saddlery, travel goods, handbags (from 1.58 to 0.80), explosives and pyrotechnic products (from 1.58 to 0.80), preparations of vegetables, fruits, nuts (from 1.06 to 0.57), footwear (from 1.38 to 0.87), preparations of meat, fish, molluscs (from 1.44 to 0.98), apparel and clothing accessories, both not knitted or crocheted (from 1.34 to 1.09) and knitted or crocheted (from 1.32 to 1.02), toys and games (from 1.43 to 1.25). In the same years, China experienced strong increases in comparative advantages in: knitted or crocheted fabrics (from − 0.1 to 0.98), glasses and glassware (from − 0.11 to 0.31), ships, boats and floating structures (from 0.18 to 0.86), machinery, both non-electrical (from − 0.15 to 0.28) and electrical (from − 0.08 to 0.06), furniture (from 1.19 to 1.23), articles of iron and steel (from 0.47 to 0.78), silk (from 0.62 to 1.07), miscellaneous articles of base metal (from 0.63 to 0.93), electronic integrated circuits (from 0.45 to 1.01). China’s comparative disadvantage disappeared for parts of motor vehicles (from − 0.27 to 0.02) and decreased significantly for parts of aircraft (from − 0.47 to − 0.13).

France strengthened its comparative advantage in perfumery, cosmetics and toilet preparations (from 0.49 to 0.51), other vegetable textile fibre manufactures (from 0.35 to 0.95), explosives and pyrotechnic products (from 0.26 to 0.42), articles of leather (from 0.19 to 0.25) and miscellaneous chemical products (from 0.11 to 0.24). France’s comparative advantages decreased in aircraft (from 0.38 to 0.24), rubbers and articles thereof (from 0.19 to 0.02), beverages (from 0.70 to 0.63), vehicles other than railways or tramways (from 0.27 to − 0.03), glasses and glassware (from 0.11 to 0.01).

Germany deepened its comparative advantages in optical, photographic, cinematographic, medical or surgical instruments (from 0.09 to 0.18), machinery, mechanical appliances, nuclear reactors, boilers, parts thereof (from 0.09 to 0.14). The country lessened its specialisation in railway or tramway locomotives, railway or tramway track (from 0.13 to − 0.16), man-made staple fibres (from 0.15 to − 0.28), knitted or crocheted fabrics (from 0.19 to 0.03), wadding, felt and nonwovens (from 0.32 to 0.1), miscellaneous articles of base metal (from 0.24 to 0.11), vehicles other than railway or tramway rolling stock (from 0.32 to 0.17), printed books (from 0.23 to 0.12). Germany shifted from a significant comparative disadvantage in ‘parts of’ aircraft and spacecraft (− 0.41) to a slight advantage (0.06).

In 2001, Italy had a substantial comparative advantage in furniture, ceramic products, articles of stone and clothing accessories. However, in 2019, the country experienced a decline in comparative advantages in all these product categories: furniture (from 0.88 to 0.49), ceramic products (from 0.79 to 0.56), articles of stone (from 0.65 to 0.3), and miscellaneous articles of base metal (from 0.61 to 0.26). Further drops have been detected for apparels and clothing accessories, both knitted or crocheted (from 0.34 to − 0.03) and not knitted or crocheted (from 0.35 to 0.11), headgears and parts thereof (from 0.52 to 0.12), footwear (from 0.41 to 0.16). Relatively large increases have been registered for ships, boats and floating structures (from 0.13 to 0.34), machinery and mechanical appliances (from 0.25 to 0.32), aircraft, spacecraft, and parts thereof (from − 0.23 to 0.05). Italy registered significant changes in comparative advantages or disadvantages for three out of five intermediate products connected to the “new” international fragmentation: from − 0.26 to 0.04 for electronic integrated circuits and parts thereof; from 0.04 to − 0.73 for parts of aircraft and spacecraft; from 0.16 to − 0.16 for parts of telephone sets, telephones and cellular networks.

Spain retained its comparative advantages in railway locomotives, explosives and pyrotechnic products while registering a marked increase in zinc (from 0.77 to 1.23), beverages, spirits and vinegar (from 0.32 to 0.47), essential oils, perfumery and cosmetics (from 0.03 to 0.21), salt, sulphur, earth and stone, lime and cement (from 0.12 to 0.39), aircraft and spacecraft (from − 0.07 to 0.21). On the contrary, the country lowered its comparative advantages in sugars and sugar confectionery (from 0.20 to − 0.07), animal or vegetable fats and oils (from 0.74 to 0.29), preparations of meat, fish, molluscs (from 0.44 to 0.11), soap, washing preparations, waxes, candles (from 0.44 to 0.14), furskins and artificial fur (from 0.63 to 0.33), lac, gums, resins (from 0.59 to 0.36), cork (from 0.26 to 0.14).

The UK increased its comparative advantages in parts of aircraft and spacecraft (from 0.00 to 0.54), beverages, spirits and vinegar (from 0.06 to 0.24), raw hides and skins and leather (from 0.18 to 0.43), iron and steel (from 0.13 to 0.23), organic chemicals (from 0.2 to 0.32), printed books and newspapers (from 0.27 to 0.33), while decreased its comparative advantages in electronic integrated circuits and parts thereof (from 0.89 to 0.12); salt, sulphur, earths and stone (from 0.31 to 0.02), mineral fuels and oils (from 0.29 to 0.08), inorganic chemicals (from 0.23 to 0.07), pharmaceutical products (from 0.27 to 0.18). In 2019, the country moved from a comparative advantage to a disadvantage in photographic or cinematographic goods (from 0.18 to − 0.29), tobacco (from 0.39 to − 0.43), articles of stone, plaster, cement (from 0.13 to − 0.16), albuminoidal substances (from 0.12 to − 0.07).

From 2001 to 2019, the US experienced a decline of comparative advantages in tobacco (from 0.77 to 0.11), fertilisers (from 0.36 to 0.03), salt, sulphur, earths and stone (from 0.2 to 0.04), essential oils, perfumery, cosmetics (from 0.39 to 0.18), miscellaneous edible preparations (from 0.55 to 0.26), explosives, pyrotechnic products (from 0.25 to 0.11), plastics (from 0.38 to 0.25). The US comparative advantages soared in ores (from 0.14 to 0.36), railway or tramway locomotives, railway or tramway track (from 0.14 to 0.33), cotton (from 0.52 to 1), photographic or cinematographic goods (from 0.28 to 0.34), pulp of wood or of other fibrous cellulosic material (from 0.46 to 0.67). For parts of aircraft and spacecraft, the US shifted from a comparative advantage (0.57) to a comparative disadvantage (− 0.31).

The results of the traditional symmetric Balassa index and the Lafay index for 2001 and 2019 are reported in Tables S3–S4 and Tables S5–S6, respectively (Online Supplementary Material).

### Similarity in the Structure of Comparative Advantages

Several studies have compared different classes of indices to measure trade similarity. De Benedictis and Tamberi (2000) carried out a comparison of the Italian pattern of comparative advantages in manufactures with the patterns of three high-wage countries (Japan, USA, and France) and three low-wage countries (Taiwan, Romania, and Thailand) in 1986 and 1996. At the 2-digit level of disaggregation (35 sectors), the Spearman correlation of Italian comparative advantages turned out to be significantly positive compared to Taiwan, Romania and Thailand, but significantly negative compared to Japan, USA and France. However, at the 4-digit level (538 sectors) most of the values of the Spearman coefficient turned out to be not significantly different from zero, except that with the US, which remained significantly negative, but with half the values at the 2-digit level of disaggregation.

De Benedictis and Tamberi (2004), applying kernel density estimation, concentration and Markov stationarity methods, showed that the Japanese structure of comparative advantages was characterised by a concentration on a few sectors, while Italy, Germany and France had a more diversified pattern of comparative advantages. De Benedictis and Tajoli (2004, 2007a, b, 2008) performed some very interesting comparative analyses on the patterns of manufactured exports of the Central-Eastern European Countries (CEECs) with respect to the pre-2004 European Union members (EU15). Some of the works concerned mainly the dynamics of manufactured exports of some of the CEECs (in particular Poland, Hungary, Romania and Bulgaria) and the EU15 over the period from 1989 to 2001. The second strand of analysis concerned the empirical relationship over the period from 1993 to 2002 of the convergence of the CEECs countries to the EU15 in terms of exports structures and income per capita. In this latter analysis, all the 10 CEECs countries were considered.

De Benedictis and Tajoli (2004, 2007a), in particular, examined how the export composition of Poland, Hungary, Romania and Bulgaria changed over time and how their export pattern changed with respect to the EU15 export composition. They analysed the dynamics of both the self-similarity (how the export composition of each country changed with respect to the beginning of the transition process) and EU-similarity (how the export pattern of each CEEC country changed with respect to the EU15 export pattern).

Poland, Hungary, Romania and Bulgaria changed their exports patterns towards the EU, but changes were different for every country according to their involvement in international production networks, through processing trade. Plotting the Pearson correlation coefficient rxy and the Bray–Curtis similarity index sxy^9^, changes in the CEECs export structure relative to their initial situation and the EU15 were more readily evident. These indices confirmed that many changes occurred in the pattern of export shares of the CEECs from 1989 to 2001, but important changes took place by the end of the 1990s. Both rxy and sxy showed that Poland moved away from its initial specialisation. The change in the Hungarian pattern of trade was even sharper, while the dynamics were different for Romania and Bulgaria. Romania changed more than Hungary and Poland, but most of the changes took place before 1992. Something similar occurred to Bulgaria, which changed especially at the beginning of the transition and, after a period of stability in the mid-1990s, moved again in 1999–2001; the extent of the change for Romania was stronger with rxy than with sxy. As the transition process went on, substantial differences emerged among the CEECs: Romanian and Bulgarian exports were concentrated in textiles, apparel and footwear, while Poland’s and Hungary’s exports grew in auto vehicles and machinery, despite the very large initial gap with the EU in these sectors. In this catching-up process, foreign capital and technological cooperation with EU firms played an important role.

De Benedictis and Tajoli (2004, 2007a) also examined whether the observed change in the CEECs export structures brought these countries closer to the EU structure. They found evidence that Romania presented a fluctuating path until 1995 and some convergence toward the EU export structure after 1997. Bulgaria’s export structure had been diverging from that of the EU, as it appeared from both rxy and sxy. Contrary to the other three cases, for Hungary, rxy and sxy were monotonically related only up to 1995. In terms of rxy, Hungary approached the EU and got even closer than Poland in 2001; but according to sxy, after a period of fast reduction in the distance from the EU, from 1995 onward Hungary reversed its trajectory.

The second strand of research examined the convergence in terms of exports structures and income per capita between the CEECs countries and the EU15 over 1993–2002. In this context, De Benedictis and Tajoli (2004, 2007b, 2008) compared the export patterns of the CEECs to those of the EU15, focusing on specialisation as suppliers for the EU market. Their main result was that similarity in export composition had a positive, significant and nonlinear impact on catching-up, and seemed to be driven by the growth of the main export market more than by other factors. Results were robust to controlling for openness and country size and investment, schooling, and the quality of institutions.

For this analysis, all the ten countries candidates for EU membership in the 1990s were considered: Bulgaria, Czech Republic, Estonia, Hungary, Latvia, Lithuania, Poland, Romania, Slovak Republic, and Slovenia. The average annual rate of catch-up in purchasing power standards showed large differences between these countries, not directly correlated with the starting point. Slovenia (one of the CEECs with the highest GDP per capita in 1993) and Estonia (one of the CEECs with the lowest GDP per capita in 1993) both showed a relatively high catch-up rate across the entire period. The Czech Republic, with an income level close to the EU average in 1993, had a low catch-up rate. The performance was also different across time. Bulgaria and Romania, ranking at the bottom of the group in 1993, showed a positive trend toward the EU average income only from 1998 onward.

For the CEECs and the EU, a correlation between convergence in export patterns and income levels per capita was quite robust since 2004 (De Benedictis and Tajoli 2004). De Benedictis and Tajoli (2007b, 2008) explored the mechanisms that might have given rise to it. The first channel between export patterns and income convergence links similarity in trade structure with productivity improvements since technological spillovers are more likely to occur within technologically similar industries. The second channel enhances the positive demand-side effects, which rely on trade volumes to exploit the scale effects built-in endogenous growth models. A third channel considers export diversification as a form of insurance against industry-specific adverse shocks. De Benedictis and Tajoli (2007b, 2008) documented that similarity in trade structure permits a reduction in income gaps and that higher similarity magnifies income convergence.

To evaluate the degree of similarity in the patterns of comparative advantages across countries, we have computed the Pearson correlation matrix^10^ of the Revealed Symmetric Augmented Comparative Advantage indices for 2001 and 2019 (Table 2).

**Table 2:** Correlation among RSACA Indices, selected countries; Source: Elaborations on UN COMTRADE data

Corr	BULG	CHIN	FRAN	GERM	INDO	ITA	JAP	KOR	PHIL	ROM	TAIW	THAI	UK	US	VIET
Year 2001															
BULG	1.00														
CHIN	0.32	1.00													
FRAN	− 0.20	− 0.14	1.00												
GERM	− 0.36	− 0.27	*0.52*	1.00											
INDO	0.22	0.33	*− 0.50*	*− 0.54*	1.00										
ITA	− 0.14	0.36	0.33	0.29	0.06	1.00									
JAP	− 0.35	− 0.30	0.38	***0.67***	*− 0.50*	0.25	1.00								
KOR	− 0.10	0.27	0.13	0.33	− 0.06	0.49	0.44	1.00							
PHIL	0.22	*0.52*	− 0.24	− 0.39	*0.56*	0.18	− 0.35	0.07	1.00						
ROM	***0.65***	0.33	− 0.19	− 0.31	0.29	− 0.01	− 0.24	0.03	0.44	1.00					
TAIW	− 0.26	0.35	− 0.01	0.15	0.11	*0.52*	0.23	***0.69***	0.14	− 0.04	1.00				
THAI	0.17	*0.57*	− 0.14	− 0.37	*0.56*	0.25	− 0.35	0.20	*0.54*	0.10	0.25	1.00			
UK	− 0.23	− 0.33	0.40	*0.58*	*− 0.59*	0.15	*0.50*	0.05	*− 0.52*	− 0.36	− 0.13	− 0.48	1.00		
US	− 0.33	*− 0.59*	0.33	*0.57*	*− 0.54*	− 0.13	0.33	− 0.17	− 0.48	− 0.39	− 0.24	− 0.48	0.46	1.00	
VIET	0.18	*0.59*	− 0.28	*− 0.58*	***0.69***	0.19	*− 0.57*	− 0.09	***0.65***	0.22	0.07	***0.65***	*− 0.52*	***− 0.60***	1
Year 2019															
BULG	1.00														
CHIN	− 0.25	1.00													
FRAN	− 0.02	− 0.33	1.00												
GERM	− 0.07	− 0.09	0.47	1.00											
INDO	0.12	− 0.05	− 0.31	− 0.41	1.00										
ITA	− 0.10	0.27	0.28	*0.50*	− 0.19	1.00									
JAP	− 0.05	− 0.13	0.36	***0.60***	− 0.44	0.20	1.00								
KOR	− 0.09	0.04	0.27	*0.53*	− 0.45	0.26	***0.70***	1.00							
PHIL	0.14	0.02	− 0.13	− 0.34	*0.51*	− 0.18	− 0.36	− 0.30	1.00						
ROM	0.24	0.01	− 0.07	0.15	0.23	0.22	− 0.01	0.06	0.18	1.00					
TAIW	− 0.32	0.43	− 0.05	0.28	− 0.31	0.29	0.37	*0.52*	− 0.22	− 0.02	1.00				
THAI	0.01	0.13	0.06	0.09	0.24	0.26	− 0.11	− 0.01	0.12	0.11	0.14	1.00			
UK	− 0.06	− 0.43	*0.53*	0.48	− 0.46	0.21	*0.50*	0.37	− 0.31	0.05	0.09	− 0.22	1.00		
US	− 0.02	*− 0.56*	0.38	*0.52*	− 0.27	0.03	0.42	0.17	− 0.31	− 0.01	0.00	− 0.12	*0.56*	1.00	
VIET	0.10	0.50	− 0.30	− 0.25	*0.50*	0.21	− 0.46	− 0.25	0.36	0.33	− 0.08	0.34	− 0.46	*− 0.53*	1

Correlation ≥ 0.50 in italics; correlation ≥ 0.60 in bold italics

The country pairs with the highest degree of similarity (Table 2) in comparative advantages are: (1) Korea and Taiwan with a correlation coefficient of 0.69 in 2001 and 0.52 in 2019; (2) Indonesia and Vietnam with a correlation coefficient of 0.69 in 2001 and 0.50 in 2019; (3) Germany and Japan with a correlation coefficient of 0.67 in 2001 and 0.60 in 2019; (4) the US and the UK with a correlation coefficient of 0.46 in 2001 and 0.56 in 2019. It is interesting to notice that the correlation coefficient between Romania and Bulgaria has significantly decreased from 0.65 in 2001 to 0.24 in 2019.

The correlation coefficient between Italy and Taiwan has dropped from 0.52 in 2001 to 0.29 in 2019. Likewise, the correlation coefficients between China and Thailand, China and the Philippines, and China and Pakistan have significantly fallen from values equal to or above 0.50 in 2001 to values less than 0.18 in 2019. This result implies that the export structure between these pairs of countries was somewhat overlapping in 2001, but it has significantly changed in 2019. Conversely, Japan and Korea have experienced a surge in similarity over time. Detailed information on the whole sample is presented in Tables S8 and S9 (Online Supplementary Material).

The US display a specialisation pattern more similar to Germany, Switzerland and the UK, but dissimilar from China, Indonesia and Bangladesh. Within the Euro Area, Germany registers a trade structure more similar to Japan, the UK and the US in 2001 and a stable trade structure with respect to Japan and the US in 2019. In 2001, Italy shows an export–import structure comparable to that one of Spain, Korea and Taiwan. In 2019, the Italian export–import structure is more alike to that of Turkey, Germany and Spain (Table 3).

**Table 3:** Top similar and dissimilar export structures, selected countries; Source: Elaborations on Tables S-8; S-10

Top similar for the US		Top dissimilar for the US	
2001	2019	2001	2019
Germany	UK	China	China
Switzerland	Germany	Indonesia	Bangladesh
UK/Netherland	Switzerland	Thailand/Philippines	Cambodia

Top similar for Germany		Top dissimilar for Germany	
2001	2019	2001	2019
Japan	Japan	Vietnam	Cambodia
UK	Korea	Indonesia/Tunisia	Bangladesh/Indonesia
US	US	Philippines	Ethiopia/Philippines

Top similar for Italy		Top dissimilar for Italy	
2001	2019	2001	2019
Taiwan	Germany	Norway	Norway
Korea	Turkey	Chile	Hong Kong
Spain	Spain	Ethiopia	Chile

Top similar for China		Top dissimilar for China	
2001	2019	2001	2019
Vietnam	Vietnam	US	US
Thailand	Taiwan	Switzerland	Norway
Philippines	India	UK/Sweden	UK/Sweden

The patterns of China’s comparative advantages are dissimilar from those of the US, the UK, Sweden, Switzerland and Norway (Table 3). The Netherlands and Belgium have strengthened their trade structure correlation between 2001 and 2019. Similarly, Finland and Norway have increased their similarity in trade specialisation during the considered years (Tables S8-S9, Online Supplementary Material).

To complete the preliminary analysis, we have calculated, for each specific country, the correlation coefficients between the RSACA indices in 2001 and the RSACA indices in 2019. In this way, it is possible to assess to which extent a country’s trade structure has changed between the two years (Table 4).

**Table 4:** Correlation (ρ) in the specialisation structure (RSACA) between 2001 and 2019; Source: Elaborations on UN COMTRADE data

Country	ρ	Country	ρ
Hong Kong	− 0.14	Czech Rep	0.73
Romania	0.54	Ireland	0.73
Hungary	0.55	Belgium	0.74
Singapore	0.56	Portugal	0.75
Poland	0.56	Spain	0.77
Malaysia	0.57	Bangladesh	0.77
Cambodia	0.62	Taiwan	0.80
Colombia	0.63	Switzerland	0.80
Ethiopia	0.63	Indonesia	0.80
Korea	0.63	India	0.80
Bulgaria	0.64	Turkey	0.81
Vietnam	0.64	Brazil	0.81
Philippines	0.66	France	0.82
Greece	0.66	Finland	0.83
Thailand	0.67	Pakistan	0.83
UK	0.67	Italy	0.85
Tunisia	0.69	Norway	0.85
Sweden	0.72	Chile	0.86
Netherland	0.72	US	0.87
Mexico	0.72	Germany	0.88
China	0.72	Japan	0.93

Hong Kong is the country that has undergone the largest structural trade shift: its trade pattern has changed radically (ρ =  − 0.14). Romania, Hungary, Singapore and Poland follow soon after. Japan, Germany, the US, Chile, Italy and Norway instead show a quite stable trade structure with correlation values equal to or larger than 0.85 (Table 4).

## Comparative Advantage Shifts

This section presents the theoretical model and the econometric analysis with the purpose to evaluate the patterns of comparative advantages for our sample of countries and products. Most empirical research in international trade had the objective to test whether the patterns of comparative advantages were in accordance with trade theories and, in particular, with the factor proportions theory. The methodology here adopted, instead, infers some commodity characteristics from the patterns of comparative advantage as revealed by trade flows and relative human capital or technology endowments of countries, considering as sufficiently proved empirically the correctness of the factor proportions theory in terms of human capital or technology variables. More specifically, our empirical analysis allows us to test if human capital and technology variables can be useful to distinguish between manufactures whose comparative advantages are positively associated with human capital and technology endowments of countries, manufactures whose comparative advantages are negatively related to human capital and technology endowments, and manufactures whose comparative advantages are not significantly associated with human capital and technology endowments. This assessment, at different points in time, enables us to detect any possible changes in the specialisation structure and the relative human capital or technology intensity of products.

### The Model

Using *j* subscripts to identify the country, *i* subscripts to identify the product and *t* subscripts to indicate the year, the specialisation equation takes the form:

5 $${S}_{jit}={\beta }_{0}+{\beta }_{1}{LC}_{jt}+{\beta }_{2}{Edu}_{jt}+{\beta }_{3}{Pat}_{jt}+{\beta }_{4}{Size}_{jt}+{\varepsilon }_{jt}$$

where *S* is the specialisation index and the explanatory variables *LC*, *Edu*, *Pat* and *Size*, indicate country *j*’s labour cost, education, patents per capita and home market size, respectively. Here, *β*_*0*_, *β*_*1*_*, β*_*2*_*, β*_*3*_ and *β*_*4*_ are the parameters to be estimated, and ${\varepsilon }_{jt}$ is the classical error term. *t* = 2001, 2009.

The theoretical framework of model (5) draws on the sizable number of theoretical and empirical analyses that, starting from the late 1950s, have proved the great importance of various components of human capital for the pattern of international specialisation in manufactures (e.g. Levchenko and Zhang 2016; Baldwin and Lopez-Gonzalez 2015; Mora 2002; Krugman 1998; Flam and Helpman 1987; Vollrath 1991; Hirsch 1974, 1965, 1967; Freeman 1963; Keesing 1966, 1971; most of the works included in Vernon 1970; Hufbauer 1970, 1966; Posner 1961; Hoffmeyer 1958).

Labour cost, the level of education of the labour force and patents per capita can be considered different indicators of a country’s human capital relative endowment. The number of patents per capita and the level of formal education are direct measures of human capital. In particular, the level of education is a measure of the resources devoted to improving the quality of workers, while the number of patents per capita is a measure of the success of a country in pursuing innovations.^11^ The price of labour can be considered as an indirect indicator of human capital or technological ability. A more educated and creative labour force can reach a higher equilibrium price in a competitive world environment. According to the factor proportions theory, countries with a relatively higher human capital or technology endowment would have a comparative advantage in human capital or technology-intensive products. Given the chief importance of learning by doing in these products (Cuaresma and Wörz 2005; Bensidoun et al. 2001; Feenstra and Rose 2000; Rosenberg 1970), countries specialised in these productions would raise their technological capabilities and the quality of exports. A possible explanation of the changes in the pattern of comparative advantages is the orthodox version of the product cycle model, which identifies the decline over time in the technological sophistication of products as the main source of specialisation changes (Aquino 1981; Vernon 1966; Hoffmeyer 1958). This explanation is also in line with the role of technology and innovation as an engine of comparative advantages (Rodrik 2018; Amendola et al. 1998; Posner 1961). New products are technology-intensive and on average high-income countries tend to have a comparative advantage in their production. As products grow older their technological sophistication decreases and comparative advantages tend to switch towards countries relatively well-endowed with factors other than highly qualified labour, i.e. low-wage countries. In the meanwhile, human capital rich countries can strengthen their comparative advantages in technology-intensive products. Put differently, the type of economies is often linked to the technology endowment. High-wage countries tend to have higher technological endowments and products will be characterised by higher-technology content (quality competitiveness), i.e. goods will be produced by workers paid with higher wages. Conversely, lower-wage countries have on average lower technology or human capital endowments and have a comparative advantage in goods produced by workers paid with lower wages (cost competitiveness).

Furthermore, model 5 includes home market size to account for scale economies as already emphasized by Adam Smith (1776) in his example of the British pin factory and afterwards by Ohlin (1933), Drèze (1960) and the new strand of trade theory (Feenstra 2018; Helpman 1981; Krugman 1979). It finally accounts for the fragmentation of the production activities by including a group of parts and components. With fragmentation, comparative advantages rise in finer stages of production and specialisation occurs in niche tasks.

### Data and Empirical Analysis

Our analysis focuses on 42 countries and 91 groups of tradable products in 2001 and 2019 so to detect possible changes in the first two decades of this century. The choice of our selected sample has been determined by geographical coverage, data availability and data reliability (Tables A1-A2, Appendix).

To estimate the parameters of model (5), we use, as a dependent variable, the indices of revealed comparative advantages, presented in Sect. 3.1. In what follows, we only present the results for the RSACA indices that, as mentioned in Sect. 3, capture specialisation fairly well. The explanatory variables comprise: (i) a wage variable proxied by country *j*’s labour costs per working hour (constant 2017 international $); (ii) a variable reflecting the education attainment, namely the percentage of the population aged 25–64 years having completed at least an upper secondary level of education; (iii) a variable somehow related to the theory of vertical differentiation and quality ladder, viz. per capita patents, or patents application normalised by country’s population and (iv) the size of country *j*’s home market measured by the real GDP evaluated at PPP (constant 2017 international $). The first three variables seize different traits of human capital. Indeed, following a tradition of empirical analysis (e.g. Amendola et al. 1998; Amable and Verspagen 1995; Pavitt 1988; Soete 1987), we assume that the technological activities of each country can be measured by their patenting activities that are an outcome of the human capital activity. From this perspective, patenting can be considered an indicator of the innovative capacities of a country, strongly linked to the quality of exports.^12^

All data have been extracted from the World Bank database, except for labour costs and trade flows that have been taken from ILO and UN COMTRADE, respectively.

To sum up, the cost of labour coefficient *β*_*1*_, the level of formal education coefficient *β*_*2*_, and the patents per capita coefficient *β*_*3*_ provide different measures of the impact of a country’s human capital and/or technology endowment on comparative advantages in the considered product. On the basis of their sign and statistical significance, products can be classified as high-technology (significantly positive coefficients) or low-technology (significantly negative coefficients). Statistically not significant coefficients can indicate either medium-technology products (Aquino^13^,^1981^; Balassa^14^,^1979^) or heterogeneous classes of products (Mora^15^,^2002^), comprising both high-technology and low-technology products.

Changes over time in the statistical significance of at least one human capital or technology coefficient point to a shift in comparative advantages and offer a rough signal of the value chain dynamics for each product. For example, a decline in the statistical significance of the labour cost coefficient from a significantly positive to a significantly negative value could reflect a shift of comparative advantages towards countries with lower labour costs. This suggests that specific activities, production stages and/or the entire productions have moved from high-wage to low-wage countries, reflecting most likely a reduction in the human capital-intensity or technology-intensity of the product.

As regards the scale-coefficient *β*_*4*_, a significantly positive value identifies products in which countries with a larger home market have a comparative advantage. Conversely, a significantly negative value characterises products in which countries with a smaller domestic market have a comparative advantage. A not significant *β*_*4*_ could identify products in which countries with a mid-size home market have a comparative advantage, or classes of products heterogeneous from the scale-economy point of view.

The descriptive statistics of the explanatory variables are sketched in Table 5.

**Table 5:** Descriptive statistics

Variable	Mean	Std. Dev	Min	Max
**2001**				
Labour cost	8.96	7.61	1.50	24.60
gdp_real_ppp	1.30E + 12	2.38E + 12	1.95E + 10	1.43E + 13
edu	45.84	24.53	6.42	85.48
patents_pop	21.06	52.40	0.00	301.43
**2019**				
Labour cost	17.98	15.10	2.00	50.60
gdp_real_ppp	2.45E + 12	4.65E + 12	7.24E + 10	2.25E + 13
edu	55.78	23.60	8.84	91.15
patents_pop	24.21	59.66	0.01	331.92

Note: labour costs per working hour (constant 2017 international $); real GDP evaluated at PPP (constant 2017 international $); edu = the percentage of the population aged 25–64 that completed upper secondary education; patents = patents application normalised by country’s population

A useful first impression of the relationships among our variables can be gleaned from the correlation matrix (Table 6). It emerges that our three human capital proxies are rather weakly correlated. Those measures, thus, capture empirically different aspects of the complex dimension of human capital.

**Table 6:** Correlation matrix

	Labour cost	edu	Patents	gdp_real
**2019**				
Labour cost	1			
edu	0.75	1		
Patents	0.44	0.45	1	
gdp_real	0.35	0.26	0.35	1
**2001**				
Labour cost	1			
edu	0.71	1		
Patents	0.21	0.28	1	
gdp_real	0.04	− 0.03	0.32	1

Note: labour costs per working hour (constant 2017 international $); real GDP evaluated at PPP (constant 2017 international $); edu= the percentage of the population aged 25–64 that completed upper secondary education; patents= patents application normalised by country’s population

### Estimation Results

Tables 7 and 8 report the cross-sectional estimations of model (5) for 2001 and 2019. Table 9 explicitly focuses on the class of intermediate products. We present the results for the RSACA indices used as the dependent variable.

**Table 7:** 2SLS estimates for trade specialisation 2001

Cod	Product	Labour cost		GDP real ppp		Edu		Patents pop		Cons	R-sq
b04	Dairy products	− 0.230	(0.155)	− 0.372	(0.758)	0.164***	(0.002)	− 0.264	(0.132)	− 0.571***	0.264
b05	Products of animal origin	− 0.191	(0.184)	0.659	(0.136)	− 0.043	(0.276)	− 0.391***	(0.000)	0.352*	0.370
b13	Lac; gums, resins	0.076	(0.742)	0.553	(0.393)	− 0.030	(0.625)	− 0.344***	(0.007)	− 0.034	0.110
b14	Vegetable plaiting materials	− 0.845***	(0.001)	0.062	(0.948)	0.054	(0.489)	− 0.242	(0.245)	0.618**	0.310
b15	Animal/vegetable fats, oils	0.083	(0.697)	− 0.644	(0.345)	0.013	(0.842)	− 0.131	(0.226)	− 0.191	
b16	Preparations of meat, fish, molluscs	− 0.692***	(0.001)	0.333	(0.707)	0.065	(0.302)	− 0.262*	(0.065)	0.469***	0.387
b17	Sugars and sugar confectionery	− 0.136	(0.619)	0.286	(0.786)	0.005	(0.955)	− 0.203	(0.189)	0.033	0.052
b18	Cocoa and cocoa preparations	− 0.062	(0.736)	0.392	(0.225)	0.016	(0.757)	− 0.254***	(0.000)	− 0.151	0.078
b19	Preparations of cereals, flour	− 0.121	(0.500)	0.901*	(0.062)	0.030	(0.583)	− 0.275**	(0.040)	− 0.146	0.070
b20	Preparations of vegetables, fruit, nuts	− 0.462**	(0.019)	0.688	(0.159)	− 0.003	(0.951)	− 0.399***	(0.000)	0.505***	0.468
b21	Miscellaneous edible preparations	0.057	(0.770)	1.047**	(0.043)	− 0.004	(0.934)	− 0.336***	(0.001)	− 0.200	0.184
b22	Beverages, spirits and vinegar	− 0.034	(0.877)	− 0.200	(0.649)	− 0.023	(0.725)	− 0.267***	(0.001)	0.229	0.192
b23	Residues from food industries	− 0.086	(0.659)	1.352	(0.177)	0.050	(0.286)	− 0.438***	(0.002)	− 0.594***	0.260
b24	Tobacco and manufactured tobacco	− 0.198	(0.386)	1.652**	(0.032)	0.072	(0.269)	− 0.545***	(0.000)	− 0.380*	0.145
b25	Salt; sulphur; lime and cement	− 0.358**	(0.024)	0.175	(0.754)	0.119**	(0.033)	− 0.239**	(0.016)	− 0.347	0.096
b26	Ores, slag and ash	− 0.168	(0.621)	− 1.013	(0.434)	− 0.019	(0.841)	− 0.319	(0.160)	0.226	0.055
b27	Mineral fuels, oils and products	− 0.325	(0.168)	− 0.454	(0.419)	0.151**	(0.040)	− 0.316***	(0.002)	− 0.669***	0.056
b28	Inorganic chemicals	0.042	(0.781)	0.458	(0.356)	0.064	(0.129)	− 0.251***	(0.004)	− 0.529***	0.225
b29	Organic chemicals	− 0.008	(0.961)	0.064	(0.876)	0.109***	(0.003)	− 0.085	(0.288)	− 0.689***	0.399
b30	Pharmaceutical products	0.456***	(0.004)	0.187	(0.840)	− 0.025	(0.606)	− 0.188	(0.152)	− 0.592***	0.239
b31	Fertilisers	0.149	(0.596)	− 0.125	(0.826)	0.104	(0.218)	− 0.206	(0.108)	− 0.904***	0.144
b32	Tanning extracts; paints and varnishes	0.595***	(0.001)	1.354	(0.113)	− 0.070	(0.222)	− 0.115	(0.371)	− 0.709***	0.135
b33	Essential oils; perfumery, cosmetics	0.103	(0.488)	1.422**	(0.012)	0.001	(0.979)	− 0.341***	(0.000)	− 0.455***	0.112
b34	Soap, washing, waxes, candles	0.321**	(0.035)	0.753**	(0.040)	− 0.023	(0.654)	− 0.130	(0.138)	− 0.479***	0.126
b35	Albuminoidal substances	0.945**	(0.048)	− 2.054	(0.523)	− 0.096	(0.337)	0.012	(0.972)	− 0.465	0.207
b36	Explosives; pyrotechnic products	− 0.006	(0.982)	2.799**	(0.024)	0.007	(0.940)	− 0.618***	(0.008)	− 0.367	
b37	Photographic or cinematog. goods	0.312	(0.136)	0.711	(0.106)	− 0.016	(0.778)	0.077	(0.616)	− 0.713***	0.303
b38	Miscellaneous chemical products	0.541***	(0.000)	− 0.311	(0.742)	− 0.010	(0.882)	− 0.071	(0.543)	− 0.726***	0.447
b39	Plastics and articles thereof	0.338***	(0.001)	0.236	(0.627)	− 0.025	(0.479)	0.060	(0.665)	− 0.444***	0.155
b40	Rubber and articles thereof	− 0.014	(0.920)	0.375	(0.473)	− 0.002	(0.955)	0.174	(0.133)	− 0.160	0.081
b41	Raw hides and skins	0.102	(0.666)	− 0.030	(0.972)	− 0.025	(0.711)	− 0.047	(0.766)	0.005	0.020
b42	Articles of leather; travel goods, bags	− 0.647***	(0.009)	2.151**	(0.030)	− 0.044	(0.576)	− 0.428**	(0.017)	0.734***	0.245
b43	Furskins and artificial fur	0.088	(0.435)	− 0.213	(0.592)	− 0.019	(0.532)	− 0.428***	(0.000)	0.137	0.391
b44	Wood and articles of wood	− 0.433**	(0.014)	− 0.578	(0.447)	0.130**	(0.010)	− 0.435***	(0.002)	− 0.051	0.327
b45	Cork and articles of cork	0.142	(0.432)	− 0.067	(0.850)	− 0.089**	(0.010)	0.021	(0.844)	0.015	0.167
b46	Manufactures of straw, basketware	− 0.855***	(0.002)	1.672*	(0.055)	0.034	(0.671)	− 0.493***	(0.001)	0.441*	0.287
b47	Pulp of wood cellulosic material	0.298	(0.407)	− 1.463	(0.110)	0.090	(0.390)	− 0.342*	(0.062)	− 0.738***	0.066
b48	Paper and paperboard	0.399**	(0.042)	0.058	(0.879)	− 0.009	(0.855)	− 0.014	(0.930)	− 0.530***	0.051
b49	Printing industry products	0.282*	(0.091)	0.148	(0.747)	− 0.047	(0.445)	− 0.111	(0.359)	− 0.160	
b50	Silk	0.049	(0.742)	1.024*	(0.089)	− 0.060	(0.215)	− 0.149	(0.140)	− 0.095	
b51	Wool, horsehair yarn and woven fabric	0.356***	(0.003)	− 0.159	(0.546)	− 0.047	(0.241)	− 0.117**	(0.021)	− 0.322***	0.067
b52	Cotton	0.489***	(0.005)	0.687	(0.352)	− 0.116**	(0.028)	− 0.126	(0.369)	− 0.175	0.161
b53	Other vegetable textile fibres	0.268	(0.209)	1.661*	(0.077)	− 0.039	(0.611)	− 0.444***	(0.005)	− 0.415	
b54	Man-made filaments and textiles	0.289*	(0.058)	0.552	(0.442)	− 0.035	(0.450)	0.239	(0.132)	− 0.449*	0.175
b55	Man-made staple fibres	0.524***	(0.000)	− 0.077	(0.947)	− 0.090	(0.106)	0.171	(0.262)	− 0.254	0.100
b56	Wadding, felt; special yarns; cordage	0.500***	(0.000)	0.684	(0.196)	− 0.128**	(0.031)	0.046	(0.802)	− 0.097	
b57	Carpets, other textile floor coverings	− 0.078	(0.727)	2.222**	(0.031)	− 0.107*	(0.097)	− 0.425**	(0.033)	0.302	
b58	Special woven fabrics; tapestries	0.564***	(0.001)	1.319	(0.196)	− 0.121*	(0.068)	− 0.006	(0.983)	− 0.322	
b59	Impregnated, laminated textile fabrics	0.903***	(0.000)	− 0.851	(0.554)	− 0.115	(0.172)	0.251	(0.405)	− 0.589***	0.100
b60	Knitted or crocheted fabrics	0.573**	(0.028)	− 0.048	(0.933)	− 0.136	(0.107)	0.333	(0.258)	− 0.164	0.136
b61	Apparels, knitted or crocheted	− 0.497***	(0.009)	1.384*	(0.060)	− 0.15***	(0.008)	− 0.242	(0.183)	1.339***	0.597
b62	Apparels, not knitted or crocheted	− 0.714***	(0.001)	1.581*	(0.062)	− 0.027	(0.692)	− 0.386**	(0.014)	0.884***	0.465
b63	Other made-up textile articles	− 0.300	(0.228)	1.870*	(0.055)	− 0.099	(0.196)	− 0.383	(0.129)	0.749**	0.204
b64	Footwear, gaiters and the like	− 0.132	(0.571)	1.836*	(0.067)	− 0.156**	(0.032)	− 0.363*	(0.094)	0.782***	0.147
b65	Headgear and parts thereof	− 0.302	(0.186)	1.355	(0.148)	− 0.113	(0.109)	− 0.036	(0.884)	0.787***	0.222
b66	Umbrellas, walking sticks	− 0.010	(0.956)	1.310	(0.283)	− 0.045	(0.450)	− 0.562***	(0.003)	− 0.084	
b67	Prepared feathers; artificial flowers	− 0.394*	(0.082)	1.503	(0.124)	− 0.033	(0.579)	− 0.228	(0.206)	0.237	
b68	Articles of stone, plaster, cement	0.337*	(0.053)	1.581*	(0.092)	− 0.118*	(0.071)	− 0.308**	(0.043)	0.032	
b69	Ceramic products	− 0.050	(0.803)	1.123	(0.100)	− 0.072	(0.278)	− 0.121	(0.489)	0.227	0.072
b70	Glass and glassware	0.061	(0.663)	0.497	(0.192)	0.018	(0.701)	− 0.070	(0.427)	− 0.277*	0.041
b71	Natural/cultured pearls, jewellery	− 0.445***	(0.001)	0.133	(0.648)	0.101**	(0.022)	− 0.162***	(0.010)	− 0.003	0.152
b72	Iron and steel	0.210	(0.261)	0.198	(0.740)	0.065	(0.236)	0.073	(0.469)	− 0.798***	0.260
b73	Articles of iron or steel	0.185	(0.243)	1.279	(0.112)	− 0.023	(0.701)	− 0.079	(0.589)	− 0.314	
b74	Copper and articles thereof	− 0.330	(0.143)	− 0.759	(0.244)	0.155**	(0.028)	0.160	(0.300)	− 0.479***	0.143
b75	Nickel and articles thereof	− 0.145	(0.456)	− 0.512	(0.471)	0.058	(0.226)	− 0.142	(0.453)	− 0.339**	
b76	Aluminium and articles thereof	0.128	(0.350)	0.529	(0.229)	0.041	(0.309)	− 0.372***	(0.000)	− 0.504***	0.164
b78	Lead and articles thereof	− 0.022	(0.932)	0.613	(0.656)	0.101	(0.164)	− 0.233	(0.223)	− 0.868***	0.112
b79	Zinc and articles thereof	0.072	(0.840)	− 0.506	(0.672)	0.123	(0.209)	0.036	(0.849)	− 0.894***	0.206
b80	Tin and articles thereof	0.005	(0.982)	0.787	(0.228)	− 0.063	(0.202)	− 0.230	(0.138)	− 0.066	0.052
b81	Other base metals; cermets	− 0.168	(0.381)	0.301	(0.689)	0.052	(0.277)	− 0.126	(0.444)	− 0.276**	0.002
b82	Tools, cutlery, spoons and forks	0.219	(0.222)	1.903**	(0.031)	− 0.067	(0.294)	− 0.057	(0.721)	− 0.309	
b83	Miscellaneous articles of base metal	0.338**	(0.012)	1.282*	(0.096)	− 0.085	(0.106)	− 0.122	(0.459)	− 0.198	
b84	Machinery, nuclear reactors	0.286**	(0.013)	0.225	(0.471)	− 0.011	(0.766)	0.045	(0.518)	− 0.437***	0.199
b85	Electrical machinery; recorders, tv	0.208**	(0.043)	0.298	(0.236)	− 0.029	(0.364)	0.040	(0.464)	− 0.203**	
b86	Railway or tramway locomotives; track	− 0.044	(0.832)	2.259**	(0.013)	0.026	(0.732)	− 0.144	(0.345)	− 0.643***	
b87	Vehicles other than railway or tram	0.246	(0.110)	0.547	(0.339)	− 0.069	(0.229)	0.327**	(0.032)	− 0.216	0.018
b88	Aircraft, spacecraft, and parts thereof	0.057	(0.737)	0.297	(0.646)	0.014	(0.771)	− 0.018	(0.852)	− 0.441***	0.075
b89	Ships, boats and floating structures	0.219	(0.298)	− 0.513	(0.511)	0.015	(0.809)	0.497***	(0.000)	− 0.412**	0.270
b90	Optical, photographic, medical tools	0.283***	(0.002)	0.503**	(0.047)	− 0.024	(0.363)	− 0.043	(0.516)	− 0.444***	0.388
b91	Clocks and watches and parts thereof	− 0.216*	(0.065)	0.811*	(0.084)	0.016	(0.689)	− 0.067	(0.421)	− 0.193	
b92	Musical instruments	− 0.121	(0.520)	1.253**	(0.028)	− 0.056	(0.383)	0.059	(0.651)	0.111	
b93	Arms and ammunition	0.300	(0.268)	1.064*	(0.072)	− 0.004	(0.964)	− 0.472***	(0.000)	− 0.410	
b94	Furniture; bedding, mattresses	− 0.332*	(0.086)	0.755	(0.307)	0.004	(0.940)	− 0.315***	(0.010)	0.392**	0.352
b95	Toys, games and sports requisites	− 0.244	(0.173)	1.235	(0.140)	− 0.043	(0.469)	− 0.104	(0.402)	0.342*	0.020
b96	Miscellaneous manufactured articles	0.208	(0.146)	1.271	(0.141)	− 0.045	(0.476)	0.024	(0.896)	− 0.356	
b97	Works of art and antiques	− 0.036	(0.632)	0.065	(0.564)	0.011	(0.534)	− 0.082**	(0.041)	0.021	0.075

Sample: 42 countries. Cod: SITC code. Dependent variable: RSACA. P-values in brackets; *p < 0.10, **p < 0.05, ***p < 0.01. Instrumented: labour cost; gdp real ppp Instruments: terms of trade, mobile, pop, r&d

**Table 8:** 2SLS estimates for trade specialisation 2019

Cod	Product	Labour cost		GDP real ppp		Edu		Patents pop		Cons	R-sq
b04	Dairy products	0.024	(0.760)	0.189	(0.427)	0.619	(0.114)	− 0.246**	(0.013)	− 0.56***	0.177
b05	Products of animal origin	− 0.030	(0.702)	0.310***	(0.005)	− 0.117	(0.742)	− 0.265**	(0.017)	0.024	0.116
b13	Lac; gums, resins	− 0.024	(0.823)	0.548***	(0.001)	− 0.125	(0.848)	− 0.250*	(0.077)	− 0.139	0.052
b14	Vegetable plaiting materials	− 0.265*	(0.060)	0.238**	(0.027)	0.894	(0.309)	− 0.563***	(0.000)	− 0.051	0.218
b15	Animal/vegetable fats, oils	− 0.104	(0.233)	− 0.372**	(0.024)	0.558	(0.193)	− 0.148**	(0.026)	− 0.103	0.093
b16	Preparations of meat, fish, molluscs	− 0.159	(0.111)	0.505***	(0.000)	− 0.124	(0.839)	− 0.344**	(0.023)	0.341	0.328
b17	Sugars and sugar confectionery	0.037	(0.729)	0.291	(0.272)	− 0.686	(0.290)	− 0.212**	(0.029)	0.266	0.061
b18	Cocoa and cocoa preparations	0.027	(0.645)	0.103	(0.124)	− 0.066	(0.838)	− 0.146**	(0.040)	− 0.148	0.043
b19	Preparations of cereals, flour	− 0.060	(0.362)	− 0.084	(0.691)	0.117	(0.799)	− 0.047	(0.550)	0.143	0.029
b20	Preparations of vegetables, fruit	− 0.110	(0.312)	0.168*	(0.060)	− 0.770	(0.206)	− 0.214*	(0.060)	0.682***	0.394
b21	Miscellaneous edible preparations	0.064	(0.302)	0.376*	(0.097)	0.198	(0.638)	− 0.172**	(0.028)	− 0.319	0.077
b22	Beverages, spirits and vinegar	− 0.032	(0.760)	− 0.163	(0.250)	− 0.079	(0.892)	− 0.090	(0.240)	0.160	0.043
b23	Residues from food industries	0.054	(0.531)	0.549**	(0.032)	0.463	(0.348)	− 0.366***	(0.004)	− 0.628***	0.162
b24	Tobacco and manufactured tobacco	− 0.092	(0.295)	0.386	(0.228)	0.424	(0.390)	− 0.229	(0.424)	− 0.089	0.063
b25	Salt; sulphur; lime and cement	− 0.028	(0.778)	− 0.067	(0.689)	0.021	(0.974)	− 0.080	(0.195)	0.014	
b26	Ores, slag and ash	− 0.140	(0.278)	− 0.254	(0.352)	0.332	(0.667)	− 0.466***	(0.000)	0.221	0.251
b27	Mineral fuels, oils and products	− 0.006	(0.941)	0.044	(0.677)	0.734*	(0.064)	− 0.208**	(0.031)	− 0.680***	0.147
b28	Inorganic chemicals	0.000	(0.995)	0.068	(0.595)	0.443	(0.272)	− 0.045	(0.337)	− 0.368**	0.082
b29	Organic chemicals	0.148**	(0.016)	0.234	(0.172)	0.097	(0.780)	0.037	(0.626)	− 0.594***	0.324
b30	Pharmaceutical products	0.099	(0.124)	0.415	(0.275)	0.285	(0.447)	− 0.232	(0.177)	− 0.568***	0.122
b31	Fertilisers	0.268**	(0.021)	− 0.037	(0.917)	− 0.237	(0.762)	0.043	(0.658)	− 0.683*	0.117
b32	Tanning extracts; paints	0.215***	(0.000)	0.526**	(0.035)	0.023	(0.951)	− 0.001	(0.988)	− 0.771***	0.425
b33	Essential oils; perfumery, cosmetics	0.007	(0.918)	0.121	(0.632)	0.463	(0.216)	0.108	(0.166)	− 0.451***	0.173
b34	Soap, washing, waxes, candles	0.019	(0.766)	0.287**	(0.028)	0.708*	(0.057)	0.018	(0.768)	− 0.643***	0.241
b35	Albuminoidal substances	0.277***	(0.001)	0.237**	(0.045)	− 0.259	(0.637)	− 0.037	(0.617)	− 0.634***	0.363
b36	Explosives; pyrotechnic products	0.065	(0.534)	0.775***	(0.005)	0.209	(0.733)	− 0.275	(0.160)	− 0.428*	0.026
b37	Photographic or cinematog. goods	0.076	(0.290)	− 0.008	(0.926)	0.414	(0.283)	0.234	(0.178)	− 0.779***	0.290
b38	Miscellaneous chemical products	0.085	(0.176)	0.423**	(0.011)	0.790**	(0.011)	− 0.096	(0.134)	− 0.896***	0.538
b39	Plastics and articles thereof	0.185**	(0.019)	0.057	(0.490)	− 0.498	(0.306)	0.194***	(0.000)	− 0.279	0.066
b40	Rubber and articles thereof	0.028	(0.727)	0.113	(0.403)	− 0.061	(0.902)	0.144**	(0.010)	− 0.152	0.031
b41	Raw hides and skins	0.093	(0.295)	− 0.071	(0.782)	− 0.279	(0.644)	0.001	(0.985)	0.010	0.083
b42	Articles of leather; travel goods, bags	− 0.133	(0.247)	0.263**	(0.034)	− 1.016	(0.129)	− 0.301***	(0.003)	0.799***	0.485
b43	Furskins and artificial fur	− 0.083	(0.110)	0.232***	(0.003)	0.460	(0.126)	− 0.349***	(0.000)	− 0.176	0.291
b44	Wood and articles of wood	− 0.108	(0.241)	− 0.013	(0.928)	0.768	(0.122)	− 0.404***	(0.001)	− 0.170	0.279
b45	Cork and articles of cork	0.084	(0.214)	− 0.050	(0.678)	− 0.838*	(0.084)	0.045	(0.505)	0.037	0.052
b46	Manufactures of straw, basketware	− 0.163*	(0.090)	0.680***	(0.000)	− 0.850	(0.177)	− 0.300	(0.101)	0.476*	0.293
b47	Pulp of wood cellulosic material	0.054	(0.685)	− 0.386*	(0.059)	1.262*	(0.087)	− 0.291***	(0.003)	− 0.767***	0.252
b48	Paper and paperboard	0.246**	(0.023)	0.461***	(0.001)	− 0.515	(0.362)	− 0.011	(0.916)	− 0.433*	
b49	Printing industry products	0.001	(0.988)	0.318***	(0.007)	0.648	(0.142)	− 0.119	(0.178)	− 0.537***	0.190
b50	Silk	0.155**	(0.033)	0.656***	(0.001)	0.026	(0.963)	− 0.177	(0.189)	− 0.718***	0.119
b51	Wool, yarn and woven fabric	− 0.032	(0.624)	0.291**	(0.014)	1.156***	(0.005)	− 0.277***	(0.000)	− 0.867***	0.261
b52	Cotton	0.162**	(0.015)	0.604**	(0.047)	− 0.140	(0.776)	− 0.188**	(0.049)	− 0.490*	0.217
b53	Other vegetable textile fibres	0.139**	(0.040)	0.248**	(0.036)	− 0.233	(0.537)	− 0.271***	(0.000)	− 0.274*	0.082
b54	Man-made filaments and textiles	0.208*	(0.094)	0.626***	(0.008)	− 0.328	(0.693)	0.100	(0.328)	− 0.654**	0.222
b55	Man− made staple fibres	0.155	(0.194)	0.594***	(0.006)	− 0.130	(0.866)	0.082	(0.411)	− 0.565*	0.223
b56	Wadding, felt; special yarns; cordage	0.237**	(0.025)	0.428***	(0.005)	− 0.695	(0.302)	− 0.023	(0.806)	− 0.316	
b57	Carpets, other textile floor coverings	0.103	(0.331)	0.710***	(0.010)	− 1.178*	(0.054)	− 0.205	(0.290)	0.173	0.087
b58	Special woven fabrics; tapestries	0.342**	(0.010)	0.536**	(0.021)	− 1.382	(0.130)	0.126	(0.329)	− 0.216	0.078
b59	Impregnated, laminat textile fabrics	0.357***	(0.001)	0.309**	(0.028)	− 0.887	(0.250)	0.182***	(0.010)	− 0.544*	0.227
b60	Knitted or crocheted fabrics	0.365**	(0.020)	0.324*	(0.079)	− 1.522	(0.141)	0.399***	(0.000)	− 0.184	0.183
b61	Apparels, knitted or crocheted	− 0.146	(0.292)	0.179	(0.129)	− 1.995**	(0.013)	− 0.141	(0.133)	1.419***	0.638
b62	Apparels, not knitted or crocheted	− 0.195	(0.126)	0.317***	(0.009)	− 1.232*	(0.088)	− 0.256***	(0.009)	1.033***	0.578
b63	Other made-up textile articles	− 0.049	(0.523)	0.526***	(0.001)	− 1.208**	(0.022)	− 0.165	(0.285)	0.623***	0.401
b64	Footwear, gaiters and the like	0.016	(0.884)	0.268***	(0.005)	− 1.71***	(0.010)	− 0.182**	(0.030)	0.832***	0.512
b65	Headgear and parts thereof	0.033	(0.681)	0.171	(0.293)	− 1.79***	(0.000)	0.021	(0.751)	0.834***	0.414
b66	Umbrellas, walking sticks	0.186***	(0.005)	0.485	(0.186)	− 1.183*	(0.067)	− 0.278 **	(0.040)	− 0.168	0.079
b67	Prepared feathers; artificial flowers	0.018	(0.801)	0.566***	(0.006)	− 1.3***	(0.009)	− 0.125	(0.369)	0.247	0.266
b68	Articles of stone, plaster, cement	0.010	(0.877)	0.466***	(0.001)	0.016	(0.972)	− 0.108*	(0.098)	− 0.222	0.070
b69	Ceramic products	0.029	(0.755)	0.727***	(0.000)	− 0.167	(0.803)	− 0.192**	(0.028)	− 0.336	0.094
b70	Glass and glassware	− 0.003	(0.961)	0.290***	(0.001)	0.330	(0.445)	− 0.021	(0.655)	− 0.413**	0.158
b71	Natural/cultured pearls, jewellery	− 0.135*	(0.061)	− 0.235*	(0.050)	0.293	(0.522)	− 0.014	(0.810)	0.228	0.043
b72	Iron and steel	0.160*	(0.056)	0.431**	(0.017)	0.253	(0.607)	0.054	(0.492)	− 0.784***	0.259
b73	Articles of iron or steel	0.181*	(0.062)	0.418**	(0.011)	− 0.682	(0.321)	0.032	(0.685)	− 0.191	
b74	Copper and articles thereof	− 0.140	(0.114)	− 0.31***	(0.003)	1.13**	(0.030)	0.044	(0.704)	− 0.366**	0.079
b75	Nickel and articles thereof	− 0.100	(0.266)	− 0.146	(0.250)	0.829	(0.131)	− 0.192***	(0.005)	− 0.395**	0.017
b76	Aluminium and articles thereof	0.064	(0.215)	0.469***	(0.001)	0.294	(0.380)	− 0.198*	(0.062)	− 0.542***	0.142
b78	Lead and articles thereof	0.015	(0.895)	− 0.117	(0.444)	0.414	(0.451)	0.064	(0.446)	− 0.393	0.084
b79	Zinc and articles thereof	0.080	(0.545)	− 0.006	(0.982)	0.737	(0.269)	0.212*	(0.087)	− 0.826***	0.234
b80	Tin and articles thereof	0.141*	(0.072)	− 0.091	(0.711)	− 0.643*	(0.072)	− 0.231***	(0.005)	− 0.053	0.076
b81	Other base metals; cermets	− 0.031	(0.638)	0.063	(0.663)	0.499	(0.161)	− 0.103	(0.177)	− 0.439***	0.077
b82	Tools, cutlery, spoons and forks	0.235**	(0.020)	0.322***	(0.007)	− 0.999	(0.138)	0.182***	(0.006)	− 0.194	
b83	Miscellaneous articles of base metal	0.140	(0.184)	0.401**	(0.010)	− 0.420	(0.552)	0.069	(0.469)	− 0.337	
b84	Machinery, nuclear reactors	0.118*	(0.051)	0.178**	(0.013)	− 0.134	(0.722)	0.092**	(0.042)	− 0.358***	0.037
b85	Electrical machinery; recorders, tv	0.055	(0.371)	− 0.068	(0.407)	− 0.240	(0.518)	0.151***	(0.000)	− 0.133	
b86	Railways or tramways; track	− 0.054	(0.447)	0.511***	(0.000)	0.672	(0.120)	0.034	(0.443)	− 0.658***	0.294
b87	Vehicles other than railway or tram	0.040	(0.639)	0.208	(0.413)	− 0.122	(0.805)	0.236**	(0.012)	− 0.238	0.092
b88	Aircraft, spacecraft, and parts	− 0.007	(0.916)	0.044	(0.750)	0.480	(0.278)	− 0.049	(0.279)	− 0.530**	0.079
b89	Ships, boats and floating structures	− 0.012	(0.893)	0.194*	(0.054)	0.198	(0.639)	0.363***	(0.000)	− 0.148	0.299
b90	Optical, photographic, medical tools	0.069	(0.237)	− 0.056	(0.351)	0.150	(0.660)	0.051	(0.239)	− 0.306**	0.130
b91	Clocks and watches and parts	0.100*	(0.085)	0.041	(0.542)	− 0.630*	(0.076)	− 0.135***	(0.001)	− 0.077	0.031
b92	Musical instruments	0.012	(0.899)	0.161**	(0.040)	− 0.633	(0.201)	0.068	(0.343)	0.086	0.159
b93	Arms and ammunition	0.080	(0.383)	0.122	(0.329)	− 0.357	(0.513)	− 0.062	(0.220)	0.044	0.042
b94	Furniture; bedding, mattresses	− 0.078	(0.297)	0.252	(0.208)	− 0.627	(0.234)	− 0.099	(0.238)	0.390*	0.366
b95	Toys, games and sports requisites	0.043	(0.604)	0.336*	(0.061)	− 1.12**	(0.042)	− 0.066	(0.263)	0.307	0.225
b96	Miscellaneous manufactured articles	0.119*	(0.088)	0.472***	(0.001)	− 0.331	(0.542)	− 0.023	(0.776)	− 0.353	
b97	Works of art and antiques	− 0.062*	(0.077)	0.042	(0.539)	0.090	(0.732)	− 0.095**	(0.025)	− 0.001	0.089

Sample: 42 countries. Dependent variable: RSACA. P-values in brackets; * *p* < 0.10, ** *p* < 0.05, *** *p* < 0.01. Instrumented: labour cost; gdp real ppp Instruments: terms of trade, mobile, pop, r&d

**Table 9:** 2SLS estimates for trade specialisation in the main intermediate products of Machinery and equipment (b84), Electrical machinery (b85), Motor vehicles (b87), and Aircraft (b88)

	b8473	b8542	b8708	b8803	b851770
	Parts and accessories (other than covers, carrying cases and the like) suitable for calculating machines and calculators	Electronic integrated circuits; parts thereof	Parts and accessories for tractors, motor vehicles for the transport of persons, …	Parts of aircraft and spacecraft of heading 8801 or 8802, n.e.s	Parts of telephone sets, telephones for cellular networks or for other wireless networks and …
**2001**					
Labour cost	− 0.004 (0.983)	1.048*** (0.008)	− 0.079 (0.628)	− 0.130 (0.460)	0.315** (0.011)
GDP real	0.746 (0.340)	1.296 (0.187)	0.497 (0.212)	− 0.023 (0.943)	0.253 (0.536)
Edu	− 0.068 (0.291)	− 0.182 (0.118)	0.019 (0.581)	0.081* (0.099)	− 0.048 (0.219)
Patents pop	0.060 (0.646)	− 0.019 (0.929)	0.220*** (0.003)	− 0.009 (0.927)	0.064 (0.674)
Cons	0.109 (0.513)	− 0.536 (0.128)	− 0.213** (0.021)	− 0.458*** (0.006)	− 0.378*** (0.009)
R-sq	0.097	0.041	0.274	0.061	0.027
chi2	5.623	16.861	40.042	4.987	9.477
**2019**					
Labour cost	0.160** (0.044)	0.211 (0.375)	− 0.033 (0.653)	− 0.126 (0.141)	0.108 (0.128)
GDP real	0.319 (0.121)	0.513*** (0.006)	0.034 (0.783)	0.134 (0.525)	− 0.271 (0.334)
Edu	− 1.260*** (0.007)	− 0.299 (0.839)	0.058 (0.881)	0.836* (0.091)	− 0.032 (0.941)
Patents pop	0.135* (0.073)	0.312*** (0.003)	0.264*** (0.000)	0.018 (0.799)	0.186*** (0.009)
Cons	0.144 (0.240)	− 0.585 (0.231)	− 0.096 (0.476)	− 0.236 (0.155)	− 0.371** (0.018)
R-sq	0.328	0.182	0.256		0.126
chi2	13.590	30.457	43.858	4.912	15.557

Sample: 42 countries. Cod: SITC code. Dependent variable: RSACA. P-values in brackets; *p < 0.10, **p < 0.05, ***p < 0.01. Instrumented: labour cost; gdp real ppp Instruments: terms of trade, mobile, pop, r&d

To deal with the potential endogeneity problem due to reverse causality, omitted variables and/or measurement errors, we use the instrumental variables two-stage least squares (2SLS) method. Standard OLS estimations, indeed, could lead to inconsistent estimates of the coefficients and the inference could be biased. To test the endogeneity of the regressors, we apply the post-estimation “estat endogenous” Stata command that implements the Durbin-Wu-Hausman test. The results lead to the rejection of the null hypothesis that labour costs, home market size and quality of exports are exogenous; hence they are endogenous and need some instrumental variables. On the contrary, we find education to be exogenous.

Relying on economic theory and data availability, we instrument the variable LC with the mobile cellular subscriptions (per 100 people), the variable home market size with population and terms of trade, and patents with R&D expenditure as a percentage of GDP.

Mobile cellular telephone subscriptions are subscriptions to a public mobile telephone service that provide access to the PSTN using cellular technology. The indicator, which includes the number of post-paid subscriptions and active prepaid accounts offering voice communications, has a strong correlation with labour costs.^16^ The terms of trade index, used along with population to instrument home market size, is calculated as the percentage ratio of the export unit value indexes to the import unit value indexes, measured relative to the base year 2000. Unit value indexes are based on data reported by countries that demonstrate consistency under UNCTAD quality controls, estimated and calculated by the UNCTAD as unit value indexes at the country level using the current year’s trade values as weights. Finally, the considered instrumental variable for patents is R&D expenditures, namely the gross domestic expenditures on research and development (R&D) expressed as a percentage of GDP. R&D covers basic research, applied research, and experimental development. All data for instruments have been collected from the World Bank.

Two types of information can be extracted from the estimations: (i) the human capital or technology intensity of each class of products and the localization of the production in 2001 and 2019, as revealed by the pattern of comparative advantages and the human capital or technology endowments of countries; (ii) the changes in these characteristics between 2001 and 2019.

Regarding the product characteristics, we propose to classify products into three main groups as outlined in Tables 10 and 11. The two tables report in brackets the explanatory variables with a coefficient significantly different from zero, together with the sign of the coefficient and the level of statistical significance indicated by the p-value*100.

**Table 10:** Classification of products, 2019

Human capital or skill intensive or high-technology products (31)	
Organic chemicals	(labour cost ( +), p-value: 1.6%)
Fertilizers	(labour cost ( +), p-value: 2.1%)
Tanning extracts, paints	(labour cost ( +), p-value: 0.00%)
Soap, washing, policing products	(education ( +), p-value: 5.7%)
Albuminoidal substances	(labour cost ( +), p-value: 0.1%)
Miscellaneous chemicals	(education ( +), p-value: 1.1%)
Plastics and articles thereof	(labour cost ( +), p-value: 1.9%; patents ( +), p-value: 0.00%)
Rubber and articles thereof	(patents ( +), p-value: 1.0%)
Articles of paper	(labour cost ( +), p-value: 2.3%)
Silk	(labour cost ( +), p-value: 3.3%)
Man-made filaments	(labour cost ( +), p-value: 9.4%)
Wadding, felt, special yarns	(labour cost ( +), p-value: 2.5%)
Special woven fabrics, tapestries	(labour cost ( +), p-value: 1.0%)
Impregnated, laminated textile fabrics	(labour cost ( +), p-value: 0.1%; patents ( +), p-value: 1.0%)
Textile fabrics, textile industrial use	(labour cost ( +), p-value: 0.1%; patents ( +), p-value: 1.0%)
Knitted or crocheted fabrics	(labour cost ( +), p-value: 2.0%; patents ( +), p-value: 0.00%)
Iron and steel	(labour cost ( +), p-value: 5.6%)
Articles of iron and steel	(labour cost ( +), p-value 6.2%)
Zinc and articles thereof	(patents ( +), p-value: 8.7%)
Copper and articles thereof	(education ( +), p-value: 3.0%)
Tools, cutlery of base metal	(labour cost ( +), p-value: 2.0%; patents ( +), p-value: 0.6%)
Machinery, nuclear reactors, parts	(labour cost ( +), p-value: 5.1%; patents ( +), p-value: 4.2%)
Electrical machinery and parts	(patents ( +), p-value: 0.00%)
Vehicles other than railway or tram	(patents ( +), p-value: 1.2%)
Ships, boats, etc	(patents ( +), p-value: 0.00%)
Miscellaneous manufactured articles	(labour cost ( +), p-value: 8.8%)
Electronic integrated circuits; parts thereof	(patents ( +), p-value: 0.3%)
Parts of telephone sets, or for other wireless networks	(patents ( +), p-value: 0.9%)
Parts and accessories for calculators	(labour cost ( +), p-value: 4.4%; patents ( +), p-value: 7.3%)
Parts and accessories for motor vehicles	(patents ( +), p-value: 0.00%)
Parts of aircraft and spacecraft	(labour cost ( +), p-value: 9.1%)

Human capital or skill not intensive or low-technology products (34)	
Dairy products	(patents (–), p-value: 1.3%)
Products of animal origin	(patents (−), p-value: 1.7%)
Lac; gums, resins	(patents (−), p-value: 7.7%)
Vegetable plaiting materials	(labour cost (−), p-value: 6.0%; patents (−), p-value: 0.00%)
Animal or vegetable fats and oils	(patents (−), p-value: 2.6%)
Preparations of meat and fish	(patents (−), p-value: 2.3%)
Sugars and sugar confectionery	(patents (−), p-value: 2.9%)
Cocoa preparations	(patents (−), p-value: 4.0%)
Preparation of vegetables	(patents (−), p-value: 6.0%)
Miscellaneous edible preparations	(patents (−), p-value: 2.8%)
Residues and waste from food industries	(patents (−), p-value: 0.4%)
Ores, slag and ash	(patents (−), p-value: 0.00%)
Articles of leather, handbags	(patents (−), p-value: 0.3%)
Manuf. furskin and artif fur	(patents (−), p-value: 0.00%)
Articles of wood	(patents (−), p-value: 0.1%)
Cork and articles of cork	(education (−), p-value: 8.4%)
Manuf. of straw	(labour cost (−), p-value: 9.0%)
Carpets and other textile floor coverings	(education (−), p-value: 5.4%)
Apparel, cloth, knitted, crocheted	(education (−), p-value: 1.3%)
Apparel, cloth, not knitted or crocheted	(education (−), p-value: 8.8%; patents (−), p-value: 0.9%)
Other textile articles; sets; worn clothing	(education (−), p-value: 2.2%)
Footwear and parts	(education (−), p-value: 1.0%; patents (−), p-value: 3.0%)
Headgear and parts thereof	(education (−), p-value: 0.00%)
Umbrella	(education (−), p-value: 6.7%; patents (−), p-value: 4.0%)
Prepared feathers; artificial flowers	(education (−), p-value: 0.9%)
Articles of stone, plaster, cement	(patents (−), p-value: 9.8%)
Ceramic products	(patents (−), p-value: 2.8%)
Natural/cultured pearls	(labour cost (−), p-value: 6.1%)
Nickel and articles thereof	(patents (−), p-value: 0.5%)
Aluminium and articles thereof	(patents (−), p-value: 6.2%)
Tin and articles thereof	(education (−), p-value: 7.2%; patents (−), p-value: 0.5%)
Clocks, watches and parts	(education (−), p-value: 7.6%; patents (−), p-value: 0.1%)
Toys, games, sports and parts	(education (−), p-value: 4.2%)
Works of art and antiques	(labour cost (−), p-value: 7.7%; patents (−), p-value: 2.5%)
**Medium technology or very heterogeneous classes of manufactured products (21)** **Indeterminate (5 with contrasting signs)**: 27. Mineral fuels; 47. Pulp of wood; 51. Wool, yarn; 52. Cotton; 53 Other veg. textile	

The explanatory variables with a coefficient significantly different from zero are reported in brackets, together with the sign of the coefficient and the level of statistical significance indicated as p-values*100

**Table 11:** Classification of products, 2001

Human capital or skill intensive or high-technology products (25)	
Organic chemicals	(education ( +), p-value: 0.3%)
Pharmaceutical products	(labour cost ( +), p-value: 0.4%)
Tanning or dyeing extracts	(labour cost ( +), p-value: 0.1%)
Soap, washing, policing products	(labour cost ( +), p-value: 3.5%)
Albuminoidal substances	(labour cost ( +), p-value: 4.8%)
Miscellaneous chemicals	(labour cost ( +), p-value: 0.00%)
Plastics and articles there of	(labour cost ( +), p-value: 0.1%)
Articles of paper	(labour cost ( +), p-value: 4.2%)
Products of the printing industry	(labour cost ( +), p-value: 9.1%)
Man-made filaments	(labour cost ( +), p-value: 5.8%)
Man-made staple fibres	(labour cost ( +), p-value: 0.00%)
Impregnated, coated,textile fabrics	(labour cost ( +), p-value: 0.00%)
Dairy products	(education ( +), p-value: 0.2%)
Knitted or crocheted fabrics	(labour cost ( +), p-value: 2.8%)
Copper and articles	(education ( +), p-value: 2.8%)
Miscellaneous articles of base metal	(labour cost ( +), p-value: 1.2%)
Machinery, nuclear reactors, parts	(labour cost ( +), p-value: 1.3%)
Electrical machinery and parts	(labour cost ( +), p-value: 4.3%)
Vehicles other than railway or tramway rolling stock	(patents ( +), p-value: 3.2%)
Ships, boats, floating structures	(patents ( +), p-value: 0.00%)
Optical, photographic, medical instruments	(labour cost ( +), p-value: 0.2%)
Electronic integrated circuits; parts thereof	(patents ( +), p-value: 0.8%)
Parts of telephone sets, or for other wireless networks	(patents ( +), p-value: 1.1%)
Parts and accessories for motor vehicles	(patents ( +), p-value: 0.3%)
Parts of aircraft and spacecraft	(labour cost ( +), p-value: 9.9%)

Human capital or skill not intensive or low-technology products (35)	
Products of animal origin	(patents (−), p-value: 0.00%)
Lac; gums, resins	(patents (−), p-value: 0.7%)
Vegetable plaiting materials	(labour cost (−), p-value: 0.1%)
Preparations of meat and fish	(labour cost (−), p-value: 0.01%; patents (−), p-value: 2.3%)
Cocoa preparations	(patents (−), p-value: 4.0%)
Preparations of cereals, flour	(patents (−), p-value: 4.0%)
Preparations of vegetables, fruit, nuts	(labour cost (−), p-value. 1.9%; patents (−), p-value: 0.00%)
Miscellaneous edible preparations	(patents (−), p-value: 0.00%)
Beverages, spirits and vinegar	(patents (−), p-value: 0.00%)
Residues from food industries	(patents (−), p-value: 0.00%)
Tobacco	(patents (−), p-value: 0.00%)
Salt; sulphur; lime and cement	(labour cost (−), p-value: 2.4%; edu ( +) p-value: 3.3%; patents (−), p-value: 1.6%)
Inorganic chemicals	(patents (−), p-value: 0.4%)
Essential oils, perfumery, cosmetics	(patents (−), p-value: 0.00%)
Explosives	(patents (−), p-value: 0.8%)
Articles of leather, handbags	(labour cost (−), p-value: 0.9%; patents (−), p-value: 1.7%)
Manuf. furskins and artificial fur	(patents (−), p-value: 0.05%)
Articles of wood	(labour cost (−), p-value: 1.4%; patents (−), p-value: 0.2%)
Cork and articles of cork	(education (−), p-value: 1.00%)
Manufactures of straw, basketware	(labour cost (−), p-value: 0.2%; patents (−), p-value: 0.1%)
Pulp of wood	(patents (−), p-value: 6.2%)
Other vegetable textile fibres	(patents (−), p-value: 0.5%)
Carpets and other textile floor coverings	(education (−), p-value: 9.7%; patents (−), p-value: 3.3%)
Apparel, knitted or crocheted	(labour cost (−), p-value: 0.9%; education (−), p-value: 0.8%)
Apparel, not knitted or crocheted	(labour cost (−), p-value: 0.1%; patents (−), p-value: 1.4%)
Footwear and parts	(education (–), p-value: 3.2%; patents (−), p-value: 9.4%)
Umbrellas, walking sticks	(patents (−), p-value: 0.3%)
Prepared feathers; artificial flowers	(labour cost (−), p-value: 8.2%)
Articles of stones	(labour cost ( +), p-value: 5.3%; edu (−), p-value: 7.1%; patents (−), p-value: 4.3%)
Natural/Cultured pearls	(labour cost (−), p-value: 0.1%; edu ( +), p-value: 2.2%; patents (−), p-value: 1.0%)
Aluminium and articles thereof	(patents (−), p-value: 0.5%)
Clocks, watches and parts	( labour cost (−), p-value: 6.5%)
Arms and ammunitions	(patents (−), p-value: 0.5%)
Furniture	(labour cost (−), p-value: 8.6%; patents (−), p-value: 1.0%)
Works of art and antiques	(patents (−), p-value: 4.1%)
**Medium technology or very heterogeneous classes of manufactured products (26)** **Indeterminate (5 with contrasting signs):** 27. Mineral fuels; 51. Wool, yarn; 52. Cotton; 56. Wadding, felt; 58. Special woven fabrics	

The explanatory variables with a coefficient significantly different from zero are reported in brackets, together with the sign of the coefficient and the level of statistical significance indicated as p-values*100

In 2019, comparative advantages in 31 classes of products turned out to be positively associated^17^ with at least one measure of a country’s human capital or technology endowment (high technology products); comparative advantages in 34 classes of products proved to be negatively related to at least one measure of human capital or technology endowment (low technology products). This result is consistent with the Heckscher-Ohlin theorem and other literature (e.g. Cali et al. 2016; Miroudot and Squicciarini 2015) that documents how skills are a key factor for countries to raise domestic value content in their exports. It is also in line with the studies by Mora (2002), Harrigan (1997) and Dollar (1993) that indicate technological differences and complementary technical labour as a source of comparative advantage and international specialisation. Comparative advantages in 21 products did not result in any statistically significant association with human capital or technology endowment (medium-technology or heterogeneous classes of products). Contrasting statistically significant coefficients were obtained for 5 products (Table 10).

In 2001, comparative advantages in 25 classes of products have been positively associated with^18^ at least one measure of human capital or technology endowment (high technology products); comparative advantages in 35 products exhibited a negative association with at least one measure of human capital or technology endowment (low technology products). Comparative advantages in 26 products have not been found significantly related to any measure of human capital or technology endowments (medium-technology or heterogeneous classes of products). Contrasting statistically significant coefficients were registered for 5 products (Table 11).

In particular, high-human capital intensive products – identified by significantly positive estimates of at least one human capital-coefficient in both 2001 and 2019 – are organic chemicals; tanning, paints and varnishes; albuminoidal substances; soaps; plastics and articles thereof; paper, paperboard and articles thereof; man-made filaments; copper and articles thereof; machinery, mechanical appliances, nuclear reactors, boilers; electrical machinery; vehicles rather than railways and tramways; ships and boats. This means that the production of these goods is located mainly in countries with a relatively higher human capital or technology endowment, most likely because they are intensive of human or technological capital (Tables 7 and 8).

Low-technology or human capital products—identified by significantly negative estimates of at least one of the human capital or technology endowment coefficient in 2001 and 2019—include products of animal origin; lac, gums; vegetable plaiting materials; preparations of meat, fish; preparations of cocoa; vegetable preparations; miscellaneous edible products; residues food industries; furskins; prepared feathers; umbrella; clocks and watches; works of art. This suggests that the production of these goods can be found in countries with a relatively lower human capital or technology endowment since they have a low-human capital or technology intensity (Tables 7 and 8). This result is consistent with the analysis by the World Bank (2016) that showed that recent trade dynamics generate an adverse impact on the comparative advantages of developing countries. Global value chains, in fact, demand skills and capabilities that are in short supply in developing countries and weaken their traditional comparative advantage in unskilled labour (Rodrick 2018).

Medium-human capital intensive or heterogeneous products—identified by statistically not significant coefficients in 2001 and 2019—embrace photographic goods; raw hides, skins and leather; glass and glassware; lead; base metals; railways and tramways; aircraft and parts thereof; musical instruments (Tables 7 and 8).

With reference to intermediate products (Table 9), electronic integrated circuits, parts and accessories for motor vehicles, parts of aircraft and spacecraft, and parts of telephone sets or other wireless networks turned out to be technology-intensive both in 2001 and in 2019.

**Fig. 1:**
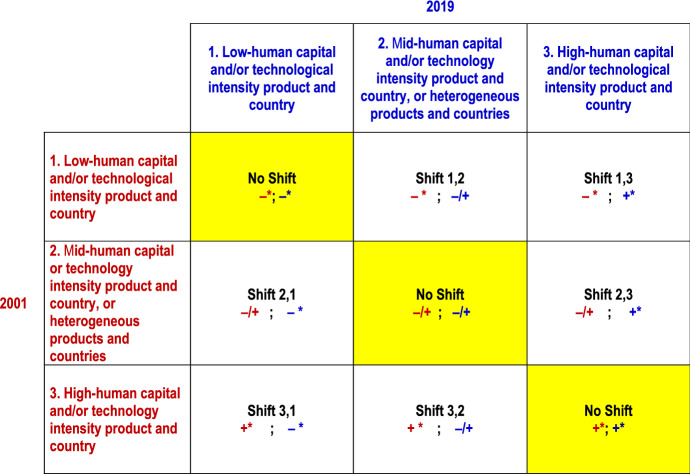
The comparative advantage shift-matrix

To evaluate more specifically the shifts in comparative advantages revealed by the human capital coefficient behaviour, we construct the comparative advantage shift-matrix (Fig. 1).

**Fig. 2:**
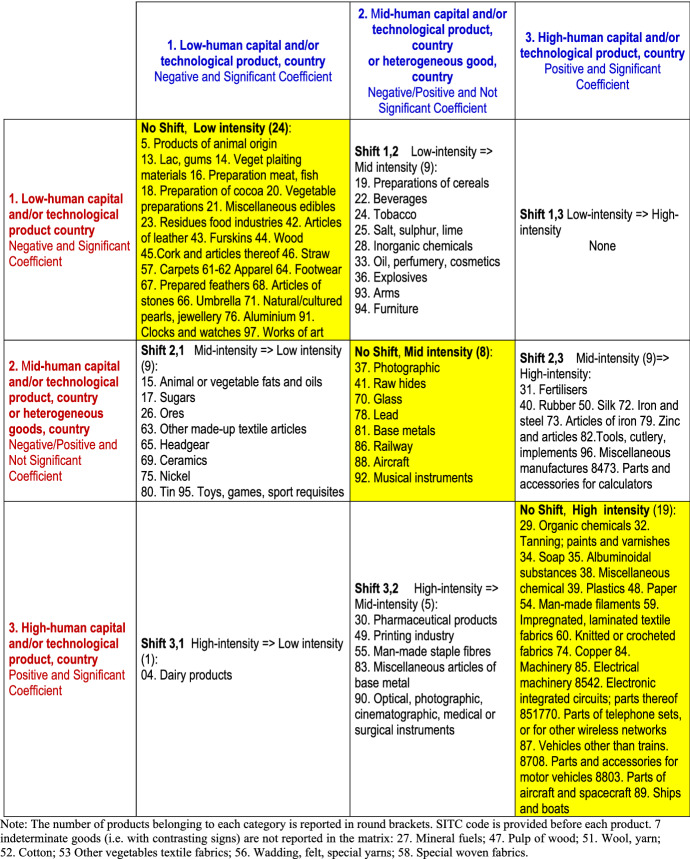
The comparative advantage shift-matrix 2001-2019

This matrix emphasises the movements in manufacturing sectors across countries over time. The matrix of Fig. 2 shows 3 × 3 possible situations that can be encountered when analysing the combinations of the product content, the statistical significance and the sign of at least one human capital or technology coefficient of the model. The first positions (in red) in each cell refer to year 1, in our case 2001; the second positions (in blue) refer to year 2, i.e. 2019. The signs of the human capital coefficients can be negative and significant (-*) implying a low-human capital and/or low technology product and country when there is at least one significantly negative coefficient; positive and significant (+ *) implying a high-human capital and/or high technology product and country when there is at least one significant positive coefficient; not significant (− or +) implying a mid-human capital, mid-technology product and country or heterogeneous products and country^19^ when there are not significant coefficients. When the sign of significant coefficients diverges we consider the prevailing sign among the three coefficients. Few cases remained undetermined. Figure 2 reports the detailed results.

The empirical evidence suggests an increasing statistical significance of human capital-coefficients for some products and a decreasing statistical significance of human capital-coefficients for other products between 2001 and 2019 (Fig. 2).

An increase over time of the statistical significance of human capital or technology coefficients points to a shift of comparative advantage towards countries with higher human capital or technology endowments. A decline over time of the human capital or technology coefficients suggests a shift of comparative advantage towards countries with lower human capital or technology endowments. In particular, products above the bisector of the matrix show a shift towards a higher human capital or technology intensity and their production would be localized in countries with higher human and technological capital. Products below the bisector highlight movements towards a lower human capital or technology intensity and thus a production localized in countries with lower human and technological capital endowments.

Between 2001 and 2019, the estimates reveal a shift from the class of mid-technology or heterogeneous products to the class of high-technology products for fertilizers; rubber; silk; articles of iron, articles of steel; articles of zinc; tools and cutlery and miscellaneous manufactures.

While in 2001 the human capital or technology coefficients were significantly negative for a set of products (i.e. beverages; preparation of cereals; tobacco; inorganic chemicals; explosives and pyrotechnic products; pulp of wood; vegetable textile fibres; arms and ammunitions), they turned out to be not significant in 2019.

The human capital or technology intensity has decreased from high to mid-level for pharmaceutical products; printing industry; miscellaneous articles of base metals; optical, photographic, cinematographic, medical or surgical instruments.

The human capital or technology intensity has dropped from mid to low-level for animal or vegetable fats and oils; sugars; ores; other made-up textile articles; ceramics; nickel; tin and related products; toys, games, sport equipment.

All products along the main diagonal (bold italic cells) of the matrix have not registered any shift in their human capital or technology intensity between 2001 and 2019. Such a finding would lend support to the analysis by Krugman (1987) in which the patterns of specialisation, once established, tend to persist and extend over time. Thus, for instance, vehicles other than tramways and railways, ships and boats; machinery, mechanical appliances, miscellaneous chemicals and the majority of the considered intermediates enter this category.

Regarding the scale-coefficients, a significantly positive value identifies products in which countries with a larger home market have a comparative advantage (Weder 2003; Davis and Weinstein 2003). This is the case, for instance, of railway or tramway locomotives, rolling stock, miscellaneous chemicals, tools and cutlery, articles of leathers, soap and explosives both in 2001 and 2019. Economies of scales have become less relevant for aircraft, spacecraft and part thereof; vehicles rather than railways and optical products in 2019. They have become more relevant for iron, aluminium and steel; ships, boats and floating structures and machinery, man-made filaments, paperboard and electronic integrated circuits. The scale-coefficient is significantly negative for copper, pulp of wood, cultured pearls in 2019, indicating that countries with a larger home market have a comparative disadvantage in these products.

According to Hufbauer (1970) and Katrak (1973), a significantly positive scale-coefficient would identify a product characterised by relatively larger scale economies. According to Drèze (1960), instead, this would identify a product which not only has relatively larger scale economies but is also characterised by a relatively low degree of standardisation of international demand, i.e. a product for which only the countries with a larger home market can take the full benefits of scale economies.

## Conclusions

The present study has examined the patterns of comparative advantages in manufactures for 91 (final and intermediate) products and 42 countries in 2001 and 2019. As a measure of comparative advantages, we have adopted the difference between the symmetric Balassa index for exports minus the symmetric Balassa index for imports, whose values range from – 2 to 2. For each product, this index of comparative advantages has been related to three measures of a country’s human capital endowment—the cost of labour, the level of formal education and the number of patents per capita—and a measure of home market size, mainly added as a control variable. The cross-sectional estimates show that comparative advantages in most of the manufactures are significantly associated with inter-country differences in human capital endowments and/or innovative performance of countries, as well as a country’s home-market size.

In 2019, comparative advantages in 65 products are related to the human capital or technology endowment. Specifically, comparative advantages in 31 products turned out to be positively and significantly associated with at least one of the three indicators of a country’s human capital endowment or technology. Comparative advantages in 34 products were negatively and significantly associated with at least one indicator of the human capital or technology endowment. For five products, one of the human capital coefficients was significantly positive, but another one was significantly negative; for 21 products, none of the human capital or technology coefficients turned out to be significantly different from zero. These results provide additional evidence of the importance of human capital or technology for international specialisation in manufactures. From 2001 to 2019, 33 products shifted from one to another class, 51 products presented no shifts, while 7 products remained undetermined. Delving more deeply into the parts and components of final goods, we find that fragmentation seems to be related to a magnification of comparative advantages as countries tend to specialise in activities that require more skilled workers.

The possibility of distinguishing between high and low-technology products could have some interesting policy implications, since high-technology products seem to create a higher rate of learning by doing externalities, and hence contribute more to the upgrade of the human capital or technology endowment of a country.

Despite the usual caveat related to the hypotheses of the model, the results regarding the behaviour of the labour cost, level of education and patents coefficients have been rather interesting. For 17 classes of products, the results point to shifts of some of the human capital coefficients from significantly negative or non-significantly different from zero values to significantly positive values, suggesting a shift over time of comparative advantages towards higher-technology countries. A reverse pattern has been registered for 15 classes of products that shifted towards lower-technology countries. This stems primarily from the fact that innovation and technological capital entail progressive improvements in the quality of products and the fragmentation of production across borders generates a finer international division of labour and greater specialisation in tasks.

The main implication of these results is that the shifts of productive activities towards low-wage countries are less pervasive and disruptive than one would expect under the product-life cycle model. The reason is due to the characteristics of the current international specialisation that takes place more and more at the level of different productive phases of increasingly complex products and production processes rather than at the final-product level. Hence, public policy should be directed to support firms to upgrade the quality of existing products and foster the level of education and training. In general, the satisfactory international performance of most high wage countries is quite clear by comparing the trade dynamics and current account balances of high wage and low wage countries.

According to the OECD estimates (Economic Outlook, May 2021), in 2019 (the most recent normal year before the Covid pandemic) the greatest surpluses in current account balances as a percentage of GDP have been registered by high-wage countries such as the Netherlands (9.9%), Switzerland (6.7%), Ireland (9.1%), Norway (8.1%), Germany (7.24%), Denmark (8.9%), Korea (3.6%), Japan (3.45%). Italy has registered on average in 2019 a current account surplus of 3.3% of GDP, but if we consider only the northern regions, the surplus would have been more than 8% of GDP. Among high-wage countries, a significantly negative current account balance has been registered in 2019 only by the US (− 2.2%) and the UK (− 3.1%). On the other side, most low-wage countries have registered in 2019 a negative or near to zero current account balance: Argentina − 0.75%, Turkey − 3.1%, Greece − 1.5%, India − 0.8%, Slovak republic − 2.7%, Poland 0.5%, Portugal − 0.4%, China 0.73%. These data are dramatically inconsistent with the impression of a large scale delocation process from high-wage towards low-wage countries!

### Electronic supplementary material

Below is the link to the electronic supplementary material.

Supplementary file1 (DOCX 636 KB)

## Appendix

See Table A1.

**Table A1:** Country sample

Bangladesh	Korea, Republic of
Belgium	Malaysia
Brazil	Mexico
Bulgaria	Netherlands
Cambodia	Norway
Chile	Pakistan
China	Philippines
Colombia	Poland
Czech Rep	Portugal
Ethiopia	Romania
Finland	Singapore
France	Spain
Germany	Sweden
Greece	Switzerland
Hong Kong SAR, China	Taiwan
Hungary	Thailand
India	Tunisia
Indonesia	Turkey
Ireland	UK
Italy	USA
Japan	Vietnam

## Footnotes

1 About one-half of world trade occurred over the last ten years through Global Value Chains, in which products cross at least one border, and typically many borders (World Bank 2020). However, the growth of GVCs has lost momentum after the financial crisis of 2009 and has been hit hard by the COVID-19 pandemic (Javorcik 2020; World Bank 2020; OECD 2019; Ferrantino and Taglioni 2014; Hoekman 2015; Baldwin 2011).

2 According to World Trade Organization (2019, p. 12), in 1995, purely domestic production activities accounted for about 85% of world GDP, and traditional trade activities for about 5.8% of world GDP; 6.5% was accounted by simple GVC and 2.7% by complex GVC. From 1996 to 2008 the share of complex GVC increased to about 4.5%; the share of simple GVC to 8.5%; the share of traditional trade activities increases to about 8%, while the share of domestic production activities decreased to about 79% of world GDP. After the great financial crisis, GVC production activities registered a decrease with a partial recovery in 2017. In 2017 the share of complex GVC production activities was at about the same level as in 2008. The shares of simple GVC and traditional trade were still somewhat smaller than in 2008, and the share of domestic production activities was a bit higher at about 80.5% of world GDP.

3 Defined as the sum for all products of the absolute values of the differences between the export shares of the patterns to be compared, divided by the sum for all products of the same export shares.

4 See also the studies by Iapadre (2001), De Benedictis and Tamberi (2004), De Benedictis (2005).

5 See Algieri (2007).

6 Empirical studies of outsourcing as a worldwide phenomenon have been hampered by a lack of systematic data distinguishing between trade in final goods and trade in parts and components. Moreover, the relevance and detailed characteristics of international fragmentation of production are difficult to measure, as a proper analysis of the phenomenon would require firm-level data, to appropriately follow the sequence of phases in the production processes.

7 In our sample of products and countries, the values of the Lafay index and our RSACA index turned out to be highly positively correlated across countries but not across products (Table S7, online Supplementary material). This means that the results of our analysis would not change significantly when using the RSACA index instead of the Lafay index.

8 Some of the intermediate goods are typical of the traditional fragmentation of production (i.e. lac; gums, resins and other vegetable saps and extracts; Salt; sulphur; earths and stone; plastering materials, lime and cement; Ores, slag and ash; Mineral fuels, mineral oils and products of their distillation; bituminous substances; mineral waxes; Inorganic chemicals; organic or inorganic compounds of precious metals, of rare-earth metals, of radioactive elements or of isotopes; Organic chemicals; Fertilisers; Tanning or dyeing extracts; tannins and their derivatives; dyes, pigments and other colouring matter; paints and varnishes; putty and other mastics; inks; Albuminoidal substances; modified starches; glues; enzymes; Raw hides and skins and leather; Wool, fine or coarse animal hair; horsehair yarn and woven fabric; Cotton; Other vegetable textile fibres; paper yarn and woven fabrics of paper yarn; Man-made filaments; strip and the like of man-made textile materials; Man-made staple fibres; Wadding, felt and nonwovens; special yarns; twine, cordage, ropes and cables and articles thereof; Iron and steel). Other intermediate goods are connected to the new type of international fragmentation that registered rapid growth from 1990 to the great financial crisis of 2007–2008. This is the case of parts, components and equipment of electrical and not electrical machinery, motor vehicles, aircraft and telephone sets (i.e. Parts for calculating machines; Electronic integrated circuits; Parts and accessories suitable for use solely or principally with calculating machines and calculators; Electronic integrated circuits; parts thereof; Parts and accessories for tractors, motor vehicles for the transport of ten or more persons, motor cars and other motor vehicles principally designed for the transport of persons, motor vehicles for the transport of goods and special purpose motor vehicles; Parts of aircraft and spacecraft; Parts of telephone sets, telephones for cellular networks or other wireless networks and of other apparatus for the transmission or reception of voice, images or other data).

9 The Bray–Curtis metrics is an index of distance, hence of dissimilarity; the correspondent index of similarity was obtained as sxy = 1 – the Bray–Curtis metrics; both for rxy and sxy a value of 1 represented identity with the initial situation or with the EU, and a lower value indicated the extent of the difference.

10 De Benedictis and Tajoli (2004, 2007a, 2007b) have shown that the Pearson correlation coefficient could have some shortcomings as a measure of similarity in case of strongly asymmetric distributions. Since we have verified that the distributions of our indices presented neither strong asymmetries nor frequent cases of “overshoots”, we are fairly confident about the appropriateness of the Pearson correlation coefficient as a measure of inter-country similarity of the patterns of comparative advantages. The frequency distributions of the RSACA indices and the truncated normality tests for a selected number of countries are reported in the online supplementary material (Figure S1). The results for other countries are provided upon request.

11 Alternately, the real capital stock has been used as a product innovation proxy (e.g. Algieri 2014; Madden et al. 1999).

12 Andersson and Ejermo (2008), for example, examine to which extent technology specialisation, measured by citation-weighed patents, affects trade flows and they find that regions with higher technology specialisation export products of higher quality.

13 “… explaining the pattern of specialisation in each group of products at any moment in time, does *not* mean that the estimated coefficients can be expected to be statistically significant for all industries and all years. … It does imply instead that, at any moment in time, there are some products (high-technology products) whose pattern of comparative advantage is significantly and positively related to wages, some products (low-technology products) whose pattern of specialisation is significantly and negatively related to wages, and some other products (medium-technology products) whose pattern of specialisation is not related in a statistically significant way to wages” (Aquino, 1981 p. 47).

14 “…it should be added that coefficient values near to zero have an economic interpretation even if they are not significantly different from zero; they indicate that a country is at the dividing line as far as comparative advantage in capital- and labour-intensive products is concerned” (Balassa, 1979 p. 262).

15 “…None of the variables [non-metallic mineral product, other manufacturing products] are significant due to the heterogeneity of production considered in these branches (Mora 2002, p. 311)”.

16 We have also considered other instruments such as broadband connections, the value-added of the secondary sector, but their correlation was lower than mobile subscriptions.

17 with a p-value of at least 10%

18 with a p-value of at least 10%.

19 Inconclusive results could be also due to the heterogeneity of countries; in particular, this possibility seems to be relevant for large countries, such as China and India. In this regard, particularly relevant are some considerations by Williams (1929) and Ohlin (1933).
