# Supplementary material for: Trade Specialisation and Changing Patterns of Comparative Advantages in Manufactured Goods
Source: Ital Econ J. 2022 Mar 16;8(3):607–67. doi: 10.1007/s40797-022-00185-4 (PMC8924731; doi:10.1007/s40797-022-00185-4)
Supplement: Supplementary file 1 — Supplementary file1 (DOCX 636 KB) [file 40797_2022_185_MOESM1_ESM.docx]

**Online Supplementary Material**

Table S 1 RSACA. 2001

| **Product label** | **Code** | **Bang** | **Belg** | **Brazil** | **Bulg** | **Camb** | **Chile** | **Chin** | **Col** | **Czech** | **Eth** | **Fin** | **Fran** | **Germ** | **Gre** | **Hon** | **Hung** | **India** | **Indo** | **Irel** | **Italy** | **Jap** |
| --- | --- | --- | --- | --- | --- | --- | --- | --- | --- | --- | --- | --- | --- | --- | --- | --- | --- | --- | --- | --- | --- | --- |
| Dairy products | '04 | -1.35 | -0.06 | -0.52 | 0.46 | -0.08 | 0.23 | -0.06 | 0.16 | 0.50 | -0.49 | 0.33 | 0.27 | 0.04 | -0.12 | -0.47 | 0.47 | 0.48 | -0.66 | 0.44 | -0.50 | -0.69 |
| Products of animal origin | '05 | -0.19 | -0.09 | 0.38 | 0.01 | -0.02 | -0.06 | 0.56 | -0.27 | -0.50 | 1.62 | -0.71 | -0.31 | -0.25 | -0.20 | -0.10 | 0.71 | 0.71 | -0.54 | 0.10 | -0.45 | -1.33 |
| Lac; gums, resins | '13 | -0.73 | -0.21 | -0.09 | -0.49 | -0.66 | 0.67 | 0.33 | -1.30 | 0.16 | 0.92 | -0.87 | 0.25 | -0.01 | 0.66 | -0.08 | -0.58 | 0.73 | -0.07 | 0.51 | -0.01 | -0.99 |
| Vegetable plaiting materials | '14 | 1.08 | -0.12 | 0.83 | 0.01 | 1.08 | 1.25 | -0.15 | -0.11 | -0.78 | 2.00 | -0.78 | -0.24 | -0.36 | 0.25 | 0.02 | 0.98 | 1.29 | 1.32 | -0.02 | -0.94 | -1.26 |
| Animal or vegetable fats and oils | '15 | -1.87 | 0.06 | 0.70 | -0.22 | -1.02 | -0.96 | -0.73 | -0.15 | -0.23 | -0.63 | -0.32 | -0.31 | -0.02 | 0.88 | -0.48 | 0.01 | -0.59 | 1.28 | -0.60 | -0.21 | -0.53 |
| Preparations of meat, fish, molluscs | '16 | 0.02 | 0.04 | 1.22 | 0.16 | -0.19 | 1.03 | 1.44 | -0.35 | -0.22 | -0.13 | -0.71 | -0.19 | -0.24 | -0.08 | -0.60 | 0.84 | 0.08 | 0.76 | 0.13 | -0.34 | -1.23 |
| Sugars and sugar confectionery | '17 | -1.53 | -0.01 | 1.54 | -0.64 | -1.72 | -0.61 | -0.34 | 0.85 | 0.26 | 0.69 | -0.31 | 0.60 | 0.10 | 0.31 | -0.15 | 0.13 | 1.29 | -0.93 | -0.41 | -0.31 | -0.64 |
| Cocoa and cocoa preparations | '18 | -0.13 | 0.28 | 0.54 | -0.35 | -0.01 | -0.11 | -0.19 | 0.43 | -0.29 | -0.10 | -0.32 | -0.14 | -0.13 | -0.33 | -0.15 | -0.19 | -0.10 | 0.69 | -0.14 | -0.06 | -0.75 |
| Preparations of cereals, flour | '19 | -0.68 | 0.23 | 0.15 | 0.16 | -1.22 | 0.07 | 0.45 | -0.07 | -0.29 | -1.15 | -0.77 | -0.02 | 0.02 | 0.15 | -0.34 | -0.13 | 0.37 | 0.27 | 0.10 | 0.62 | -0.66 |
| Preparations of vegetables, fruit, nuts | '20 | -0.19 | 0.13 | 0.91 | 0.39 | -0.23 | 0.90 | 1.06 | -0.11 | -0.36 | -0.46 | -0.86 | -0.28 | -0.46 | 0.74 | -0.29 | 0.81 | 0.58 | 0.50 | -0.72 | 0.40 | -1.27 |
| Miscellaneous edible preparations | '21 | -0.52 | 0.16 | 0.81 | -0.57 | -0.55 | 0.56 | 0.28 | 0.22 | -0.31 | -1.06 | -0.77 | 0.23 | -0.01 | -0.07 | -0.23 | -0.06 | 0.52 | -0.50 | 0.42 | 0.22 | -0.51 |
| Beverages, spirits and vinegar | '22 | -0.16 | -0.16 | -0.06 | 0.86 | -0.63 | 1.17 | 0.34 | -0.45 | 0.22 | -0.26 | -0.53 | 0.70 | -0.38 | 0.09 | -0.37 | 0.43 | 0.14 | -0.02 | -0.07 | 0.60 | -0.91 |
| Residues and waste from food industries | '23 | -1.43 | 0.03 | 1.26 | 0.02 | -0.18 | 0.22 | -0.35 | -1.00 | -0.53 | 0.26 | -0.77 | -0.09 | -0.14 | -0.39 | -0.11 | -0.20 | 1.17 | -1.09 | -0.71 | -0.78 | -0.98 |
| Tobacco and manufactured tobacco substitutes | '24 | -0.16 | -0.06 | 1.46 | 0.42 | -1.16 | 0.21 | 0.11 | -0.07 | 0.03 | -0.83 | -0.74 | -0.69 | 0.09 | 0.45 | 0.07 | -0.24 | 1.06 | -0.17 | -0.07 | -0.88 | -1.08 |
| Salt; sulphur; earths and stone; lime and cement | '25 | -1.79 | 0.12 | 0.35 | 0.36 | -1.74 | 0.12 | 0.31 | 0.58 | 0.12 | -0.51 | -0.69 | 0.03 | -0.06 | 0.98 | -0.36 | -0.27 | 0.23 | -0.13 | -0.46 | -0.42 | -0.70 |
| Ores, slag and ash | '26 | -0.06 | -0.46 | 0.84 | -0.57 | -0.02 | 1.44 | -1.39 | -0.23 | -0.95 | 1.50 | -1.39 | -0.54 | -0.92 | 0.64 | 0.01 | -0.49 | 0.29 | 1.05 | -0.16 | -0.77 | -1.56 |
| Mineral fuels, mineral oils and products of their distillation | '27 | -0.63 | -0.32 | -0.58 | 0.24 | -1.13 | -0.95 | -0.35 | 1.34 | -0.48 | -1.29 | -0.60 | -0.56 | -0.69 | -0.12 | -0.28 | -0.55 | -0.81 | 0.15 | -0.50 | -0.66 | -1.28 |
| Inorganic chemicals | '28 | -0.48 | -0.09 | -0.02 | 0.37 | -0.36 | 0.28 | 0.27 | -0.56 | -0.26 | -0.86 | -0.20 | 0.08 | 0.03 | 0.21 | -0.12 | 0.23 | -0.55 | -0.72 | 0.20 | -0.10 | -0.30 |
| Organic chemicals | '29 | -0.81 | 0.01 | -0.54 | 0.25 | -0.59 | 0.25 | -0.32 | -0.95 | 0.27 | -0.35 | -0.42 | -0.01 | -0.14 | -0.52 | -0.04 | 0.09 | 0.13 | -0.61 | 0.70 | -0.29 | 0.10 |
| Pharmaceutical products | '30 | -0.66 | 0.04 | -0.79 | -0.32 | -1.23 | -0.70 | -0.10 | -0.19 | -0.60 | -1.06 | -0.60 | 0.23 | 0.20 | -0.11 | -0.06 | -0.14 | 0.86 | -0.12 | 0.41 | -0.02 | -0.41 |
| Fertilisers | '31 | -0.21 | 0.33 | -1.16 | 0.81 | -1.04 | 0.01 | -0.65 | -1.22 | -0.14 | -1.79 | 0.12 | -0.70 | 0.11 | 0.11 | -0.01 | -0.55 | -1.18 | -0.44 | -0.83 | -0.63 | -0.54 |
| Tanning or dyeing extracts; paints and varnishes | '32 | -1.26 | 0.15 | -0.42 | -0.87 | -0.51 | -1.22 | -0.24 | 0.32 | -0.37 | -1.04 | 0.12 | -0.04 | 0.33 | -0.17 | -0.10 | -0.63 | 0.51 | -0.94 | -0.69 | -0.17 | 0.30 |
| Essential oils; perfumery, cosmetics | '33 | -0.62 | -0.04 | -0.10 | 0.07 | -0.42 | -0.38 | 0.19 | -0.31 | -0.51 | -0.96 | -0.58 | 0.49 | 0.03 | -0.05 | -0.35 | -0.51 | 0.44 | -0.45 | 0.34 | -0.03 | -0.45 |
| Soap, washing preparations, waxes, candles | '34 | -0.72 | 0.14 | -0.27 | -0.86 | -1.14 | -1.02 | -0.10 | -0.02 | 0.17 | -1.23 | -0.53 | -0.02 | 0.24 | -0.09 | -0.01 | -0.08 | -0.24 | -0.37 | -0.78 | 0.14 | 0.10 |
| Albuminoidal substances | '35 | -0.54 | 0.08 | 0.05 | -0.32 | -0.92 | -0.77 | -0.50 | -0.13 | -0.50 | -0.98 | -0.10 | 0.20 | -0.02 | -0.47 | -0.13 | -0.30 | 0.36 | -1.23 | 0.32 | -0.23 | -0.40 |
| Explosives; pyrotechnic products; matches | '36 | -0.62 | 0.00 | 0.60 | 0.23 | -0.68 | 0.60 | 1.56 | -0.95 | 0.75 | -1.89 | -0.34 | 0.26 | -0.17 | -1.19 | -0.01 | 0.34 | 0.72 | -0.68 | 0.15 | -0.60 | -0.81 |
| Photographic or cinematographic goods | '37 | -0.86 | 0.47 | -0.24 | 0.46 | -0.36 | -1.07 | -0.09 | -1.26 | -0.50 | -0.87 | -0.65 | 0.00 | 0.03 | 0.32 | -0.28 | -0.60 | -0.74 | -0.75 | -0.58 | -0.33 | 0.81 |
| Miscellaneous chemical products | '38 | -0.92 | 0.16 | -0.41 | -0.93 | -0.39 | -0.92 | -0.36 | -0.04 | -0.46 | -1.27 | -0.30 | 0.11 | 0.24 | -0.13 | -0.01 | -0.27 | 0.10 | -0.49 | 0.62 | -0.15 | 0.07 |
| Plastics and articles thereof | '39 | -0.93 | 0.20 | -0.36 | -0.32 | -0.74 | -0.67 | -0.45 | -0.10 | -0.24 | -0.95 | -0.19 | -0.04 | 0.16 | 0.06 | 0.02 | -0.12 | 0.15 | -0.34 | -0.50 | -0.02 | 0.16 |
| Rubber and articles thereof | '40 | -0.79 | -0.16 | -0.03 | -0.10 | 0.46 | -0.50 | -0.14 | -0.51 | 0.13 | -1.53 | -0.26 | 0.19 | -0.05 | -0.24 | -0.12 | -0.03 | 0.25 | 0.34 | -0.40 | -0.03 | 0.47 |
| Raw hides and skins (other than furskins) and leather | '41 | 1.64 | 0.00 | 0.68 | -0.39 | -1.15 | 0.27 | -0.63 | 1.02 | -0.43 | 1.97 | -0.14 | 0.07 | -0.01 | 0.67 | -0.08 | -0.63 | 0.43 | -0.62 | 0.53 | -0.02 | -0.31 |
| Articles of leather; saddlery; travel goods, handbags | '42 | 0.18 | 0.01 | 0.14 | 0.70 | 0.14 | -0.95 | 1.58 | 0.54 | -0.20 | -0.43 | -0.53 | 0.19 | -0.49 | -0.55 | 0.10 | 0.28 | 1.62 | 1.04 | -0.55 | 0.42 | -1.37 |
| Furskins and artificial fur; manufactures thereof | '43 | -0.12 | -0.15 | 0.32 | 0.17 | -0.05 | 0.01 | 0.29 | 0.03 | 0.20 | 1.16 | 0.21 | -0.17 | -0.18 | 0.10 | -0.07 | 0.03 | -0.06 | -0.13 | 0.06 | -0.18 | -1.15 |
| Wood and articles of wood | '44 | -0.46 | 0.03 | 1.30 | 0.73 | 1.09 | 1.21 | -0.22 | 0.11 | 0.48 | -0.61 | 0.35 | -0.07 | -0.10 | -0.52 | -0.01 | 0.21 | -0.85 | 1.22 | -0.52 | -0.47 | -1.43 |
| Cork and articles of cork | '45 | -0.25 | -0.32 | -0.54 | -0.24 | -0.55 | -1.45 | -0.22 | -0.02 | -0.69 | -0.56 | -0.28 | -0.62 | -0.68 | -0.86 | 0.05 | -0.95 | -0.11 | 0.09 | -0.12 | -0.32 | -0.51 |
| Manufactures of straw, basketware | '46 | 1.40 | -0.08 | -0.30 | -0.26 | -0.49 | -0.71 | 1.58 | -0.08 | -0.47 | 0.52 | -0.64 | -0.75 | -0.65 | -0.86 | 0.19 | 0.08 | 0.32 | 1.36 | -0.48 | -0.81 | -1.59 |
| Pulp of wood or of other fibrous cellulosic material | '47 | -1.05 | -0.11 | 0.81 | 0.47 | -0.01 | 1.40 | -1.50 | -1.22 | 0.35 | -0.87 | 0.72 | -0.56 | -0.74 | -0.40 | 0.16 | -0.62 | -1.18 | -0.22 | -0.17 | -1.25 | -0.88 |
| Paper and paperboard; articles of paper pulp, of paper or of paperboard | '48 | -1.18 | -0.04 | 0.27 | -0.59 | -1.23 | -0.12 | -0.45 | -0.04 | -0.11 | -1.09 | 0.92 | -0.08 | 0.03 | -0.52 | -0.08 | -0.22 | -0.28 | 0.61 | -0.72 | -0.01 | -0.08 |
| Printed books, newspapers, pictures and products of the printing industry | '49 | -0.38 | 0.02 | -0.69 | -0.44 | 1.60 | 0.08 | 0.06 | 0.43 | 0.06 | -1.13 | 0.20 | 0.00 | 0.23 | 0.07 | 0.22 | -0.36 | -0.54 | -0.11 | -0.44 | 0.36 | -0.40 |
| Silk | '50 | 0.19 | -0.20 | 0.99 | -0.91 | 0.12 | -0.07 | 0.62 | -0.37 | -0.31 | 0.00 | -0.19 | -0.13 | -0.21 | -0.73 | -0.11 | -0.63 | 0.10 | -0.35 | -0.13 | 0.00 | -0.50 |
| Wool, fine or coarse animal hair; horsehair yarn and woven fabric | '51 | -0.64 | -0.12 | 0.12 | -0.44 | -0.11 | 0.21 | -0.26 | -0.72 | -0.04 | -0.01 | -0.29 | 0.03 | -0.10 | -0.03 | -0.15 | -0.69 | -0.41 | -0.53 | -0.35 | 0.14 | -0.33 |
| Cotton | '52 | -1.01 | 0.05 | 0.56 | -0.65 | -1.01 | -0.32 | 0.02 | -0.82 | -0.10 | 1.52 | -0.26 | 0.02 | -0.10 | 0.78 | 0.04 | -0.38 | 0.60 | -0.40 | -0.32 | 0.06 | -0.16 |
| Other vegetable textile fibres; paper yarn and woven fabrics of paper yarn | '53 | 1.26 | 0.02 | 0.32 | -1.31 | -0.13 | -0.14 | 0.13 | -0.33 | 0.27 | -1.42 | -0.24 | 0.35 | -0.20 | -0.52 | -0.01 | -0.09 | 0.57 | -0.99 | 0.00 | 0.08 | -0.43 |
| Man-made filaments; strip and the like of man-made textile materials | '54 | -1.06 | -0.01 | -0.96 | -0.58 | -0.97 | -0.54 | -0.39 | -0.79 | -0.26 | -1.61 | -0.46 | -0.10 | 0.00 | -0.33 | -0.11 | -0.24 | 0.44 | 0.20 | 0.10 | 0.02 | 0.75 |
| Man-made staple fibres | '55 | -1.62 | 0.03 | -0.34 | -0.49 | -1.09 | -0.67 | -0.05 | -0.49 | -0.10 | -0.93 | -0.02 | 0.10 | 0.15 | -0.46 | 0.00 | -0.62 | 0.88 | 0.13 | 0.19 | 0.17 | 0.52 |
| Wadding, felt and nonwovens; special yarns; twine, cordage, ropes | '56 | 0.82 | -0.19 | -0.03 | -0.85 | -1.70 | -0.84 | -0.18 | -0.16 | -0.06 | -0.76 | 0.01 | -0.04 | 0.32 | 0.02 | -0.06 | -0.14 | 0.21 | -0.45 | -0.59 | 0.33 | 0.10 |
| Carpets and other textile floor coverings | '57 | 0.06 | 0.82 | 0.04 | 0.32 | -0.20 | -0.98 | 0.96 | -0.13 | -0.30 | -0.78 | -0.70 | -0.18 | -0.63 | 0.28 | -0.18 | -0.47 | 1.55 | 0.36 | -0.53 | -0.28 | -0.90 |
| Special woven fabrics; tufted textile fabrics; lace; tapestries | '58 | -0.14 | 0.58 | -0.08 | -1.05 | -1.81 | -0.71 | 0.00 | -0.09 | -0.01 | -0.85 | -0.23 | 0.22 | 0.17 | -0.12 | 0.06 | -0.87 | 0.98 | -0.30 | -0.42 | 0.07 | 0.11 |
| Impregnated, coated, covered or laminated textile fabrics | '59 | -1.50 | 0.36 | -0.54 | -1.43 | -0.88 | -0.93 | -0.57 | 0.21 | -0.27 | -1.19 | -0.30 | -0.11 | 0.13 | -0.44 | -0.14 | -0.76 | -0.55 | -0.57 | -0.33 | 0.27 | 0.22 |
| Knitted or crocheted fabrics | '60 | -0.50 | -0.07 | -0.15 | -1.11 | -1.20 | -0.26 | -0.10 | 0.32 | -0.45 | 0.10 | -0.22 | 0.06 | 0.19 | 0.24 | -0.03 | -1.00 | -0.07 | -0.77 | -0.47 | 0.35 | 0.30 |
| Articles of apparel and clothing accessories, knitted or crocheted | '61 | 1.79 | -0.10 | 0.25 | 0.35 | 1.75 | -0.79 | 1.34 | 0.94 | 0.05 | -0.28 | -0.62 | -0.36 | -0.58 | 0.67 | 0.15 | 0.36 | 1.50 | 1.30 | -0.69 | 0.34 | -1.19 |
| Articles of apparel and clothing accessories, not knitted or crocheted | '62 | 0.78 | -0.10 | 0.04 | 1.01 | 0.10 | -0.76 | 1.32 | 1.02 | 0.27 | -0.62 | -0.60 | -0.33 | -0.49 | 0.08 | 0.20 | 0.62 | 1.59 | 1.45 | -0.59 | 0.35 | -1.22 |
| Other made-up textile articles; sets; worn clothing | '63 | 1.16 | 0.03 | 0.98 | 0.72 | -0.31 | -0.88 | 1.54 | 0.45 | 0.56 | -1.36 | -0.61 | -0.41 | -0.42 | 0.37 | 0.07 | 0.24 | 1.19 | 0.94 | -0.58 | 0.03 | -1.10 |
| Footwear, gaiters and the like | '64 | 0.59 | 0.11 | 1.40 | 0.64 | 0.74 | -0.97 | 1.38 | -0.27 | 0.02 | -0.81 | -0.42 | -0.49 | -0.56 | -0.45 | 0.09 | 0.40 | 1.25 | 1.14 | -0.64 | 0.41 | -1.02 |
| Headgear and parts thereof | '65 | 1.82 | 0.18 | -0.07 | 0.36 | 1.22 | -0.89 | 1.56 | 0.06 | 0.48 | -0.75 | -0.77 | -0.36 | -0.30 | -0.53 | 0.23 | 0.00 | 0.40 | 0.96 | -0.61 | 0.52 | -0.49 |
| Umbrellas, sun umbrellas, walking sticks | '66 | -0.40 | -0.10 | -0.72 | -0.39 | -0.27 | -0.77 | 1.41 | -0.48 | 0.14 | -1.56 | -0.51 | -0.46 | -0.63 | -0.07 | 0.07 | -0.31 | -0.61 | 0.79 | -0.50 | -0.06 | -1.37 |
| Prepared feathers and down; artificial flowers; articles of human hair | '67 | 0.18 | -0.01 | -0.32 | -0.16 | -0.35 | -0.58 | 0.97 | -0.49 | -0.15 | -0.29 | -0.43 | -0.56 | -0.47 | -0.72 | 0.12 | -0.17 | 1.33 | 0.94 | -0.18 | -0.38 | -0.94 |
| Articles of stone, plaster, cement, mica or similar materials | '68 | -0.85 | 0.25 | 0.52 | -0.21 | -0.95 | -0.60 | 0.66 | 0.15 | 0.17 | -0.84 | 0.19 | -0.07 | -0.07 | 0.77 | -0.40 | -0.30 | 1.02 | 0.19 | -0.76 | 0.65 | -0.23 |
| Ceramic products | '69 | -0.04 | -0.19 | 0.65 | 0.25 | -1.35 | -0.69 | 0.99 | 0.32 | 0.30 | -0.85 | -0.51 | -0.22 | -0.11 | -0.23 | -0.16 | -0.04 | 0.29 | 0.22 | -0.63 | 0.79 | 0.13 |
| Glass and glassware | '70 | -0.87 | 0.17 | -0.04 | 0.03 | -0.70 | -0.46 | -0.11 | 0.11 | 0.47 | -1.08 | 0.12 | 0.11 | 0.03 | -0.59 | -0.21 | -0.01 | 0.11 | 0.48 | -0.14 | 0.08 | 0.20 |
| Natural or cultured pearls, precious stones, imitation jewellery, coin | '71 | 0.00 | 0.02 | 0.35 | 0.00 | 0.03 | 1.01 | 0.30 | 0.68 | 0.13 | 1.17 | -0.02 | -0.06 | -0.15 | -0.01 | -0.07 | -0.02 | 0.00 | 0.65 | -0.29 | -0.03 | -0.58 |
| Iron and steel | '72 | -1.33 | 0.26 | 0.90 | 0.50 | -0.80 | -0.70 | -0.77 | 0.02 | 0.02 | -1.39 | 0.14 | 0.09 | 0.01 | -0.24 | -0.14 | -0.16 | 0.08 | -0.86 | -0.46 | -0.30 | 0.64 |
| Articles of iron or steel | '73 | -0.92 | -0.03 | 0.04 | 0.03 | -0.80 | -0.50 | 0.47 | -0.09 | 0.25 | -1.34 | -0.05 | 0.04 | 0.08 | 0.22 | -0.04 | -0.19 | 0.66 | -0.45 | -0.63 | 0.50 | 0.32 |
| Copper and articles thereof | '74 | -0.50 | 0.11 | -0.47 | 0.71 | -0.04 | 1.69 | -0.98 | -0.52 | -0.48 | -0.71 | 0.31 | -0.11 | 0.03 | 0.32 | -0.02 | -0.33 | -0.06 | 0.27 | -0.49 | -0.42 | 0.38 |
| Nickel and articles thereof | '75 | -0.07 | -0.34 | 0.18 | -0.25 | 0.00 | -0.26 | -0.77 | 0.19 | -0.53 | -0.03 | 0.03 | -0.33 | -0.30 | -0.25 | -0.07 | -0.29 | -1.12 | 0.96 | -0.39 | -0.95 | -0.61 |
| Aluminium and articles thereof | '76 | -0.70 | -0.09 | 0.49 | -0.12 | -0.81 | -0.98 | -0.25 | -0.14 | -0.18 | -0.68 | -0.37 | -0.14 | -0.10 | 0.66 | -0.06 | 0.06 | 0.15 | -0.17 | -0.36 | -0.11 | -0.71 |
| Lead and articles thereof | '78 | -1.33 | 0.23 | -1.38 | 0.71 | 0.04 | -0.44 | 1.05 | -0.30 | -0.75 | 0.33 | -0.32 | -0.11 | 0.01 | 0.47 | -0.05 | -0.66 | -1.24 | -1.09 | -0.59 | -0.67 | -0.44 |
| Zinc and articles thereof | '79 | -1.81 | -0.12 | -0.19 | 1.26 | -0.82 | -0.97 | 0.41 | -1.32 | -0.55 | -0.93 | 1.31 | 0.03 | -0.32 | -0.40 | 0.06 | -0.67 | -1.24 | -1.35 | -0.24 | -0.82 | -0.06 |
| Tin and articles thereof | '80 | -0.83 | 0.27 | 1.11 | -0.63 | -0.33 | -0.95 | 0.40 | -0.76 | -0.48 | -0.34 | -0.41 | -0.36 | -0.66 | -0.77 | -0.05 | -0.72 | -0.27 | 1.36 | -0.30 | -0.56 | -0.56 |
| Other base metals; cermets; articles thereof | '81 | -0.19 | -0.04 | -0.32 | -0.17 | -0.02 | 0.75 | 0.59 | -0.27 | -0.41 | -0.03 | 0.40 | -0.03 | -0.25 | -0.26 | -0.02 | -0.22 | -0.73 | -0.36 | -0.43 | -0.27 | -0.20 |
| Tools, implements, cutlery, spoons and forks, of base metal | '82 | -0.36 | -0.13 | -0.11 | -0.04 | -0.63 | -1.06 | 0.70 | -0.29 | -0.02 | -1.09 | -0.58 | -0.21 | 0.11 | -0.46 | 0.08 | -0.45 | 0.50 | -0.45 | -0.43 | -0.10 | 0.41 |
| Miscellaneous articles of base metal | '83 | -0.51 | -0.44 | -0.05 | -0.27 | -0.95 | -0.63 | 0.63 | -0.30 | 0.05 | -1.11 | -0.36 | 0.04 | 0.24 | -0.06 | 0.05 | -0.22 | 0.56 | -0.46 | -0.59 | 0.61 | -0.01 |
| Machinery, mechanical appliances, nuclear reactors, boilers | '84 | -0.76 | -0.09 | -0.37 | -0.30 | -0.43 | -0.90 | -0.15 | -0.80 | -0.09 | -0.73 | -0.11 | -0.08 | 0.09 | -0.30 | -0.08 | 0.09 | -0.30 | -0.53 | -0.02 | 0.25 | 0.27 |
| Electrical machinery and equipment; sound recorders and reproducers, tv | '85 | -0.50 | -0.05 | -0.48 | -0.33 | -0.36 | -0.72 | -0.08 | -0.59 | -0.02 | -0.63 | 0.13 | 0.07 | -0.04 | -0.12 | -0.01 | 0.03 | -0.19 | 0.37 | -0.05 | -0.10 | 0.25 |
| Railway or tramway locomotives; railway or tramway track | '86 | -0.30 | -0.25 | -0.40 | -0.07 | -0.17 | -1.01 | 0.85 | -1.26 | 0.38 | -0.45 | -0.79 | 0.27 | 0.13 | -1.79 | -0.73 | 0.22 | 0.21 | -0.49 | -0.44 | 0.11 | 0.41 |
| Vehicles other than railway or tramway rolling stock | '87 | -0.51 | 0.08 | 0.11 | -0.92 | -0.58 | -0.71 | -0.01 | -0.16 | 0.33 | -1.04 | -0.26 | 0.11 | 0.32 | -0.75 | -0.12 | 0.10 | 0.28 | -0.63 | -0.63 | -0.20 | 0.88 |
| Aircraft, spacecraft, and parts thereof | '88 | -0.23 | -0.01 | 0.62 | -0.51 | -0.02 | 0.14 | -0.92 | -1.34 | -0.14 | -0.83 | -0.47 | 0.38 | -0.08 | -0.43 | -0.53 | -0.01 | -0.32 | -0.23 | -0.81 | -0.23 | -0.18 |
| Ships, boats and floating structures | '89 | -1.72 | -0.14 | -0.08 | -0.27 | 0.14 | -0.41 | 0.18 | -0.30 | 0.07 | -0.01 | 0.51 | 0.15 | -0.06 | -0.99 | -0.11 | -0.04 | -0.95 | -1.55 | -0.26 | 0.13 | 1.20 |
| Optical, photographic, cinematographic, medical or surgical instruments | '90 | -0.32 | -0.18 | -0.66 | -0.50 | -0.46 | -0.73 | -0.26 | -0.80 | -0.29 | -0.74 | -0.06 | -0.08 | 0.09 | -0.50 | 0.08 | -0.11 | -0.45 | -0.21 | 0.08 | -0.19 | 0.22 |
| Clocks and watches and parts thereof | '91 | -0.22 | -0.19 | -0.58 | 0.01 | -0.02 | -0.62 | 0.29 | -0.58 | -0.24 | -0.14 | -0.38 | -0.17 | -0.25 | -0.69 | 0.07 | -0.20 | 0.29 | -0.04 | -0.30 | -0.34 | -0.35 |
| Musical instruments; parts and accessories of such articles | '92 | -0.03 | -0.14 | -0.64 | -0.01 | 0.72 | -0.82 | 0.70 | -0.73 | 0.69 | -0.48 | -0.72 | -0.13 | 0.02 | -0.65 | -0.06 | -0.24 | 0.26 | 0.75 | -0.50 | 0.22 | 0.16 |
| Arms and ammunition; parts and accessories thereof | '93 | -1.57 | 0.42 | 0.43 | 0.00 | 0.01 | -0.34 | 0.06 | -1.66 | 0.72 | -1.62 | -0.27 | 0.40 | -0.01 | -0.48 | -0.04 | 0.05 | 0.24 | -0.96 | -0.21 | 0.49 | -0.60 |
| Furniture; bedding, mattresses, stuffed furnishings; lamps | '94 | -0.30 | -0.05 | 0.47 | 0.37 | -0.26 | -0.17 | 1.19 | 0.25 | 0.48 | -0.90 | -0.11 | -0.20 | -0.21 | -0.39 | 0.03 | 0.45 | 0.05 | 1.23 | -0.57 | 0.88 | -0.83 |
| Toys, games and sports requisites | '95 | -0.06 | -0.07 | -0.14 | 0.24 | -0.23 | -0.80 | 1.43 | -0.42 | 0.23 | -0.18 | -0.25 | -0.25 | -0.19 | -0.21 | 0.20 | 0.78 | 0.22 | 0.42 | -0.30 | 0.05 | -0.13 |
| Miscellaneous manufactured articles | '96 | -1.36 | 0.03 | 0.11 | -0.66 | -1.67 | -0.70 | 0.65 | -0.12 | 0.27 | -1.16 | -0.51 | 0.01 | 0.20 | -0.18 | 0.16 | -0.42 | 0.46 | 0.02 | -0.05 | 0.17 | 0.36 |
| Works of art, collectors' pieces and antiques | '97 | 0.02 | 0.06 | 0.01 | 0.00 | 0.07 | -0.08 | 0.06 | 0.07 | 0.14 | 0.33 | -0.04 | 0.61 | 0.01 | 0.25 | -0.08 | 0.03 | 0.08 | 0.06 | -0.23 | 0.15 | -0.28 |
| Commodities not elsewhere specified | '99 | 0.05 | 0.12 | 0.86 | -0.31 | -0.02 | 0.78 | -0.27 | -0.52 | 0.38 | 0.00 | -0.58 | 0.00 | -0.11 | 0.55 | 0.02 | 0.20 | 0.29 | 0.00 | -0.21 | -0.56 | 0.35 |
| Parts and accessories for calculating machines | 8473 | -0.50 | -0.13 | 0.08 | -1.62 | -0.98 | 0.01 | 1.08 | -0.35 | -0.09 | -0.16 | -0.50 | -0.34 | -0.39 | -0.31 | -0.01 | -0.14 | -0.33 | 0.72 | -0.03 | -0.20 | 0.01 |
| Electronic integrated circuits; parts thereof | 8542 | -0.01 | -0.11 | 0.06 | -1.66 | -0.62 | 0.01 | 0.45 | -1.95 | 0.28 | -1.86 | 0.27 | 0.55 | 0.19 | -1.75 | 1.23 | -1.51 | -0.83 | -0.25 | 1.32 | -0.26 | 0.89 |
| Parts and accessories for tractors, motor vehicles | 8708 | -0.09 | -0.38 | -0.09 | -0.28 | -0.06 | -0.35 | -0.27 | -0.25 | 0.14 | -0.71 | -0.64 | 0.21 | 0.13 | -0.55 | -0.10 | 0.14 | 0.21 | -0.82 | -0.24 | 0.28 | 0.81 |
| Parts of aircraft and spacecraft | 8803 | -0.60 | 0.18 | -0.68 | -0.03 | -0.04 | 0.41 | -0.47 | -1.11 | -0.05 | -1.38 | -0.47 | -0.23 | -0.41 | -0.24 | -0.07 | -0.04 | -0.19 | -0.53 | -0.28 | 0.04 | 0.05 |
| Parts of telephone sets, telephones for cellular networks | 851770 | -0.39 | -0.01 | -1.15 | -0.83 | -0.62 | -0.75 | 0.04 | -0.90 | -0.36 | 0.06 | -0.36 | -0.01 | -0.03 | 0.11 | 0.03 | -0.21 | -0.47 | -0.11 | 0.14 | 0.16 | -0.05 |

| **Product label** | **Code** | **Kor** | **Mal** | **Mex** | **Neth** | **Nor** | **Paki** | **Phil** | **Pol** | **Port** | **Rom** | **Sing** | **Spa** | **Swe** | **Switz** | **Taiwan** | **Thai** | **Tun** | **Turk** | **UK** | **US** | **Viet** |
| --- | --- | --- | --- | --- | --- | --- | --- | --- | --- | --- | --- | --- | --- | --- | --- | --- | --- | --- | --- | --- | --- | --- |
| Dairy products | '04 | -0.38 | -0.66 | -0.82 | 0.27 | 0.15 | -0.03 | -1.13 | 0.94 | -0.20 | -0.11 | -0.41 | -0.12 | -0.20 | 0.11 | -0.57 | -0.50 | -0.24 | 0.24 | -0.29 | 0.04 | 0.04 |
| Products of animal origin | '05 | -0.57 | -0.26 | -0.81 | 0.21 | -0.23 | 1.34 | 0.02 | -0.03 | 0.00 | 0.00 | 0.02 | 0.20 | -0.54 | -0.53 | 0.11 | -0.16 | 0.59 | 0.48 | -0.11 | 0.34 | 0.42 |
| Lac; gums, resins | '13 | -0.21 | -0.63 | -0.23 | -0.03 | -0.24 | 0.88 | 0.56 | -0.90 | -0.17 | -0.63 | 0.12 | 0.59 | -0.40 | 0.25 | 0.02 | -0.27 | -0.69 | -0.73 | -0.26 | 0.02 | 0.34 |
| Vegetable plaiting materials | '14 | -0.80 | -0.33 | 0.09 | -0.61 | -0.38 | -0.38 | -0.40 | 0.51 | -0.04 | 0.68 | 0.36 | -0.26 | -1.28 | -0.56 | -0.08 | -0.10 | 0.04 | 1.01 | -0.52 | -0.21 | 0.62 |
| Animal or vegetable fats and oils | '15 | -0.66 | 0.92 | -0.68 | 0.14 | -0.74 | -0.80 | 1.04 | -0.66 | 0.12 | 0.01 | 0.07 | 0.74 | -0.10 | -0.47 | -0.40 | 0.29 | 0.28 | 0.03 | -0.39 | 0.29 | -0.14 |
| Preparations of meat, fish, molluscs | '16 | 0.18 | 0.28 | -0.14 | 0.17 | 0.33 | 0.57 | 0.72 | 0.81 | 0.21 | 0.05 | -0.51 | 0.44 | -0.54 | -0.86 | 0.17 | 1.64 | -0.04 | 0.40 | -0.87 | -0.22 | 1.34 |
| Sugars and sugar confectionery | '17 | -0.49 | -0.62 | 0.20 | 0.26 | -0.93 | 1.11 | -0.17 | 0.61 | -0.43 | -1.35 | -0.36 | 0.20 | -0.35 | -0.19 | -0.45 | 1.44 | -0.99 | 1.43 | -0.24 | -0.09 | 0.22 |
| Cocoa and cocoa preparations | '18 | -0.35 | 0.08 | -0.39 | 0.14 | -0.71 | -0.22 | -0.60 | 0.15 | -1.04 | -0.68 | 0.19 | -0.13 | -0.17 | 0.34 | -0.31 | -0.18 | -0.11 | 0.27 | -0.17 | -0.14 | -0.12 |
| Preparations of cereals, flour | '19 | 0.21 | -0.15 | -0.09 | 0.33 | -1.06 | -0.46 | -0.29 | 0.42 | -0.44 | -0.33 | -0.10 | 0.12 | -0.22 | 0.05 | -0.61 | 0.22 | 0.88 | 0.79 | 0.04 | 0.08 | 0.96 |
| Preparations of vegetables, fruit, nuts | '20 | -0.46 | -0.18 | 0.02 | 0.23 | -0.92 | 0.04 | 0.68 | 0.50 | 0.15 | -0.44 | -0.26 | 0.59 | -0.93 | -0.42 | -0.24 | 1.15 | 0.75 | 1.51 | -0.65 | 0.10 | 1.02 |
| Miscellaneous edible preparations | '21 | -0.44 | -0.10 | -0.21 | 0.43 | -0.88 | -0.06 | -0.59 | 0.00 | -0.33 | -1.15 | 0.04 | 0.06 | -0.40 | 0.23 | -0.50 | 0.55 | -0.37 | 0.19 | -0.04 | 0.55 | 0.47 |
| Beverages, spirits and vinegar | '22 | -0.25 | 0.16 | 0.77 | 0.27 | -0.89 | 0.40 | -0.22 | -0.10 | 0.42 | 0.16 | -0.14 | 0.32 | -0.15 | -0.94 | -0.45 | 0.11 | 0.52 | 0.23 | 0.06 | -0.52 | 0.13 |
| Residues and waste from food industries | '23 | -0.93 | -0.49 | -0.67 | 0.15 | -0.39 | -0.45 | -0.91 | -0.53 | -0.60 | -0.33 | 0.04 | -0.44 | -0.79 | -0.31 | -0.65 | -0.31 | -0.57 | -0.55 | -0.35 | 1.00 | -1.27 |
| Tobacco and manufactured tobacco substitutes | '24 | -0.33 | -0.02 | 0.08 | 0.42 | -0.62 | 0.34 | -0.65 | -0.02 | 0.13 | -1.06 | 0.02 | -0.79 | -0.43 | 0.29 | -1.17 | -0.18 | -0.16 | 0.30 | 0.39 | 0.77 | -0.35 |
| Salt; sulphur; earths and stone; lime and cement | '25 | -0.47 | -0.39 | 0.14 | -0.13 | -0.12 | 0.10 | -0.56 | -0.16 | -0.36 | 0.19 | -0.62 | 0.12 | -0.39 | -0.55 | -0.65 | 0.70 | 0.03 | 0.88 | 0.31 | 0.20 | -0.57 |
| Ores, slag and ash | '26 | -1.46 | -0.46 | -0.11 | -0.35 | -0.56 | -0.59 | -0.36 | -0.66 | 0.88 | -0.73 | 0.08 | -0.31 | 0.31 | 0.01 | -0.92 | -0.61 | 0.22 | 0.14 | -0.78 | 0.14 | 1.00 |
| Mineral fuels, mineral oils and products of their distillation | '27 | -0.73 | 0.28 | 0.40 | -0.17 | 1.16 | -1.01 | -0.84 | -0.28 | -0.69 | -0.36 | -0.24 | -0.59 | -0.44 | -0.55 | -0.79 | -0.62 | -0.02 | -1.06 | 0.29 | -0.73 | 0.39 |
| Inorganic chemicals | '28 | -0.54 | -0.59 | -0.27 | 0.05 | -0.37 | -0.89 | -0.64 | 0.13 | -0.18 | 0.44 | -0.37 | -0.20 | -0.45 | 0.00 | -0.54 | -0.64 | 0.46 | -0.06 | 0.23 | 0.22 | -0.94 |
| Organic chemicals | '29 | -0.03 | -0.16 | -0.45 | 0.22 | -0.14 | -1.13 | -0.62 | -0.12 | -0.11 | 0.10 | 0.46 | -0.08 | -0.48 | 0.20 | -0.48 | -0.40 | -0.50 | -0.82 | 0.20 | 0.03 | -0.49 |
| Pharmaceutical products | '30 | -0.29 | -0.33 | -0.13 | 0.00 | -0.75 | -0.45 | -0.54 | -0.95 | -0.48 | -1.04 | 0.01 | -0.19 | 0.38 | 0.16 | -0.53 | -0.34 | -0.90 | -0.80 | 0.27 | 0.09 | -0.96 |
| Fertilisers | '31 | -0.07 | -0.36 | -0.50 | 0.61 | -0.94 | -1.45 | -0.75 | 0.32 | -0.09 | 1.17 | -0.01 | -0.25 | -0.29 | -0.26 | -0.37 | -1.26 | 1.56 | -0.99 | -0.24 | 0.36 | -1.38 |
| Tanning or dyeing extracts; paints and varnishes | '32 | -0.28 | -0.30 | -0.23 | 0.25 | -0.85 | -1.20 | -0.75 | -0.66 | -0.59 | -1.06 | 0.18 | 0.20 | -0.11 | 0.29 | -0.10 | -0.88 | -0.45 | -0.65 | 0.29 | 0.43 | -1.06 |
| Essential oils; perfumery, cosmetics | '33 | -0.62 | -0.59 | -0.21 | -0.06 | -1.09 | -0.51 | -0.74 | -0.05 | -0.80 | -0.93 | -0.06 | 0.03 | -0.34 | 0.30 | -0.51 | 0.16 | 0.11 | -0.37 | 0.15 | 0.39 | -0.25 |
| Soap, washing preparations, waxes, candles | '34 | -0.34 | 0.07 | 0.14 | 0.24 | -0.65 | -0.93 | -0.51 | 0.01 | -0.31 | -0.58 | -0.10 | 0.44 | -0.24 | -0.11 | -0.44 | -0.47 | 0.01 | 0.34 | 0.11 | 0.45 | 0.07 |
| Albuminoidal substances | '35 | -0.33 | -0.36 | -0.87 | 0.22 | -0.63 | -0.30 | -0.96 | -0.28 | -0.66 | -1.20 | -0.07 | -0.53 | -0.04 | 0.14 | 0.19 | 0.47 | -0.71 | -0.77 | 0.12 | 0.41 | -0.82 |
| Explosives; pyrotechnic products; matches | '36 | -0.05 | -0.16 | -0.39 | 0.26 | -1.42 | 1.00 | -0.05 | 0.03 | -0.87 | -0.68 | -0.61 | 0.47 | 0.24 | 0.24 | -0.12 | 0.09 | 0.30 | 0.27 | -0.03 | 0.25 | -0.48 |
| Photographic or cinematographic goods | '37 | -0.80 | -0.30 | -0.25 | 0.18 | -0.76 | -0.84 | -0.83 | -0.84 | -0.63 | -0.66 | 0.01 | -0.24 | -0.43 | -0.23 | -0.93 | -0.67 | -0.70 | -0.87 | 0.18 | 0.28 | -0.33 |
| Miscellaneous chemical products | '38 | -0.59 | 0.13 | -0.65 | 0.18 | -0.31 | -1.24 | -0.63 | -0.80 | -0.19 | -0.83 | -0.04 | -0.15 | -0.10 | 0.23 | -0.57 | -0.75 | -0.67 | -0.72 | 0.27 | 0.63 | -1.15 |
| Plastics and articles thereof | '39 | 0.42 | -0.17 | -0.57 | 0.28 | -0.70 | -0.50 | -0.58 | -0.32 | -0.17 | -0.50 | 0.13 | 0.08 | -0.08 | -0.05 | 0.35 | 0.04 | -0.53 | -0.36 | -0.05 | 0.38 | -0.69 |
| Rubber and articles thereof | '40 | 0.44 | 0.40 | -0.59 | -0.08 | -0.82 | -1.08 | -0.27 | 0.19 | -0.07 | -0.24 | -0.06 | 0.20 | -0.26 | -0.42 | 0.26 | 0.72 | -0.18 | 0.29 | 0.03 | 0.08 | 0.41 |
| Raw hides and skins (other than furskins) and leather | '41 | -0.07 | -0.37 | -0.56 | 0.13 | -0.20 | 0.89 | -0.59 | -0.33 | -0.71 | -0.70 | 0.05 | -0.05 | 0.15 | 0.06 | 0.08 | -0.28 | -0.67 | -0.74 | 0.18 | 0.59 | -1.16 |
| Articles of leather; saddlery; travel goods, handbags | '42 | 0.21 | -0.05 | -0.17 | -0.23 | -0.81 | 1.71 | 1.28 | 0.18 | -0.53 | 0.82 | -0.42 | -0.14 | -0.54 | -0.38 | 0.04 | 1.07 | 0.96 | 0.99 | -0.50 | -0.81 | 1.45 |
| Furskins and artificial fur; manufactures thereof | '43 | -0.88 | 0.01 | -0.06 | 0.12 | 0.36 | -0.12 | 0.06 | 0.15 | 0.37 | 0.04 | -0.02 | 0.63 | 0.23 | -0.66 | 0.22 | -0.26 | -0.09 | 0.05 | 0.01 | -0.13 | 0.19 |
| Wood and articles of wood | '44 | -0.89 | 1.00 | -0.24 | -0.53 | -0.65 | -0.25 | -0.08 | 0.63 | 0.10 | 0.86 | -0.05 | -0.27 | 0.36 | -0.20 | -0.44 | 0.23 | -0.71 | -0.02 | -0.72 | -0.27 | 0.14 |
| Cork and articles of cork | '45 | -0.12 | -0.05 | -0.18 | -0.17 | -0.37 | -0.05 | -0.15 | -0.42 | 0.11 | -0.72 | -0.04 | 0.26 | -0.15 | -0.27 | 0.01 | -0.15 | 1.47 | -0.40 | -0.31 | -0.45 | -0.10 |
| Manufactures of straw, basketware | '46 | -0.72 | -0.13 | -0.08 | -0.38 | -0.76 | 0.21 | 1.73 | 0.78 | -0.46 | 1.16 | -0.11 | -0.58 | -0.52 | -0.71 | 0.18 | 0.59 | 0.86 | -0.46 | -0.75 | -0.93 | 1.55 |
| Pulp of wood or of other fibrous cellulosic material | '47 | -1.40 | -0.39 | -0.81 | -0.29 | 0.38 | -0.91 | -0.33 | -0.62 | 0.95 | 0.25 | 0.25 | 0.16 | 0.77 | -0.41 | -1.12 | -0.35 | -0.40 | -0.99 | -0.70 | 0.46 | -1.15 |
| Paper and paperboard; articles of paper pulp, of paper or of paperboard | '48 | 0.36 | -0.51 | -0.56 | -0.01 | -0.25 | -0.89 | -0.50 | 0.03 | 0.15 | -0.48 | -0.18 | -0.02 | 0.76 | -0.04 | -0.17 | 0.07 | -0.33 | -0.30 | -0.35 | 0.07 | -0.54 |
| Printed books, newspapers, pictures and products of the printing industry | '49 | -0.05 | -0.22 | -0.44 | 0.08 | -1.01 | -0.22 | -0.54 | -0.20 | -0.56 | -0.36 | 0.34 | 0.63 | -0.34 | -0.40 | -0.36 | -0.10 | -0.67 | -0.31 | 0.27 | 0.34 | -0.06 |
| Silk | '50 | -0.15 | -0.42 | -0.34 | -0.14 | -0.25 | -0.63 | -0.38 | -0.57 | -0.69 | -0.24 | -0.41 | -0.64 | -0.13 | -0.14 | -0.11 | 0.11 | -0.32 | -0.50 | -0.23 | -0.60 | 0.23 |
| Wool, fine or coarse animal hair; horsehair yarn and woven fabric | '51 | -0.55 | -0.02 | -0.33 | -0.07 | -0.27 | -0.35 | -0.71 | -0.33 | -0.07 | -1.03 | -0.06 | 0.13 | -0.22 | -0.18 | -0.06 | -0.48 | -1.38 | -0.15 | 0.09 | -0.13 | -0.17 |
| Cotton | '52 | -0.36 | -0.44 | -0.78 | 0.07 | -0.30 | 0.32 | -1.09 | -1.16 | -0.36 | -1.29 | -0.12 | 0.25 | -0.17 | 0.18 | 0.02 | -0.36 | -0.75 | 0.04 | -0.27 | 0.52 | -0.77 |
| Other vegetable textile fibres; paper yarn and woven fabrics of paper yarn | '53 | -0.67 | -0.09 | -0.27 | 0.11 | -0.34 | -1.17 | 0.10 | -0.11 | -0.85 | -0.86 | -0.03 | -0.33 | -0.30 | -0.14 | 0.10 | -0.80 | -0.28 | -1.06 | -0.03 | -0.29 | 0.50 |
| Man-made filaments; strip and the like of man-made textile materials | '54 | 0.69 | 0.38 | -0.48 | -0.16 | -0.30 | 0.45 | -0.76 | -0.53 | -0.65 | -0.97 | 0.25 | 0.01 | -0.33 | 0.15 | 1.01 | -0.03 | -1.19 | 0.03 | -0.03 | 0.21 | -0.50 |
| Man-made staple fibres | '55 | 0.20 | -0.05 | -0.36 | -0.06 | -0.40 | -0.08 | -0.84 | -0.77 | 0.02 | -0.61 | -0.07 | 0.21 | -0.05 | -0.06 | 0.76 | 0.44 | -0.57 | 0.17 | -0.01 | 0.33 | -0.69 |
| Wadding, felt and nonwovens; special yarns; twine, cordage, ropes | '56 | 0.47 | -0.25 | -0.50 | 0.09 | -0.50 | 0.72 | 0.14 | -0.53 | 0.42 | -0.90 | -0.29 | -0.22 | -0.04 | -0.04 | 0.51 | 0.18 | -0.53 | -0.10 | -0.09 | 0.26 | -0.36 |
| Carpets and other textile floor coverings | '57 | -0.06 | -0.19 | -0.47 | 0.36 | -1.06 | 1.15 | 0.02 | -0.23 | 0.25 | -0.03 | -0.42 | -0.26 | -0.58 | -0.28 | -0.17 | 0.62 | 0.20 | 0.75 | -0.45 | -0.13 | 0.40 |
| Special woven fabrics; tufted textile fabrics; lace; tapestries | '58 | 0.96 | -0.37 | -0.79 | 0.04 | -0.46 | 0.14 | -0.51 | -1.02 | -0.29 | -1.13 | -0.31 | -0.05 | -0.19 | 0.45 | 1.16 | 0.05 | -0.96 | 0.34 | -0.23 | 0.34 | -1.08 |
| Impregnated, coated, covered or laminated textile fabrics | '59 | 0.82 | -0.42 | -0.95 | 0.09 | -0.62 | -0.99 | -1.16 | -0.60 | 0.24 | -1.41 | -0.32 | -0.01 | -0.05 | 0.30 | 0.99 | -0.23 | -1.35 | 0.34 | 0.30 | 0.36 | -1.43 |
| Knitted or crocheted fabrics | '60 | 1.26 | -0.40 | -0.82 | 0.05 | -0.17 | 0.70 | -1.12 | -0.91 | -0.53 | -1.34 | -0.30 | 0.27 | 0.06 | 0.04 | 1.54 | -0.59 | -1.36 | 0.60 | -0.05 | 0.10 | -1.02 |
| Articles of apparel and clothing accessories, knitted or crocheted | '61 | 0.56 | 0.64 | 0.33 | -0.27 | -0.95 | 1.73 | 1.19 | 0.42 | 0.76 | 0.80 | 0.08 | -0.15 | -0.61 | -0.71 | 0.50 | 1.19 | 0.64 | 1.56 | -0.60 | -0.67 | 1.19 |
| Articles of apparel and clothing accessories, not knitted or crocheted | '62 | 0.23 | 0.38 | 0.59 | -0.31 | -0.99 | 1.65 | 1.36 | 0.97 | 0.58 | 0.97 | -0.11 | -0.09 | -0.62 | -0.63 | 0.11 | 1.09 | 0.55 | 1.40 | -0.54 | -0.88 | 0.54 |
| Other made-up textile articles; sets; worn clothing | '63 | 0.67 | -0.31 | 0.59 | -0.17 | -1.01 | 0.91 | 0.43 | 0.41 | 1.07 | 0.39 | -0.22 | 0.02 | -0.45 | -0.57 | 0.53 | 0.76 | 0.39 | 1.50 | -0.40 | -0.56 | 1.40 |
| Footwear, gaiters and the like | '64 | 0.32 | 0.02 | 0.12 | -0.17 | -0.86 | 0.77 | 0.11 | 0.26 | 0.74 | 0.53 | -0.16 | 0.68 | -0.62 | -0.64 | 0.25 | 1.12 | 0.51 | 0.31 | -0.59 | -0.98 | 0.49 |
| Headgear and parts thereof | '65 | 1.08 | 0.37 | 0.17 | -0.20 | -1.00 | 0.04 | 0.96 | 0.32 | 0.01 | 0.53 | -0.15 | -0.26 | -0.04 | -0.80 | 1.35 | 1.00 | 0.80 | 0.14 | -0.41 | -0.78 | 0.94 |
| Umbrellas, sun umbrellas, walking sticks | '66 | -0.79 | -0.15 | -0.26 | -0.20 | -0.77 | 0.02 | -0.56 | 0.35 | -0.42 | -0.23 | -0.23 | -0.42 | -0.51 | -0.42 | 0.82 | 0.33 | 0.71 | -0.33 | -0.28 | -0.83 | 0.53 |
| Prepared feathers and down; artificial flowers; articles of human hair | '67 | 0.25 | -0.06 | -0.40 | -0.18 | -0.67 | -0.07 | 0.80 | -0.55 | -0.57 | -0.30 | -0.11 | -0.39 | -0.34 | -0.56 | -0.07 | 1.29 | 0.51 | -0.47 | -0.44 | -1.07 | 1.10 |
| Articles of stone, plaster, cement, mica or similar materials | '68 | -0.35 | -0.09 | 0.21 | -0.13 | -0.73 | 0.33 | -0.29 | -0.01 | 0.33 | -0.77 | -0.53 | 0.53 | -0.13 | -0.22 | -0.25 | -0.13 | -0.13 | 0.59 | 0.13 | -0.28 | -0.13 |
| Ceramic products | '69 | -0.63 | 0.05 | 0.28 | -0.25 | -0.99 | -0.59 | 0.04 | -0.08 | 0.48 | 0.03 | -0.50 | 0.77 | -0.30 | -0.75 | -0.36 | 0.69 | 0.43 | 0.87 | -0.03 | -0.42 | 1.11 |
| Glass and glassware | '70 | -0.22 | -0.07 | 0.04 | -0.04 | -0.59 | -0.54 | -0.01 | 0.17 | 0.34 | 0.22 | -0.27 | 0.11 | -0.19 | -0.29 | -0.03 | 0.00 | -0.54 | 0.68 | -0.07 | 0.17 | -0.42 |
| Natural or cultured pearls, precious stones, imitation jewellery, coin | '71 | -0.12 | -0.34 | 0.02 | -0.06 | -0.03 | -0.80 | 0.20 | 0.33 | -0.31 | 0.66 | -0.10 | -0.15 | 0.12 | -0.12 | -0.52 | 0.02 | -0.13 | -0.22 | 0.01 | -0.03 | 0.27 |
| Iron and steel | '72 | 0.00 | -0.75 | -0.45 | 0.08 | -0.11 | -1.21 | -1.02 | 0.01 | -0.42 | 0.39 | -0.32 | -0.07 | 0.13 | -0.38 | -0.01 | -0.76 | -0.80 | 0.20 | 0.13 | -0.09 | -1.38 |
| Articles of iron or steel | '73 | 0.27 | -0.36 | -0.30 | -0.10 | -0.80 | -0.49 | -0.45 | 0.27 | 0.02 | 0.19 | -0.35 | 0.22 | -0.04 | 0.07 | 0.73 | -0.26 | -0.28 | 0.22 | 0.01 | -0.03 | -0.39 |
| Copper and articles thereof | '74 | -0.24 | -0.37 | -0.30 | -0.23 | -0.19 | -0.55 | 0.32 | 0.85 | -0.59 | -0.07 | -0.18 | 0.00 | 0.10 | -0.14 | -0.18 | -0.57 | -0.57 | -0.07 | -0.08 | -0.16 | -0.88 |
| Nickel and articles thereof | '75 | -1.26 | -0.14 | -0.51 | 0.78 | -0.18 | -0.77 | -0.15 | -0.41 | -0.16 | -0.66 | -0.26 | -0.87 | -0.73 | -0.22 | -1.15 | -0.32 | -0.03 | -0.47 | -0.03 | -0.02 | 0.13 |
| Aluminium and articles thereof | '76 | -0.49 | -0.40 | -0.57 | 0.02 | 0.27 | -0.79 | -0.47 | -0.05 | -0.13 | 0.55 | -0.42 | 0.05 | -0.04 | 0.01 | -0.31 | -0.45 | -0.29 | 0.02 | -0.06 | -0.05 | -0.75 |
| Lead and articles thereof | '78 | -0.60 | -0.25 | -0.53 | -0.33 | -0.61 | -1.33 | -0.76 | 0.19 | -0.92 | 0.71 | -0.07 | -0.91 | 0.66 | -0.21 | -0.67 | -1.16 | -0.75 | -0.88 | -0.17 | -0.11 | -1.28 |
| Zinc and articles thereof | '79 | 0.39 | -0.82 | 0.53 | 0.17 | 1.12 | -1.15 | -0.97 | 0.85 | -0.79 | 0.64 | -0.44 | 0.77 | -0.56 | -0.53 | -0.58 | 0.01 | -0.70 | -0.92 | -0.09 | -0.76 | -1.01 |
| Tin and articles thereof | '80 | -1.09 | 0.36 | -0.77 | -0.35 | -0.70 | -0.64 | -0.28 | -0.69 | -0.06 | -0.85 | 0.29 | -0.84 | -0.32 | -0.45 | -0.48 | 1.47 | -0.50 | -0.74 | -0.33 | -0.46 | 0.86 |
| Other base metals; cermets; articles thereof | '81 | -0.91 | -0.38 | -1.09 | 0.03 | -0.05 | -0.30 | 1.15 | -0.27 | -0.59 | -0.42 | -0.09 | -0.25 | -0.58 | -0.17 | -0.62 | -0.06 | -0.06 | -0.30 | -0.13 | 0.20 | 0.01 |
| Tools, implements, cutlery, spoons and forks, of base metal | '82 | 0.23 | -0.51 | -0.02 | -0.16 | -0.79 | 0.23 | -0.19 | -0.14 | -0.07 | -0.48 | -0.15 | -0.05 | 0.10 | 0.26 | 0.68 | -0.83 | -0.66 | -0.45 | 0.07 | 0.01 | -0.09 |
| Miscellaneous articles of base metal | '83 | 0.23 | -0.10 | -0.01 | -0.12 | -0.49 | -0.42 | -0.17 | -0.20 | 0.11 | -0.65 | -0.11 | 0.13 | -0.14 | -0.13 | 0.91 | -0.09 | -0.41 | -0.01 | -0.15 | 0.03 | -0.47 |
| Machinery, mechanical appliances, nuclear reactors, boilers | '84 | 0.13 | 0.15 | -0.06 | 0.03 | -0.58 | -0.84 | 0.22 | -0.21 | -0.30 | -0.34 | 0.12 | -0.16 | -0.03 | 0.16 | 0.21 | 0.07 | -0.71 | -0.45 | 0.08 | 0.17 | -0.43 |
| Electrical machinery and equipment; sound recorders and reproducers, tv | '85 | 0.09 | -0.04 | 0.03 | -0.14 | -0.48 | -0.48 | 0.08 | -0.01 | 0.09 | -0.10 | 0.05 | -0.08 | 0.04 | 0.00 | -0.01 | -0.02 | 0.12 | -0.06 | 0.08 | 0.12 | -0.18 |
| Railway or tramway locomotives; railway or tramway track | '86 | -0.37 | -0.73 | 0.22 | -0.03 | -0.78 | -1.59 | -0.47 | 0.38 | -0.42 | 0.32 | -0.75 | 0.54 | -0.19 | 0.00 | -0.66 | -0.42 | -0.77 | -0.72 | -0.30 | 0.14 | -0.47 |
| Vehicles other than railway or tramway rolling stock | '87 | 0.79 | -0.31 | 0.26 | -0.17 | -0.79 | -0.62 | -0.12 | 0.06 | 0.01 | -0.31 | -0.21 | 0.18 | 0.10 | -0.72 | 0.16 | 0.15 | -0.48 | 0.27 | -0.26 | -0.26 | -0.63 |
| Aircraft, spacecraft, and parts thereof | '88 | -0.21 | -0.43 | 0.08 | -0.21 | -0.90 | -0.74 | 0.01 | -0.02 | -0.22 | 0.21 | -0.80 | -0.07 | -0.33 | -0.34 | -0.95 | -1.07 | -0.76 | 0.41 | -0.09 | 0.45 | -0.16 |
| Ships, boats and floating structures | '89 | 1.02 | -0.39 | -0.14 | 0.27 | -0.21 | -0.46 | 0.22 | 0.21 | 0.11 | 1.27 | 0.26 | -0.11 | 0.28 | -0.26 | -0.10 | -0.44 | -0.39 | -0.43 | 0.12 | 0.12 | -1.00 |
| Optical, photographic, cinematographic, medical or surgical instruments | '90 | -0.58 | -0.18 | 0.05 | 0.08 | -0.42 | -0.03 | 0.09 | -0.49 | -0.38 | -0.65 | -0.05 | -0.33 | -0.06 | 0.28 | -0.43 | 0.01 | -0.40 | -0.70 | 0.07 | 0.33 | -0.40 |
| Clocks and watches and parts thereof | '91 | -0.02 | -0.39 | -0.14 | -0.31 | -0.50 | -0.27 | 0.36 | -0.29 | -0.66 | -0.15 | -0.13 | -0.45 | -0.42 | 0.35 | -0.33 | 0.13 | 0.07 | -0.47 | -0.44 | -0.60 | -0.06 |
| Musical instruments; parts and accessories of such articles | '92 | 0.45 | 0.56 | 0.14 | -0.10 | -0.96 | 0.44 | -0.31 | -0.32 | -0.46 | 0.79 | -0.18 | 0.00 | -0.38 | -0.38 | 0.59 | -0.09 | -0.32 | -0.16 | -0.44 | -0.28 | -0.23 |
| Arms and ammunition; parts and accessories thereof | '93 | -0.53 | -0.07 | -0.07 | -0.16 | -0.32 | 1.03 | 0.17 | -0.33 | 0.10 | 1.48 | -0.01 | -0.01 | -0.13 | -0.06 | 0.08 | -0.28 | -0.33 | -0.76 | 0.03 | 0.43 | -0.32 |
| Furniture; bedding, mattresses, stuffed furnishings; lamps | '94 | -0.08 | 0.84 | 0.51 | -0.33 | -0.64 | 0.20 | 0.62 | 0.76 | 0.19 | 0.78 | -0.33 | 0.36 | 0.03 | -0.55 | 0.64 | 0.89 | 0.06 | 0.29 | -0.33 | -0.43 | 1.13 |
| Toys, games and sports requisites | '95 | 0.06 | 0.18 | 0.17 | 0.16 | -0.71 | 1.29 | 0.41 | -0.04 | -0.48 | 0.48 | -0.17 | 0.03 | -0.30 | -0.17 | 0.91 | 0.75 | 0.16 | -0.18 | -0.36 | -0.47 | 0.62 |
| Miscellaneous manufactured articles | '96 | 0.55 | -0.16 | -0.02 | -0.08 | -0.91 | -0.21 | -0.22 | -0.46 | -0.46 | -1.16 | -0.12 | -0.04 | -0.32 | 0.07 | 0.61 | 0.11 | -0.46 | -0.38 | -0.18 | -0.21 | -0.10 |
| Works of art, collectors' pieces and antiques | '97 | 0.02 | 0.00 | 0.01 | -0.11 | 0.21 | 0.00 | 0.03 | 0.08 | 0.13 | 0.16 | -0.07 | -0.52 | -0.01 | -0.07 | -0.09 | 0.02 | 0.07 | -0.11 | 0.09 | 0.10 | 0.01 |
| Commodities not elsewhere specified | '99 | 0.00 | -0.34 | -0.26 | 0.06 | 0.82 | -0.08 | 0.02 | -0.09 | 0.15 | 0.09 | 0.58 | 0.33 | 0.06 | 0.00 | -0.54 | 0.70 | -0.28 | -0.20 | -0.50 | -0.18 | -0.09 |
| Parts and accessories for calculating machines | 8473 | 0.35 | 0.31 | -0.06 | -0.02 | -0.69 | 0.00 | -0.01 | -0.45 | -0.02 | 0.04 | 0.02 | -0.23 | -0.33 | -0.23 | 0.85 | 0.28 | -0.18 | -0.23 | -0.20 | 0.08 | 0.89 |
| Electronic integrated circuits; parts thereof | 8542 | -0.10 | -0.05 | -1.17 | -0.81 | -1.27 | -0.51 | 1.83 | -1.64 | 0.34 | -1.02 | 1.63 | -1.38 | -0.27 | -0.29 | 1.27 | -0.48 | -1.67 | -0.01 | 0.89 | 0.94 | -1.89 |
| Parts and accessories for tractors, motor vehicles | 8708 | 0.15 | -0.17 | -0.24 | -0.20 | -0.22 | 0.00 | 0.42 | 0.17 | -0.13 | 0.22 | -0.12 | -0.05 | -0.12 | -0.02 | 0.24 | -0.49 | -0.20 | -0.05 | -0.10 | 0.26 | -0.05 |
| Parts of aircraft and spacecraft | 8803 | -0.45 | -0.29 | 0.07 | 0.05 | -0.84 | 0.00 | 0.42 | 0.23 | -0.16 | -0.03 | -0.40 | 0.51 | 0.10 | -0.27 | -0.35 | -1.06 | -0.32 | -0.35 | 0.00 | 0.57 | -0.05 |
| Parts of telephone sets, telephones for cellular networks | 851770 | 0.51 | -0.03 | -0.09 | -0.25 | -0.59 | -1.21 | -0.30 | -0.45 | -0.50 | -0.15 | -0.04 | -0.49 | 0.07 | -0.17 | -0.16 | 0.33 | -0.62 | -0.58 | -0.44 | -0.07 | -0.41 |

**Table S 2 . RSACA. 2019**

| **Product label** | **Code** | **Bang** | **Belg** | **Brazil** | **Bulg** | **Camb** | **Chile** | **Chin** | **Col** | **Czech** | **Eth** | **Fin** | **Fran** | **Germ** | **Gre** | **Hon** | **Hung** | **India** | **Indo** | **Irel** | **Italy** | **Jap** |
| --- | --- | --- | --- | --- | --- | --- | --- | --- | --- | --- | --- | --- | --- | --- | --- | --- | --- | --- | --- | --- | --- | --- |
| Dairy products | '04 | -1.15 | -0.02 | -0.35 | -0.04 | -0.46 | -0.31 | -0.69 | -0.68 | 0.03 | -0.18 | 0.06 | 0.26 | -0.03 | 0.10 | 1.06 | 0.00 | 0.42 | -0.50 | 0.30 | -0.10 | -0.69 |
| Products of animal origin | '05 | -0.88 | -0.12 | 0.27 | -0.17 | -0.27 | -0.22 | 0.36 | 0.67 | -0.35 | 0.98 | -0.61 | -0.21 | -0.17 | -0.21 | 1.27 | 0.08 | 0.48 | -0.94 | 0.08 | -0.33 | -1.04 |
| Lac; gums, resins | '13 | -1.03 | -0.24 | 0.04 | -0.90 | -0.58 | 0.65 | 0.52 | -0.91 | 0.08 | 0.80 | -0.49 | 0.29 | -0.17 | 0.19 | 1.57 | -0.51 | 0.60 | 0.28 | -0.70 | 0.02 | -0.90 |
| Vegetable plaiting materials | '14 | -0.39 | -0.14 | 0.78 | -0.05 | -0.03 | 1.59 | -0.08 | -0.41 | -0.13 | -0.17 | -0.37 | -0.35 | -0.55 | -0.88 | 1.62 | 1.01 | 0.18 | 1.61 | -0.51 | -0.47 | -1.45 |
| Animal or vegetable fats and oils | '15 | -1.06 | -0.09 | 0.07 | 0.54 | 0.29 | -0.37 | -0.82 | 0.15 | 0.01 | -0.36 | -0.87 | -0.20 | -0.23 | 0.48 | 1.61 | 0.40 | -0.73 | 1.45 | -0.75 | -0.33 | -0.46 |
| Preparations of meat, fish, molluscs | '16 | 0.07 | -0.07 | 1.13 | 0.19 | -0.47 | 0.29 | 0.98 | -1.01 | -0.19 | -0.27 | -1.04 | -0.33 | -0.14 | -0.31 | 0.92 | -0.04 | 0.68 | 1.21 | -0.09 | -0.26 | -1.08 |
| Sugars and sugar confectionery | '17 | -1.53 | 0.17 | 1.56 | -0.07 | 0.35 | -0.94 | -0.02 | 0.51 | 0.13 | 0.88 | -0.39 | 0.42 | 0.08 | -0.34 | 1.42 | 0.08 | 0.94 | -0.77 | -0.67 | -0.52 | -0.44 |
| Cocoa and cocoa preparations | '18 | -0.13 | 0.11 | -0.03 | -0.01 | -0.14 | -0.68 | -0.15 | 0.40 | -0.28 | -0.42 | -0.44 | -0.20 | -0.02 | -0.47 | 1.34 | -0.25 | 0.03 | 0.18 | -0.43 | 0.18 | -0.61 |
| Preparations of cereals, flour | '19 | -0.21 | 0.30 | 0.00 | 0.27 | -1.00 | -0.15 | -0.58 | -0.05 | -0.09 | 0.19 | -0.70 | 0.15 | 0.10 | 0.13 | 1.05 | -0.29 | 0.48 | 0.37 | 0.11 | 0.51 | -0.23 |
| Preparations of vegetables, fruit, nuts | '20 | 0.05 | 0.31 | 0.61 | 0.04 | 0.24 | 0.40 | 0.57 | -0.08 | -0.43 | 0.93 | -1.07 | -0.31 | -0.38 | 0.61 | 1.24 | 0.27 | 0.58 | 0.03 | -0.81 | 0.41 | -1.11 |
| Miscellaneous edible preparations | '21 | -0.58 | 0.17 | 0.52 | -0.24 | -1.06 | -0.17 | -0.07 | 0.05 | -0.11 | -0.95 | -0.56 | 0.14 | 0.15 | 0.14 | 0.85 | 0.07 | 0.58 | 0.22 | -0.44 | 0.32 | -0.06 |
| Beverages, spirits and vinegar | '22 | 0.11 | 0.11 | -0.05 | -0.14 | -1.17 | 0.62 | -0.38 | -0.89 | -0.13 | -0.13 | -0.55 | 0.63 | -0.23 | 0.04 | 0.58 | 0.31 | -0.18 | 0.04 | 0.00 | 0.67 | -0.55 |
| Residues and waste from food industries | '23 | -1.22 | 0.09 | 1.25 | 0.41 | -1.05 | -0.30 | -0.20 | -1.18 | 0.00 | 0.20 | -0.61 | 0.08 | -0.04 | -0.58 | 1.44 | 0.29 | 0.57 | -0.51 | -0.67 | -0.40 | -0.86 |
| Tobacco and manufactured tobacco substitutes | '24 | 0.77 | 0.00 | 1.46 | -0.07 | -0.76 | 0.42 | -0.17 | -0.18 | 0.11 | -0.56 | -0.71 | -0.62 | 0.04 | 0.31 | 0.96 | -0.11 | 1.03 | 0.25 | -0.47 | -0.18 | -1.35 |
| Salt; sulphur; earths and stone; lime and cement | '25 | -1.55 | 0.01 | 0.01 | 0.12 | -1.27 | -0.10 | -0.47 | -0.76 | -0.03 | 0.37 | -0.32 | 0.04 | 0.00 | 0.96 | 1.15 | -0.53 | 0.13 | -0.36 | 0.17 | -0.11 | -0.31 |
| Ores, slag and ash | '26 | -0.13 | -0.25 | 1.20 | -0.11 | -0.08 | 1.36 | -1.59 | 0.14 | -0.38 | 0.97 | -0.59 | -0.32 | -0.64 | 0.30 | 1.04 | -0.21 | 0.29 | 0.77 | -0.15 | -0.31 | -1.34 |
| Mineral fuels, mineral oils and products of their distillation | '27 | -0.93 | -0.13 | 0.18 | -0.11 | -0.94 | -1.06 | -0.86 | 0.86 | -0.33 | -0.27 | -0.23 | -0.51 | -0.49 | 0.11 | -0.10 | -0.40 | -0.31 | 0.23 | -0.56 | -0.59 | -0.97 |
| Inorganic chemicals | '28 | -0.83 | 0.07 | 0.28 | 0.44 | -0.41 | 0.31 | 0.15 | -0.45 | -0.32 | -0.41 | 0.04 | 0.01 | 0.06 | 0.25 | 1.09 | 0.06 | -0.41 | -0.27 | 0.20 | -0.18 | -0.04 |
| Organic chemicals | '29 | -0.78 | 0.01 | -0.77 | -0.26 | -0.10 | -0.33 | -0.10 | -1.02 | -0.06 | -0.40 | -0.04 | -0.07 | -0.31 | -1.10 | 0.66 | 0.03 | 0.17 | -0.35 | 0.39 | -0.40 | 0.08 |
| Pharmaceutical products | '30 | -0.12 | 0.05 | -0.75 | -0.08 | -0.45 | -0.68 | -0.45 | -0.74 | -0.32 | -1.06 | -0.48 | 0.25 | 0.12 | 0.08 | 0.31 | 0.11 | 0.95 | -0.09 | 0.43 | 0.05 | -0.63 |
| Fertilisers | '31 | -1.63 | 0.33 | -1.48 | 0.05 | -1.47 | 0.10 | 0.29 | -0.81 | -0.34 | -1.33 | 0.45 | -0.55 | -0.04 | 0.11 | 1.58 | -0.47 | -1.37 | -0.38 | -0.99 | -0.26 | -0.35 |
| Tanning or dyeing extracts; paints and varnishes | '32 | -1.52 | 0.15 | -0.60 | -0.68 | -0.90 | -0.82 | 0.10 | -0.54 | -0.35 | -1.07 | -0.09 | -0.02 | 0.23 | 0.08 | 1.02 | -0.51 | 0.45 | -0.51 | -0.21 | 0.02 | 0.55 |
| Essential oils; perfumery, cosmetics | '33 | -0.60 | -0.06 | 0.04 | 0.06 | -0.72 | -0.90 | -0.56 | 0.04 | -0.15 | -0.38 | -0.54 | 0.51 | 0.05 | -0.17 | 0.02 | -0.09 | 0.43 | -0.28 | 0.52 | 0.22 | 0.20 |
| Soap, washing preparations, waxes, candles | '34 | -1.04 | 0.22 | -0.30 | -0.21 | -1.01 | -1.05 | -0.14 | 0.00 | -0.03 | -1.10 | -0.49 | 0.16 | 0.17 | -0.10 | 1.19 | -0.04 | 0.05 | 0.27 | -0.83 | 0.17 | 0.47 |
| Albuminoidal substances | '35 | -1.05 | 0.22 | 0.08 | 0.26 | -1.07 | -0.77 | -0.17 | -0.39 | -0.68 | -1.10 | 0.18 | 0.36 | 0.14 | -0.61 | 1.16 | -0.38 | -0.18 | -0.95 | 0.45 | 0.07 | -0.14 |
| Explosives; pyrotechnic products; matches | '36 | -0.37 | 0.02 | 0.35 | 0.34 | -0.39 | 0.01 | 0.81 | -1.46 | 0.72 | -1.11 | -0.14 | 0.42 | -0.07 | -0.06 | 1.83 | -0.72 | 1.08 | -1.03 | 0.08 | -0.35 | -0.63 |
| Photographic or cinematographic goods | '37 | -1.00 | 0.30 | -0.56 | -0.14 | -0.11 | -0.66 | -0.56 | -0.88 | -0.24 | -0.79 | -0.41 | -0.19 | 0.04 | -0.72 | 1.19 | -0.13 | -0.58 | -0.68 | -0.98 | -0.55 | 1.14 |
| Miscellaneous chemical products | '38 | -0.98 | 0.12 | -0.80 | -0.08 | -0.83 | -0.78 | -0.12 | -0.19 | -0.33 | -1.13 | 0.05 | 0.24 | 0.14 | -0.08 | 0.50 | -0.19 | 0.17 | 0.21 | 0.42 | -0.10 | 0.35 |
| Plastics and articles thereof | '39 | -0.99 | 0.19 | -0.42 | -0.16 | -0.39 | -0.67 | -0.03 | -0.07 | -0.23 | -0.66 | -0.03 | -0.01 | 0.07 | 0.01 | -0.20 | -0.11 | -0.12 | -0.62 | -0.51 | -0.05 | 0.22 |
| Rubber and articles thereof | '40 | -0.70 | 0.01 | -0.28 | -0.01 | 0.25 | -0.52 | 0.07 | -0.90 | 0.13 | -1.11 | -0.20 | 0.02 | -0.05 | -0.61 | 0.68 | 0.12 | 0.22 | 0.47 | -0.49 | -0.06 | 0.40 |
| Raw hides and skins (other than furskins) and leather | '41 | 0.01 | 0.15 | 1.38 | -0.62 | -1.04 | 0.42 | -0.84 | 0.83 | -0.50 | 1.09 | 0.16 | -0.02 | -0.01 | 0.45 | 0.56 | -0.85 | 0.22 | -0.84 | 0.56 | 0.08 | -0.10 |
| Articles of leather; saddlery; travel goods, handbags | '42 | 0.88 | -0.05 | -0.65 | 0.05 | 1.38 | 0.19 | 0.80 | -0.35 | -0.19 | 1.22 | -0.53 | 0.25 | -0.39 | -0.50 | -0.10 | 0.15 | 0.89 | 0.15 | -0.49 | 0.32 | -1.30 |
| Furskins and artificial fur; manufactures thereof | '43 | -0.08 | -0.13 | 0.40 | 0.24 | -0.01 | -0.08 | 0.29 | 0.48 | -0.20 | -0.09 | 0.02 | -0.23 | -0.35 | 0.07 | 0.35 | -0.20 | 0.05 | -0.35 | -0.06 | -0.07 | -0.53 |
| Wood and articles of wood | '44 | -0.20 | -0.01 | 1.23 | 0.22 | 0.02 | 1.02 | -0.34 | -0.44 | 0.35 | -0.56 | 0.38 | -0.16 | -0.05 | -0.43 | 0.81 | -0.04 | -0.39 | 0.91 | -0.30 | -0.40 | -1.22 |
| Cork and articles of cork | '45 | -0.11 | -0.27 | -0.60 | -0.15 | -0.30 | -0.29 | -0.14 | -0.18 | -0.68 | -0.92 | -0.91 | -0.37 | -0.61 | -0.89 | 1.93 | -0.43 | -0.06 | -0.45 | -0.78 | -0.68 | -0.35 |
| Manufactures of straw, basketware | '46 | 1.49 | -0.34 | -0.50 | -0.43 | 0.28 | -0.73 | 1.48 | -0.20 | -0.65 | 0.48 | -0.88 | -0.67 | -0.61 | -1.08 | 1.69 | -0.42 | 0.84 | 1.51 | -0.75 | -0.56 | -1.41 |
| Pulp of wood or of other fibrous cellulosic material | '47 | -1.25 | -0.04 | 1.33 | 0.79 | -0.01 | 1.61 | -1.50 | -1.07 | 0.28 | -0.21 | 0.75 | -0.17 | -0.59 | -0.09 | 1.44 | -0.37 | -1.26 | 0.20 | -0.17 | -0.95 | -0.27 |
| Paper and paperboard; articles of paper pulp, of paper or of paperboard | '48 | -1.02 | -0.08 | 0.41 | -0.21 | -1.24 | -0.22 | 0.52 | -0.25 | -0.19 | -1.08 | 0.90 | -0.10 | 0.08 | -0.48 | 0.33 | -0.21 | 0.04 | 0.45 | -0.84 | 0.02 | -0.11 |
| Printed books, newspapers, pictures and products of the printing industry | '49 | -0.70 | 0.03 | -0.30 | 0.24 | -0.73 | -0.58 | 0.16 | -0.10 | 0.11 | -1.16 | -0.38 | -0.03 | 0.12 | 0.15 | 0.53 | 0.14 | 0.23 | -0.48 | -0.63 | 0.28 | -0.38 |
| Silk | '50 | -1.28 | -0.12 | 0.58 | -1.14 | -1.30 | -0.16 | 1.07 | -0.28 | -0.16 | -1.43 | -0.15 | -0.10 | -0.20 | -0.31 | 1.07 | -0.40 | -0.24 | -0.22 | -0.11 | -0.12 | -0.55 |
| Wool, fine or coarse animal hair; horsehair yarn and woven fabric | '51 | -1.36 | 0.05 | 0.25 | -0.06 | -1.45 | 0.66 | -0.28 | -0.40 | 0.02 | -0.84 | -0.48 | -0.29 | -0.23 | -0.06 | 0.65 | -0.12 | -0.05 | -1.00 | -0.24 | 0.03 | -0.44 |
| Cotton | '52 | -1.24 | -0.01 | 1.29 | -0.74 | -1.73 | -0.39 | 0.01 | -1.20 | -0.18 | 0.31 | -0.06 | -0.11 | -0.13 | 1.01 | 0.29 | -0.19 | 0.63 | -0.45 | -0.05 | -0.05 | -0.13 |
| Other vegetable textile fibres; paper yarn and woven fabrics of paper yarn | '53 | 0.07 | 0.31 | 0.31 | -0.42 | -0.30 | -0.42 | -0.01 | -0.28 | -0.29 | -0.47 | -0.10 | 0.95 | -0.19 | -0.40 | 1.49 | -0.14 | 0.18 | -0.86 | -0.11 | 0.01 | -0.44 |
| Man-made filaments; strip and the like of man-made textile materials | '54 | -1.79 | -0.07 | -1.22 | -0.37 | -1.26 | -0.64 | 0.76 | -0.61 | -0.17 | -1.02 | -0.47 | -0.24 | -0.19 | 0.09 | 0.52 | -0.27 | 0.49 | -0.35 | -0.17 | -0.04 | 0.34 |
| Man-made staple fibres | '55 | -1.53 | 0.24 | -1.15 | -0.12 | -1.48 | -0.67 | 0.71 | -1.18 | -0.24 | -1.24 | -0.55 | 0.00 | -0.28 | -0.26 | 0.72 | -0.45 | 0.48 | 0.18 | 0.38 | -0.11 | 0.35 |
| Wadding, felt and nonwovens; special yarns; twine, cordage, ropes | '56 | -0.63 | -0.06 | -0.37 | 0.06 | -1.24 | -1.06 | 0.60 | -0.80 | -0.10 | -1.01 | -0.02 | -0.02 | 0.10 | 0.32 | 0.92 | 0.17 | 0.22 | -0.62 | -0.69 | 0.26 | -0.10 |
| Carpets and other textile floor coverings | '57 | 0.37 | 0.75 | -0.64 | -0.32 | -0.41 | -1.05 | 1.00 | -0.69 | -0.39 | -0.96 | -0.95 | -0.32 | -0.48 | -0.38 | 1.33 | -0.46 | 1.20 | -0.22 | -0.81 | -0.11 | -0.93 |
| Special woven fabrics; tufted textile fabrics; lace; tapestries | '58 | -1.69 | 0.28 | -0.50 | -0.57 | -1.70 | -0.64 | 0.95 | -0.23 | -0.42 | -0.34 | -0.17 | 0.21 | 0.15 | -0.08 | 0.30 | -0.71 | 0.43 | -1.01 | -0.32 | 0.13 | -0.04 |
| Impregnated, coated, covered or laminated textile fabrics | '59 | -1.66 | 0.38 | -0.65 | -0.86 | -0.73 | -1.08 | 0.57 | -0.21 | -0.13 | -1.23 | -0.14 | -0.05 | 0.19 | -0.50 | 0.48 | -0.36 | -0.37 | -0.78 | -0.39 | 0.21 | 0.23 |
| Knitted or crocheted fabrics | '60 | -1.52 | -0.03 | -0.85 | -0.88 | -1.07 | -0.40 | 0.98 | -0.41 | -0.22 | -0.21 | -0.09 | 0.03 | 0.03 | 0.04 | -0.14 | -0.35 | -0.01 | -1.23 | -0.22 | 0.09 | 0.40 |
| Articles of apparel and clothing accessories, knitted or crocheted | '61 | 1.80 | -0.06 | -0.51 | 0.35 | 1.75 | -1.21 | 1.09 | -0.18 | -0.30 | 1.57 | -0.77 | -0.39 | -0.43 | -0.15 | -0.56 | -0.38 | 1.15 | 0.90 | -0.88 | -0.03 | -1.20 |
| Articles of apparel and clothing accessories, not knitted or crocheted | '62 | 1.52 | -0.19 | -0.51 | 0.50 | 1.31 | -1.18 | 1.02 | -0.01 | -0.22 | 0.68 | -0.64 | -0.28 | -0.37 | -0.36 | -0.57 | -0.32 | 1.14 | 0.98 | -0.84 | 0.11 | -1.23 |
| Other made-up textile articles; sets; worn clothing | '63 | 1.12 | -0.10 | -0.44 | -0.03 | 0.14 | -1.17 | 1.36 | -0.10 | -0.01 | -0.09 | -0.62 | -0.47 | -0.34 | -0.33 | 0.48 | -0.18 | 1.12 | 0.00 | -0.69 | -0.34 | -1.06 |
| Footwear, gaiters and the like | '64 | 0.80 | 0.10 | 0.42 | 0.01 | 0.77 | -1.26 | 0.87 | -0.82 | -0.27 | 0.96 | -0.43 | -0.26 | -0.31 | -0.26 | -0.43 | -0.13 | 0.72 | 0.73 | -0.74 | 0.16 | -0.97 |
| Headgear and parts thereof | '65 | 1.02 | -0.06 | -0.76 | -0.07 | 1.07 | -1.10 | 1.31 | -0.85 | -0.12 | 0.93 | -0.91 | -0.18 | -0.31 | -0.43 | 1.12 | -0.37 | 0.28 | 0.17 | -0.65 | 0.12 | -0.59 |
| Umbrellas, sun umbrellas, walking sticks | '66 | -0.90 | -0.26 | -1.07 | -0.56 | 0.27 | -0.85 | 1.60 | -0.99 | -0.19 | -1.61 | -0.53 | -0.80 | -0.64 | -0.47 | 1.41 | -0.58 | -0.51 | -0.75 | -0.61 | -0.42 | -1.41 |
| Prepared feathers and down; artificial flowers; articles of human hair | '67 | 0.22 | -0.30 | -0.74 | -0.25 | 0.87 | -0.68 | 1.06 | -0.99 | -0.37 | 0.22 | -0.51 | -0.69 | -0.48 | -0.78 | 0.49 | -0.57 | 0.90 | 0.33 | -0.66 | -0.51 | -0.94 |
| Articles of stone, plaster, cement, mica or similar materials | '68 | -0.82 | 0.10 | 0.36 | -0.04 | -1.12 | -0.74 | 0.72 | -0.24 | 0.06 | -0.63 | -0.03 | -0.24 | 0.02 | 0.67 | 0.25 | -0.04 | 0.47 | -0.58 | -0.37 | 0.30 | 0.19 |
| Ceramic products | '69 | -0.33 | -0.17 | 0.18 | 0.09 | -1.55 | -1.30 | 1.11 | -0.36 | 0.01 | -0.83 | -0.61 | -0.54 | -0.09 | -0.62 | 0.32 | 0.12 | 0.63 | -0.40 | -0.62 | 0.56 | 0.03 |
| Glass and glassware | '70 | -0.74 | 0.14 | -0.43 | 0.50 | -0.95 | -0.54 | 0.31 | -0.06 | 0.24 | -0.80 | -0.15 | 0.01 | 0.00 | -0.59 | -0.41 | 0.09 | 0.05 | -0.18 | -0.62 | 0.04 | 0.16 |
| Natural or cultured pearls, precious stones, imitation jewellery, coin | '71 | -0.73 | 0.04 | 0.58 | 0.09 | 0.55 | 0.74 | -0.54 | 1.14 | -0.07 | 1.19 | 0.33 | 0.10 | -0.15 | 0.00 | -1.37 | 0.01 | -0.01 | 0.56 | -0.18 | 0.02 | -0.05 |
| Iron and steel | '72 | -1.37 | 0.15 | 0.77 | -0.25 | -1.04 | -0.50 | 0.15 | -0.30 | -0.25 | -1.43 | 0.23 | 0.15 | -0.13 | -0.11 | -0.12 | -0.26 | 0.14 | -0.13 | -0.40 | -0.24 | 0.60 |
| Articles of iron or steel | '73 | -1.06 | -0.03 | -0.24 | -0.08 | -0.81 | -0.73 | 0.78 | -0.60 | 0.07 | -1.07 | 0.06 | -0.11 | 0.02 | 0.28 | -0.14 | -0.29 | 0.39 | -0.52 | -0.60 | 0.41 | 0.14 |
| Copper and articles thereof | '74 | -0.50 | -0.03 | -0.18 | 0.44 | -0.08 | 1.56 | -0.92 | -0.05 | -0.48 | -0.47 | 0.43 | -0.01 | -0.04 | 0.21 | -0.18 | -0.46 | -0.59 | 0.19 | -0.24 | -0.32 | 0.50 |
| Nickel and articles thereof | '75 | -0.05 | -0.24 | -0.43 | -0.24 | -0.02 | -0.07 | -0.87 | -0.12 | -0.38 | -0.25 | 0.02 | -0.19 | -0.14 | -0.06 | 0.81 | -0.37 | -0.64 | 0.93 | -0.38 | -0.37 | -0.52 |
| Aluminium and articles thereof | '76 | -0.86 | -0.03 | -0.44 | -0.02 | -0.89 | -0.46 | 0.57 | 0.10 | -0.25 | -0.87 | -0.09 | -0.08 | -0.14 | 0.39 | -0.06 | -0.18 | 0.27 | -0.60 | -0.44 | -0.06 | -0.60 |
| Lead and articles thereof | '78 | -1.34 | 0.52 | -0.80 | 0.23 | 0.19 | 0.51 | -0.48 | -0.48 | -0.35 | 0.06 | -0.22 | 0.26 | -0.22 | -0.21 | 1.48 | -0.41 | 0.00 | -0.91 | -0.18 | -0.36 | -0.14 |
| Zinc and articles thereof | '79 | -1.19 | 0.15 | 0.31 | 1.06 | -0.28 | -0.59 | -0.78 | -0.98 | -0.49 | -0.42 | 1.58 | -0.06 | -0.46 | -0.65 | 0.40 | -0.46 | 0.13 | -1.25 | -0.01 | -0.55 | 0.38 |
| Tin and articles thereof | '80 | -0.64 | 0.20 | 1.31 | -0.66 | -0.29 | -0.47 | -0.16 | -0.58 | -0.43 | -0.04 | -0.21 | -0.23 | -0.72 | -0.23 | 0.87 | -0.37 | -1.01 | 1.44 | -0.15 | -0.58 | -0.83 |
| Other base metals; cermets; articles thereof | '81 | -0.10 | -0.20 | -0.05 | -0.01 | -0.29 | 0.80 | -0.03 | -0.14 | -0.24 | -0.07 | -0.09 | -0.04 | -0.22 | -0.40 | 0.36 | -0.17 | -0.65 | -0.52 | -0.69 | -0.46 | -0.14 |
| Tools, implements, cutlery, spoons and forks, of base metal | '82 | -0.58 | -0.06 | -0.30 | -0.33 | -0.59 | -0.99 | 0.62 | -0.46 | -0.05 | -0.82 | -0.40 | -0.21 | 0.10 | -0.48 | -0.18 | -0.51 | 0.06 | -0.77 | -0.33 | -0.04 | 0.38 |
| Miscellaneous articles of base metal | '83 | -0.95 | -0.31 | -0.43 | 0.01 | -1.02 | -0.72 | 0.93 | -0.69 | 0.03 | -0.99 | -0.18 | -0.25 | 0.11 | -0.05 | -0.34 | -0.21 | 0.02 | -0.84 | -0.28 | 0.26 | -0.22 |
| Machinery, mechanical appliances, nuclear reactors, boilers | '84 | -0.91 | -0.06 | -0.25 | -0.06 | -0.59 | -0.89 | 0.28 | -0.81 | 0.05 | -0.07 | 0.00 | -0.04 | 0.14 | -0.14 | -0.96 | 0.03 | -0.13 | -0.71 | -0.31 | 0.32 | 0.33 |
| Electrical machinery and equipment; sound recorders and reproducers, tv | '85 | -0.55 | -0.13 | -0.65 | 0.08 | -0.08 | -0.70 | 0.06 | -0.68 | -0.03 | -0.27 | -0.11 | -0.04 | -0.06 | -0.17 | -1.54 | 0.05 | -0.31 | -0.34 | 0.04 | -0.10 | 0.05 |
| Railway or tramway locomotives; railway or tramway track | '86 | -0.98 | -0.14 | -0.30 | 0.04 | -0.65 | -1.20 | 0.90 | -0.32 | 0.16 | -0.27 | -0.65 | -0.03 | -0.16 | -0.45 | 0.04 | 0.11 | -0.08 | -0.41 | -0.19 | -0.07 | 0.27 |
| Vehicles other than railway or tramway rolling stock | '87 | -0.52 | -0.05 | -0.14 | -0.30 | -0.64 | -1.08 | -0.10 | -0.72 | 0.30 | -0.79 | -0.13 | -0.10 | 0.17 | -0.62 | -0.14 | 0.26 | 0.57 | 0.05 | -0.63 | -0.15 | 0.86 |
| Aircraft, spacecraft, and parts thereof | '88 | -0.92 | 0.03 | 0.34 | -0.11 | -0.12 | -0.16 | -0.70 | -1.16 | -0.06 | -1.15 | -0.49 | 0.24 | 0.16 | -0.16 | -0.42 | -0.59 | -0.28 | -0.31 | -0.67 | 0.05 | -0.44 |
| Ships, boats and floating structures | '89 | -1.28 | -0.01 | -0.24 | -0.08 | -0.18 | -0.24 | 0.86 | -0.03 | -0.01 | 0.00 | 1.02 | 0.39 | 0.20 | -0.65 | -0.17 | -0.02 | 0.20 | -0.65 | -0.19 | 0.34 | 1.18 |
| Optical, photographic, cinematographic, medical or surgical instruments | '90 | -0.43 | -0.03 | -0.65 | 0.04 | -0.23 | -0.73 | -0.27 | -0.81 | -0.07 | -0.62 | 0.22 | -0.02 | 0.18 | -0.25 | -0.94 | 0.13 | -0.25 | -0.48 | 0.44 | -0.09 | 0.15 |
| Clocks and watches and parts thereof | '91 | -0.19 | -0.06 | -0.49 | -0.43 | 0.12 | -0.36 | -0.05 | -0.60 | -0.23 | -0.08 | -0.32 | 0.02 | -0.23 | -0.34 | -1.59 | -0.18 | -0.24 | -0.60 | -0.37 | -0.15 | -0.66 |
| Musical instruments; parts and accessories of such articles | '92 | -0.17 | -0.11 | -0.88 | 0.10 | -0.05 | -1.06 | 0.49 | -0.84 | -0.02 | -0.21 | -0.92 | -0.11 | -0.07 | -0.38 | -0.42 | -0.26 | -0.07 | 0.38 | -0.73 | 0.02 | 0.12 |
| Arms and ammunition; parts and accessories thereof | '93 | -1.00 | -0.08 | 0.78 | 0.00 | 0.68 | -0.43 | 0.10 | -0.09 | 0.28 | -0.05 | 0.37 | -0.17 | 0.07 | 0.26 | 0.08 | -0.42 | 0.30 | -1.05 | -0.25 | 0.51 | -0.08 |
| Furniture; bedding, mattresses, stuffed furnishings; lamps | '94 | -0.22 | -0.25 | -0.13 | 0.25 | 0.61 | -0.90 | 1.23 | -0.36 | 0.09 | -0.76 | -0.52 | -0.50 | -0.23 | -0.36 | -0.33 | 0.00 | 0.16 | 0.21 | -0.60 | 0.49 | -0.76 |
| Toys, games and sports requisites | '95 | 0.04 | -0.14 | -0.62 | 0.23 | -0.03 | -1.11 | 1.25 | -0.80 | 0.24 | -0.16 | -0.53 | -0.46 | -0.31 | 0.05 | -1.04 | 0.01 | 0.02 | 0.07 | -0.67 | -0.22 | -0.32 |
| Miscellaneous manufactured articles | '96 | -1.31 | 0.06 | -0.31 | -0.43 | -1.07 | -0.94 | 0.82 | -0.06 | 0.27 | -0.81 | -0.77 | -0.13 | 0.03 | -0.06 | -0.79 | 0.20 | 0.17 | -0.26 | -0.38 | 0.04 | 0.37 |
| Works of art, collectors' pieces and antiques | '97 | -0.01 | -0.25 | 0.87 | -0.02 | -0.05 | -0.13 | -0.16 | 0.10 | -0.10 | 0.64 | -0.10 | 0.35 | -0.06 | 0.21 | -1.67 | -0.20 | 0.04 | -0.02 | 0.05 | 0.09 | -0.26 |
| Commodities not elsewhere specified | '99 | -0.51 | -0.02 | 0.00 | -0.02 | 0.00 | 0.85 | -0.20 | -0.66 | -0.01 | 1.02 | -0.70 | -0.02 | 0.03 | -0.36 | -0.16 | -0.36 | -0.33 | -0.28 | -0.81 | 0.32 | 0.36 |
| Parts and accessories for calculating machines | 8473 | -0.21 | -0.09 | 0.06 | -1.47 | -0.01 | 0.03 | 1.29 | -0.17 | -0.39 | -0.18 | -0.27 | -0.06 | -0.27 | -0.37 | 0.28 | -0.54 | -0.37 | 0.02 | -0.34 | -0.06 | -0.32 |
| Electronic integrated circuits; parts thereof | 8542 | -0.01 | -0.08 | 0.01 | -1.09 | -0.40 | -0.01 | 1.01 | -1.98 | 0.24 | -1.75 | 0.19 | 0.47 | 0.28 | -1.70 | 1.95 | -1.73 | -0.23 | -1.06 | 0.48 | 0.04 | 0.93 |
| Parts and accessories for tractors, motor vehicles | 8708 | -0.20 | -0.22 | -0.45 | -0.05 | -0.03 | -0.42 | 0.02 | -0.68 | 0.06 | -0.51 | -0.85 | -0.03 | 0.11 | -0.34 | 0.24 | 0.06 | 0.28 | -0.34 | -0.21 | 0.21 | 0.66 |
| Parts of aircraft and spacecraft | 8803 | -0.18 | 0.31 | -0.07 | -0.17 | -0.25 | -0.02 | -0.13 | -0.16 | 0.29 | -0.39 | -0.37 | -0.09 | 0.06 | -0.15 | 0.89 | 0.05 | 0.68 | -0.46 | -0.23 | -0.73 | 0.35 |
| Parts of telephone sets, telephones for cellular networks | 851770 | -0.72 | -0.05 | -1.26 | -0.17 | -0.09 | -0.23 | 0.02 | -0.20 | 0.08 | -0.66 | -0.17 | -0.18 | -0.06 | -0.11 | 0.22 | -0.37 | -1.18 | -1.39 | -0.28 | -0.16 | -0.08 |

| **Product label** | **Code** | **Kor** | **Mal** | **Mex** | **Neth** | **Nor** | **Paki** | **Phil** | **Pol** | **Port** | **Rom** | **Sing** | **Spa** | **Swe** | **Switz** | **Taiwan** | **Thai** | **Tun** | **Turk** | **UK** | **US** | **Viet** |
| --- | --- | --- | --- | --- | --- | --- | --- | --- | --- | --- | --- | --- | --- | --- | --- | --- | --- | --- | --- | --- | --- | --- |
| Dairy products | '04 | -0.53 | -0.33 | -0.77 | 0.25 | -0.22 | -0.04 | -1.16 | 0.38 | -0.13 | -0.39 | -0.50 | -0.06 | -0.58 | 0.00 | -0.59 | -0.33 | -0.22 | 0.66 | -0.06 | 0.40 | -0.49 |
| Products of animal origin | '05 | -0.62 | -0.23 | -0.77 | 0.03 | -0.31 | 1.32 | -0.51 | 0.06 | 0.11 | -0.30 | -0.23 | 0.15 | -0.06 | -0.40 | -0.12 | -0.02 | -0.01 | 0.14 | 0.04 | 0.29 | -0.75 |
| Lac; gums, resins | '13 | -0.51 | -0.69 | -0.09 | -0.31 | -0.60 | 0.62 | 0.79 | -0.72 | -0.84 | -0.78 | -0.23 | 0.36 | -0.42 | 0.05 | -0.56 | -0.68 | -0.50 | -0.35 | -0.20 | -0.22 | -0.17 |
| Vegetable plaiting materials | '14 | -1.42 | 1.24 | 0.80 | -0.33 | -0.56 | -0.09 | 0.17 | -1.16 | -0.55 | 0.04 | -0.32 | -0.50 | -0.19 | -0.37 | -0.83 | -0.71 | -0.19 | 0.30 | -0.97 | -0.40 | 0.76 |
| Animal or vegetable fats and oils | '15 | -0.61 | 0.58 | -0.38 | -0.09 | -0.70 | -1.17 | 0.26 | -0.34 | 0.18 | 0.23 | -0.42 | 0.29 | -0.15 | -0.33 | -0.28 | 0.19 | 0.46 | -0.04 | -0.26 | -0.12 | -0.48 |
| Preparations of meat, fish, molluscs | '16 | -0.60 | 0.23 | -0.35 | -0.08 | -0.59 | 0.15 | 0.93 | 0.80 | -0.02 | -0.09 | -0.53 | 0.11 | -0.58 | -0.64 | -0.19 | 1.13 | 0.69 | 0.38 | -0.90 | -0.31 | 1.39 |
| Sugars and sugar confectionery | '17 | -0.56 | -0.68 | 0.37 | 0.22 | -0.88 | 1.28 | -0.57 | 0.27 | -0.26 | -0.79 | -0.35 | -0.07 | -0.32 | -0.17 | -0.33 | 1.17 | -0.25 | 0.70 | -0.27 | -0.18 | -0.43 |
| Cocoa and cocoa preparations | '18 | -0.38 | 0.06 | 0.12 | 0.00 | -0.66 | -0.42 | -0.62 | 0.12 | -0.75 | -0.43 | 0.08 | -0.12 | -0.17 | 0.11 | -0.28 | -0.28 | 0.01 | 0.13 | -0.30 | -0.26 | -0.10 |
| Preparations of cereals, flour | '19 | 0.11 | 0.21 | 0.50 | 0.26 | -1.00 | 0.05 | -0.04 | 0.53 | -0.11 | -0.35 | 0.45 | 0.18 | -0.14 | -0.11 | -0.07 | 0.46 | 0.74 | 1.09 | -0.16 | -0.15 | 0.01 |
| Preparations of vegetables, fruit, nuts | '20 | -0.49 | -0.41 | 0.32 | 0.20 | -0.86 | 0.44 | 0.42 | 0.20 | 0.13 | -0.70 | -0.30 | 0.47 | -0.76 | -0.60 | -0.32 | 0.82 | 0.00 | 1.29 | -0.67 | -0.10 | 0.69 |
| Miscellaneous edible preparations | '21 | -0.31 | 0.05 | -0.15 | 0.24 | -0.70 | 0.01 | -0.87 | 0.24 | -0.27 | -0.42 | 0.76 | 0.15 | -0.10 | 0.06 | -0.15 | 0.44 | -0.03 | 0.15 | 0.02 | 0.26 | -0.15 |
| Beverages, spirits and vinegar | '22 | -0.14 | -0.05 | 0.90 | 0.10 | -0.87 | 1.36 | -0.65 | -0.10 | 0.46 | -0.40 | 0.00 | 0.47 | -0.29 | -0.09 | -0.50 | 0.68 | 0.27 | 0.13 | 0.24 | -0.38 | -0.02 |
| Residues and waste from food industries | '23 | -0.85 | -0.44 | -0.63 | 0.17 | -0.35 | 0.15 | -1.15 | -0.15 | -0.30 | -0.26 | 0.12 | -0.22 | -0.38 | -0.31 | -0.51 | -0.08 | -0.07 | -0.80 | -0.18 | 0.74 | -0.76 |
| Tobacco and manufactured tobacco substitutes | '24 | 0.36 | -0.29 | 0.17 | 0.11 | -1.29 | 0.28 | 0.53 | 0.51 | 0.50 | 0.45 | 0.09 | -0.79 | 0.31 | 0.29 | -0.26 | -0.10 | -0.33 | 0.33 | -0.43 | 0.11 | 0.14 |
| Salt; sulphur; earths and stone; lime and cement | '25 | -0.28 | -0.05 | 0.20 | -0.09 | 0.23 | 0.82 | -1.06 | -0.47 | 0.43 | -0.21 | -0.61 | 0.39 | -0.47 | -0.47 | -0.65 | 0.56 | 0.21 | 0.92 | 0.02 | 0.04 | 0.85 |
| Ores, slag and ash | '26 | -1.25 | -0.30 | 0.55 | -0.23 | -0.43 | 0.51 | 0.69 | -0.44 | 0.72 | -0.30 | -0.01 | -0.22 | 0.71 | 0.00 | -0.56 | -0.09 | 0.05 | 0.12 | -0.24 | 0.36 | -0.45 |
| Mineral fuels, mineral oils and products of their distillation | '27 | -0.54 | 0.04 | -0.19 | -0.04 | 1.02 | -1.12 | -0.75 | -0.46 | -0.27 | -0.25 | -0.19 | -0.30 | -0.21 | -0.28 | -0.60 | -0.63 | -0.50 | -0.68 | 0.08 | 0.25 | -0.43 |
| Inorganic chemicals | '28 | -0.24 | -0.51 | -0.45 | 0.16 | -0.46 | -0.93 | -0.45 | -0.14 | -0.53 | -0.04 | -0.26 | -0.07 | -0.64 | -0.14 | -0.24 | -0.47 | 0.62 | 0.26 | 0.07 | 0.17 | -0.15 |
| Organic chemicals | '29 | 0.21 | -0.04 | -0.55 | 0.07 | 0.24 | -1.32 | -0.30 | -0.29 | -0.06 | -0.31 | 0.28 | -0.36 | -0.07 | 0.17 | -0.05 | 0.05 | -0.62 | -0.80 | 0.32 | 0.08 | -0.58 |
| Pharmaceutical products | '30 | -0.23 | -0.30 | -0.30 | 0.21 | -0.46 | -0.16 | -0.59 | -0.24 | -0.28 | -0.53 | 0.37 | -0.01 | 0.35 | 0.24 | -0.50 | -0.32 | -0.59 | -0.44 | 0.18 | -0.19 | -0.51 |
| Fertilisers | '31 | -0.16 | -0.13 | -0.56 | 0.53 | -0.26 | -1.54 | -1.01 | -0.11 | -0.14 | -0.53 | 0.01 | 0.10 | -0.41 | -0.19 | -0.18 | -0.90 | 1.20 | -0.34 | -0.25 | 0.03 | -0.58 |
| Tanning or dyeing extracts; paints and varnishes | '32 | -0.12 | -0.15 | -0.54 | 0.18 | -0.63 | -0.69 | -0.51 | -0.27 | -0.46 | -0.68 | 0.05 | 0.22 | 0.05 | 0.21 | -0.08 | -0.44 | -0.60 | -0.32 | 0.34 | 0.46 | -1.02 |
| Essential oils; perfumery, cosmetics | '33 | 0.50 | -0.47 | -0.22 | 0.00 | -0.81 | -0.37 | -0.80 | 0.12 | -0.57 | -0.28 | 0.26 | 0.21 | -0.33 | 0.37 | -0.41 | 0.08 | -0.02 | -0.04 | 0.10 | 0.18 | -0.32 |
| Soap, washing preparations, waxes, candles | '34 | 0.02 | 0.13 | -0.09 | 0.13 | -0.73 | -0.40 | -0.24 | 0.22 | -0.38 | -0.29 | 0.09 | 0.14 | -0.11 | -0.18 | -0.38 | 0.03 | -0.63 | 0.07 | 0.10 | 0.50 | -0.19 |
| Albuminoidal substances | '35 | -0.07 | -0.34 | -0.71 | 0.23 | -0.33 | -0.37 | -1.16 | -0.27 | 0.08 | -0.75 | -0.07 | -0.37 | -0.06 | 0.19 | -0.02 | 0.44 | -0.88 | -0.26 | -0.07 | 0.35 | -0.73 |
| Explosives; pyrotechnic products; matches | '36 | -0.17 | -0.41 | -0.17 | 0.16 | -0.99 | 0.82 | 0.37 | -0.04 | -0.38 | -1.06 | 0.26 | 0.51 | 0.06 | -0.06 | -0.30 | -0.74 | 0.08 | -0.28 | 0.07 | 0.11 | -0.16 |
| Photographic or cinematographic goods | '37 | -0.04 | 0.12 | -0.46 | 0.24 | -0.56 | -0.79 | -0.58 | -0.47 | -0.52 | -0.52 | -0.42 | -0.31 | -0.34 | -0.43 | -0.35 | -0.70 | -0.84 | -0.71 | -0.29 | 0.34 | -0.81 |
| Miscellaneous chemical products | '38 | -0.37 | -0.03 | -0.71 | 0.05 | -0.24 | -1.01 | -0.65 | -0.10 | -0.51 | -0.69 | 0.17 | 0.00 | 0.05 | -0.20 | -0.36 | -0.57 | -0.65 | -0.39 | 0.24 | 0.58 | -0.55 |
| Plastics and articles thereof | '39 | 0.45 | -0.06 | -0.49 | 0.16 | -0.66 | -0.47 | -0.40 | -0.08 | 0.02 | -0.37 | 0.24 | 0.06 | -0.03 | -0.17 | 0.41 | 0.17 | -0.26 | -0.22 | -0.05 | 0.25 | -0.59 |
| Rubber and articles thereof | '40 | 0.43 | 0.22 | -0.38 | -0.07 | -0.74 | -0.57 | -0.01 | 0.12 | 0.24 | 0.25 | 0.16 | 0.10 | -0.30 | -0.41 | 0.27 | 0.67 | -0.60 | 0.14 | -0.14 | -0.18 | 0.25 |
| Raw hides and skins (other than furskins) and leather | '41 | 0.09 | -0.35 | -0.52 | 0.23 | 0.07 | 0.84 | -0.92 | -0.19 | -0.38 | -0.55 | 0.09 | 0.19 | 0.07 | 0.09 | 0.29 | 0.07 | -0.70 | 0.07 | 0.43 | 0.63 | -0.47 |
| Articles of leather; saddlery; travel goods, handbags | '42 | -0.93 | -0.63 | -0.36 | -0.08 | -0.80 | 1.50 | 0.65 | -0.25 | -0.28 | -0.23 | -0.17 | -0.15 | -0.40 | -0.42 | -0.53 | -0.19 | 0.61 | 0.22 | -0.39 | -0.74 | 1.17 |
| Furskins and artificial fur; manufactures thereof | '43 | -1.15 | -0.18 | -0.07 | 0.03 | 0.25 | 0.02 | 0.00 | 0.41 | -0.26 | -0.45 | -0.08 | 0.33 | -0.27 | -0.57 | -0.11 | -0.36 | -0.30 | 0.39 | -0.26 | -0.18 | -0.80 |
| Wood and articles of wood | '44 | -0.85 | 0.58 | -0.39 | -0.43 | -0.41 | -0.28 | 0.20 | 0.48 | 0.05 | 0.46 | -0.18 | 0.12 | 0.24 | -0.58 | -0.59 | 0.67 | -0.61 | 0.41 | -0.88 | -0.18 | 0.26 |
| Cork and articles of cork | '45 | -0.13 | -0.07 | -0.79 | -0.14 | -0.31 | -0.11 | -0.43 | -0.28 | 0.07 | -0.93 | -0.04 | 0.14 | -0.12 | -0.55 | -0.10 | -0.11 | 1.11 | -0.31 | -0.54 | -0.79 | -0.15 |
| Manufactures of straw, basketware | '46 | -0.71 | -0.28 | 0.08 | -0.31 | -1.02 | 0.32 | 1.29 | -0.10 | -0.90 | -0.66 | -0.17 | -0.41 | -0.57 | -0.78 | -0.10 | 0.00 | -0.77 | -0.48 | -0.91 | -1.26 | 0.96 |
| Pulp of wood or of other fibrous cellulosic material | '47 | -0.98 | -0.34 | -0.84 | -0.04 | 0.71 | -1.09 | 0.37 | -0.45 | 0.98 | -0.30 | 0.23 | -0.05 | 0.78 | -0.11 | -0.64 | -0.65 | -1.10 | -0.33 | 0.11 | 0.67 | -0.91 |
| Paper and paperboard; articles of paper pulp, of paper or of paperboard | '48 | 0.12 | -0.38 | -0.64 | -0.07 | -0.38 | -0.66 | -0.83 | -0.02 | 0.33 | -0.40 | -0.06 | 0.08 | 0.65 | -0.40 | -0.10 | -0.10 | -0.27 | -0.10 | -0.27 | 0.14 | -0.40 |
| Printed books, newspapers, pictures and products of the printing industry | '49 | -0.09 | 0.14 | -0.28 | 0.23 | -0.79 | -0.32 | -0.58 | 0.42 | -0.31 | 0.11 | 0.25 | 0.19 | -0.22 | -0.45 | 0.15 | -0.31 | -0.26 | 0.01 | 0.33 | 0.17 | -0.14 |
| Silk | '50 | -0.30 | -0.37 | -0.07 | -0.25 | -0.24 | -1.28 | -0.41 | -0.45 | -1.06 | -0.01 | -0.17 | -0.26 | -0.01 | -0.20 | -0.21 | -0.15 | -0.69 | -1.00 | -0.01 | -0.42 | 0.18 |
| Wool, fine or coarse animal hair; horsehair yarn and woven fabric | '51 | -0.70 | -0.05 | -0.21 | -0.26 | -0.44 | -0.17 | -0.19 | -0.31 | -0.28 | -0.09 | -0.04 | 0.13 | -0.28 | -0.22 | -0.18 | -0.44 | -0.66 | -0.37 | 0.22 | -0.18 | -1.25 |
| Cotton | '52 | -0.49 | -0.37 | -0.35 | 0.02 | -0.09 | 0.23 | -0.70 | -0.54 | -0.54 | -0.77 | -0.03 | 0.22 | -0.09 | -0.05 | -0.05 | -0.33 | -0.94 | -0.15 | -0.03 | 1.00 | -0.15 |
| Other vegetable textile fibres; paper yarn and woven fabrics of paper yarn | '53 | -0.76 | 0.04 | -0.59 | -0.08 | -0.31 | -0.46 | 0.38 | -0.06 | -0.92 | -0.62 | -0.02 | -0.07 | -0.24 | -0.10 | -0.02 | 0.00 | 0.07 | -0.68 | -0.12 | -0.44 | -0.56 |
| Man-made filaments; strip and the like of man-made textile materials | '54 | 0.32 | -0.09 | -0.69 | 0.01 | -0.31 | -0.82 | -0.80 | -0.63 | -0.71 | -0.55 | 0.19 | -0.02 | -0.20 | -0.09 | 0.85 | -0.14 | -1.02 | -0.12 | -0.06 | 0.02 | -0.48 |
| Man-made staple fibres | '55 | 0.38 | 0.28 | -0.60 | -0.09 | -0.16 | 0.05 | -0.82 | -0.59 | 0.09 | -0.08 | -0.03 | 0.05 | -0.24 | -0.07 | 0.63 | 0.58 | -1.02 | -0.03 | -0.04 | 0.31 | -0.58 |
| Wadding, felt and nonwovens; special yarns; twine, cordage, ropes | '56 | -0.05 | -0.08 | -0.67 | 0.02 | -0.74 | -0.56 | -0.42 | -0.27 | 0.43 | -0.61 | -0.23 | 0.14 | -0.10 | -0.45 | 0.39 | 0.28 | -0.47 | 0.43 | -0.16 | 0.15 | -0.42 |
| Carpets and other textile floor coverings | '57 | -0.33 | -0.58 | -0.38 | 0.49 | -1.16 | 1.06 | -0.48 | -0.01 | 0.09 | -0.67 | -0.29 | -0.25 | -0.46 | -0.39 | -0.25 | 0.35 | -0.50 | 1.38 | -0.44 | -0.43 | 0.35 |
| Special woven fabrics; tufted textile fabrics; lace; tapestries | '58 | 0.44 | -0.38 | -0.73 | 0.02 | -0.46 | 0.07 | -0.54 | -0.73 | 0.39 | -0.33 | -0.08 | 0.11 | -0.18 | -0.02 | 1.11 | 0.03 | -0.67 | 0.27 | -0.16 | -0.09 | -0.95 |
| Impregnated, coated, covered or laminated textile fabrics | '59 | 0.41 | -0.40 | -0.64 | 0.14 | -0.44 | -0.76 | -0.96 | -0.20 | 0.33 | -0.46 | -0.15 | 0.06 | -0.05 | 0.16 | 0.61 | -0.64 | -1.30 | -0.06 | 0.08 | 0.03 | -0.32 |
| Knitted or crocheted fabrics | '60 | 1.13 | 0.16 | -0.86 | -0.13 | -0.22 | -0.14 | -1.21 | -0.32 | 0.04 | -0.86 | -0.05 | 0.22 | 0.11 | -0.11 | 1.41 | -0.21 | -1.36 | 0.55 | -0.08 | 0.02 | -0.47 |
| Articles of apparel and clothing accessories, knitted or crocheted | '61 | -0.61 | -0.05 | -0.14 | -0.22 | -0.95 | 1.68 | 0.36 | -0.20 | 0.33 | -0.11 | -0.20 | -0.18 | -0.48 | -0.61 | -0.35 | 0.35 | 0.80 | 1.24 | -0.46 | -1.01 | 1.49 |
| Articles of apparel and clothing accessories, not knitted or crocheted | '62 | -0.83 | -0.23 | 0.03 | -0.25 | -1.05 | 1.68 | 0.17 | -0.15 | -0.03 | 0.36 | -0.20 | -0.07 | -0.41 | -0.57 | -0.37 | -0.01 | 0.74 | 0.95 | -0.35 | -0.97 | 1.48 |
| Other made-up textile articles; sets; worn clothing | '63 | -0.30 | -0.43 | 0.14 | -0.26 | -0.93 | 0.72 | -0.22 | 0.02 | 0.62 | 0.22 | -0.16 | -0.24 | -0.42 | -0.66 | 0.00 | 0.03 | 0.39 | 1.20 | -0.30 | -0.79 | 1.01 |
| Footwear, gaiters and the like | '64 | -0.71 | -0.41 | -0.28 | -0.17 | -0.95 | 0.58 | -0.34 | -0.14 | 0.42 | 0.18 | -0.14 | -0.03 | -0.43 | -0.47 | -0.37 | -0.03 | 0.57 | 0.33 | -0.34 | -0.96 | 1.09 |
| Headgear and parts thereof | '65 | -0.23 | 0.15 | -0.02 | 0.06 | -1.02 | -0.03 | 0.03 | -0.05 | 0.24 | -0.24 | -0.33 | -0.23 | -0.34 | -0.80 | 0.26 | 0.11 | 0.12 | 0.22 | -0.21 | -0.78 | 1.09 |
| Umbrellas, sun umbrellas, walking sticks | '66 | -1.10 | -0.30 | -0.60 | -0.16 | -0.82 | -0.28 | -0.67 | -0.15 | -0.34 | -0.66 | -0.22 | -0.50 | -0.56 | -0.37 | -0.13 | -0.59 | 0.29 | -0.10 | -0.61 | -1.03 | 0.23 |
| Prepared feathers and down; artificial flowers; articles of human hair | '67 | -0.49 | -0.37 | -0.47 | -0.33 | -0.90 | -0.25 | 0.53 | -0.94 | -0.77 | -0.60 | -0.13 | -0.61 | -0.64 | -0.50 | -0.05 | -0.13 | -0.28 | -0.46 | -0.85 | -1.25 | -0.20 |
| Articles of stone, plaster, cement, mica or similar materials | '68 | -0.38 | 0.24 | 0.01 | -0.17 | -0.93 | -0.08 | -0.31 | 0.23 | 0.45 | -0.52 | -0.64 | 0.47 | -0.47 | -0.48 | -0.37 | 0.17 | -0.23 | 0.67 | -0.16 | -0.20 | -0.06 |
| Ceramic products | '69 | -0.77 | -0.35 | 0.05 | -0.28 | -0.93 | -0.78 | -1.17 | 0.09 | 0.56 | -0.43 | -0.31 | 0.76 | -0.57 | -0.69 | -0.71 | 0.01 | -0.07 | 0.62 | -0.49 | -0.47 | -0.04 |
| Glass and glassware | '70 | -0.18 | 0.06 | 0.03 | -0.17 | -0.72 | -0.33 | -0.38 | 0.18 | 0.19 | -0.38 | -0.49 | 0.01 | -0.39 | -0.40 | 0.00 | -0.10 | -0.10 | 0.31 | -0.12 | 0.03 | -0.23 |
| Natural or cultured pearls, precious stones, imitation jewellery, coin | '71 | -0.11 | -0.24 | 0.49 | -0.01 | 0.13 | 0.00 | 0.63 | 0.08 | 0.09 | 0.18 | -0.06 | 0.06 | 0.25 | -0.05 | -0.23 | 0.10 | -0.04 | -0.21 | -0.14 | 0.23 | 0.26 |
| Iron and steel | '72 | 0.14 | -0.28 | -0.59 | 0.06 | 0.10 | -1.24 | -1.05 | -0.31 | -0.16 | -0.05 | -0.18 | -0.03 | 0.17 | -0.30 | -0.03 | -1.01 | -0.52 | -0.07 | 0.23 | -0.03 | -0.46 |
| Articles of iron or steel | '73 | 0.07 | -0.21 | -0.20 | -0.05 | -0.79 | -0.64 | -0.45 | 0.18 | 0.23 | -0.02 | -0.22 | 0.30 | -0.12 | -0.22 | 0.64 | -0.23 | 0.09 | 0.55 | -0.15 | -0.18 | -0.12 |
| Copper and articles thereof | '74 | -0.11 | -0.15 | -0.17 | 0.02 | -0.12 | 0.76 | 0.53 | 0.35 | -0.29 | -0.45 | -0.01 | 0.29 | 0.14 | -0.18 | -0.12 | -0.31 | -0.61 | -0.22 | 0.12 | 0.09 | -0.45 |
| Nickel and articles thereof | '75 | -0.80 | -0.08 | -0.19 | 0.14 | -0.05 | -0.36 | 1.28 | -0.56 | -0.18 | -0.44 | -0.12 | -0.66 | -0.39 | -0.10 | -0.72 | -0.34 | -0.52 | -0.63 | 0.17 | 0.29 | -0.31 |
| Aluminium and articles thereof | '76 | -0.35 | -0.08 | -0.72 | -0.12 | 0.47 | -0.50 | -0.24 | -0.11 | 0.04 | 0.14 | -0.21 | 0.08 | -0.04 | -0.24 | -0.23 | -0.33 | -0.27 | 0.02 | -0.11 | -0.14 | -0.53 |
| Lead and articles thereof | '78 | 0.37 | 0.33 | 0.69 | 0.14 | -0.75 | -0.08 | -0.48 | -0.04 | -0.28 | 0.04 | 0.18 | -0.85 | 1.01 | -0.11 | -0.61 | -1.07 | 0.34 | -1.04 | 0.25 | -0.63 | -0.74 |
| Zinc and articles thereof | '79 | 0.99 | -0.47 | 0.55 | 0.00 | 1.45 | -0.67 | -0.43 | -0.05 | -0.77 | -0.92 | -0.27 | 1.23 | 0.11 | -0.19 | -0.78 | -0.94 | -0.53 | -1.45 | -0.20 | -0.61 | -1.20 |
| Tin and articles thereof | '80 | -0.89 | 0.27 | -0.77 | -0.12 | -0.13 | -0.61 | -0.07 | 0.08 | -0.13 | -0.58 | -0.12 | -0.05 | -0.08 | -0.15 | -0.16 | 0.26 | 0.04 | -1.06 | -0.56 | -0.51 | -0.84 |
| Other base metals; cermets; articles thereof | '81 | -0.70 | -0.43 | -0.48 | -0.02 | 0.36 | -0.29 | -0.42 | -0.40 | -0.43 | -0.38 | -0.15 | -0.39 | -0.61 | -0.28 | -0.52 | -0.16 | -0.37 | -0.61 | 0.13 | 0.25 | -0.23 |
| Tools, implements, cutlery, spoons and forks, of base metal | '82 | 0.35 | -0.44 | -0.38 | -0.12 | -0.73 | 0.52 | -0.63 | 0.01 | -0.04 | -0.61 | 0.00 | -0.01 | 0.24 | 0.20 | 0.70 | -0.44 | -0.67 | -0.28 | -0.15 | -0.22 | 0.01 |
| Miscellaneous articles of base metal | '83 | 0.28 | -0.23 | -0.05 | -0.20 | -0.67 | -0.49 | -0.12 | 0.01 | 0.02 | -0.11 | -0.06 | 0.02 | -0.21 | -0.34 | 0.69 | 0.06 | -0.49 | 0.25 | -0.21 | -0.25 | -0.37 |
| Machinery, mechanical appliances, nuclear reactors, boilers | '84 | 0.10 | -0.06 | 0.01 | 0.07 | -0.48 | -0.68 | 0.14 | 0.05 | -0.21 | -0.01 | -0.02 | -0.10 | 0.07 | 0.01 | -0.08 | 0.15 | -0.39 | -0.05 | 0.12 | -0.08 | -0.27 |
| Electrical machinery and equipment; sound recorders and reproducers, tv | '85 | 0.23 | 0.11 | -0.08 | -0.09 | -0.44 | -0.68 | 0.31 | -0.05 | -0.05 | 0.11 | 0.09 | -0.12 | -0.14 | -0.09 | 0.21 | -0.10 | 0.31 | -0.13 | -0.16 | -0.11 | 0.11 |
| Railway or tramway locomotives; railway or tramway track | '86 | -0.04 | -0.35 | 0.39 | -0.52 | -0.87 | -0.86 | -0.46 | 0.21 | -0.15 | 0.17 | -0.48 | 0.77 | -0.44 | 0.01 | -0.40 | -0.81 | -1.34 | -0.16 | -0.84 | 0.33 | -0.13 |
| Vehicles other than railway or tramway rolling stock | '87 | 0.57 | -0.36 | 0.44 | -0.14 | -0.96 | -0.47 | -0.66 | 0.06 | 0.07 | 0.25 | -0.11 | 0.15 | 0.08 | -0.64 | -0.02 | 0.48 | -0.23 | 0.56 | -0.02 | -0.20 | -0.23 |
| Aircraft, spacecraft, and parts thereof | '88 | -0.34 | -0.44 | 0.11 | -0.32 | -0.62 | -0.01 | -0.58 | -0.35 | -0.73 | 0.11 | -0.38 | 0.21 | -0.29 | -0.32 | -0.70 | -0.50 | 0.35 | -0.64 | 0.25 | 0.60 | -0.11 |
| Ships, boats and floating structures | '89 | 1.03 | -0.02 | 0.40 | 0.11 | -0.22 | -0.60 | 0.68 | -0.01 | 0.11 | 0.86 | 0.24 | -0.12 | -0.19 | -0.34 | -0.05 | 0.15 | 0.27 | -0.05 | 0.23 | 0.06 | 0.23 |
| Optical, photographic, cinematographic, medical or surgical instruments | '90 | 0.00 | 0.19 | 0.07 | 0.08 | -0.30 | 0.16 | 0.18 | -0.08 | 0.10 | 0.18 | 0.22 | -0.36 | -0.04 | 0.28 | 0.03 | -0.10 | 0.33 | -0.51 | 0.20 | 0.18 | -0.28 |
| Clocks and watches and parts thereof | '91 | -0.76 | -0.46 | -0.30 | -0.23 | -0.48 | -0.18 | 0.24 | -0.29 | -0.11 | -0.25 | -0.13 | -0.26 | -0.23 | 0.25 | -0.53 | 0.09 | 0.44 | -0.47 | -0.14 | -0.34 | -0.23 |
| Musical instruments; parts and accessories of such articles | '92 | -0.31 | 0.56 | 0.13 | 0.13 | -1.05 | 0.51 | -0.33 | -0.25 | -0.51 | -0.14 | -0.34 | -0.46 | -0.06 | -0.59 | 0.54 | -0.28 | 0.24 | -0.28 | -0.19 | -0.08 | -0.01 |
| Arms and ammunition; parts and accessories thereof | '93 | -0.26 | -0.22 | 0.01 | -0.60 | 0.01 | 0.80 | -0.81 | -0.38 | 0.12 | 0.82 | -0.02 | 0.54 | 0.13 | 0.06 | 0.43 | -0.08 | -0.96 | 0.21 | 0.18 | 0.39 | 0.00 |
| Furniture; bedding, mattresses, stuffed furnishings; lamps | '94 | -0.33 | 0.36 | 0.43 | -0.30 | -0.94 | 0.13 | -0.08 | 0.49 | 0.29 | 0.42 | -0.32 | -0.05 | -0.17 | -0.83 | 0.19 | -0.03 | 0.38 | 0.74 | -0.37 | -0.73 | 0.99 |
| Toys, games and sports requisites | '95 | -0.60 | -0.12 | -0.25 | -0.09 | -0.85 | 0.95 | -0.27 | -0.11 | -0.60 | -0.44 | -0.12 | -0.14 | -0.35 | -0.60 | 0.44 | 0.24 | 0.41 | -0.12 | -0.31 | -0.61 | 0.74 |
| Miscellaneous manufactured articles | '96 | -0.32 | -0.11 | 0.07 | 0.05 | -0.97 | -0.60 | -0.44 | 0.21 | -0.35 | -0.55 | -0.14 | -0.13 | 0.15 | -0.15 | -0.04 | 0.14 | 0.31 | 0.45 | -0.46 | -0.32 | -0.38 |
| Works of art, collectors' pieces and antiques | '97 | -0.46 | -0.19 | -0.10 | -0.14 | -0.59 | -0.36 | 0.03 | -0.10 | -0.11 | 0.02 | -0.14 | 0.05 | -0.16 | -0.21 | -0.31 | -0.22 | 0.00 | 0.02 | 0.38 | 0.11 | 0.01 |
| Commodities not elsewhere specified | '99 | 0.00 | -0.26 | -0.71 | 0.21 | 0.49 | -0.27 | -0.63 | -0.86 | 0.06 | 0.00 | 0.48 | -0.09 | -0.35 | 0.00 | -0.57 | 0.00 | 0.00 | 0.01 | -0.07 | -0.47 | -0.19 |
| Parts and accessories for calculating machines | 8473 | 0.21 | -0.07 | -0.96 | -0.09 | -0.45 | -0.07 | 0.01 | -0.62 | -0.12 | 0.11 | -0.17 | -0.32 | -0.24 | -0.22 | 0.30 | 0.03 | -0.29 | -0.13 | -0.15 | 0.14 | -0.14 |
| Electronic integrated circuits; parts thereof | 8542 | 0.67 | 0.06 | -1.01 | -0.28 | -1.52 | -0.11 | 1.74 | -1.07 | -0.50 | -0.33 | 1.52 | -1.52 | -0.13 | 0.02 | 1.68 | -0.77 | -1.60 | -0.08 | 0.12 | 0.76 | -0.41 |
| Parts and accessories for tractors, motor vehicles | 8708 | 0.69 | -0.42 | 0.03 | -0.25 | -0.22 | -0.22 | 0.39 | 0.21 | 0.11 | 0.27 | -0.01 | -0.14 | -0.12 | -0.04 | 0.29 | 0.07 | 0.40 | 0.05 | -0.26 | -0.02 | -0.21 |
| Parts of aircraft and spacecraft | 8803 | 0.36 | -0.10 | 0.30 | 0.16 | 0.07 | 0.25 | -0.02 | 0.33 | 0.17 | 0.52 | -0.05 | 0.31 | 0.24 | -0.22 | -0.67 | 0.04 | 0.46 | 0.10 | 0.54 | -0.31 | 0.01 |
| Parts of telephone sets, telephones for cellular networks | 851770 | 0.25 | -0.13 | -0.16 | -0.20 | -0.09 | -0.35 | -0.43 | 0.00 | -0.01 | 0.26 | -0.02 | -0.19 | 0.08 | -0.15 | 0.07 | 0.03 | -0.69 | -0.18 | -0.13 | -0.07 | 0.04 |

**Table S 3. Comparative advantages Balassa symmetric. 2001**

| **Product label** | **Code** | **Bang** | **Belg** | **Brazil** | **Bulg** | **Camb** | **Chile** | **Chin** | **Col** | **Czech** | **Eth** | **Fin** | **Fran** | **Germ** | **Gre** | **Hon** | **Hung** | **India** | **Indo** | **Irel** | **Italy** | **Jap** |
| --- | --- | --- | --- | --- | --- | --- | --- | --- | --- | --- | --- | --- | --- | --- | --- | --- | --- | --- | --- | --- | --- | --- |
| Dairy products | '04 | -1.00 | 0.33 | -0.71 | 0.06 | -0.25 | -0.13 | -0.75 | 0.19 | 0.09 | -0.94 | 0.07 | 0.43 | 0.23 | 0.43 | -0.75 | -0.07 | -0.47 | -0.28 | 0.40 | -0.11 | -0.99 |
| Products of animal origin | '05 | -0.10 | -0.26 | 0.48 | -0.11 | -0.97 | 0.03 | 0.62 | -0.23 | -0.45 | 0.78 | -0.79 | -0.18 | -0.04 | -0.33 | -0.43 | 0.59 | 0.29 | -0.67 | -0.52 | -0.46 | -0.86 |
| Lac; gums, resins | '13 | -0.98 | -0.44 | 0.27 | -0.63 | -0.99 | 0.84 | -0.18 | -0.94 | -0.09 | 0.96 | -0.86 | 0.35 | 0.07 | 0.27 | -0.65 | -0.90 | 0.88 | 0.09 | 0.29 | -0.01 | -0.76 |
| Vegetable plaiting materials | '14 | 0.38 | -0.55 | 0.37 | -0.71 | 0.17 | 0.62 | 0.32 | -0.40 | -0.94 | 1.00 | -0.99 | -0.53 | -0.28 | -0.02 | 0.05 | 0.53 | 0.70 | 0.74 | -0.61 | -0.88 | -0.93 |
| Animal or vegetable fats and oils | '15 | -0.97 | 0.11 | 0.58 | -0.09 | -0.84 | -0.52 | -0.76 | 0.34 | -0.42 | 0.06 | -0.65 | -0.37 | -0.25 | 0.78 | -0.66 | -0.27 | 0.21 | 0.78 | -0.73 | 0.07 | -0.90 |
| Preparations of meat, fish, molluscs | '16 | -0.96 | 0.05 | 0.44 | -0.31 | -1.00 | 0.70 | 0.49 | -0.07 | -0.58 | -1.00 | -0.83 | -0.13 | -0.27 | -0.09 | -0.87 | 0.12 | -0.92 | -0.16 | 0.22 | -0.32 | -0.65 |
| Sugars and sugar confectionery | '17 | -0.96 | 0.24 | 0.89 | -0.12 | -0.99 | -0.32 | -0.65 | 0.84 | 0.00 | 0.55 | -0.32 | 0.34 | -0.20 | -0.04 | -0.66 | -0.43 | 0.53 | -0.42 | -0.38 | -0.49 | -0.91 |
| Cocoa and cocoa preparations | '18 | -0.95 | 0.48 | 0.26 | -0.20 | -1.00 | 0.05 | -0.90 | 0.14 | -0.16 | -1.00 | -0.36 | 0.19 | 0.03 | -0.17 | -0.84 | -0.30 | -0.91 | 0.55 | 0.10 | -0.20 | -0.95 |
| Preparations of cereals, flour | '19 | -0.88 | 0.43 | -0.45 | 0.16 | -0.97 | -0.13 | -0.31 | 0.24 | -0.26 | -0.44 | -0.54 | 0.25 | 0.06 | 0.40 | -0.70 | -0.40 | -0.43 | -0.34 | 0.55 | 0.45 | -0.76 |
| Preparations of vegetables, fruit, nuts | '20 | -0.99 | 0.32 | 0.69 | 0.28 | -0.96 | 0.66 | 0.25 | -0.34 | -0.52 | -0.99 | -0.84 | -0.04 | -0.21 | 0.87 | -0.85 | 0.48 | -0.30 | -0.18 | -0.65 | 0.32 | -0.95 |
| Miscellaneous edible preparations | '21 | -0.90 | 0.09 | 0.52 | -0.31 | -0.97 | 0.58 | -0.33 | 0.59 | -0.06 | -1.00 | -0.61 | 0.19 | -0.01 | 0.19 | -0.50 | -0.19 | 0.09 | -0.66 | 0.50 | -0.06 | -0.51 |
| Beverages, spirits and vinegar | '22 | -0.99 | -0.05 | -0.36 | 0.41 | -0.99 | 0.74 | -0.49 | -0.55 | -0.18 | -0.69 | -0.57 | 0.63 | -0.29 | 0.34 | -0.58 | -0.30 | -0.77 | -0.87 | 0.20 | 0.36 | -0.89 |
| Residues and waste from food industries | '23 | -1.00 | 0.19 | 0.85 | 0.13 | -0.97 | 0.71 | -0.52 | -0.43 | -0.39 | -0.61 | -0.78 | 0.06 | -0.16 | -0.09 | -0.95 | 0.13 | 0.52 | -0.42 | -0.41 | -0.61 | -0.90 |
| Tobacco and manufactured tobacco substitutes | '24 | -0.53 | -0.17 | 0.68 | 0.56 | -0.29 | -0.52 | -0.42 | -0.05 | -0.17 | -1.00 | -0.94 | -0.44 | 0.02 | 0.82 | -0.13 | -0.73 | 0.11 | 0.16 | -0.47 | -0.62 | -0.71 |
| Salt; sulphur; earths and stone; lime and cement | '25 | -0.99 | 0.15 | 0.30 | 0.63 | -1.00 | 0.11 | 0.29 | 0.52 | 0.07 | -0.29 | -0.25 | -0.09 | -0.18 | 0.81 | -0.79 | -0.48 | 0.61 | 0.29 | -0.73 | -0.17 | -0.59 |
| Ores, slag and ash | '26 | -0.99 | -0.53 | 0.87 | 0.21 | -1.00 | 0.95 | -0.85 | -0.89 | -0.93 | 0.50 | -0.76 | -0.84 | -0.89 | -0.12 | -0.92 | -0.94 | 0.51 | 0.77 | -0.53 | -0.90 | -0.97 |
| Mineral fuels, mineral oils and products of their distillation | '27 | -0.97 | -0.40 | -0.43 | -0.02 | -1.00 | -0.71 | -0.53 | 0.61 | -0.56 | -1.00 | -0.54 | -0.60 | -0.75 | 0.08 | -0.96 | -0.68 | -0.31 | 0.42 | -0.94 | -0.69 | -0.93 |
| Inorganic chemicals | '28 | -0.34 | 0.13 | 0.20 | 0.04 | -1.00 | 0.56 | 0.22 | -0.31 | -0.24 | -1.00 | 0.01 | 0.14 | -0.01 | -0.10 | -0.66 | -0.13 | -0.07 | -0.32 | -0.04 | -0.28 | -0.21 |
| Organic chemicals | '29 | -1.00 | 0.37 | -0.17 | -0.12 | -0.99 | 0.00 | -0.18 | -0.52 | -0.24 | -1.00 | -0.61 | 0.11 | 0.00 | -0.78 | -0.66 | -0.29 | 0.24 | -0.16 | 0.78 | -0.20 | 0.04 |
| Pharmaceutical products | '30 | -0.93 | 0.41 | -0.61 | -0.11 | -1.00 | -0.76 | -0.75 | 0.09 | -0.50 | -0.98 | -0.50 | 0.36 | 0.20 | 0.25 | -0.74 | -0.17 | 0.14 | -0.87 | 0.64 | 0.14 | -0.60 |
| Fertilisers | '31 | 0.49 | 0.24 | -0.38 | 0.76 | -1.00 | 0.53 | -0.27 | -0.53 | -0.27 | -0.98 | -0.11 | -0.64 | -0.21 | 0.14 | -0.99 | -0.59 | -0.68 | -0.05 | -0.65 | -0.78 | -0.85 |
| Tanning or dyeing extracts; paints and varnishes | '32 | -1.00 | 0.27 | -0.20 | -0.68 | -1.00 | -0.83 | -0.12 | 0.66 | -0.15 | -1.00 | 0.24 | 0.05 | 0.29 | -0.04 | -0.07 | -0.55 | 0.37 | -0.54 | -0.83 | -0.03 | -0.09 |
| Essential oils; perfumery, cosmeticse | '33 | -0.98 | -0.10 | -0.26 | 0.48 | -0.99 | -0.10 | -0.58 | 0.12 | -0.45 | -0.91 | -0.64 | 0.62 | 0.03 | 0.31 | -0.48 | -0.50 | -0.03 | -0.42 | 0.71 | 0.06 | -0.55 |
| Soap, washing preparations, waxes, candles | '34 | -0.99 | 0.37 | -0.34 | -0.41 | -1.00 | -0.72 | -0.34 | 0.21 | 0.34 | -0.68 | -0.38 | 0.16 | 0.22 | 0.29 | -0.34 | 0.12 | -0.47 | 0.05 | -0.61 | 0.13 | -0.26 |
| Albuminoidal substances | '35 | -0.99 | 0.14 | 0.14 | -0.20 | -0.99 | -0.57 | -0.48 | 0.10 | -0.48 | -1.00 | 0.30 | 0.23 | 0.09 | -0.41 | -0.43 | -0.20 | -0.11 | -0.82 | 0.43 | -0.17 | -0.31 |
| Explosives; pyrotechnic products; matches | '36 | -0.96 | -0.35 | -0.01 | 0.49 | -0.99 | 0.56 | 0.62 | -0.55 | 0.54 | -1.00 | -0.22 | 0.05 | -0.21 | -0.75 | -0.60 | 0.05 | 0.13 | -0.20 | -0.13 | -0.56 | -0.83 |
| Photographic or cinematographic goods | '37 | -1.00 | 0.45 | 0.04 | 0.24 | -0.98 | -0.93 | -0.28 | -0.92 | -0.78 | -0.98 | -0.72 | 0.10 | 0.02 | 0.21 | -0.07 | -0.75 | -0.59 | -0.93 | -0.81 | -0.31 | 0.53 |
| Miscellaneous chemical products | '38 | -0.96 | 0.24 | -0.20 | -0.74 | -1.00 | -0.65 | -0.32 | 0.36 | -0.45 | -1.00 | -0.22 | 0.25 | 0.22 | -0.03 | -0.39 | -0.25 | 0.08 | -0.30 | 0.40 | -0.07 | 0.06 |
| Plastics and articles thereof | '39 | -0.92 | 0.38 | -0.32 | -0.22 | -0.99 | -0.51 | -0.12 | 0.08 | 0.01 | -1.00 | -0.16 | 0.03 | 0.16 | 0.04 | 0.17 | -0.01 | -0.23 | -0.27 | -0.55 | 0.08 | -0.15 |
| Rubber and articles thereof | '40 | -1.00 | -0.02 | 0.21 | -0.04 | 0.29 | -0.22 | -0.20 | -0.11 | 0.40 | -1.00 | -0.29 | 0.21 | 0.04 | -0.35 | -0.61 | 0.05 | 0.01 | 0.41 | -0.75 | 0.03 | 0.19 |
| Raw hides and skins (other than furskins) and leather | '41 | 0.84 | -0.62 | 0.63 | 0.18 | -0.87 | -0.36 | -0.07 | 0.40 | -0.45 | 0.98 | -0.67 | -0.24 | -0.37 | 0.16 | 0.48 | -0.23 | 0.50 | -0.43 | -0.38 | 0.61 | -0.79 |
| Articles of leather; saddlery; travel goods, handbags | '42 | -0.65 | -0.15 | -0.51 | 0.12 | -0.64 | -0.97 | 0.72 | 0.16 | -0.36 | -0.96 | -0.79 | 0.21 | -0.56 | -0.60 | 0.72 | -0.17 | 0.69 | 0.15 | -0.84 | 0.38 | -0.95 |
| Furskins and artificial fur; manufactures thereof | '43 | -1.00 | -0.76 | -0.67 | -0.11 | -1.00 | -0.77 | 0.46 | -0.95 | -0.18 | 0.16 | 0.82 | -0.55 | -0.35 | 0.94 | 0.69 | -0.28 | -0.98 | -0.98 | -0.93 | 0.11 | -0.98 |
| Wood and articles of wood | '44 | -0.73 | -0.09 | 0.46 | 0.26 | 0.16 | 0.74 | -0.11 | -0.59 | 0.29 | -0.98 | 0.64 | -0.25 | -0.23 | -0.55 | -0.33 | 0.06 | -0.87 | 0.69 | -0.61 | -0.35 | -0.97 |
| Cork and articles of cork | '45 | -1.00 | -0.89 | -0.89 | 0.37 | -0.99 | -0.66 | -0.77 | -0.72 | -0.90 | -1.00 | -0.98 | -0.02 | -0.58 | -0.91 | -0.70 | -0.92 | -0.89 | -0.69 | -1.00 | 0.02 | -0.98 |
| Manufactures of straw, basketware | '46 | 0.40 | -0.16 | -0.98 | -0.97 | -0.74 | -0.99 | 0.84 | -0.87 | -0.83 | 0.62 | -0.95 | -0.66 | -0.76 | -0.83 | 0.45 | -0.49 | -0.66 | 0.75 | -0.93 | -0.83 | -0.96 |
| Pulp of wood or of other fibrous cellulosic material | '47 | -1.00 | -0.13 | 0.77 | 0.05 | -1.00 | 0.91 | -0.98 | -0.96 | 0.08 | -1.00 | 0.70 | -0.50 | -0.55 | -0.77 | -0.81 | -0.82 | -0.97 | 0.52 | -0.92 | -0.91 | -0.80 |
| Paper and paperboard; articles of paper pulp, of paper or of paperboard | '48 | -0.99 | 0.05 | 0.04 | -0.34 | -0.99 | 0.06 | -0.50 | 0.16 | 0.01 | -0.97 | 0.83 | 0.07 | 0.12 | -0.29 | -0.25 | -0.12 | -0.57 | 0.36 | -0.75 | -0.02 | -0.54 |
| Printed books, newspapers, pictures and products of the printing industry | '49 | -0.83 | 0.08 | -0.77 | -0.77 | 0.94 | 0.06 | -0.40 | 0.53 | 0.38 | -0.96 | 0.25 | 0.10 | 0.16 | -0.01 | 0.10 | -0.45 | -0.50 | -0.82 | -0.27 | 0.10 | -0.67 |
| Silk | '50 | 0.78 | -0.91 | 0.36 | -0.77 | -0.76 | -0.72 | 0.78 | -0.87 | -0.98 | -1.00 | -0.97 | -0.22 | -0.36 | -0.93 | 0.40 | -0.97 | 0.89 | -0.97 | -0.97 | 0.54 | -0.19 |
| Wool, fine or coarse animal hair; horsehair yarn and woven fabric | '51 | -0.94 | -0.45 | -0.61 | 0.34 | -1.00 | -0.13 | 0.35 | -0.60 | 0.37 | -1.00 | -0.77 | -0.13 | -0.08 | -0.16 | 0.20 | -0.43 | -0.24 | -0.89 | -0.61 | 0.69 | -0.30 |
| Cotton | '52 | -0.10 | -0.15 | 0.13 | -0.01 | -0.98 | -0.49 | 0.38 | -0.29 | 0.00 | 0.80 | -0.86 | -0.24 | -0.43 | 0.75 | 0.55 | -0.37 | 0.79 | 0.36 | -0.98 | 0.24 | -0.45 |
| Other vegetable textile fibres; paper yarn and woven fabrics of paper yarn | '53 | 0.97 | 0.50 | -0.17 | -0.54 | -1.00 | -0.59 | 0.57 | -0.97 | 0.37 | -1.00 | -0.82 | 0.35 | -0.52 | -0.44 | 0.57 | 0.14 | 0.78 | -0.92 | -0.46 | 0.41 | -0.79 |
| Man-made filaments; strip and the like of man-made textile materials | '54 | -0.30 | -0.10 | -0.63 | 0.00 | -0.63 | -0.68 | 0.11 | -0.29 | -0.04 | -1.00 | -0.77 | -0.16 | -0.17 | -0.21 | 0.30 | -0.25 | 0.46 | 0.61 | -0.42 | 0.21 | 0.07 |
| Man-made staple fibres | '55 | -0.73 | -0.04 | -0.61 | 0.20 | -0.14 | -0.49 | 0.45 | -0.23 | 0.02 | -0.73 | -0.37 | -0.08 | -0.04 | -0.36 | 0.25 | -0.54 | 0.50 | 0.61 | -0.37 | 0.20 | -0.06 |
| Wadding, felt and nonwovens; special yarns; twine, cordage, ropes | '56 | 0.60 | -0.09 | -0.07 | -0.39 | -0.92 | -0.49 | -0.12 | 0.32 | 0.30 | -1.00 | 0.13 | 0.01 | 0.31 | 0.22 | -0.16 | -0.10 | -0.16 | -0.36 | -0.85 | 0.39 | -0.03 |
| Carpets and other textile floor coverings | '57 | -0.84 | 0.77 | -0.52 | 0.04 | -1.00 | -0.89 | 0.15 | -0.62 | -0.09 | -0.98 | -0.80 | -0.23 | -0.31 | 0.38 | -0.72 | -0.56 | 0.83 | -0.48 | -0.44 | -0.51 | -0.95 |
| Special woven fabrics; tufted textile fabrics; lace; tapestries | '58 | 0.54 | 0.21 | -0.54 | -0.40 | -0.96 | -0.74 | 0.39 | -0.03 | 0.31 | -0.99 | -0.66 | 0.11 | -0.17 | -0.01 | 0.42 | -0.43 | 0.60 | -0.01 | -0.81 | 0.05 | -0.34 |
| Impregnated, coated, covered or laminated textile fabrics | '59 | -1.00 | 0.06 | -0.51 | -0.86 | -0.99 | -0.67 | -0.11 | 0.22 | 0.20 | -1.00 | -0.04 | -0.11 | 0.09 | -0.56 | 0.14 | -0.42 | -0.32 | -0.06 | -0.81 | 0.21 | -0.25 |
| Knitted or crocheted fabrics | '60 | 0.16 | -0.53 | -0.64 | -0.45 | -0.24 | -0.79 | 0.34 | 0.19 | -0.37 | 0.06 | -0.80 | -0.11 | -0.22 | 0.39 | 0.63 | -0.72 | -0.50 | -0.46 | -0.99 | 0.16 | -0.46 |
| Articles of apparel and clothing accessories, knitted or crocheted | '61 | 0.89 | -0.25 | -0.61 | 0.70 | 0.96 | -0.85 | 0.57 | 0.19 | -0.44 | -0.68 | -0.80 | -0.30 | -0.52 | 0.76 | 0.62 | 0.10 | 0.53 | 0.34 | -0.81 | 0.22 | -0.95 |
| Articles of apparel and clothing accessories, not knitted or crocheted | '62 | 0.94 | -0.17 | -0.78 | 0.74 | 0.21 | -0.84 | 0.61 | 0.31 | -0.19 | -0.77 | -0.72 | -0.25 | -0.36 | 0.02 | 0.51 | 0.18 | 0.65 | 0.48 | -0.76 | 0.30 | -0.94 |
| Other made-up textile articles; sets; worn clothing | '63 | 0.85 | -0.05 | 0.24 | 0.42 | 0.46 | -0.71 | 0.63 | 0.24 | 0.43 | -0.80 | -0.65 | -0.35 | -0.34 | 0.42 | -0.09 | -0.05 | 0.79 | 0.16 | -0.71 | -0.18 | -0.83 |
| Footwear, gaiters and the like | '64 | 0.00 | 0.05 | 0.61 | 0.62 | 0.40 | -0.82 | 0.65 | -0.35 | -0.22 | -0.84 | -0.68 | -0.41 | -0.53 | -0.42 | 0.58 | 0.18 | 0.34 | 0.54 | -0.91 | 0.59 | -0.97 |
| Headgear and parts thereof | '65 | 0.87 | -0.02 | -0.82 | -0.29 | 0.89 | -0.94 | 0.65 | -0.28 | 0.26 | -1.00 | -0.65 | -0.38 | -0.49 | -0.58 | 0.61 | -0.18 | -0.58 | 0.08 | -0.94 | 0.37 | -0.34 |
| Umbrellas, sun umbrellas, walking sticks | '66 | -0.99 | -0.39 | -0.98 | -0.40 | -0.99 | -0.96 | 0.82 | -0.95 | -0.09 | -1.00 | -0.94 | -0.59 | -0.58 | -0.19 | 0.79 | -0.69 | -0.86 | 0.12 | -0.98 | -0.06 | -0.95 |
| Prepared feathers and down; artificial flowers; articles of human hair | '67 | -0.77 | -0.46 | -0.99 | -0.96 | -0.99 | -1.00 | 0.79 | -0.94 | -0.78 | -1.00 | -0.96 | -0.80 | -0.73 | -0.88 | 0.85 | -0.82 | 0.48 | 0.40 | -0.96 | -0.58 | -0.97 |
| Articles of stone, plaster, cement, mica or similar materials | '68 | -1.00 | 0.16 | 0.41 | -0.08 | -1.00 | -0.65 | 0.20 | -0.03 | 0.35 | -0.96 | 0.16 | 0.01 | 0.05 | 0.58 | -0.65 | -0.13 | 0.45 | -0.21 | -0.63 | 0.57 | -0.14 |
| Ceramic products | '69 | -0.36 | -0.26 | 0.19 | 0.51 | -1.00 | -0.47 | 0.33 | 0.30 | 0.47 | -0.95 | -0.53 | -0.06 | -0.02 | 0.12 | -0.27 | 0.09 | -0.19 | 0.02 | -0.73 | 0.66 | -0.10 |
| Glass and glassware | '70 | -0.99 | 0.29 | -0.19 | 0.11 | -0.99 | -0.50 | -0.02 | 0.08 | 0.68 | -1.00 | 0.09 | 0.27 | 0.07 | -0.56 | -0.32 | 0.04 | -0.19 | 0.04 | -0.30 | 0.16 | 0.06 |
| Natural or cultured pearls, precious stones, imitation jewellery, coin | '71 | -1.00 | 0.55 | -0.31 | -1.00 | -0.41 | 0.06 | -0.38 | -0.24 | -0.68 | 0.18 | -0.85 | -0.48 | -0.52 | -0.75 | 0.20 | -0.94 | 0.80 | -0.31 | -0.93 | 0.04 | -0.65 |
| Iron and steel | '72 | -0.98 | 0.33 | 0.49 | 0.60 | -1.00 | -0.71 | -0.39 | 0.18 | 0.26 | -0.97 | 0.29 | 0.16 | 0.02 | -0.09 | -0.41 | -0.26 | 0.10 | -0.59 | -0.90 | -0.04 | 0.19 |
| Articles of iron or steel | '73 | -0.96 | -0.04 | -0.08 | 0.04 | -0.88 | -0.45 | 0.20 | -0.13 | 0.55 | -1.00 | 0.04 | 0.05 | 0.13 | 0.16 | -0.41 | 0.01 | 0.27 | -0.30 | -0.76 | 0.34 | -0.03 |
| Copper and articles thereof | '74 | -0.99 | 0.07 | -0.45 | 0.83 | -1.00 | 0.96 | -0.44 | -0.50 | -0.37 | -0.81 | 0.26 | -0.06 | 0.05 | 0.54 | -0.07 | -0.34 | -0.12 | 0.11 | -0.90 | -0.12 | -0.04 |
| Nickel and articles thereof | '75 | -0.99 | -0.30 | 0.20 | -0.95 | -1.00 | -1.00 | -0.75 | -0.50 | -0.80 | -0.97 | 0.57 | -0.17 | -0.19 | -0.99 | -0.34 | -0.95 | -0.81 | 0.34 | -0.87 | -0.80 | -0.29 |
| Aluminium and articles thereof | '76 | -1.00 | -0.05 | 0.36 | 0.06 | -1.00 | -0.71 | -0.32 | -0.12 | 0.04 | -1.00 | -0.40 | -0.08 | 0.09 | 0.71 | -0.55 | 0.30 | -0.15 | -0.19 | -0.64 | 0.04 | -0.53 |
| Lead and articles thereof | '78 | -1.00 | 0.25 | -0.92 | 0.93 | -0.95 | -0.32 | 0.55 | -0.10 | -0.21 | 0.00 | -0.88 | -0.17 | -0.02 | 0.20 | -0.65 | -0.73 | -0.70 | -0.63 | -0.31 | -0.68 | -0.85 |
| Zinc and articles thereof | '79 | -1.00 | 0.23 | -0.34 | 0.89 | -0.99 | -0.98 | 0.43 | -0.99 | -0.42 | -1.00 | 0.63 | 0.00 | -0.24 | -0.25 | 0.07 | -0.66 | -0.83 | -0.79 | -0.99 | -0.70 | -0.60 |
| Tin and articles thereof | '80 | -1.00 | -0.01 | 0.34 | -0.98 | -0.99 | -0.95 | 0.64 | -0.93 | -0.72 | -1.00 | -0.91 | -0.49 | -0.56 | -0.92 | 0.06 | -0.93 | 0.02 | 0.86 | -0.79 | -0.74 | -0.37 |
| Other base metals; cermets; articles thereof | '81 | -1.00 | 0.07 | -0.23 | -0.64 | -0.99 | -0.02 | 0.41 | -1.00 | 0.06 | -1.00 | 0.41 | 0.00 | -0.14 | -0.88 | -0.59 | -0.51 | -0.77 | -0.93 | -0.90 | -0.41 | 0.16 |
| Tools, implements, cutlery, spoons and forks, of base metal | '82 | -0.95 | -0.13 | -0.06 | -0.24 | -0.98 | -0.85 | 0.31 | -0.07 | 0.13 | -1.00 | -0.52 | -0.22 | 0.19 | -0.48 | -0.11 | -0.48 | 0.12 | -0.61 | -0.61 | -0.01 | 0.10 |
| Miscellaneous articles of base metal | '83 | -0.93 | -0.46 | -0.26 | -0.28 | -0.99 | -0.69 | 0.23 | -0.36 | 0.44 | -1.00 | -0.34 | 0.03 | 0.26 | 0.00 | 0.13 | 0.01 | -0.11 | -0.62 | -0.87 | 0.38 | -0.42 |
| Machinery, mechanical appliances, nuclear reactors, boilers | '84 | -0.90 | -0.24 | -0.29 | -0.37 | -0.95 | -0.85 | -0.09 | -0.79 | -0.02 | -0.87 | -0.14 | -0.06 | 0.11 | -0.50 | -0.14 | 0.23 | -0.58 | -0.51 | 0.28 | 0.15 | 0.16 |
| Electrical machinery and equipment; sound recorders and reproducers, tv | '85 | -0.98 | -0.36 | -0.40 | -0.55 | -0.99 | -0.92 | 0.14 | -0.78 | 0.01 | -1.00 | 0.24 | -0.11 | -0.10 | -0.43 | 0.29 | 0.27 | -0.63 | -0.16 | 0.10 | -0.33 | 0.21 |
| Railway or tramway locomotives; railway or tramway track | '86 | -0.23 | -0.06 | -0.36 | -0.11 | -1.00 | -0.97 | 0.62 | -0.91 | 0.62 | -0.99 | -0.74 | 0.01 | 0.09 | -0.96 | -0.90 | 0.41 | -0.43 | -0.74 | -0.92 | -0.03 | -0.19 |
| Vehicles other than railway or tramway rolling stock | '87 | -0.98 | 0.23 | -0.04 | -0.90 | -0.94 | -0.76 | -0.68 | -0.38 | 0.25 | -1.00 | -0.40 | 0.17 | 0.31 | -0.82 | -0.87 | -0.01 | -0.62 | -0.84 | -0.85 | -0.07 | 0.37 |
| Aircraft, spacecraft, and parts thereof | '88 | -0.98 | -0.68 | 0.56 | -0.86 | -0.99 | -0.80 | -0.86 | -0.73 | -0.70 | -1.00 | -0.67 | 0.50 | 0.21 | -0.69 | -0.93 | -0.99 | -0.83 | -0.96 | -0.78 | -0.27 | -0.64 |
| Ships, boats and floating structures | '89 | -0.94 | -0.93 | -0.82 | -0.46 | -0.76 | -0.62 | -0.03 | -0.94 | -0.90 | -1.00 | 0.65 | 0.04 | -0.14 | -0.18 | -0.96 | -0.96 | -0.72 | -0.79 | -0.97 | 0.07 | 0.47 |
| Optical, photographic, cinematographic, medical or surgical instruments | '90 | -0.86 | -0.35 | -0.56 | -0.68 | -0.99 | -0.96 | -0.15 | -0.87 | -0.41 | -1.00 | -0.12 | -0.09 | 0.10 | -0.67 | 0.20 | -0.32 | -0.62 | -0.69 | 0.14 | -0.22 | 0.36 |
| Clocks and watches and parts thereof | '91 | -1.00 | -0.69 | -0.96 | -0.75 | -0.99 | -0.97 | 0.31 | -0.94 | -0.87 | -1.00 | -0.93 | -0.25 | -0.45 | -0.76 | 0.78 | -0.90 | -0.40 | -0.94 | -0.94 | -0.26 | -0.10 |
| Musical instruments; parts and accessories of such articles | '92 | -0.99 | -0.56 | -0.73 | -0.64 | -0.24 | -0.95 | 0.44 | -0.97 | 0.49 | -1.00 | -0.91 | -0.28 | -0.04 | -0.83 | -0.10 | -0.77 | -0.57 | 0.72 | -0.95 | 0.03 | 0.46 |
| Arms and ammunition; parts and accessories thereof | '93 | -0.99 | 0.34 | 0.21 | -1.00 | -0.98 | -0.70 | -0.85 | -0.97 | 0.34 | -1.00 | -0.01 | 0.02 | -0.46 | -0.37 | -0.99 | -0.44 | -0.69 | -0.95 | -0.98 | 0.23 | -0.45 |
| Furniture; bedding, mattresses, stuffed furnishings; lamps | '94 | -0.99 | -0.03 | -0.13 | 0.09 | -0.87 | -0.49 | 0.37 | -0.31 | 0.46 | -0.98 | -0.10 | -0.12 | -0.05 | -0.45 | -0.03 | 0.32 | -0.81 | 0.33 | -0.78 | 0.52 | -0.81 |
| Toys, games and sports requisites | '95 | -0.73 | -0.34 | -0.87 | -0.28 | -0.98 | -0.89 | 0.65 | -0.65 | -0.11 | -1.00 | -0.48 | -0.32 | -0.35 | -0.36 | 0.77 | 0.31 | -0.63 | -0.35 | -0.63 | -0.22 | -0.08 |
| Miscellaneous manufactured articles | '96 | -0.77 | -0.21 | -0.07 | -0.28 | -0.91 | -0.51 | 0.46 | 0.16 | 0.20 | -1.00 | -0.64 | 0.06 | 0.09 | -0.18 | 0.46 | -0.49 | 0.12 | -0.07 | -0.29 | 0.12 | 0.27 |
| Works of art, collectors' pieces and antiques | '97 | -0.96 | -0.61 | -0.95 | -0.97 | -0.87 | -0.93 | -0.91 | -0.90 | -0.82 | -0.66 | -0.94 | 0.11 | -0.31 | 0.60 | -0.56 | -0.87 | -0.91 | -0.91 | -0.82 | -0.66 | -0.74 |
| Commodities not elsewhere specified | '99 | -0.95 | -0.62 | -0.14 | 0.46 | -0.96 | -0.04 | -0.87 | -1.00 | -0.62 | -1.00 | -0.69 | -0.99 | 0.14 | -0.20 | -0.96 | -0.51 | 0.02 | -1.00 | 0.14 | -0.31 | 0.13 |

| **Product label** | **Code** | **Kor** | **Mal** | **Mex** | **Neth** | **Nor** | **Paki** | **Phil** | **Pol** | **Port** | **Rom** | **Sing** | **Spa** | **Sweden** | **Switz** | **Taiw** | **Thai** | **Tun** | **Turk** | **UK** | **US** | **Viet** |
| --- | --- | --- | --- | --- | --- | --- | --- | --- | --- | --- | --- | --- | --- | --- | --- | --- | --- | --- | --- | --- | --- | --- |
| Dairy products | '04 | -0.99 | -0.64 | -0.82 | 0.64 | -0.61 | -0.70 | -0.67 | 0.35 | 0.02 | -0.55 | -0.69 | 0.07 | -0.41 | -0.14 | -0.97 | -0.49 | -0.55 | -0.57 | -0.27 | -0.61 | 0.55 |
| Products of animal origin | '05 | -0.59 | -0.82 | -0.77 | 0.17 | -0.17 | 0.35 | -0.76 | 0.40 | 0.15 | -0.07 | -0.65 | 0.06 | -0.38 | -0.70 | 0.27 | -0.07 | 0.57 | 0.24 | -0.49 | 0.19 | 0.55 |
| Lac; gums, resins | '13 | -0.19 | -0.97 | -0.14 | -0.11 | 0.10 | 0.73 | 0.54 | -0.74 | -0.24 | -0.83 | -0.43 | 0.50 | -0.73 | 0.29 | -0.36 | -0.08 | -0.96 | -0.69 | -0.42 | 0.07 | 0.19 |
| Vegetable plaiting materials | '14 | -0.86 | -0.65 | 0.32 | -0.47 | -1.00 | 0.46 | -0.64 | -0.01 | -0.59 | 0.07 | 0.25 | -0.20 | -0.96 | -0.91 | -0.61 | -0.05 | -0.49 | 0.76 | -0.93 | -0.44 | 0.87 |
| Animal or vegetable fats and oils | '15 | -0.94 | 0.83 | -0.85 | 0.37 | -0.62 | 0.10 | 0.60 | -0.64 | 0.19 | -0.20 | -0.31 | 0.52 | -0.40 | -0.94 | -0.85 | -0.30 | 0.54 | 0.41 | -0.58 | -0.23 | 0.09 |
| Preparations of meat, fish, molluscs | '16 | -0.21 | -0.44 | -0.60 | 0.22 | 0.10 | -0.36 | 0.00 | 0.27 | 0.15 | -0.35 | -0.82 | 0.33 | -0.34 | -0.99 | -0.58 | 0.88 | -0.90 | -0.59 | -0.54 | -0.33 | 0.37 |
| Sugars and sugar confectionery | '17 | -0.43 | -0.42 | -0.23 | 0.05 | -0.84 | 0.47 | -0.21 | 0.21 | -0.22 | -0.81 | -0.76 | 0.18 | -0.45 | -0.54 | -0.79 | 0.66 | -0.59 | 0.60 | -0.21 | -0.45 | 0.16 |
| Cocoa and cocoa preparations | '18 | -0.84 | -0.02 | -0.73 | 0.63 | -0.59 | -0.86 | -0.73 | 0.39 | -0.89 | -0.94 | -0.21 | -0.18 | -0.06 | 0.29 | -0.99 | -0.77 | -0.61 | 0.20 | -0.07 | -0.31 | -0.98 |
| Preparations of cereals, flour | '19 | -0.26 | -0.16 | -0.26 | 0.35 | -0.78 | -0.72 | -0.23 | 0.08 | -0.11 | -0.55 | -0.45 | 0.17 | -0.04 | -0.01 | -0.70 | 0.02 | 0.31 | 0.19 | 0.17 | -0.15 | 0.48 |
| Preparations of vegetables, fruit, nuts | '20 | -0.71 | -0.74 | -0.29 | 0.53 | -0.95 | -0.72 | 0.32 | 0.43 | 0.16 | -0.58 | -0.74 | 0.54 | -0.77 | -0.55 | -0.69 | 0.53 | 0.10 | 0.68 | -0.57 | -0.05 | 0.29 |
| Miscellaneous edible preparations | '21 | -0.50 | -0.23 | -0.15 | 0.41 | -0.76 | -0.51 | -0.32 | 0.25 | -0.16 | -0.90 | -0.17 | 0.22 | -0.15 | 0.25 | -0.57 | 0.28 | -0.39 | 0.06 | 0.00 | 0.15 | 0.14 |
| Beverages, spirits and vinegar | '22 | -0.69 | -0.62 | 0.25 | 0.31 | -0.89 | -0.48 | -0.83 | -0.59 | 0.55 | -0.50 | -0.35 | 0.45 | -0.04 | -0.69 | -0.87 | -0.46 | -0.25 | -0.67 | 0.46 | -0.41 | -0.72 |
| Residues and waste from food industries | '23 | -0.87 | -0.50 | -0.89 | 0.48 | -0.19 | -0.81 | -0.47 | -0.15 | -0.30 | -0.29 | -0.81 | -0.23 | -0.83 | -0.53 | -0.79 | 0.11 | -0.23 | -0.63 | -0.42 | 0.25 | -0.78 |
| Tobacco and manufactured tobacco substitutes | '24 | -0.62 | -0.19 | -0.80 | 0.65 | -0.94 | -0.63 | -0.52 | -0.22 | -0.10 | -0.62 | 0.22 | -0.46 | -0.79 | 0.05 | -0.98 | -0.51 | 0.05 | 0.60 | 0.14 | 0.24 | -0.09 |
| Salt; sulphur; earths and stone; lime and cement | '25 | -0.50 | -0.51 | -0.20 | 0.04 | 0.15 | -0.04 | -0.28 | 0.02 | -0.13 | 0.21 | -0.90 | 0.32 | -0.30 | -0.77 | -0.53 | 0.45 | 0.60 | 0.73 | -0.11 | -0.10 | -0.29 |
| Ores, slag and ash | '26 | -0.96 | -0.77 | -0.57 | -0.73 | -0.54 | -0.71 | 0.02 | -0.65 | -0.02 | -0.27 | -0.77 | -0.12 | 0.17 | -0.97 | -0.97 | -0.80 | -0.41 | -0.18 | -0.79 | -0.43 | 0.02 |
| Mineral fuels, mineral oils and products of their distillation | '27 | -0.33 | -0.04 | -0.13 | -0.11 | 0.73 | -0.62 | -0.85 | -0.28 | -0.70 | -0.26 | -0.15 | -0.54 | -0.50 | -0.94 | -0.76 | -0.55 | -0.05 | -0.75 | -0.13 | -0.70 | 0.48 |
| Inorganic chemicals | '28 | -0.40 | -0.72 | -0.48 | 0.06 | 0.05 | -0.93 | -0.68 | -0.06 | -0.56 | 0.29 | -0.73 | -0.30 | -0.43 | -0.30 | -0.53 | -0.62 | 0.68 | 0.00 | 0.09 | 0.12 | -0.83 |
| Organic chemicals | '29 | 0.04 | -0.35 | -0.58 | 0.32 | -0.54 | -0.69 | -0.94 | -0.40 | -0.39 | -0.38 | 0.17 | -0.08 | -0.62 | 0.57 | -0.35 | -0.41 | -0.96 | -0.66 | 0.14 | 0.05 | -0.80 |
| Pharmaceutical products | '30 | -0.87 | -0.93 | -0.56 | 0.15 | -0.67 | -0.67 | -0.92 | -0.64 | -0.32 | -0.92 | -0.67 | -0.03 | 0.47 | 0.73 | -0.96 | -0.85 | -0.91 | -0.65 | 0.39 | -0.05 | -0.92 |
| Fertilisers | '31 | -0.50 | -0.23 | -0.59 | 0.27 | -0.91 | -0.72 | -0.39 | 0.36 | -0.29 | 0.70 | -0.96 | -0.29 | -0.53 | -0.94 | -0.85 | -0.79 | 0.89 | -0.61 | -0.62 | 0.12 | -0.58 |
| Tanning or dyeing extracts; paints and varnishes | '32 | -0.19 | -0.43 | -0.23 | 0.22 | -0.65 | -0.86 | -0.86 | -0.28 | -0.33 | -0.85 | 0.10 | 0.27 | 0.01 | 0.56 | 0.05 | -0.54 | -0.39 | -0.31 | 0.16 | -0.01 | -0.78 |
| Essential oils; perfumery, cosmetics | '33 | -0.74 | -0.76 | -0.32 | -0.03 | -0.87 | -0.83 | -0.81 | 0.14 | -0.62 | -0.73 | -0.05 | 0.25 | -0.42 | 0.44 | -0.56 | -0.02 | -0.12 | -0.35 | 0.30 | 0.05 | -0.48 |
| Soap, washing preparations, waxes, candles | '34 | -0.45 | -0.13 | -0.03 | 0.27 | -0.43 | -0.63 | -0.59 | 0.32 | -0.02 | -0.35 | -0.39 | 0.41 | -0.03 | -0.07 | -0.40 | -0.31 | -0.13 | 0.43 | 0.11 | 0.05 | 0.17 |
| Albuminoidal substances | '35 | -0.34 | -0.68 | -0.76 | 0.41 | -0.81 | -0.63 | -0.90 | 0.18 | -0.66 | -0.96 | -0.40 | -0.41 | 0.14 | 0.15 | 0.04 | 0.28 | -0.66 | -0.67 | 0.00 | 0.18 | -0.57 |
| Explosives; pyrotechnic products; matches | '36 | -0.68 | -0.68 | -0.16 | -0.02 | -0.90 | 0.78 | -0.43 | 0.07 | -0.74 | -0.85 | -0.52 | -0.02 | 0.11 | 0.40 | -0.90 | -0.28 | 0.10 | 0.03 | -0.02 | 0.23 | -0.44 |
| Photographic or cinematographic goods | '37 | -0.57 | -0.61 | -0.13 | 0.20 | -0.77 | -0.92 | -0.99 | -0.94 | -0.65 | -0.95 | 0.39 | -0.23 | -0.31 | -0.35 | -0.68 | -0.87 | -0.98 | -0.86 | 0.30 | 0.14 | -0.44 |
| Miscellaneous chemical products | '38 | -0.42 | 0.07 | -0.64 | 0.22 | -0.38 | -0.90 | -0.65 | -0.61 | -0.23 | -0.74 | -0.05 | -0.10 | -0.03 | 0.23 | -0.20 | -0.62 | -0.71 | -0.64 | 0.19 | 0.23 | -0.82 |
| Plastics and articles thereof | '39 | 0.15 | -0.21 | -0.26 | 0.26 | -0.71 | -0.41 | -0.68 | -0.04 | -0.15 | -0.40 | -0.13 | 0.09 | -0.02 | -0.02 | 0.27 | 0.13 | -0.41 | -0.23 | -0.14 | 0.09 | -0.48 |
| Rubber and articles thereof | '40 | 0.16 | 0.35 | -0.39 | -0.08 | -0.89 | -0.92 | -0.59 | 0.31 | 0.09 | -0.19 | -0.38 | 0.33 | -0.11 | -0.63 | -0.06 | 0.63 | -0.30 | 0.24 | -0.11 | 0.02 | 0.31 |
| Raw hides and skins (other than furskins) and leather | '41 | 0.35 | -0.90 | -0.42 | -0.31 | -0.61 | 0.69 | -0.93 | 0.00 | -0.19 | 0.09 | -0.57 | 0.15 | -0.47 | -0.65 | 0.24 | 0.05 | -0.04 | -0.27 | -0.40 | 0.00 | -0.58 |
| Articles of leather; saddlery; travel goods, handbags | '42 | -0.27 | -0.83 | -0.50 | -0.48 | -0.95 | 0.81 | 0.43 | -0.20 | -0.64 | 0.26 | -0.83 | -0.19 | -0.67 | -0.27 | -0.46 | 0.41 | 0.23 | 0.44 | -0.53 | -0.63 | 0.60 |
| Furskins and artificial fur; manufactures thereof | '43 | -0.56 | -0.92 | -0.95 | -0.31 | 0.10 | -1.00 | -0.91 | 0.48 | -0.11 | -0.07 | -0.97 | 0.39 | -0.39 | -0.94 | -0.74 | -0.93 | -0.99 | 0.66 | -0.53 | -0.38 | 0.07 |
| Wood and articles of wood | '44 | -0.90 | 0.49 | -0.63 | -0.57 | -0.31 | -0.85 | -0.41 | 0.48 | 0.14 | 0.61 | -0.75 | -0.25 | 0.53 | -0.32 | -0.63 | -0.06 | -0.67 | -0.50 | -0.74 | -0.18 | 0.07 |
| Cork and articles of cork | '45 | -0.98 | -0.98 | -0.75 | -0.81 | -0.99 | -0.96 | -1.00 | -0.77 | 0.99 | -0.73 | -0.97 | 0.82 | -0.89 | -0.06 | -0.81 | -0.99 | 0.77 | -0.97 | -0.82 | -0.59 | -0.98 |
| Manufactures of straw, basketware | '46 | -0.72 | -0.95 | -0.86 | -0.25 | -0.97 | -0.75 | 0.87 | 0.54 | -0.83 | 0.27 | -0.94 | -0.56 | -0.59 | -0.98 | -0.33 | -0.27 | 0.06 | -0.93 | -0.86 | -0.78 | 0.94 |
| Pulp of wood or of other fibrous cellulosic material | '47 | -1.00 | -1.00 | -0.90 | -0.27 | 0.01 | -0.98 | -0.55 | -0.72 | 0.69 | -0.63 | -0.71 | 0.04 | 0.73 | -0.53 | -0.96 | -0.20 | -0.49 | -1.00 | -0.78 | 0.26 | -0.99 |
| Paper and paperboard; articles of paper pulp, of paper or of paperboard | '48 | -0.21 | -0.62 | -0.48 | 0.10 | -0.09 | -0.96 | -0.70 | 0.31 | 0.27 | -0.42 | -0.63 | 0.05 | 0.68 | 0.02 | -0.43 | -0.25 | -0.34 | -0.34 | -0.22 | -0.04 | -0.51 |
| Printed books, newspapers, pictures and products of the printing industry | '49 | -0.51 | -0.59 | -0.40 | 0.00 | -0.65 | -0.80 | -0.89 | -0.14 | -0.47 | -0.52 | 0.12 | 0.40 | -0.26 | 0.10 | -0.72 | -0.69 | -0.91 | -0.72 | 0.42 | 0.20 | -0.87 |
| Silk | '50 | 0.35 | -0.88 | -1.00 | -0.94 | -0.99 | -0.78 | -0.88 | -0.96 | -0.91 | -0.23 | -0.58 | -0.74 | -0.99 | -0.27 | -0.95 | 0.05 | -0.39 | -0.73 | -0.48 | -0.82 | 0.81 |
| Wool, fine or coarse animal hair; horsehair yarn and woven fabric | '51 | -0.37 | -0.57 | -0.68 | -0.66 | -0.76 | -0.60 | -0.91 | 0.08 | 0.37 | -0.25 | -0.96 | -0.06 | -0.91 | -0.47 | -0.30 | -0.41 | -0.89 | 0.29 | -0.03 | -0.83 | -0.93 |
| Cotton | '52 | -0.14 | -0.57 | -0.56 | -0.57 | -0.91 | 0.95 | -0.89 | -0.87 | 0.14 | -0.59 | -0.77 | 0.07 | -0.78 | -0.30 | -0.08 | -0.01 | 0.12 | 0.63 | -0.67 | -0.04 | -0.36 |
| Other vegetable textile fibres; paper yarn and woven fabrics of paper yarn | '53 | -0.49 | -0.98 | -0.93 | -0.24 | -0.97 | -0.48 | -0.16 | 0.19 | -0.49 | -0.29 | -0.95 | -0.28 | -0.79 | -0.65 | -0.48 | -0.70 | 0.35 | -0.53 | -0.08 | -0.83 | 0.07 |
| Man-made filaments; strip and the like of man-made textile materials | '54 | 0.67 | 0.00 | -0.28 | -0.47 | -0.88 | 0.84 | -0.64 | -0.16 | -0.36 | -0.28 | -0.56 | 0.07 | -0.76 | -0.20 | 0.68 | 0.14 | -0.67 | 0.52 | -0.15 | -0.26 | -0.04 |
| Man-made staple fibres | '55 | 0.36 | -0.25 | -0.34 | -0.44 | -0.94 | 0.27 | -0.61 | -0.44 | 0.32 | 0.15 | -0.46 | 0.17 | -0.58 | -0.60 | 0.50 | 0.48 | 0.22 | 0.69 | -0.23 | -0.29 | 0.12 |
| Wadding, felt and nonwovens; special yarns; twine, cordage, ropes | '56 | 0.29 | -0.64 | -0.23 | 0.06 | -0.37 | 0.51 | -0.11 | -0.15 | 0.51 | -0.28 | -0.77 | -0.06 | -0.05 | -0.10 | 0.22 | 0.12 | -0.09 | 0.08 | -0.15 | -0.02 | 0.21 |
| Carpets and other textile floor coverings | '57 | -0.70 | -0.83 | -0.58 | 0.45 | -0.94 | 0.87 | -0.79 | -0.03 | 0.16 | -0.07 | -0.86 | -0.52 | -0.45 | -0.08 | -0.91 | -0.23 | -0.52 | 0.73 | -0.09 | -0.13 | -0.27 |
| Special woven fabrics; tufted textile fabrics; lace; tapestries | '58 | 0.58 | -0.79 | -0.27 | -0.39 | -0.89 | 0.17 | -0.21 | -0.56 | -0.03 | -0.37 | -0.72 | -0.16 | -0.60 | 0.19 | 0.52 | 0.17 | -0.17 | 0.76 | -0.29 | -0.03 | -0.39 |
| Impregnated, coated, covered or laminated textile fabrics | '59 | 0.62 | -0.83 | -0.60 | -0.31 | -0.70 | -0.92 | -0.98 | -0.08 | 0.66 | -0.78 | -0.76 | 0.00 | 0.04 | 0.14 | 0.72 | -0.11 | -0.93 | 0.39 | -0.11 | -0.07 | -0.67 |
| Knitted or crocheted fabrics | '60 | 0.73 | -0.28 | -0.59 | -0.60 | -0.89 | 0.31 | -0.54 | -0.54 | -0.27 | -0.75 | -0.34 | -0.02 | -0.58 | -0.52 | 0.75 | -0.32 | -0.78 | 0.52 | -0.55 | -0.29 | -0.76 |
| Articles of apparel and clothing accessories, knitted or crocheted | '61 | 0.01 | -0.28 | 0.20 | -0.36 | -0.92 | 0.79 | 0.28 | -0.05 | 0.67 | 0.61 | -0.18 | -0.26 | -0.67 | -0.72 | -0.14 | 0.34 | 0.72 | 0.79 | -0.46 | -0.43 | 0.29 |
| Articles of apparel and clothing accessories, not knitted or crocheted | '62 | -0.21 | -0.52 | 0.25 | -0.36 | -0.94 | 0.69 | 0.46 | 0.41 | 0.41 | 0.82 | -0.66 | -0.21 | -0.65 | -0.55 | -0.44 | 0.18 | 0.89 | 0.67 | -0.43 | -0.65 | 0.76 |
| Other made-up textile articles; sets; worn clothing | '63 | -0.03 | -0.73 | 0.19 | -0.22 | -0.80 | 0.97 | -0.15 | 0.53 | 0.82 | 0.35 | -0.68 | -0.11 | -0.27 | -0.53 | -0.16 | 0.01 | 0.68 | 0.83 | -0.35 | -0.38 | 0.59 |
| Footwear, gaiters and the like | '64 | -0.27 | -0.79 | -0.56 | -0.18 | -0.94 | 0.00 | -0.56 | -0.04 | 0.77 | 0.83 | -0.78 | 0.39 | -0.80 | -0.66 | -0.35 | 0.28 | 0.72 | -0.31 | -0.53 | -0.75 | 0.89 |
| Headgear and parts thereof | '65 | 0.51 | -0.53 | -0.11 | -0.37 | -0.87 | -0.85 | 0.16 | -0.07 | -0.15 | -0.02 | -0.89 | -0.38 | -0.02 | -0.80 | 0.53 | 0.09 | 0.15 | -0.58 | -0.36 | -0.44 | 0.64 |
| Umbrellas, sun umbrellas, walking sticks | '66 | -0.89 | -0.88 | -0.77 | -0.32 | -0.99 | -0.90 | -0.92 | 0.13 | -0.28 | -0.55 | -0.91 | -0.44 | -0.80 | -0.50 | 0.29 | -0.30 | 0.18 | -0.86 | -0.41 | -0.84 | -0.39 |
| Prepared feathers and down; artificial flowers; articles of human hair | '67 | -0.10 | -0.98 | -0.87 | -0.49 | -0.95 | -1.00 | 0.12 | -0.80 | -0.90 | -0.97 | -0.93 | -0.66 | -0.70 | -0.97 | -0.87 | 0.47 | -0.08 | -0.96 | -0.78 | -0.69 | 0.41 |
| Articles of stone, plaster, cement, mica or similar materials | '68 | -0.39 | -0.50 | -0.07 | -0.16 | -0.58 | -0.24 | -0.69 | 0.22 | 0.48 | -0.53 | -0.79 | 0.48 | -0.11 | 0.02 | -0.42 | -0.33 | -0.47 | 0.40 | -0.05 | -0.21 | -0.40 |
| Ceramic products | '69 | -0.80 | -0.30 | 0.03 | -0.39 | -0.78 | -0.57 | -0.33 | 0.24 | 0.67 | 0.28 | -0.87 | 0.71 | -0.30 | -0.70 | -0.61 | 0.39 | 0.37 | 0.53 | -0.11 | -0.39 | 0.54 |
| Glass and glassware | '70 | -0.14 | -0.11 | 0.08 | -0.16 | -0.58 | -0.76 | -0.32 | 0.41 | 0.31 | 0.22 | -0.53 | 0.14 | -0.13 | -0.16 | -0.01 | -0.09 | -0.68 | 0.45 | -0.18 | 0.03 | -0.56 |
| Natural or cultured pearls, precious stones, imitation jewellery, coin | '71 | -0.24 | -0.49 | -0.60 | -0.85 | -0.69 | -0.80 | -0.69 | -0.56 | -0.77 | -0.34 | -0.33 | -0.68 | -0.69 | 0.48 | -0.92 | 0.21 | -0.85 | -0.13 | 0.43 | 0.02 | -0.56 |
| Iron and steel | '72 | 0.27 | -0.69 | -0.50 | 0.00 | -0.23 | -0.96 | -0.92 | 0.16 | -0.24 | 0.54 | -0.72 | 0.14 | 0.36 | -0.53 | 0.19 | -0.42 | -0.74 | 0.56 | -0.20 | -0.45 | -0.87 |
| Articles of iron or steel | '73 | -0.02 | -0.20 | -0.03 | -0.12 | -0.46 | -0.64 | -0.74 | 0.48 | 0.05 | 0.34 | -0.50 | 0.17 | 0.08 | 0.12 | 0.28 | -0.11 | -0.28 | 0.36 | -0.14 | -0.12 | -0.27 |
| Copper and articles thereof | '74 | 0.01 | -0.11 | -0.10 | -0.48 | -0.50 | -0.74 | 0.25 | 0.52 | -0.59 | -0.03 | -0.35 | -0.04 | 0.06 | -0.23 | 0.21 | -0.31 | -0.46 | 0.05 | -0.30 | -0.33 | -0.89 |
| Nickel and articles thereof | '75 | -0.93 | -0.88 | -0.93 | 0.08 | 0.68 | -1.00 | -1.00 | -0.98 | -1.00 | -0.88 | -0.30 | -0.87 | -0.25 | -0.58 | -0.80 | -0.99 | -0.94 | -0.97 | 0.14 | -0.17 | -0.61 |
| Aluminium and articles thereof | '76 | -0.34 | -0.46 | -0.53 | 0.07 | 0.61 | -0.88 | -0.81 | 0.16 | 0.02 | 0.39 | -0.55 | 0.00 | 0.02 | 0.07 | -0.28 | -0.28 | -0.57 | -0.01 | -0.19 | -0.20 | -0.80 |
| Lead and articles thereof | '78 | -0.27 | 0.21 | -0.58 | -0.46 | -0.86 | -0.96 | -0.92 | 0.41 | -0.77 | 0.45 | 0.24 | -0.61 | 0.21 | -0.70 | -0.25 | -0.81 | -0.38 | -0.51 | 0.14 | -0.41 | -0.76 |
| Zinc and articles thereof | '79 | 0.25 | -0.62 | 0.10 | 0.26 | 0.43 | -1.00 | -0.83 | 0.49 | -0.82 | 0.60 | -0.12 | 0.22 | -0.60 | -0.79 | -0.06 | -0.28 | -0.93 | -0.58 | -0.48 | -0.73 | -0.58 |
| Tin and articles thereof | '80 | -0.76 | 0.74 | -0.92 | -0.47 | -0.91 | -0.99 | -0.54 | -0.97 | -0.20 | -1.00 | 0.74 | -0.83 | -0.73 | -0.89 | -0.04 | 0.71 | -0.85 | -0.95 | -0.50 | -0.44 | 0.61 |
| Other base metals; cermets; articles thereof | '81 | -0.71 | -0.97 | -0.84 | -0.20 | 0.10 | -0.99 | 0.34 | -0.77 | -0.66 | -0.93 | -0.52 | -0.68 | -0.36 | -0.29 | -0.40 | -0.31 | -1.00 | -0.93 | 0.17 | 0.18 | -0.36 |
| Tools, implements, cutlery, spoons and forks, of base metal | '82 | -0.07 | -0.59 | 0.05 | -0.01 | -0.71 | -0.12 | -0.80 | -0.01 | -0.09 | -0.60 | -0.24 | -0.05 | 0.38 | 0.43 | 0.44 | -0.62 | -0.72 | -0.68 | 0.18 | -0.03 | -0.54 |
| Miscellaneous articles of base metal | '83 | -0.34 | -0.54 | 0.38 | -0.19 | -0.47 | -0.96 | -0.55 | 0.18 | 0.20 | -0.41 | -0.50 | 0.26 | 0.06 | -0.19 | 0.30 | -0.32 | -0.30 | -0.19 | -0.16 | 0.07 | -0.71 |
| Machinery, mechanical appliances, nuclear reactors, boilers | '84 | 0.01 | 0.18 | -0.01 | 0.16 | -0.57 | -0.91 | 0.20 | -0.19 | -0.40 | -0.44 | 0.29 | -0.22 | 0.02 | 0.08 | 0.27 | 0.11 | -0.75 | -0.45 | 0.11 | 0.16 | -0.48 |
| Electrical machinery and equipment; sound recorders and reproducers, tv | '85 | 0.25 | 0.44 | 0.30 | -0.23 | -0.67 | -0.96 | 0.53 | -0.16 | -0.07 | -0.26 | 0.44 | -0.31 | 0.04 | -0.25 | 0.29 | 0.20 | -0.10 | -0.33 | 0.02 | 0.09 | -0.48 |
| Railway or tramway locomotives; railway or tramway track | '86 | -0.42 | -0.75 | 0.25 | -0.57 | -0.51 | -1.00 | -0.99 | 0.55 | -0.49 | 0.54 | -0.95 | 0.13 | -0.04 | 0.12 | -0.98 | -0.98 | -0.97 | -0.52 | -0.33 | 0.05 | -1.00 |
| Vehicles other than railway or tramway rolling stock | '87 | 0.03 | -0.92 | 0.30 | -0.26 | -0.80 | -0.95 | -0.63 | -0.01 | 0.21 | -0.63 | -0.86 | 0.45 | 0.12 | -0.78 | -0.50 | -0.34 | -0.63 | -0.10 | -0.11 | -0.05 | -0.74 |
| Aircraft, spacecraft, and parts thereof | '88 | -0.74 | -0.78 | -0.78 | -0.68 | -0.52 | -0.93 | -0.67 | -0.58 | -0.37 | -0.65 | -0.48 | -0.23 | -0.51 | -0.46 | -0.93 | -0.93 | -0.87 | -0.07 | 0.24 | 0.53 | -0.97 |
| Ships, boats and floating structures | '89 | 0.78 | -0.86 | -0.99 | -0.20 | 0.62 | -0.69 | -0.68 | 0.75 | -0.54 | 0.43 | -0.18 | -0.09 | -0.26 | -0.98 | -0.38 | -0.98 | -0.90 | 0.14 | -0.55 | -0.48 | -0.89 |
| Optical, photographic, cinematographic, medical or surgical instruments | '90 | -0.47 | -0.21 | -0.02 | 0.26 | -0.47 | -0.41 | -0.33 | -0.73 | -0.58 | -0.76 | -0.08 | -0.45 | 0.03 | 0.30 | -0.16 | -0.26 | -0.66 | -0.87 | 0.10 | 0.32 | -0.74 |
| Clocks and watches and parts thereof | '91 | -0.47 | -0.36 | -0.60 | -0.76 | -0.90 | -0.97 | 0.18 | -0.85 | -0.76 | -0.97 | 0.16 | -0.42 | -0.82 | 0.92 | -0.56 | 0.20 | -0.45 | -0.89 | -0.59 | -0.66 | -0.79 |
| Musical instruments; parts and accessories of such articles | '92 | 0.42 | 0.42 | -0.12 | -0.27 | -0.96 | -0.53 | -0.94 | -0.77 | -0.87 | 0.07 | -0.22 | -0.24 | -0.54 | -0.32 | 0.51 | -0.47 | -0.97 | -0.78 | -0.33 | 0.03 | -0.59 |
| Arms and ammunition; parts and accessories thereof | '93 | -0.29 | -0.72 | -0.70 | -0.95 | 0.24 | 0.23 | -0.63 | -0.74 | 0.33 | 0.48 | -0.95 | -0.04 | -0.45 | 0.17 | -0.82 | -0.86 | -1.00 | -0.03 | 0.34 | 0.59 | -0.67 |
| Furniture; bedding, mattresses, stuffed furnishings; lamps | '94 | -0.75 | 0.12 | 0.33 | -0.29 | -0.26 | -0.64 | -0.11 | 0.71 | 0.18 | 0.56 | -0.83 | 0.18 | 0.23 | -0.28 | 0.00 | 0.09 | -0.45 | -0.23 | -0.32 | -0.20 | 0.30 |
| Toys, games and sports requisites | '95 | -0.36 | -0.47 | -0.19 | 0.21 | -0.77 | 0.53 | -0.29 | -0.38 | -0.71 | -0.06 | -0.68 | -0.09 | -0.44 | -0.33 | 0.31 | 0.10 | -0.52 | -0.87 | -0.31 | -0.14 | -0.16 |
| Miscellaneous manufactured articles | '96 | 0.25 | -0.30 | 0.05 | -0.14 | -0.92 | 0.30 | -0.26 | -0.28 | -0.48 | -0.68 | -0.31 | -0.06 | -0.40 | 0.08 | 0.38 | 0.10 | -0.14 | -0.32 | -0.16 | -0.18 | 0.18 |
| Works of art, collectors' pieces and antiques | '97 | -0.75 | -0.97 | -0.93 | -0.62 | -0.53 | -0.99 | -0.97 | -0.81 | -0.75 | -0.84 | -0.87 | -0.53 | -0.74 | 0.63 | -0.97 | -0.98 | -0.93 | -0.87 | 0.72 | 0.54 | -0.97 |
| Commodities not elsewhere specified | '99 | -1.00 | -0.53 | -0.96 | 0.81 | 0.21 | -0.97 | -0.98 | -0.99 | -0.85 | -0.73 | 0.11 | -0.28 | 0.23 | -1.00 | -0.88 | 0.08 | -1.00 | -0.96 | -0.81 | 0.04 | -0.62 |

***Sources*: ITC calculations based on UN COMTRADE statistics.**

**Table S 4. Symmetric Balassa Index. 2019**

| **Product label** | **Code** | **Bang** | **Belg** | **Brazil** | **Bulg** | **Camb** | **Chile** | **Chin** | **Col** | **Czech** | **Eth** | **Fin** | **Fran** | **Germ** | **Gre** | **Hon K** | **Hung** | **India** | **Indo** | **Irel** | **Italy** | **Jap** |
| --- | --- | --- | --- | --- | --- | --- | --- | --- | --- | --- | --- | --- | --- | --- | --- | --- | --- | --- | --- | --- | --- | --- |
| Dairy products | '04 | -0.97 | 0.31 | -0.64 | 0.19 | -0.88 | -0.34 | -0.91 | -0.88 | -0.06 | -0.74 | 0.13 | 0.44 | 0.18 | 0.63 | 0.96 | -0.06 | -0.55 | -0.33 | 0.60 | 0.21 | -0.97 |
| Products of animal origin | '05 | -0.60 | -0.26 | 0.71 | -0.07 | -0.99 | 0.09 | 0.21 | 0.28 | -0.40 | 0.16 | -0.75 | -0.10 | 0.06 | -0.34 | 0.99 | 0.29 | -0.31 | -0.65 | -0.42 | -0.30 | -0.74 |
| Lac; gums, resins | '13 | -1.00 | -0.32 | 0.31 | -0.59 | -0.92 | 0.63 | 0.16 | -0.80 | -0.19 | 0.88 | -0.83 | 0.45 | -0.09 | 0.04 | 0.98 | -0.87 | 0.74 | 0.36 | -0.39 | 0.11 | -0.80 |
| Vegetable plaiting materials | '14 | 0.20 | -0.26 | 0.08 | -0.62 | -0.98 | 0.80 | -0.11 | -0.61 | -0.55 | -0.96 | -0.98 | -0.45 | -0.82 | -0.53 | 1.00 | 0.53 | 0.43 | 0.90 | -0.61 | -0.69 | -0.92 |
| Animal or vegetable fats and oils | '15 | -0.40 | -0.13 | 0.09 | 0.41 | -0.44 | -0.06 | -0.83 | 0.50 | -0.44 | -0.15 | -0.86 | -0.35 | -0.45 | 0.47 | 0.75 | 0.01 | -0.13 | 0.91 | -0.82 | -0.09 | -0.87 |
| Preparations of meat, fish, molluscs | '16 | -0.83 | -0.07 | 0.35 | 0.04 | -1.00 | 0.42 | 0.12 | -0.68 | -0.39 | -0.97 | -0.86 | -0.16 | -0.11 | -0.32 | 0.77 | 0.02 | -0.31 | 0.44 | 0.29 | -0.09 | -0.51 |
| Sugars and sugar confectionery | '17 | -0.85 | 0.11 | 0.87 | 0.21 | 0.34 | -0.65 | -0.50 | 0.72 | -0.14 | 0.57 | -0.36 | 0.22 | -0.13 | -0.12 | 0.82 | -0.09 | 0.48 | -0.14 | -0.39 | -0.52 | -0.81 |
| Cocoa and cocoa preparations | '18 | -0.94 | 0.51 | -0.23 | 0.48 | -0.96 | -0.78 | -0.89 | 0.03 | -0.31 | -1.00 | -0.42 | 0.12 | 0.20 | -0.37 | 0.77 | -0.24 | -0.64 | 0.44 | -0.18 | 0.19 | -0.92 |
| Preparations of cereals, flour | '19 | -0.50 | 0.35 | -0.55 | 0.40 | -0.68 | -0.18 | -0.67 | -0.06 | -0.11 | 0.40 | -0.47 | 0.35 | 0.07 | 0.35 | 0.62 | -0.29 | -0.43 | 0.18 | 0.53 | 0.44 | -0.61 |
| Preparations of vegetables, fruit, nuts | '20 | -0.78 | 0.48 | 0.57 | 0.19 | -0.47 | 0.52 | -0.07 | -0.12 | -0.61 | 0.52 | -0.91 | 0.02 | -0.23 | 0.81 | 0.68 | 0.16 | -0.28 | -0.39 | -0.55 | 0.35 | -0.90 |
| Miscellaneous edible preparations | '21 | -0.97 | 0.04 | 0.20 | -0.04 | -0.98 | 0.04 | -0.47 | 0.35 | -0.08 | -0.85 | -0.37 | 0.16 | 0.02 | 0.39 | 0.61 | 0.15 | -0.24 | 0.28 | -0.25 | 0.11 | -0.35 |
| Beverages, spirits and vinegar | '22 | -0.81 | 0.16 | -0.03 | 0.07 | -0.75 | 0.67 | -0.79 | -0.73 | -0.30 | -0.61 | -0.42 | 0.67 | -0.21 | 0.05 | 0.42 | 0.00 | -0.77 | -0.74 | 0.24 | 0.50 | -0.67 |
| Residues and waste from food industries | '23 | -0.86 | 0.12 | 0.79 | 0.42 | -0.65 | 0.22 | -0.58 | -0.58 | -0.07 | -0.58 | -0.50 | 0.15 | -0.13 | -0.22 | 0.58 | 0.35 | 0.05 | 0.06 | -0.27 | -0.35 | -0.93 |
| Tobacco and manufactured tobacco substitutes | '24 | -0.03 | 0.23 | 0.66 | 0.45 | -0.11 | -0.26 | -0.64 | -0.32 | 0.25 | -1.00 | -0.98 | -0.57 | -0.01 | 0.73 | 0.72 | -0.13 | 0.11 | 0.48 | -1.00 | 0.13 | -0.84 |
| Salt; sulphur; earths and stone; lime and cement | '25 | -0.81 | 0.02 | 0.13 | 0.15 | -0.82 | 0.09 | -0.32 | -0.70 | -0.30 | -0.42 | -0.02 | -0.14 | -0.25 | 0.75 | 0.72 | -0.57 | 0.44 | -0.08 | -0.19 | -0.11 | -0.43 |
| Ores, slag and ash | '26 | -0.97 | -0.61 | 0.83 | 0.37 | -0.97 | 0.93 | -0.90 | -0.80 | -0.98 | -0.03 | -0.29 | -0.92 | -0.94 | -0.55 | 0.05 | -0.99 | -0.15 | 0.17 | -0.84 | -0.96 | -0.96 |
| Mineral fuels, mineral oils and products of their distillation | '27 | -0.99 | -0.16 | 0.20 | -0.05 | -1.00 | -0.91 | -0.72 | 0.69 | -0.74 | -1.00 | -0.13 | -0.61 | -0.66 | 0.49 | -0.77 | -0.62 | 0.12 | 0.28 | -0.90 | -0.60 | -0.70 |
| Inorganic chemicals | '28 | -0.80 | 0.06 | 0.53 | 0.23 | -1.00 | 0.69 | -0.01 | -0.42 | -0.37 | -0.88 | 0.24 | -0.02 | -0.01 | -0.08 | 0.33 | -0.20 | -0.07 | -0.03 | -0.11 | -0.36 | 0.15 |
| Organic chemicals | '29 | -0.99 | 0.50 | -0.33 | -0.73 | -0.90 | -0.57 | -0.01 | -0.75 | -0.62 | -1.00 | -0.26 | -0.06 | -0.14 | -0.89 | -0.26 | -0.29 | 0.44 | -0.17 | 0.80 | -0.22 | 0.07 |
| Pharmaceutical products | '30 | -0.87 | 0.55 | -0.67 | 0.02 | -0.97 | -0.83 | -0.81 | -0.54 | -0.40 | -0.89 | -0.54 | 0.31 | 0.28 | 0.28 | -0.45 | 0.18 | 0.21 | -0.82 | 0.80 | 0.30 | -0.57 |
| Fertilisers | '31 | -1.00 | 0.23 | -0.61 | 0.45 | -1.00 | 0.41 | -0.07 | -0.25 | -0.64 | -0.99 | 0.32 | -0.62 | -0.63 | 0.20 | 0.59 | -0.50 | -0.78 | 0.04 | -0.86 | -0.62 | -0.89 |
| Tanning or dyeing extracts; paints and varnishes | '32 | -1.00 | 0.32 | -0.40 | -0.51 | -0.99 | -0.77 | -0.20 | -0.25 | -0.22 | -0.97 | 0.01 | 0.10 | 0.32 | 0.24 | 0.45 | -0.47 | 0.43 | -0.18 | -0.45 | 0.17 | 0.20 |
| Essential oils; perfumery, cosmetics | '33 | -0.99 | -0.09 | -0.32 | 0.15 | -1.00 | -0.69 | -0.57 | 0.24 | 0.00 | -0.19 | -0.77 | 0.63 | -0.03 | 0.03 | 0.18 | -0.13 | -0.05 | -0.29 | 0.72 | 0.14 | -0.01 |
| Soap, washing preparations, waxes, candles | '34 | -0.98 | 0.41 | -0.38 | -0.01 | -1.00 | -0.82 | -0.33 | 0.15 | 0.12 | -1.00 | -0.33 | 0.29 | 0.25 | 0.13 | 0.54 | 0.18 | -0.26 | 0.25 | -0.72 | 0.17 | 0.12 |
| Albuminoidal substances | '35 | -0.96 | 0.17 | 0.28 | 0.33 | -0.99 | -0.60 | -0.14 | -0.13 | -0.70 | -0.94 | 0.44 | 0.35 | 0.23 | -0.52 | 0.72 | -0.31 | -0.38 | -0.67 | 0.33 | -0.01 | -0.10 |
| Explosives; pyrotechnic products; matches | '36 | -0.99 | -0.51 | 0.14 | -0.06 | -0.69 | 0.58 | 0.17 | -0.98 | 0.70 | -1.00 | 0.19 | 0.34 | -0.16 | -0.09 | 0.95 | -0.51 | 0.21 | -0.66 | -0.50 | -0.42 | -0.84 |
| Photographic or cinematographic goods | '37 | -1.00 | 0.43 | -0.70 | -0.55 | -0.97 | -0.95 | -0.32 | -0.96 | -0.54 | -0.99 | -0.96 | -0.21 | 0.04 | -0.79 | 0.82 | -0.34 | -0.92 | -0.98 | -0.44 | -0.67 | 0.79 |
| Miscellaneous chemical products | '38 | -0.94 | 0.31 | -0.35 | 0.24 | -0.97 | -0.68 | -0.27 | 0.08 | -0.37 | -0.94 | 0.03 | 0.34 | 0.21 | -0.04 | -0.16 | -0.15 | 0.18 | 0.36 | 0.35 | -0.01 | 0.20 |
| Plastics and articles thereof | '39 | -0.87 | 0.32 | -0.33 | -0.01 | -0.37 | -0.65 | -0.02 | 0.09 | -0.03 | -0.62 | -0.03 | 0.04 | 0.10 | 0.03 | -0.62 | 0.03 | -0.19 | -0.41 | -0.57 | 0.07 | 0.03 |
| Rubber and articles thereof | '40 | -0.95 | 0.01 | -0.05 | 0.07 | 0.19 | -0.29 | -0.08 | -0.62 | 0.30 | -0.95 | -0.17 | 0.06 | 0.04 | -0.79 | -0.12 | 0.35 | 0.01 | 0.55 | -0.87 | -0.04 | 0.19 |
| Raw hides and skins (other than furskins) and leather | '41 | 0.48 | -0.65 | 0.72 | -0.29 | -0.18 | -0.43 | -0.59 | 0.14 | -0.67 | 0.97 | -0.58 | -0.02 | -0.34 | -0.11 | 0.76 | -0.55 | 0.25 | -0.37 | -0.40 | 0.74 | -0.69 |
| Articles of leather; saddlery; travel goods, handbags | '42 | 0.24 | -0.21 | -0.92 | -0.31 | 0.88 | 0.14 | 0.41 | -0.46 | -0.35 | 0.56 | -0.78 | 0.56 | -0.45 | -0.47 | 0.21 | -0.13 | 0.25 | -0.06 | -0.83 | 0.65 | -0.95 |
| Furskins and artificial fur; manufactures thereof | '43 | -0.98 | -0.89 | -0.46 | 0.05 | 0.95 | -0.99 | 0.59 | -0.48 | -0.91 | -0.99 | 0.80 | -0.40 | -0.72 | 0.82 | 0.87 | -0.93 | -0.85 | -0.99 | -0.93 | 0.17 | -1.00 |
| Wood and articles of wood | '44 | -0.98 | -0.05 | 0.38 | 0.20 | 0.16 | 0.70 | -0.17 | -0.69 | 0.25 | -0.95 | 0.69 | -0.19 | -0.08 | -0.49 | -0.02 | -0.08 | -0.66 | 0.51 | -0.42 | -0.29 | -0.88 |
| Cork and articles of cork | '45 | -1.00 | -0.77 | -0.97 | -0.18 | -0.97 | 0.44 | -0.83 | -0.86 | -0.89 | -1.00 | -0.99 | 0.27 | -0.65 | -0.82 | 0.97 | -0.76 | -0.83 | -0.97 | -0.99 | -0.08 | -0.94 |
| Manufactures of straw, basketware | '46 | 0.53 | -0.49 | -0.96 | -0.74 | -0.65 | -0.99 | 0.63 | -0.80 | -0.80 | 0.14 | -0.92 | -0.50 | -0.61 | -0.86 | 0.96 | -0.77 | 0.02 | 0.59 | -0.98 | -0.57 | -0.95 |
| Pulp of wood or of other fibrous cellulosic material | '47 | -1.00 | -0.37 | 0.89 | 0.00 | -1.00 | 0.90 | -0.96 | -0.98 | -0.18 | -0.98 | 0.87 | -0.33 | -0.50 | -0.44 | 0.45 | -0.71 | -0.98 | 0.73 | -0.79 | -0.66 | -0.45 |
| Paper and paperboard; articles of paper pulp, of paper or of paperboard | '48 | -0.90 | 0.01 | 0.09 | 0.03 | -0.81 | -0.02 | -0.04 | -0.08 | -0.04 | -0.96 | 0.84 | 0.09 | 0.19 | -0.15 | -0.20 | -0.07 | -0.17 | 0.47 | -0.74 | 0.17 | -0.43 |
| Printed books, newspapers, pictures and products of the printing industry | '49 | -0.86 | -0.03 | -0.76 | -0.03 | -0.84 | -0.65 | -0.13 | -0.17 | 0.41 | -0.80 | -0.42 | 0.15 | 0.19 | 0.05 | 0.49 | -0.10 | -0.27 | -0.83 | -0.48 | 0.05 | -0.57 |
| Silk | '50 | -0.99 | -0.82 | 0.20 | -0.47 | -1.00 | -1.00 | 0.56 | -0.98 | -0.98 | -0.46 | -0.96 | 0.08 | -0.69 | -0.83 | 0.96 | -0.97 | 0.42 | -0.90 | -0.97 | 0.65 | -0.24 |
| Wool, fine or coarse animal hair; horsehair yarn and woven fabric | '51 | -0.98 | -0.56 | -0.62 | 0.76 | -1.00 | 0.10 | 0.14 | -1.00 | 0.49 | -1.00 | -0.92 | -0.70 | -0.33 | -0.81 | 0.70 | -0.56 | -0.09 | -0.98 | -0.64 | 0.70 | -0.43 |
| Cotton | '52 | -0.29 | -0.66 | 0.67 | -0.48 | -0.88 | -0.99 | 0.28 | -0.69 | -0.66 | 0.60 | -0.92 | -0.73 | -0.70 | 0.70 | 0.09 | -0.76 | 0.72 | 0.19 | -1.00 | -0.13 | -0.67 |
| Other vegetable textile fibres; paper yarn and woven fabrics of paper yarn | '53 | 0.96 | 0.59 | 0.05 | -0.29 | -1.00 | -0.91 | 0.30 | -0.38 | -0.77 | -0.21 | -0.81 | 0.61 | -0.80 | -0.70 | 0.85 | -0.70 | 0.67 | -0.55 | -0.95 | 0.30 | -0.82 |
| Man-made filaments; strip and the like of man-made textile materials | '54 | -0.95 | -0.22 | -0.69 | -0.01 | -0.81 | -0.93 | 0.51 | -0.21 | -0.22 | -0.41 | -0.96 | -0.52 | -0.47 | 0.03 | 0.11 | -0.45 | 0.45 | 0.27 | -0.96 | 0.13 | 0.04 |
| Man-made staple fibres | '55 | -0.65 | 0.05 | -0.73 | 0.38 | -0.56 | -0.95 | 0.42 | -0.73 | -0.48 | -0.90 | -0.93 | -0.33 | -0.50 | -0.41 | 0.27 | -0.70 | 0.48 | 0.74 | -0.45 | -0.02 | -0.06 |
| Wadding, felt and nonwovens; special yarns; twine, cordage, ropes | '56 | -0.21 | -0.10 | -0.31 | 0.18 | -0.74 | -0.76 | 0.22 | -0.62 | 0.27 | -0.96 | 0.01 | -0.02 | 0.11 | 0.49 | 0.40 | 0.06 | -0.05 | -0.21 | -0.88 | 0.33 | 0.04 |
| Carpets and other textile floor coverings | '57 | -0.11 | 0.61 | -0.85 | -0.41 | -1.00 | -0.99 | 0.14 | -0.95 | -0.19 | -0.71 | -0.65 | -0.32 | -0.36 | -0.39 | 0.53 | -0.58 | 0.73 | -0.44 | -0.84 | -0.36 | -0.86 |
| Special woven fabrics; tufted textile fabrics; lace; tapestries | '58 | -0.82 | -0.25 | -0.57 | -0.11 | -0.79 | -0.97 | 0.50 | -0.20 | -0.29 | 0.14 | -0.66 | -0.04 | -0.28 | -0.14 | 0.60 | -0.59 | 0.31 | -0.43 | -0.99 | 0.16 | -0.40 |
| Impregnated, coated, covered or laminated textile fabrics | '59 | -0.97 | -0.13 | -0.54 | -0.68 | 0.04 | -0.96 | 0.37 | -0.23 | 0.09 | -0.99 | -0.08 | -0.27 | 0.10 | -0.66 | 0.28 | -0.08 | -0.23 | -0.23 | -0.87 | 0.17 | 0.00 |
| Knitted or crocheted fabrics | '60 | -0.64 | -0.72 | -0.72 | -0.28 | -0.09 | -0.86 | 0.57 | -0.09 | -0.58 | 0.47 | -0.83 | -0.51 | -0.58 | 0.25 | 0.11 | -0.54 | -0.17 | -0.52 | -0.93 | -0.03 | -0.41 |
| Articles of apparel and clothing accessories, knitted or crocheted | '61 | 0.94 | -0.06 | -0.92 | 0.29 | 0.94 | -0.98 | 0.36 | -0.48 | -0.41 | 0.87 | -0.83 | -0.17 | -0.27 | 0.15 | -0.69 | -0.58 | 0.32 | 0.26 | -0.88 | 0.18 | -0.95 |
| Articles of apparel and clothing accessories, not knitted or crocheted | '62 | 0.94 | -0.27 | -0.95 | 0.35 | 0.85 | -0.95 | 0.34 | -0.24 | -0.38 | 0.86 | -0.65 | -0.02 | -0.21 | -0.13 | -0.69 | -0.66 | 0.35 | 0.35 | -0.90 | 0.33 | -0.94 |
| Other made-up textile articles; sets; worn clothing | '63 | 0.71 | -0.23 | -0.85 | -0.01 | 0.42 | -0.91 | 0.50 | -0.35 | -0.10 | 0.17 | -0.65 | -0.35 | -0.28 | -0.31 | -0.22 | -0.31 | 0.64 | -0.54 | -0.79 | -0.44 | -0.81 |
| Footwear, gaiters and the like | '64 | 0.50 | 0.29 | -0.14 | -0.01 | 0.83 | -0.98 | 0.39 | -0.79 | -0.31 | 0.69 | -0.62 | -0.01 | -0.15 | -0.01 | -0.56 | -0.17 | 0.05 | 0.53 | -0.94 | 0.48 | -0.98 |
| Headgear and parts thereof | '65 | 0.85 | -0.08 | -0.91 | -0.43 | 0.47 | -0.96 | 0.52 | -0.54 | -0.12 | 0.46 | -0.66 | 0.08 | -0.22 | -0.36 | 0.59 | -0.72 | -0.51 | -0.23 | -0.88 | 0.27 | -0.42 |
| Umbrellas, sun umbrellas, walking sticks | '66 | -0.40 | -0.34 | -0.98 | -0.64 | 0.81 | -1.00 | 0.69 | -0.91 | -0.05 | -0.98 | -0.87 | -0.61 | -0.48 | -0.25 | 0.81 | -0.77 | -0.90 | -0.89 | -0.94 | -0.41 | -0.97 |
| Prepared feathers and down; artificial flowers; articles of human hair | '67 | 0.58 | -0.79 | -0.97 | -0.79 | 0.10 | -1.00 | 0.70 | -0.99 | -0.76 | 0.31 | -0.95 | -0.89 | -0.69 | -0.92 | 0.50 | -0.95 | 0.19 | 0.60 | -0.88 | -0.68 | -0.98 |
| Articles of stone, plaster, cement, mica or similar materials | '68 | -0.94 | -0.05 | 0.33 | 0.03 | -0.97 | -0.85 | 0.20 | -0.38 | 0.09 | -0.62 | 0.12 | -0.14 | 0.10 | 0.40 | -0.31 | 0.12 | 0.29 | -0.53 | -0.41 | 0.31 | 0.09 |
| Ceramic products | '69 | -0.51 | -0.25 | -0.12 | 0.34 | -0.98 | -0.97 | 0.50 | -0.17 | -0.04 | -0.86 | -0.64 | -0.41 | -0.09 | -0.49 | -0.37 | 0.03 | 0.27 | -0.31 | -0.92 | 0.50 | -0.17 |
| Glass and glassware | '70 | -0.95 | 0.19 | -0.57 | 0.55 | -1.00 | -0.66 | 0.24 | -0.03 | 0.37 | -0.90 | -0.10 | 0.18 | 0.06 | -0.56 | -0.49 | 0.22 | -0.19 | -0.43 | -0.72 | 0.09 | 0.04 |
| Natural or cultured pearls, precious stones, imitation jewellery, coin | '71 | -0.99 | 0.06 | -0.20 | -0.81 | -0.10 | -0.19 | -0.63 | 0.21 | -0.83 | 0.52 | -0.60 | -0.35 | -0.50 | -0.86 | -0.92 | -0.92 | 0.53 | 0.05 | -0.93 | -0.11 | -0.36 |
| Iron and steel | '72 | -0.93 | 0.30 | 0.51 | 0.03 | -0.99 | -0.45 | -0.14 | -0.09 | -0.02 | -1.00 | 0.48 | 0.10 | -0.09 | -0.09 | -0.87 | -0.25 | 0.21 | 0.36 | -0.86 | 0.10 | 0.30 |
| Articles of iron or steel | '73 | -0.97 | -0.17 | -0.33 | 0.12 | -0.68 | -0.59 | 0.23 | -0.50 | 0.33 | -0.95 | 0.29 | -0.06 | 0.11 | -0.01 | -0.85 | -0.10 | 0.16 | -0.39 | -0.77 | 0.34 | -0.06 |
| Copper and articles thereof | '74 | -0.75 | -0.01 | -0.19 | 0.81 | -0.55 | 0.94 | -0.50 | -0.21 | -0.56 | -0.83 | 0.36 | -0.22 | -0.01 | 0.43 | -0.71 | -0.58 | -0.47 | 0.16 | -0.91 | -0.04 | 0.16 |
| Nickel and articles thereof | '75 | -0.90 | -0.40 | -0.62 | -0.94 | -1.00 | -1.00 | -0.61 | -0.99 | -0.63 | -1.00 | 0.81 | -0.03 | -0.16 | -1.00 | -0.07 | -0.98 | -0.66 | 0.54 | -0.87 | -0.31 | -0.08 |
| Aluminium and articles thereof | '76 | -0.99 | -0.16 | -0.37 | 0.19 | -0.58 | -0.64 | 0.03 | 0.01 | -0.04 | -0.89 | -0.19 | -0.01 | 0.07 | 0.69 | -0.78 | 0.12 | 0.27 | -0.51 | -0.78 | 0.11 | -0.50 |
| Lead and articles thereof | '78 | -1.00 | 0.36 | -0.57 | 0.88 | -0.65 | 0.18 | -0.88 | -0.44 | 0.29 | -0.82 | -0.41 | -0.08 | -0.22 | 0.35 | 0.50 | -0.76 | 0.52 | -0.52 | -0.20 | -0.29 | -0.62 |
| Zinc and articles thereof | '79 | -0.70 | 0.49 | 0.13 | 0.72 | -0.94 | -0.94 | -0.76 | -0.98 | -0.61 | -0.94 | 0.81 | 0.02 | -0.32 | -0.88 | -0.07 | -0.59 | 0.30 | -0.82 | -0.66 | -0.29 | -0.29 |
| Tin and articles thereof | '80 | -1.00 | 0.28 | 0.57 | -0.84 | -1.00 | -0.98 | -0.72 | -0.98 | -0.80 | -1.00 | -0.88 | -0.42 | -0.58 | -0.99 | 0.39 | -0.05 | -0.77 | 0.93 | -0.97 | -0.61 | -0.39 |
| Other base metals; cermets; articles thereof | '81 | -0.97 | -0.25 | -0.11 | -0.48 | -1.00 | -0.04 | 0.12 | -0.94 | -0.29 | -1.00 | 0.51 | 0.23 | -0.01 | -0.84 | -0.38 | -0.61 | -0.75 | -0.88 | -0.77 | -0.50 | 0.16 |
| Tools, implements, cutlery, spoons and forks, of base metal | '82 | -0.94 | 0.00 | -0.26 | -0.37 | -0.98 | -0.84 | 0.28 | -0.33 | 0.17 | -0.94 | -0.31 | -0.19 | 0.27 | -0.59 | -0.77 | -0.46 | -0.12 | -0.74 | -0.48 | -0.01 | 0.23 |
| Miscellaneous articles of base metal | '83 | -0.99 | -0.51 | 0.28 | 0.17 | -0.74 | -0.73 | 0.31 | -0.66 | 0.32 | -0.97 | -0.22 | -0.12 | 0.16 | -0.04 | -0.80 | -0.01 | -0.31 | -0.73 | -0.54 | 0.25 | -0.52 |
| Machinery, mechanical appliances, nuclear reactors, boilers | '84 | -0.98 | -0.27 | -0.26 | -0.15 | -0.88 | -0.84 | 0.14 | -0.83 | 0.23 | 0.12 | 0.03 | 0.00 | 0.18 | -0.39 | -0.99 | 0.15 | -0.28 | -0.58 | -0.35 | 0.21 | 0.24 |
| Electrical machinery and equipment; sound recorders and reproducers, tv | '85 | -0.98 | -0.62 | -0.77 | -0.12 | -0.59 | -0.95 | 0.28 | -0.84 | 0.09 | -0.43 | -0.32 | -0.31 | -0.17 | -0.62 | -0.99 | 0.21 | -0.51 | -0.47 | -0.37 | -0.43 | 0.00 |
| Railway or tramway locomotives; railway or tramway track | '86 | -0.99 | -0.63 | -0.50 | 0.38 | -1.00 | -0.96 | 0.25 | -0.97 | 0.38 | -0.99 | -0.67 | -0.06 | 0.12 | -0.96 | -0.72 | 0.22 | -0.31 | -0.57 | -0.89 | 0.00 | -0.32 |
| Vehicles other than railway or tramway rolling stock | '87 | -0.95 | 0.20 | -0.22 | -0.38 | -0.47 | -0.91 | -0.48 | -0.63 | 0.41 | -0.97 | -0.04 | 0.07 | 0.33 | -0.88 | -0.99 | 0.36 | -0.19 | -0.26 | -0.94 | -0.03 | 0.45 |
| Aircraft, spacecraft, and parts thereof | '88 | -0.86 | -0.75 | 0.07 | -0.82 | -1.00 | -0.97 | -0.85 | -0.83 | -0.68 | -0.30 | -0.75 | 0.68 | 0.22 | -0.91 | -0.97 | -0.94 | -0.59 | -0.92 | 0.22 | -0.35 | -0.47 |
| Ships, boats and floating structures | '89 | -0.93 | -0.86 | 0.42 | -0.81 | -0.98 | -0.66 | 0.18 | -0.85 | -0.96 | -0.99 | 0.63 | -0.07 | -0.24 | -0.40 | -0.93 | -0.96 | 0.47 | -0.61 | -0.98 | 0.16 | 0.50 |
| Optical, photographic, cinematographic, medical or surgical instruments | '90 | -0.87 | -0.07 | -0.70 | -0.28 | -0.98 | -0.95 | -0.08 | -0.84 | -0.24 | -0.77 | 0.16 | -0.03 | 0.22 | -0.52 | -0.99 | -0.03 | -0.51 | -0.80 | 0.45 | -0.16 | 0.25 |
| Clocks and watches and parts thereof | '91 | -0.99 | -0.71 | -0.98 | -0.68 | -0.61 | -0.95 | -0.20 | -0.98 | -0.67 | -0.75 | -0.83 | 0.31 | -0.40 | -0.39 | -0.92 | -0.81 | -0.81 | -0.92 | -0.92 | -0.16 | -0.36 |
| Musical instruments; parts and accessories of such articles | '92 | -1.00 | -0.17 | -0.87 | -0.52 | -0.92 | -0.99 | 0.27 | -0.91 | -0.08 | -0.95 | -0.74 | 0.14 | 0.14 | -0.72 | -0.60 | -0.77 | -0.64 | 0.81 | -0.88 | -0.16 | 0.42 |
| Arms and ammunition; parts and accessories thereof | '93 | -0.93 | -0.64 | 0.38 | -1.00 | -0.14 | -1.00 | -0.89 | -0.03 | 0.43 | -0.83 | 0.22 | -0.62 | -0.47 | -0.29 | -0.90 | -0.39 | -0.39 | -0.99 | -0.98 | 0.43 | -0.67 |
| Furniture; bedding, mattresses, stuffed furnishings; lamps | '94 | -0.77 | -0.42 | -0.54 | 0.24 | 0.32 | -0.95 | 0.46 | -0.61 | 0.29 | -0.94 | -0.35 | -0.33 | -0.09 | -0.46 | -1.00 | 0.02 | -0.42 | -0.08 | -0.77 | 0.28 | -0.80 |
| Toys, games and sports requisites | '95 | -0.57 | -0.29 | -0.96 | 0.28 | -0.43 | -0.98 | 0.57 | -0.84 | 0.45 | -0.98 | -0.56 | -0.33 | -0.27 | 0.13 | -0.99 | -0.11 | -0.66 | -0.36 | -0.78 | -0.31 | -0.16 |
| Miscellaneous manufactured articles | '96 | -0.81 | 0.01 | -0.48 | -0.31 | -0.47 | -0.77 | 0.41 | 0.12 | 0.37 | -0.80 | -0.81 | 0.01 | 0.01 | 0.10 | -0.99 | 0.20 | -0.19 | -0.10 | -0.46 | -0.01 | 0.24 |
| Works of art, collectors' pieces and antiques | '97 | -0.99 | -0.71 | 0.04 | -0.95 | -0.98 | -0.99 | -0.75 | -0.86 | -0.92 | -0.23 | -0.93 | 0.12 | -0.55 | -0.67 | -0.99 | -0.98 | -0.66 | -0.97 | -0.84 | -0.48 | -0.60 |
| Commodities not elsewhere specified | '99 | -0.90 | -0.84 | -1.00 | -0.18 | -0.99 | 0.03 | -0.75 | -0.98 | -0.91 | 0.58 | -0.24 | -0.96 | -0.16 | -0.04 | -1.00 | -0.85 | -0.96 | -0.97 | -0.67 | -0.41 | 0.34 |

| **Product label** | **Code** | **Kor** | **Mal** | **Mex** | **Neth** | **Nor** | **Paki** | **Phil** | **Pol** | **Port** | **Rom** | **Sing** | **Spa** | **Swe** | **Switz** | **Taiwan** | **Thai** | **Tun** | **Turk** | **UK** | **US** | **Viet** |
| --- | --- | --- | --- | --- | --- | --- | --- | --- | --- | --- | --- | --- | --- | --- | --- | --- | --- | --- | --- | --- | --- | --- |
| Dairy products | '04 | -0.96 | -0.35 | -0.78 | 0.56 | -0.68 | -0.35 | -0.80 | 0.39 | 0.08 | -0.23 | -0.74 | 0.06 | -0.38 | -0.37 | -0.96 | -0.56 | -0.44 | -0.13 | 0.02 | -0.22 | -0.78 |
| Products of animal origin | '05 | -0.57 | -0.88 | -0.66 | 0.31 | -0.19 | 0.52 | -0.89 | 0.44 | 0.48 | -0.12 | -0.88 | 0.33 | -0.48 | -0.70 | 0.13 | -0.43 | -0.63 | -0.21 | -0.29 | 0.15 | -0.22 |
| Lac; gums, resins | '13 | -0.42 | -0.84 | -0.16 | -0.41 | -0.99 | 0.63 | 0.73 | -0.60 | -0.63 | -0.91 | -0.69 | 0.59 | -0.88 | -0.07 | -0.88 | -0.59 | -0.60 | -0.61 | -0.26 | -0.03 | -0.23 |
| Vegetable plaiting materials | '14 | -0.99 | 0.68 | 0.31 | 0.04 | -1.00 | 0.72 | -0.58 | -0.62 | -0.59 | -0.51 | -0.51 | -0.28 | -0.87 | -0.97 | -0.79 | -0.23 | -0.37 | 0.07 | -0.84 | -0.57 | 0.21 |
| Animal or vegetable fats and oils | '15 | -0.94 | 0.81 | -0.70 | 0.27 | -0.30 | -0.39 | 0.47 | -0.40 | 0.45 | -0.19 | -0.75 | 0.52 | -0.13 | -0.91 | -0.89 | -0.43 | 0.77 | 0.02 | -0.53 | -0.43 | -0.72 |
| Preparations of meat, fish, molluscs | '16 | -0.72 | -0.35 | -0.75 | 0.10 | -0.64 | -0.81 | 0.37 | 0.48 | 0.26 | -0.02 | -0.87 | 0.36 | -0.29 | -0.97 | -0.75 | 0.81 | -0.15 | -0.59 | -0.45 | -0.37 | 0.51 |
| Sugars and sugar confectionery | '17 | -0.59 | -0.41 | 0.28 | 0.16 | -0.94 | 0.77 | -0.23 | 0.18 | -0.16 | -0.57 | -0.83 | 0.04 | -0.33 | -0.62 | -0.66 | 0.73 | 0.33 | 0.25 | -0.32 | -0.29 | -0.63 |
| Cocoa and cocoa preparations | '18 | -0.93 | 0.40 | -0.26 | 0.55 | -0.68 | -0.97 | -0.85 | 0.46 | -0.69 | -0.23 | -0.17 | -0.05 | -0.06 | 0.00 | -0.97 | -0.80 | -0.19 | 0.16 | -0.10 | -0.39 | -0.93 |
| Preparations of cereals, flour | '19 | -0.37 | 0.15 | 0.05 | 0.41 | -0.75 | -0.18 | -0.01 | 0.41 | 0.18 | -0.15 | -0.01 | 0.22 | 0.02 | -0.28 | -0.46 | 0.19 | 0.19 | 0.44 | 0.07 | -0.24 | -0.20 |
| Preparations of vegetables, fruit, nuts | '20 | -0.66 | -0.69 | 0.02 | 0.50 | -0.92 | -0.14 | 0.38 | 0.28 | 0.35 | -0.56 | -0.85 | 0.53 | -0.54 | -0.87 | -0.76 | 0.42 | -0.55 | 0.50 | -0.40 | -0.06 | -0.05 |
| Miscellaneous edible preparations | '21 | -0.30 | 0.14 | -0.27 | 0.37 | -0.33 | -0.41 | -0.32 | 0.35 | -0.12 | -0.23 | 0.53 | 0.17 | 0.06 | -0.13 | -0.26 | 0.41 | -0.22 | -0.03 | 0.17 | 0.15 | -0.18 |
| Beverages, spirits and vinegar | '22 | -0.62 | -0.41 | 0.42 | 0.26 | -0.67 | 0.39 | -0.72 | -0.30 | 0.47 | -0.47 | 0.07 | 0.42 | -0.02 | -0.05 | -0.70 | 0.11 | -0.47 | -0.54 | 0.52 | -0.13 | -0.71 |
| Residues and waste from food industries | '23 | -0.84 | -0.34 | -0.72 | 0.39 | 0.06 | -0.15 | -0.60 | 0.21 | -0.21 | -0.09 | -0.73 | -0.03 | -0.52 | -0.71 | -0.82 | 0.30 | -0.30 | -0.53 | -0.16 | 0.22 | -0.22 |
| Tobacco and manufactured tobacco substitutes | '24 | -0.18 | -0.64 | -0.63 | -0.05 | -0.99 | -0.36 | 0.45 | 0.74 | 0.61 | 0.70 | 0.08 | -0.45 | -0.02 | -0.16 | -0.72 | -0.42 | 0.20 | 0.36 | -0.86 | -0.37 | -0.28 |
| Salt; sulphur; earths and stone; lime and cement | '25 | -0.52 | -0.14 | -0.14 | -0.05 | 0.30 | 0.79 | -0.72 | -0.40 | 0.35 | -0.44 | -0.90 | 0.26 | -0.47 | -0.86 | -0.62 | 0.25 | 0.66 | 0.73 | -0.28 | -0.21 | 0.47 |
| Ores, slag and ash | '26 | -0.91 | -0.38 | -0.05 | -0.66 | -0.64 | -0.48 | 0.15 | -0.94 | -0.24 | -0.85 | -0.98 | -0.26 | 0.22 | -0.99 | -0.66 | -0.92 | -0.93 | -0.29 | -0.94 | -0.48 | -0.94 |
| Mineral fuels, mineral oils and products of their distillation | '27 | -0.21 | 0.11 | -0.34 | 0.10 | 0.70 | -0.72 | -0.77 | -0.67 | -0.30 | -0.49 | 0.05 | -0.25 | -0.27 | -0.87 | -0.50 | -0.53 | -0.35 | -0.45 | -0.13 | 0.06 | -0.77 |
| Inorganic chemicals | '28 | 0.15 | -0.27 | -0.51 | 0.12 | -0.03 | -0.72 | -0.60 | -0.22 | -0.60 | -0.36 | -0.64 | -0.12 | -0.42 | -0.63 | -0.15 | -0.36 | 0.58 | 0.23 | -0.18 | 0.03 | -0.22 |
| Organic chemicals | '29 | 0.24 | -0.17 | -0.66 | 0.15 | -0.20 | -0.98 | -0.65 | -0.58 | -0.13 | -0.76 | 0.19 | -0.20 | -0.34 | 0.50 | 0.07 | -0.09 | -0.97 | -0.74 | 0.09 | 0.05 | -0.80 |
| Pharmaceutical products | '30 | -0.66 | -0.93 | -0.82 | 0.23 | -0.61 | -0.53 | -0.96 | -0.37 | -0.29 | -0.47 | -0.24 | 0.11 | 0.32 | 0.76 | -0.90 | -0.87 | -0.74 | -0.63 | 0.26 | 0.00 | -0.96 |
| Fertilisers | '31 | -0.68 | -0.10 | -0.63 | 0.10 | -0.79 | -1.00 | -0.79 | -0.05 | -0.28 | -0.21 | -0.96 | 0.03 | -0.68 | -0.96 | -0.79 | -0.59 | 0.66 | -0.60 | -0.64 | -0.10 | -0.52 |
| Tanning or dyeing extracts; paints and varnishes | '32 | -0.04 | -0.21 | -0.43 | 0.29 | -0.54 | -0.30 | -0.51 | 0.02 | -0.18 | -0.59 | -0.11 | 0.38 | 0.14 | 0.12 | -0.04 | -0.26 | -0.40 | 0.05 | 0.24 | 0.03 | -0.79 |
| Essential oils; perfumery, cosmetics | '33 | 0.16 | -0.63 | -0.25 | -0.05 | -0.86 | -0.74 | -0.72 | 0.31 | -0.49 | -0.17 | 0.50 | 0.37 | -0.43 | 0.16 | -0.56 | 0.13 | -0.04 | -0.26 | 0.19 | 0.03 | -0.72 |
| Soap, washing preparations, waxes, candles | '34 | -0.26 | 0.09 | -0.29 | 0.23 | -0.58 | -0.39 | -0.15 | 0.48 | -0.10 | 0.05 | -0.14 | 0.18 | 0.07 | -0.38 | -0.42 | 0.01 | -0.51 | 0.17 | 0.16 | 0.12 | -0.23 |
| Albuminoidal substances | '35 | -0.15 | -0.38 | -0.60 | 0.47 | -0.66 | -0.50 | -0.94 | -0.20 | -0.05 | -0.71 | -0.29 | -0.30 | -0.03 | -0.04 | 0.05 | 0.38 | -0.59 | -0.13 | -0.15 | 0.16 | -0.39 |
| Explosives; pyrotechnic products; matches | '36 | -0.70 | -0.58 | 0.10 | -0.27 | -0.38 | 0.28 | 0.29 | -0.10 | -0.48 | -0.53 | -0.26 | 0.17 | -0.10 | -0.31 | -0.98 | -0.71 | -0.70 | -0.33 | -0.14 | 0.32 | -0.94 |
| Photographic or cinematographic goods | '37 | 0.35 | -0.10 | -0.72 | 0.45 | -0.99 | -0.96 | -0.98 | -0.76 | -0.71 | -0.86 | -0.31 | -0.48 | -0.71 | -0.92 | 0.37 | -0.90 | -0.96 | -0.83 | -0.31 | 0.26 | -0.98 |
| Miscellaneous chemical products | '38 | -0.20 | 0.19 | -0.65 | 0.29 | -0.21 | -0.87 | -0.62 | -0.09 | -0.27 | -0.56 | 0.19 | 0.18 | 0.07 | -0.48 | -0.07 | -0.39 | -0.75 | -0.44 | 0.13 | 0.26 | -0.54 |
| Plastics and articles thereof | '39 | 0.25 | 0.06 | -0.25 | 0.12 | -0.74 | -0.33 | -0.41 | 0.15 | 0.18 | -0.17 | 0.05 | 0.08 | 0.00 | -0.36 | 0.26 | 0.23 | 0.03 | 0.04 | -0.15 | 0.09 | -0.28 |
| Rubber and articles thereof | '40 | 0.12 | 0.48 | -0.17 | -0.12 | -0.82 | -0.70 | -0.21 | 0.35 | 0.34 | 0.54 | -0.35 | 0.15 | -0.21 | -0.78 | -0.12 | 0.72 | -0.77 | 0.24 | -0.29 | -0.10 | 0.25 |
| Raw hides and skins (other than furskins) and leather | '41 | -0.09 | -0.84 | -0.29 | -0.23 | -0.49 | 0.82 | -0.92 | 0.00 | 0.25 | 0.07 | -0.63 | 0.34 | -0.46 | -0.59 | -0.08 | 0.44 | 0.09 | 0.06 | -0.20 | 0.00 | 0.26 |
| Articles of leather; saddlery; travel goods, handbags | '42 | -0.77 | -0.89 | -0.68 | -0.08 | -0.91 | 0.73 | 0.42 | -0.27 | -0.18 | -0.06 | -0.03 | 0.00 | -0.47 | -0.35 | -0.73 | -0.33 | 0.18 | -0.41 | -0.28 | -0.64 | 0.48 |
| Furskins and artificial fur; manufactures thereof | '43 | -0.87 | -0.21 | -1.00 | -0.53 | -0.04 | -0.95 | -0.62 | 0.24 | -0.52 | -0.89 | -0.96 | -0.24 | -0.68 | -0.91 | -0.98 | 0.31 | -1.00 | 0.30 | -0.69 | -0.70 | -0.29 |
| Wood and articles of wood | '44 | -0.96 | 0.24 | -0.72 | -0.42 | -0.02 | -0.60 | 0.12 | 0.30 | 0.22 | 0.53 | -0.94 | -0.13 | 0.55 | -0.63 | -0.89 | 0.14 | -0.61 | -0.17 | -0.71 | -0.17 | 0.30 |
| Cork and articles of cork | '45 | -0.98 | -0.98 | -0.91 | -0.78 | -0.99 | -1.00 | -0.92 | -0.52 | 0.99 | -0.98 | -0.95 | 0.84 | -0.85 | -0.64 | -0.91 | -0.99 | 0.63 | -0.93 | -0.77 | -0.76 | -0.97 |
| Manufactures of straw, basketware | '46 | -0.98 | -0.93 | -0.64 | -0.12 | -0.98 | -0.59 | 0.54 | 0.05 | -0.90 | -0.96 | -0.96 | -0.11 | -0.44 | -0.96 | -0.58 | -0.59 | -0.67 | -0.90 | -0.77 | -0.86 | 0.77 |
| Pulp of wood or of other fibrous cellulosic material | '47 | -0.88 | -0.49 | -0.98 | -0.04 | 0.05 | -0.99 | -0.15 | -0.41 | 0.61 | -0.74 | -0.19 | -0.11 | 0.74 | -0.86 | -0.71 | -0.62 | -0.97 | -0.91 | -0.36 | 0.36 | -0.94 |
| Paper and paperboard; articles of paper pulp, of paper or of paperboard | '48 | -0.28 | -0.35 | -0.44 | 0.01 | -0.31 | -0.60 | -0.69 | 0.36 | 0.56 | -0.23 | -0.41 | 0.19 | 0.71 | -0.49 | -0.34 | -0.20 | -0.04 | 0.06 | -0.18 | 0.01 | -0.35 |
| Printed books, newspapers, pictures and products of the printing industry | '49 | -0.68 | -0.23 | -0.42 | 0.24 | -0.44 | -0.72 | -0.80 | 0.62 | -0.34 | -0.16 | -0.14 | 0.16 | -0.19 | 0.01 | -0.38 | -0.74 | -0.61 | -0.58 | 0.59 | 0.14 | -0.71 |
| Silk | '50 | -0.24 | -0.96 | -0.92 | -0.97 | -0.99 | -0.70 | -0.98 | -0.96 | -0.94 | 0.85 | -0.85 | -0.84 | -0.97 | -0.72 | -0.99 | -0.47 | 0.21 | -0.71 | -0.24 | -0.92 | 0.59 |
| Wool, fine or coarse animal hair; horsehair yarn and woven fabric | '51 | -0.65 | -0.73 | -0.76 | -0.90 | -0.24 | -0.68 | -0.99 | -0.18 | 0.17 | 0.60 | -0.96 | -0.26 | -0.59 | -0.71 | -0.74 | -0.60 | -0.31 | -0.09 | 0.20 | -0.90 | -0.93 |
| Cotton | '52 | -0.63 | -0.39 | -0.49 | -0.70 | -0.97 | 0.96 | -0.98 | -0.87 | -0.09 | -0.63 | -0.95 | -0.17 | -0.94 | -0.75 | -0.46 | -0.25 | -0.11 | 0.52 | -0.76 | 0.24 | 0.59 |
| Other vegetable textile fibres; paper yarn and woven fabrics of paper yarn | '53 | -0.82 | -0.86 | -0.98 | -0.39 | -0.97 | -0.07 | 0.25 | -0.26 | -0.37 | -0.65 | -0.99 | -0.02 | -0.92 | -0.81 | -0.92 | -0.51 | 0.74 | 0.02 | -0.42 | -0.92 | -0.34 |
| Man-made filaments; strip and the like of man-made textile materials | '54 | 0.28 | -0.27 | -0.67 | -0.14 | -0.99 | -0.13 | -0.86 | -0.47 | -0.38 | -0.04 | -0.68 | -0.05 | -0.92 | -0.71 | 0.47 | 0.06 | -0.41 | 0.53 | -0.42 | -0.45 | 0.23 |
| Man-made staple fibres | '55 | 0.21 | -0.18 | -0.69 | -0.64 | -0.98 | 0.77 | -0.76 | -0.65 | 0.40 | 0.23 | -0.79 | 0.10 | -0.91 | -0.82 | 0.08 | 0.44 | -0.29 | 0.62 | -0.46 | -0.18 | 0.13 |
| Wadding, felt and nonwovens; special yarns; twine, cordage, ropes | '56 | -0.05 | -0.19 | -0.49 | -0.09 | -0.48 | -0.14 | -0.34 | 0.12 | 0.49 | -0.17 | -0.82 | 0.13 | -0.04 | -0.62 | 0.01 | 0.21 | 0.07 | 0.52 | -0.25 | 0.01 | 0.04 |
| Carpets and other textile floor coverings | '57 | -0.91 | -0.83 | -0.63 | 0.44 | -0.97 | 0.58 | -0.98 | 0.04 | 0.22 | -0.54 | -0.94 | -0.32 | -0.10 | -0.47 | -0.95 | -0.12 | -0.80 | 0.89 | -0.07 | -0.20 | -0.11 |
| Special woven fabrics; tufted textile fabrics; lace; tapestries | '58 | -0.12 | -0.80 | -0.55 | -0.53 | -0.96 | 0.31 | -0.18 | -0.49 | 0.45 | 0.20 | -0.86 | -0.09 | -0.69 | -0.48 | 0.44 | 0.17 | 0.06 | 0.56 | -0.30 | -0.40 | -0.18 |
| Impregnated, coated, covered or laminated textile fabrics | '59 | 0.23 | -0.59 | -0.33 | -0.07 | -0.74 | -0.50 | -0.81 | 0.07 | 0.54 | 0.18 | -0.70 | -0.06 | -0.24 | -0.20 | 0.36 | -0.34 | -0.65 | 0.11 | -0.25 | -0.02 | 0.33 |
| Knitted or crocheted fabrics | '60 | 0.41 | -0.27 | -0.88 | -0.73 | -0.98 | -0.05 | -0.97 | -0.73 | 0.05 | -0.63 | -0.83 | -0.20 | -0.69 | -0.84 | 0.54 | -0.13 | -0.74 | 0.63 | -0.74 | -0.59 | 0.40 |
| Articles of apparel and clothing accessories, knitted or crocheted | '61 | -0.81 | -0.52 | -0.54 | -0.12 | -0.90 | 0.84 | -0.29 | -0.01 | 0.47 | -0.18 | -0.77 | 0.19 | -0.37 | -0.63 | -0.88 | -0.29 | 0.48 | 0.61 | -0.22 | -0.75 | 0.64 |
| Articles of apparel and clothing accessories, not knitted or crocheted | '62 | -0.77 | -0.80 | -0.44 | -0.12 | -0.94 | 0.83 | -0.45 | 0.07 | 0.13 | 0.34 | -0.77 | 0.38 | -0.23 | -0.45 | -0.91 | -0.57 | 0.80 | 0.50 | -0.14 | -0.80 | 0.65 |
| Other made-up textile articles; sets; worn clothing | '63 | -0.57 | -0.67 | -0.17 | -0.21 | -0.75 | 0.96 | -0.50 | 0.19 | 0.49 | 0.25 | -0.86 | -0.16 | -0.34 | -0.79 | -0.56 | -0.34 | 0.61 | 0.53 | -0.23 | -0.45 | 0.33 |
| Footwear, gaiters and the like | '64 | -0.81 | -0.85 | -0.74 | -0.07 | -0.94 | -0.10 | -0.62 | 0.09 | 0.58 | 0.37 | -0.69 | 0.13 | -0.40 | -0.45 | -0.86 | -0.51 | 0.56 | -0.19 | -0.22 | -0.77 | 0.81 |
| Headgear and parts thereof | '65 | -0.40 | -0.43 | -0.19 | 0.13 | -0.80 | -0.64 | -0.22 | 0.10 | 0.23 | -0.39 | -0.77 | -0.09 | -0.04 | -0.78 | -0.28 | -0.34 | -0.43 | -0.40 | -0.07 | -0.46 | 0.51 |
| Umbrellas, sun umbrellas, walking sticks | '66 | -0.95 | -0.94 | -0.91 | -0.05 | -0.99 | -0.80 | -0.52 | -0.16 | -0.17 | -0.73 | -0.96 | -0.35 | -0.65 | -0.38 | -0.44 | -0.88 | -0.12 | -0.68 | -0.66 | -0.78 | -0.34 |
| Prepared feathers and down; artificial flowers; articles of human hair | '67 | -0.76 | -0.99 | -0.95 | -0.51 | -0.98 | -0.85 | 0.06 | -0.76 | -0.76 | -0.90 | -0.98 | -0.82 | -0.69 | -0.99 | -0.95 | -0.69 | 0.09 | -0.93 | -0.73 | -0.73 | -0.39 |
| Articles of stone, plaster, cement, mica or similar materials | '68 | -0.33 | -0.04 | -0.17 | -0.17 | -0.61 | -0.48 | -0.31 | 0.35 | 0.49 | -0.35 | -0.81 | 0.41 | -0.21 | -0.39 | -0.47 | -0.13 | -0.45 | 0.47 | -0.10 | -0.08 | -0.16 |
| Ceramic products | '69 | -0.67 | -0.54 | -0.08 | -0.44 | -0.86 | -0.81 | -0.70 | 0.11 | 0.56 | -0.21 | -0.86 | 0.58 | -0.56 | -0.77 | -0.81 | 0.05 | -0.13 | 0.35 | -0.45 | -0.43 | -0.23 |
| Glass and glassware | '70 | -0.15 | 0.11 | -0.04 | -0.29 | -0.66 | -0.61 | -0.48 | 0.36 | 0.37 | -0.21 | -0.69 | 0.10 | -0.23 | -0.48 | 0.10 | -0.16 | -0.09 | 0.21 | -0.21 | -0.10 | -0.11 |
| Natural or cultured pearls, precious stones, imitation jewellery, coin | '71 | -0.74 | -0.53 | -0.39 | -0.92 | -0.70 | -0.96 | -0.21 | -0.71 | -0.76 | -0.82 | 0.13 | -0.65 | -0.65 | 0.75 | -0.78 | 0.29 | -0.97 | 0.09 | 0.43 | 0.03 | -0.61 |
| Iron and steel | '72 | 0.33 | -0.07 | -0.51 | 0.03 | -0.25 | -0.74 | -0.79 | -0.02 | 0.05 | 0.17 | -0.66 | 0.12 | 0.35 | -0.72 | 0.12 | -0.60 | -0.35 | 0.48 | -0.18 | -0.38 | -0.09 |
| Articles of iron or steel | '73 | -0.02 | -0.32 | -0.05 | -0.10 | -0.27 | -0.68 | -0.44 | 0.34 | 0.23 | 0.29 | -0.49 | 0.20 | 0.06 | -0.31 | 0.15 | 0.04 | 0.12 | 0.39 | -0.16 | -0.18 | -0.12 |
| Copper and articles thereof | '74 | 0.02 | 0.20 | -0.24 | -0.31 | -0.43 | 0.37 | 0.44 | 0.31 | -0.42 | -0.49 | -0.61 | 0.14 | 0.01 | -0.62 | 0.24 | 0.05 | -0.24 | 0.07 | -0.37 | -0.26 | -0.24 |
| Nickel and articles thereof | '75 | -0.63 | 0.09 | -0.50 | 0.02 | 0.81 | -1.00 | 0.59 | -0.85 | -0.96 | -0.85 | -0.06 | -0.76 | -0.03 | -0.53 | -0.45 | -0.95 | -0.89 | -0.69 | 0.34 | 0.15 | -0.84 |
| Aluminium and articles thereof | '76 | -0.22 | 0.22 | -0.48 | -0.06 | 0.61 | -0.65 | -0.61 | 0.21 | 0.09 | 0.24 | -0.67 | 0.15 | 0.02 | -0.17 | -0.35 | -0.11 | -0.14 | 0.31 | -0.22 | -0.17 | -0.31 |
| Lead and articles thereof | '78 | 0.55 | 0.31 | 0.10 | -0.27 | -0.89 | -0.34 | 0.01 | 0.31 | -0.04 | 0.12 | -0.63 | -0.51 | 0.44 | -0.91 | -0.31 | -0.66 | -0.03 | -0.51 | 0.47 | -0.62 | -0.11 |
| Zinc and articles thereof | '79 | 0.47 | -0.27 | 0.18 | 0.32 | 0.72 | -0.67 | -0.92 | 0.11 | -0.71 | -0.91 | -0.82 | 0.67 | -0.19 | -0.80 | -0.36 | -0.62 | -0.47 | -0.88 | -0.52 | -0.62 | -0.86 |
| Tin and articles thereof | '80 | -0.57 | 0.75 | -0.70 | -0.13 | -0.98 | -1.00 | 0.00 | 0.25 | -0.05 | -0.70 | 0.48 | 0.17 | -0.97 | -0.93 | 0.25 | 0.51 | 0.10 | -0.89 | -0.75 | -0.46 | -0.68 |
| Other base metals; cermets; articles thereof | '81 | -0.39 | -0.57 | -0.60 | -0.37 | 0.33 | -0.99 | -0.72 | -0.74 | -0.94 | -0.41 | -0.65 | -0.71 | -0.48 | -0.47 | -0.24 | -0.63 | -0.75 | -0.64 | 0.32 | 0.29 | -0.80 |
| Tools, implements, cutlery, spoons and forks, of base metal | '82 | 0.06 | -0.70 | -0.16 | -0.12 | -0.58 | 0.21 | -0.83 | 0.29 | -0.04 | -0.45 | -0.20 | -0.04 | 0.36 | 0.22 | 0.40 | -0.27 | -0.71 | -0.39 | -0.27 | -0.11 | -0.15 |
| Miscellaneous articles of base metal | '83 | -0.18 | -0.55 | 0.17 | -0.29 | -0.74 | -0.90 | -0.20 | 0.37 | 0.21 | 0.32 | -0.57 | 0.11 | -0.09 | -0.55 | 0.10 | -0.08 | -0.30 | 0.15 | -0.21 | -0.10 | -0.38 |
| Machinery, mechanical appliances, nuclear reactors, boilers | '84 | 0.01 | -0.16 | 0.19 | 0.07 | -0.41 | -0.80 | 0.11 | 0.06 | -0.34 | -0.02 | 0.12 | -0.20 | 0.12 | -0.27 | 0.02 | 0.15 | -0.53 | -0.11 | 0.12 | 0.03 | -0.40 |
| Electrical machinery and equipment; sound recorders and reproducers, tv | '85 | 0.29 | 0.38 | 0.09 | -0.15 | -0.65 | -0.96 | 0.54 | -0.19 | -0.31 | 0.10 | 0.36 | -0.42 | -0.25 | -0.59 | 0.49 | -0.03 | 0.28 | -0.49 | -0.42 | -0.15 | 0.44 |
| Railway or tramway locomotives; railway or tramway track | '86 | -0.44 | -0.74 | 0.59 | -0.68 | -0.56 | -0.90 | -0.96 | 0.54 | -0.91 | 0.11 | -0.76 | 0.57 | -0.11 | 0.21 | -0.94 | -0.71 | -0.72 | -0.37 | -0.56 | 0.01 | -0.99 |
| Vehicles other than railway or tramway rolling stock | '87 | 0.15 | -0.81 | 0.53 | -0.29 | -0.77 | -0.94 | -0.69 | 0.18 | 0.29 | 0.36 | -0.80 | 0.37 | 0.28 | -0.81 | -0.46 | 0.18 | -0.41 | 0.30 | 0.13 | 0.01 | -0.75 |
| Aircraft, spacecraft, and parts thereof | '88 | -0.62 | -0.36 | -0.82 | -0.64 | -0.46 | -0.71 | -0.25 | -0.59 | -0.23 | -0.53 | 0.06 | 0.10 | -0.64 | -0.47 | -0.86 | -0.69 | 0.22 | -0.50 | 0.36 | 0.65 | -0.76 |
| Ships, boats and floating structures | '89 | 0.67 | -0.73 | -0.58 | 0.06 | 0.32 | -0.19 | 0.09 | 0.28 | -0.60 | -0.02 | -0.52 | -0.43 | -0.46 | -0.96 | -0.66 | -0.04 | -0.15 | -0.05 | -0.19 | -0.63 | -0.49 |
| Optical, photographic, cinematographic, medical or surgical instruments | '90 | 0.05 | 0.10 | 0.13 | 0.30 | -0.29 | -0.21 | -0.04 | -0.26 | -0.12 | 0.06 | 0.23 | -0.50 | -0.14 | 0.22 | 0.16 | -0.20 | 0.11 | -0.72 | 0.11 | 0.26 | -0.23 |
| Clocks and watches and parts thereof | '91 | -0.91 | -0.46 | -0.83 | -0.58 | -0.92 | -0.98 | 0.04 | -0.72 | -0.07 | -0.77 | 0.23 | -0.38 | -0.60 | 0.92 | -0.77 | 0.17 | -0.11 | -0.85 | -0.10 | -0.47 | -0.87 |
| Musical instruments; parts and accessories of such articles | '92 | -0.29 | 0.38 | -0.28 | 0.04 | -0.93 | -0.35 | -0.73 | -0.46 | -0.43 | -0.32 | -0.74 | -0.43 | 0.11 | -0.65 | 0.17 | -0.62 | -0.01 | -0.71 | -0.02 | 0.13 | -0.57 |
| Arms and ammunition; parts and accessories thereof | '93 | 0.02 | -0.99 | -0.75 | -0.86 | 0.43 | -0.17 | -0.38 | -0.34 | -0.03 | -0.18 | -0.99 | 0.23 | -0.06 | 0.00 | -0.49 | 0.79 | -1.00 | 0.43 | 0.36 | 0.61 | -1.00 |
| Furniture; bedding, mattresses, stuffed furnishings; lamps | '94 | -0.65 | -0.07 | 0.23 | -0.24 | -0.48 | -0.51 | -0.32 | 0.61 | 0.40 | 0.47 | -0.90 | -0.05 | 0.10 | -0.73 | -0.39 | -0.43 | -0.02 | 0.18 | -0.23 | -0.38 | 0.45 |
| Toys, games and sports requisites | '95 | -0.80 | -0.57 | -0.33 | -0.09 | -0.82 | 0.24 | -0.46 | 0.23 | -0.69 | -0.38 | -0.64 | 0.03 | -0.30 | -0.85 | 0.03 | -0.35 | -0.15 | -0.78 | -0.15 | -0.25 | 0.07 |
| Miscellaneous manufactured articles | '96 | -0.48 | -0.32 | -0.01 | -0.04 | -0.94 | -0.57 | -0.19 | 0.35 | -0.19 | -0.33 | -0.62 | -0.06 | 0.18 | -0.33 | -0.22 | -0.03 | 0.44 | 0.29 | -0.34 | -0.31 | -0.17 |
| Works of art, collectors' pieces and antiques | '97 | -0.86 | -0.99 | -0.99 | -0.78 | -0.82 | -0.97 | -0.93 | -0.96 | -0.87 | -0.98 | -0.42 | -0.68 | -0.82 | 0.47 | -0.97 | -0.98 | -1.00 | -0.92 | 0.85 | 0.59 | -0.98 |
| Commodities not elsewhere specified | '99 | -1.00 | -0.82 | -0.31 | -0.75 | 0.20 | -0.99 | -1.00 | -0.90 | -0.91 | -0.59 | 0.27 | 0.33 | 0.10 | -1.00 | -0.72 | -1.00 | -1.00 | -0.88 | -0.15 | -0.03 | -0.29 |

**Table S 5. Lafay Index. 2001**

| **Product label** | **Code** | **Bang** | **Belg** | **Brazil** | **Bulg** | **Camb** | **Chile** | **Chin** | **Col** | **Czech** | **Eth** | **Fin** | **Fran** | **Germ** | **Gre** | **Hon** | **Hung** | **India** | **Indo** | **Irel** | **Italy** | **Jap** |
| --- | --- | --- | --- | --- | --- | --- | --- | --- | --- | --- | --- | --- | --- | --- | --- | --- | --- | --- | --- | --- | --- | --- |
| Dairy products | '04 | -0.51 | -0.05 | -0.13 | 0.18 | -0.02 | 0.08 | -0.01 | 0.11 | 0.21 | -0.03 | 0.15 | 0.31 | 0.05 | -0.16 | -0.10 | 0.15 | 0.09 | -0.37 | 0.35 | -0.36 | -0.13 |
| Products of animal origin | '05 | -0.01 | -0.01 | 0.04 | 0.00 | 0.00 | -0.01 | 0.09 | -0.02 | -0.03 | 0.07 | -0.02 | -0.02 | -0.02 | -0.01 | 0.00 | 0.09 | 0.04 | -0.02 | 0.00 | -0.02 | -0.09 |
| Lac; gums, resins | '13 | -0.01 | -0.01 | -0.01 | -0.01 | -0.01 | 0.19 | 0.01 | -0.04 | 0.00 | 0.27 | -0.02 | 0.01 | 0.00 | 0.02 | 0.00 | -0.01 | 0.27 | -0.01 | 0.02 | 0.00 | -0.03 |
| Vegetable plaiting materials | '14 | 0.01 | 0.00 | 0.01 | 0.00 | 0.01 | 0.02 | -0.01 | 0.00 | 0.00 | 5.54 | 0.00 | 0.00 | 0.00 | 0.00 | 0.00 | 0.01 | 0.02 | 0.03 | 0.00 | -0.01 | -0.01 |
| Animal or vegetable fats and oils | '15 | -3.30 | 0.02 | 0.49 | -0.08 | -0.23 | -0.39 | -0.14 | -0.17 | -0.05 | -0.23 | -0.05 | -0.08 | -0.01 | 0.92 | -0.09 | 0.00 | -1.26 | 1.17 | -0.10 | -0.11 | -0.07 |
| Preparations of meat, fish, molluscs | '16 | 0.00 | 0.01 | 0.34 | 0.02 | -0.01 | 0.72 | 0.39 | -0.12 | -0.03 | 0.00 | -0.09 | -0.05 | -0.05 | -0.01 | -0.07 | 0.15 | 0.01 | 0.09 | 0.05 | -0.07 | -0.48 |
| Sugars and sugar confectionery | '17 | -0.52 | -0.02 | 2.40 | -0.37 | -0.95 | -0.20 | -0.05 | 1.51 | 0.05 | 0.12 | -0.07 | 0.20 | 0.01 | 0.04 | -0.02 | 0.01 | 0.44 | -0.37 | -0.09 | -0.06 | -0.08 |
| Cocoa and cocoa preparations | '18 | -0.01 | 0.14 | 0.12 | -0.07 | 0.00 | -0.03 | -0.01 | 0.08 | -0.06 | 0.00 | -0.04 | -0.05 | -0.03 | -0.05 | -0.01 | -0.03 | -0.01 | 0.26 | -0.04 | -0.01 | -0.07 |
| Preparations of cereals, flour | '19 | -0.08 | 0.16 | 0.02 | 0.06 | -0.24 | 0.02 | 0.06 | -0.03 | -0.07 | -0.25 | -0.19 | 0.00 | 0.01 | 0.08 | -0.04 | -0.02 | 0.04 | 0.04 | 0.13 | 0.30 | -0.10 |
| Preparations of vegetables, fruit, nuts | '20 | -0.02 | 0.09 | 0.83 | 0.17 | -0.02 | 0.74 | 0.27 | -0.02 | -0.07 | -0.02 | -0.15 | -0.11 | -0.16 | 1.79 | -0.03 | 0.41 | 0.08 | 0.08 | -0.15 | 0.19 | -0.32 |
| Miscellaneous edible preparations | '21 | -0.06 | 0.05 | 0.40 | -0.18 | -0.06 | 0.42 | 0.04 | 0.25 | -0.13 | -0.05 | -0.17 | 0.08 | -0.01 | -0.03 | -0.04 | -0.02 | 0.12 | -0.08 | 0.26 | 0.05 | -0.10 |
| Beverages, spirits and vinegar | '22 | -0.03 | -0.12 | -0.02 | 0.64 | -0.15 | 2.06 | 0.08 | -0.18 | 0.08 | -0.02 | -0.21 | 1.12 | -0.21 | 0.08 | -0.13 | 0.12 | 0.03 | 0.00 | -0.08 | 0.49 | -0.32 |
| Residues and waste from food industries | '23 | -0.47 | 0.00 | 2.11 | 0.00 | -0.02 | 0.51 | -0.08 | -0.63 | -0.18 | 0.01 | -0.16 | -0.06 | -0.05 | -0.16 | -0.01 | -0.15 | 0.54 | -0.84 | -0.27 | -0.23 | -0.22 |
| Tobacco and manufactured tobacco substitutes | '24 | -0.03 | -0.02 | 0.93 | 0.40 | -2.54 | 0.03 | 0.02 | -0.03 | 0.00 | -0.04 | -0.12 | -0.24 | 0.03 | 1.14 | 0.02 | -0.04 | 0.22 | -0.11 | -0.01 | -0.27 | -0.37 |
| Salt; sulphur; earths and stone; lime and cement | '25 | -1.51 | 0.01 | 0.11 | 0.33 | -1.18 | 0.01 | 0.10 | 0.30 | 0.01 | -0.06 | -0.36 | -0.01 | -0.04 | 0.96 | -0.05 | -0.06 | 0.21 | -0.15 | -0.07 | -0.19 | -0.18 |
| Ores, slag and ash | '26 | -0.01 | -0.16 | 2.89 | -1.78 | 0.00 | 8.49 | -0.87 | -0.04 | -0.27 | 0.20 | -1.11 | -0.12 | -0.27 | 0.10 | 0.00 | -0.09 | 0.25 | 1.41 | -0.05 | -0.19 | -1.02 |
| Mineral fuels, mineral oils and products of their distillation | '27 | -2.45 | -2.21 | -5.11 | 1.95 | -6.98 | -7.88 | -2.08 | 20.84 | -3.09 | -3.06 | -4.36 | -3.62 | -3.98 | -1.39 | -0.90 | -3.12 | -13.1 | 3.48 | -1.81 | -4.07 | -10.9 |
| Inorganic chemicals | '28 | -0.33 | -0.14 | -0.07 | 0.19 | -0.09 | 0.57 | 0.21 | -0.46 | -0.18 | -0.09 | -0.22 | 0.04 | 0.00 | 0.07 | -0.04 | 0.09 | -0.81 | -0.66 | 0.09 | -0.07 | -0.23 |
| Organic chemicals | '29 | -0.96 | -0.27 | -2.23 | 0.34 | -0.62 | 0.43 | -1.01 | -3.22 | 0.33 | -0.10 | -0.65 | -0.22 | -0.63 | -0.52 | -0.08 | 0.05 | 0.31 | -2.65 | 8.07 | -0.89 | 0.11 |
| Pharmaceutical products | '30 | -0.51 | 0.27 | -1.18 | -0.69 | -1.59 | -0.75 | -0.07 | -0.55 | -0.87 | -0.36 | -0.86 | 0.89 | 0.53 | -0.31 | -0.04 | -0.20 | 1.19 | -0.06 | 2.92 | -0.03 | -0.42 |
| Fertilisers | '31 | -0.42 | 0.09 | -1.08 | 0.82 | -0.16 | -0.03 | -0.25 | -0.75 | -0.04 | -0.43 | 0.01 | -0.14 | 0.01 | 0.01 | 0.00 | -0.10 | -0.41 | -0.20 | -0.17 | -0.09 | -0.07 |
| Tanning or dyeing extracts; paints and varnishes | '32 | -0.49 | 0.14 | -0.27 | -0.38 | -0.10 | -0.65 | -0.14 | 0.84 | -0.24 | -0.10 | 0.11 | -0.02 | 0.27 | -0.08 | -0.06 | -0.26 | 0.43 | -0.55 | -0.19 | -0.12 | 0.12 |
| Essential oils; perfumery, cosmetics | '33 | -0.12 | -0.01 | -0.03 | 0.18 | -0.08 | -0.26 | 0.04 | -0.32 | -0.20 | -0.09 | -0.18 | 0.90 | 0.03 | -0.02 | -0.11 | -0.19 | 0.18 | -0.16 | 1.09 | 0.00 | -0.14 |
| Soap, washing preparations, waxes, candles | '34 | -0.09 | 0.10 | -0.06 | -0.35 | -0.21 | -0.27 | -0.02 | 0.00 | 0.10 | -0.16 | -0.13 | -0.01 | 0.10 | -0.04 | 0.00 | -0.03 | -0.04 | -0.18 | -0.17 | 0.05 | 0.02 |
| Albuminoidal substances | '35 | -0.03 | 0.02 | 0.02 | -0.05 | -0.08 | -0.11 | -0.06 | -0.03 | -0.06 | -0.03 | -0.03 | 0.05 | 0.00 | -0.05 | -0.01 | -0.05 | 0.04 | -0.19 | 0.11 | -0.04 | -0.06 |
| Explosives; pyrotechnic products; matches | '36 | -0.01 | 0.00 | 0.01 | 0.01 | -0.01 | 0.03 | 0.05 | -0.03 | 0.03 | -0.07 | -0.01 | 0.01 | 0.00 | -0.03 | 0.00 | 0.01 | 0.01 | -0.03 | 0.00 | -0.01 | -0.01 |
| Photographic or cinematographic goods | '37 | -0.11 | 0.24 | -0.10 | 0.14 | -0.03 | -0.19 | -0.02 | -0.28 | -0.06 | -0.04 | -0.10 | 0.00 | 0.01 | 0.08 | -0.09 | -0.09 | -0.16 | -0.09 | -0.07 | -0.07 | 0.38 |
| Miscellaneous chemical products | '38 | -0.44 | 0.22 | -0.44 | -0.65 | -0.12 | -0.76 | -0.29 | -0.12 | -0.33 | -0.27 | -0.26 | 0.15 | 0.29 | -0.12 | -0.01 | -0.22 | 0.10 | -0.44 | 0.78 | -0.16 | 0.07 |
| Plastics and articles thereof | '39 | -1.52 | 1.31 | -0.93 | -0.91 | -0.96 | -1.70 | -1.94 | -0.39 | -1.04 | -0.46 | -0.52 | -0.14 | 0.65 | 0.17 | 0.13 | -0.42 | 0.29 | -0.85 | -0.93 | -0.06 | 0.35 |
| Rubber and articles thereof | '40 | -0.31 | -0.19 | -0.07 | -0.11 | 0.51 | -0.58 | -0.12 | -0.75 | 0.24 | -0.50 | -0.20 | 0.21 | -0.08 | -0.13 | -0.06 | -0.05 | 0.18 | 0.52 | -0.16 | -0.05 | 0.42 |
| Raw hides and skins (other than furskins) and leather | '41 | 2.21 | 0.00 | 0.72 | -0.38 | -0.32 | 0.05 | -0.50 | 0.43 | -0.11 | 7.43 | -0.02 | 0.02 | 0.00 | 0.17 | -0.10 | -0.31 | 0.38 | -0.18 | 0.08 | -0.01 | -0.04 |
| Articles of leather; saddlery; travel goods, handbags | '42 | 0.02 | -0.01 | 0.02 | 0.21 | 0.02 | -0.22 | 1.34 | 0.19 | -0.07 | -0.02 | -0.11 | 0.09 | -0.14 | -0.12 | 0.33 | 0.06 | 1.16 | 0.26 | -0.10 | 0.27 | -0.56 |
| Furskins and artificial fur; manufactures thereof | '43 | 0.00 | 0.00 | 0.01 | 0.01 | 0.00 | 0.00 | 0.06 | 0.00 | 0.01 | 0.02 | 0.24 | 0.00 | 0.00 | 0.68 | -0.01 | 0.01 | 0.00 | 0.00 | 0.00 | -0.01 | -0.04 |
| Wood and articles of wood | '44 | -0.24 | 0.01 | 1.46 | 0.74 | 0.76 | 3.61 | -0.29 | 0.04 | 0.63 | -0.08 | 1.46 | -0.08 | -0.10 | -0.30 | -0.02 | 0.19 | -0.52 | 2.66 | -0.33 | -0.48 | -1.55 |
| Cork and articles of cork | '45 | 0.00 | 0.00 | 0.00 | -0.02 | 0.00 | -0.10 | 0.00 | 0.00 | -0.01 | 0.00 | 0.00 | -0.03 | -0.01 | -0.01 | 0.00 | -0.01 | 0.00 | 0.00 | 0.00 | -0.01 | 0.00 |
| Manufactures of straw, basketware | '46 | 0.02 | 0.00 | 0.00 | 0.00 | -0.01 | -0.01 | 0.11 | 0.00 | 0.00 | 0.01 | -0.01 | -0.01 | -0.01 | -0.01 | 0.01 | 0.00 | 0.00 | 0.06 | 0.00 | -0.01 | -0.05 |
| Pulp of wood or of other fibrous cellulosic material | '47 | -0.20 | -0.05 | 1.09 | 0.11 | 0.00 | 3.47 | -0.58 | -0.31 | 0.09 | -0.04 | 0.73 | -0.15 | -0.22 | -0.05 | 0.01 | -0.11 | -0.28 | -0.63 | -0.02 | -0.37 | -0.20 |
| Paper and paperboard; articles of paper pulp, of paper or of paperboard | '48 | -1.21 | -0.06 | 0.40 | -0.99 | -1.39 | -0.27 | -0.49 | -0.09 | -0.20 | -0.34 | 8.54 | -0.16 | 0.07 | -0.70 | -0.08 | -0.36 | -0.24 | 1.22 | -0.65 | -0.01 | -0.06 |
| Printed books, newspapers, pictures and products of the printing industry | '49 | -0.06 | 0.02 | -0.15 | -0.08 | 6.81 | 0.05 | 0.02 | 0.46 | 0.08 | -0.09 | 0.13 | 0.01 | 0.12 | 0.03 | 0.10 | -0.09 | -0.16 | -0.01 | -0.17 | 0.15 | -0.08 |
| Silk | '50 | 0.09 | 0.00 | 0.04 | -0.02 | 0.00 | 0.00 | 0.14 | 0.00 | 0.00 | 0.00 | 0.00 | 0.00 | 0.00 | -0.01 | -0.01 | -0.01 | 0.19 | 0.00 | 0.00 | 0.01 | -0.02 |
| Wool, fine or coarse animal hair; horsehair yarn and woven fabric | '51 | -0.05 | -0.01 | 0.01 | -0.56 | -0.01 | 0.03 | -0.19 | -0.10 | -0.01 | 0.00 | -0.02 | 0.01 | -0.01 | 0.00 | -0.05 | -0.12 | -0.07 | -0.04 | -0.03 | 0.21 | -0.05 |
| Cotton | '52 | -5.86 | 0.04 | 0.30 | -1.05 | -0.31 | -0.10 | 0.09 | -0.79 | -0.04 | 0.88 | -0.05 | 0.02 | -0.02 | 1.48 | 0.19 | -0.15 | 2.31 | -1.35 | -0.05 | 0.10 | -0.04 |
| Other vegetable textile fibres; paper yarn and woven fabrics of paper yarn | '53 | 1.67 | 0.01 | 0.01 | -0.16 | 0.00 | 0.00 | 0.03 | 0.00 | 0.03 | -0.02 | 0.00 | 0.03 | 0.00 | -0.01 | 0.00 | 0.00 | 0.16 | -0.02 | 0.00 | 0.01 | -0.01 |
| Man-made filaments; strip and the like of man-made textile materials | '54 | -1.57 | 0.01 | -0.42 | -0.63 | -0.43 | -0.13 | -0.39 | -0.57 | -0.14 | -0.31 | -0.09 | -0.03 | 0.01 | -0.10 | -0.10 | -0.08 | 0.43 | 0.45 | 0.03 | 0.04 | 0.24 |
| Man-made staple fibres | '55 | -3.46 | 0.00 | -0.07 | -0.85 | -9.23 | -0.23 | -0.11 | -0.23 | -0.06 | -0.09 | -0.01 | 0.02 | 0.04 | -0.12 | -0.02 | -0.19 | 0.50 | 0.21 | 0.03 | 0.07 | 0.12 |
| Wadding, felt and nonwovens; special yarns; twine, cordage, ropes | '56 | 0.27 | -0.02 | 0.00 | -0.16 | -0.60 | -0.13 | -0.02 | -0.05 | -0.01 | -0.01 | 0.01 | 0.00 | 0.08 | 0.01 | 0.00 | -0.01 | 0.02 | -0.05 | -0.04 | 0.10 | 0.02 |
| Carpets and other textile floor coverings | '57 | 0.00 | 0.47 | 0.00 | 0.04 | -0.01 | -0.07 | 0.09 | -0.01 | -0.04 | -0.01 | -0.05 | -0.02 | -0.09 | 0.06 | -0.01 | -0.04 | 0.74 | 0.02 | -0.05 | -0.02 | -0.06 |
| Special woven fabrics; tufted textile fabrics; lace; tapestries | '58 | -0.07 | 0.08 | 0.00 | -0.24 | -0.67 | -0.04 | 0.02 | 0.00 | 0.02 | -0.01 | -0.01 | 0.04 | 0.02 | 0.00 | 0.04 | -0.12 | 0.23 | -0.03 | -0.02 | 0.02 | 0.01 |
| Impregnated, coated, covered or laminated textile fabrics | '59 | -0.26 | 0.07 | -0.06 | -0.32 | -0.07 | -0.13 | -0.16 | 0.08 | -0.09 | -0.04 | -0.05 | 0.00 | 0.05 | -0.03 | -0.01 | -0.14 | -0.09 | -0.16 | -0.02 | 0.09 | 0.03 |
| Knitted or crocheted fabrics | '60 | -0.34 | 0.00 | -0.01 | -0.45 | -5.42 | -0.02 | -0.02 | 0.11 | -0.07 | 0.01 | -0.01 | 0.03 | 0.04 | 0.11 | 0.04 | -0.17 | 0.00 | -0.14 | -0.03 | 0.10 | 0.03 |
| Articles of apparel and clothing accessories, knitted or crocheted | '61 | 12.15 | -0.13 | 0.12 | 2.41 | 36.48 | -0.62 | 2.51 | 0.93 | 0.02 | -0.06 | -0.44 | -0.47 | -0.63 | 3.26 | 0.93 | 0.42 | 2.31 | 1.30 | -0.49 | 0.53 | -1.21 |
| Articles of apparel and clothing accessories, not knitted or crocheted | '62 | 25.01 | -0.15 | 0.02 | 5.48 | 0.23 | -0.70 | 3.52 | 1.55 | 0.27 | -0.17 | -0.55 | -0.54 | -0.75 | 0.10 | 1.04 | 0.93 | 4.15 | 2.33 | -0.49 | 0.84 | -1.60 |
| Other made-up textile articles; sets; worn clothing | '63 | 1.85 | 0.01 | 0.24 | 0.31 | -0.83 | -0.20 | 0.71 | 0.16 | 0.29 | -0.17 | -0.11 | -0.10 | -0.10 | 0.17 | 0.02 | 0.06 | 1.34 | 0.19 | -0.09 | 0.01 | -0.26 |
| Footwear, gaiters and the like | '64 | 0.29 | 0.06 | 1.66 | 1.33 | 0.75 | -0.56 | 1.89 | -0.17 | -0.01 | -0.12 | -0.18 | -0.35 | -0.34 | -0.23 | 0.25 | 0.31 | 0.80 | 1.17 | -0.22 | 0.97 | -0.48 |
| Headgear and parts thereof | '65 | 0.36 | 0.00 | 0.00 | 0.01 | 0.41 | -0.03 | 0.12 | 0.00 | 0.02 | -0.01 | -0.03 | -0.02 | -0.01 | -0.02 | 0.04 | 0.00 | 0.01 | 0.03 | -0.01 | 0.03 | -0.03 |
| Umbrellas, sun umbrellas, walking sticks | '66 | 0.00 | 0.00 | -0.01 | -0.01 | 0.00 | -0.01 | 0.12 | 0.00 | 0.00 | -0.02 | -0.01 | -0.01 | -0.01 | 0.00 | 0.02 | 0.00 | -0.01 | 0.01 | 0.00 | 0.00 | -0.03 |
| Prepared feathers and down; artificial flowers; articles of human hair | '67 | 0.00 | 0.00 | 0.00 | 0.00 | -0.01 | -0.01 | 0.16 | -0.01 | 0.00 | 0.00 | -0.01 | -0.01 | -0.01 | -0.01 | 0.10 | 0.00 | 0.06 | 0.04 | 0.00 | -0.01 | -0.02 |
| Articles of stone, plaster, cement, mica or similar materials | '68 | -0.10 | 0.08 | 0.23 | -0.06 | -0.13 | -0.10 | 0.16 | 0.04 | 0.09 | -0.03 | 0.06 | -0.02 | -0.02 | 0.34 | -0.05 | -0.09 | 0.33 | 0.03 | -0.14 | 0.41 | -0.06 |
| Ceramic products | '69 | 0.00 | -0.04 | 0.20 | 0.25 | -0.34 | -0.19 | 0.31 | 0.16 | 0.25 | -0.04 | -0.10 | -0.07 | -0.03 | -0.09 | -0.03 | -0.01 | 0.06 | 0.06 | -0.10 | 0.71 | 0.04 |
| Glass and glassware | '70 | -0.19 | 0.16 | -0.01 | 0.03 | -0.14 | -0.15 | -0.05 | 0.07 | 1.03 | -0.10 | 0.07 | 0.11 | 0.02 | -0.15 | -0.07 | 0.00 | 0.04 | 0.17 | -0.04 | 0.06 | 0.11 |
| Natural or cultured pearls, precious stones, imitation jewellery, coin | '71 | 0.00 | 0.03 | 0.32 | 0.00 | 0.01 | 1.14 | 0.26 | 0.59 | 0.08 | 0.46 | -0.02 | -0.08 | -0.17 | -0.01 | -0.33 | -0.01 | -0.71 | 0.48 | -0.19 | -0.12 | -0.72 |
| Iron and steel | '72 | -2.10 | 0.76 | 2.44 | 2.64 | -0.69 | -0.85 | -1.89 | -0.03 | -0.01 | -0.78 | 0.38 | 0.16 | -0.04 | -0.46 | -0.19 | -0.27 | 0.12 | -1.42 | -0.33 | -0.87 | 1.04 |
| Articles of iron or steel | '73 | -0.68 | -0.04 | 0.06 | 0.06 | -0.62 | -0.56 | 0.73 | -0.12 | 1.27 | -0.50 | -0.08 | 0.08 | 0.16 | 0.30 | -0.03 | -0.36 | 1.00 | -0.57 | -0.46 | 1.03 | 0.36 |
| Copper and articles thereof | '74 | -0.10 | 0.07 | -0.21 | 2.91 | -0.01 | 15.97 | -0.92 | -0.22 | -0.25 | -0.07 | 0.24 | -0.07 | 0.02 | 0.42 | -0.01 | -0.15 | -0.03 | 0.15 | -0.11 | -0.33 | 0.16 |
| Nickel and articles thereof | '75 | 0.00 | -0.04 | 0.03 | -0.01 | 0.00 | -0.01 | -0.07 | 0.01 | -0.03 | 0.00 | 0.01 | -0.05 | -0.04 | -0.01 | -0.01 | -0.01 | -0.13 | 0.12 | -0.02 | -0.09 | -0.10 |
| Aluminium and articles thereof | '76 | -0.28 | -0.08 | 0.76 | -0.15 | -0.37 | -0.86 | -0.19 | -0.12 | -0.24 | -0.09 | -0.27 | -0.14 | -0.13 | 2.04 | -0.03 | 0.15 | 0.12 | -0.13 | -0.18 | -0.13 | -0.60 |
| Lead and articles thereof | '78 | -0.03 | 0.01 | -0.04 | 0.36 | 0.00 | -0.01 | 0.04 | -0.01 | -0.04 | 0.00 | 0.00 | 0.00 | 0.00 | 0.01 | 0.00 | -0.01 | -0.04 | -0.03 | -0.02 | -0.01 | 0.00 |
| Zinc and articles thereof | '79 | -0.45 | -0.02 | -0.01 | 0.75 | -0.03 | -0.05 | 0.07 | -0.09 | -0.04 | -0.01 | 0.19 | 0.00 | -0.03 | -0.03 | 0.00 | -0.04 | -0.11 | -0.15 | -0.01 | -0.05 | 0.00 |
| Tin and articles thereof | '80 | -0.01 | 0.01 | 0.02 | -0.01 | 0.00 | -0.01 | 0.04 | -0.01 | -0.01 | 0.00 | 0.00 | -0.01 | -0.01 | -0.01 | 0.00 | -0.01 | -0.01 | 0.16 | 0.00 | -0.01 | -0.01 |
| Other base metals; cermets; articles thereof | '81 | -0.01 | 0.00 | -0.03 | -0.01 | 0.00 | 0.05 | 0.10 | -0.01 | -0.09 | 0.00 | 0.08 | 0.00 | -0.03 | -0.01 | 0.00 | -0.01 | -0.04 | -0.01 | -0.02 | -0.02 | -0.04 |
| Tools, implements, cutlery, spoons and forks, of base metal | '82 | -0.05 | -0.04 | -0.04 | 0.00 | -0.10 | -0.31 | 0.34 | -0.14 | 0.00 | -0.08 | -0.17 | -0.07 | 0.08 | -0.10 | 0.03 | -0.12 | 0.19 | -0.09 | -0.09 | -0.04 | 0.16 |
| Miscellaneous articles of base metal | '83 | -0.07 | -0.12 | -0.01 | -0.09 | -0.18 | -0.14 | 0.23 | -0.09 | 0.05 | -0.08 | -0.11 | 0.01 | 0.12 | -0.02 | 0.02 | -0.12 | 0.12 | -0.09 | -0.09 | 0.31 | 0.00 |
| Machinery, mechanical appliances, nuclear reactors, boilers | '84 | -5.12 | -0.87 | -4.76 | -3.01 | -2.22 | -7.73 | -2.08 | -6.90 | -1.32 | -1.63 | -1.33 | -0.99 | 1.78 | -1.94 | -0.90 | 2.33 | -2.22 | -4.99 | -0.41 | 4.32 | 4.63 |
| Electrical machinery and equipment; sound recorders and reproducers, tv | '85 | -2.52 | -0.48 | -5.78 | -2.66 | -1.74 | -4.81 | -1.89 | -4.24 | -0.45 | -1.12 | 2.70 | 0.68 | -0.61 | -0.76 | -0.66 | 0.67 | -1.29 | 2.84 | -1.06 | -0.98 | 4.53 |
| Railway or tramway locomotives; railway or tramway track | '86 | -0.03 | -0.03 | -0.04 | 0.01 | -0.01 | -0.09 | 0.39 | -0.16 | 0.31 | -0.01 | -0.08 | 0.06 | 0.05 | -0.68 | -0.05 | 0.13 | 0.02 | -0.03 | -0.02 | 0.03 | 0.05 |
| Vehicles other than railway or tramway rolling stock | '87 | -1.64 | 1.15 | 0.92 | -4.66 | -2.10 | -3.71 | -0.03 | -0.93 | 3.94 | -1.63 | -1.51 | 1.40 | 4.47 | -2.80 | -0.34 | 0.90 | 0.87 | -2.50 | -2.56 | -2.10 | 8.95 |
| Aircraft, spacecraft, and parts thereof | '88 | -0.11 | 0.02 | 2.85 | -0.33 | -0.01 | 0.09 | -0.89 | -3.46 | -0.06 | -0.19 | -0.36 | 2.01 | 0.02 | -0.24 | -0.33 | 0.00 | -0.18 | -0.10 | -0.75 | -0.21 | -0.09 |
| Ships, boats and floating structures | '89 | -1.71 | -0.01 | 0.00 | -0.01 | 0.04 | -0.05 | 0.23 | -0.04 | 0.02 | 0.00 | 1.52 | 0.25 | 0.11 | -1.42 | -0.01 | 0.00 | -0.29 | -1.43 | -0.03 | 0.26 | 1.05 |
| Optical, photographic, cinematographic, medical or surgical instruments | '90 | -0.35 | -0.36 | -1.53 | -0.80 | -0.49 | -1.00 | -0.82 | -1.31 | -0.58 | -0.30 | -0.13 | -0.21 | 0.38 | -0.65 | 0.45 | -0.20 | -0.76 | -0.25 | 0.35 | -0.47 | 1.38 |
| Clocks and watches and parts thereof | '91 | -0.02 | -0.02 | -0.07 | 0.00 | 0.00 | -0.08 | 0.14 | -0.07 | -0.03 | 0.00 | -0.04 | -0.04 | -0.04 | -0.09 | 0.39 | -0.02 | 0.04 | 0.00 | -0.03 | -0.10 | -0.13 |
| Musical instruments; parts and accessories of such articles | '92 | 0.00 | 0.00 | -0.02 | 0.00 | 0.02 | -0.02 | 0.06 | -0.02 | 0.07 | 0.00 | -0.02 | -0.01 | 0.00 | -0.02 | 0.00 | -0.01 | 0.01 | 0.15 | -0.01 | 0.01 | 0.02 |
| Arms and ammunition; parts and accessories thereof | '93 | -0.10 | 0.06 | 0.05 | 0.00 | 0.00 | -0.01 | 0.00 | -0.16 | 0.07 | -0.04 | -0.01 | 0.03 | 0.00 | -0.01 | 0.00 | 0.01 | 0.01 | -0.03 | 0.00 | 0.05 | -0.02 |
| Furniture; bedding, mattresses, stuffed furnishings; lamps | '94 | -0.12 | -0.08 | 0.35 | 0.42 | -0.12 | -0.12 | 1.40 | 0.16 | 1.16 | -0.18 | -0.14 | -0.27 | -0.32 | -0.26 | 0.04 | 0.78 | 0.02 | 1.19 | -0.34 | 1.80 | -0.63 |
| Toys, games and sports requisites | '95 | -0.03 | -0.08 | -0.05 | 0.06 | -0.06 | -0.37 | 1.71 | -0.22 | 0.07 | -0.01 | -0.16 | -0.22 | -0.16 | -0.13 | 1.17 | 0.55 | 0.05 | 0.11 | -0.14 | -0.03 | -0.20 |
| Miscellaneous manufactured articles | '96 | -0.44 | 0.00 | 0.02 | -0.20 | -0.89 | -0.14 | 0.24 | -0.05 | 0.07 | -0.05 | -0.07 | 0.00 | 0.04 | -0.03 | 0.09 | -0.07 | 0.09 | 0.00 | -0.01 | 0.04 | 0.10 |
| Works of art, collectors' pieces and antiques | '97 | 0.00 | 0.00 | 0.00 | 0.00 | 0.00 | -0.01 | 0.00 | 0.00 | 0.01 | 0.01 | 0.00 | 0.08 | 0.00 | 0.12 | -0.01 | 0.00 | 0.00 | 0.00 | -0.02 | 0.01 | -0.02 |
| Commodities not elsewhere specified | '99 | 0.04 | 0.16 | 1.20 | -6.73 | -0.01 | 1.33 | -0.24 | -0.51 | 0.38 | … | -0.85 | 0.00 | -0.26 | 0.65 | 0.01 | 0.27 | 0.83 | 0.00 | -0.83 | -1.54 | 1.15 |

| **Product label** | **Code** | **Kor** | **Mal** | **Mex** | **Neth** | **Nor** | **Paki** | **Phil** | **Pol** | **Port** | **Rom** | **Sing** | **Spa** | **Swe** | **Swi** | **Taiwan** | **Thai** | **Tun** | **Turk** | **UK** | **US** | **Viet** |
| --- | --- | --- | --- | --- | --- | --- | --- | --- | --- | --- | --- | --- | --- | --- | --- | --- | --- | --- | --- | --- | --- | --- |
| Dairy products | '04 | -0.06 | -0.21 | -0.23 | 0.64 | 0.03 | 0.00 | -0.61 | 0.47 | -0.12 | -0.02 | -0.09 | -0.07 | -0.06 | 0.05 | -0.10 | -0.17 | -0.06 | 0.04 | -0.11 | 0.01 | 0.11 |
| Products of animal origin | '05 | -0.02 | -0.01 | -0.03 | 0.01 | -0.01 | 0.06 | 0.00 | -0.01 | 0.00 | 0.00 | 0.00 | 0.01 | -0.03 | -0.02 | 0.01 | -0.01 | 0.07 | 0.03 | 0.00 | 0.02 | 0.06 |
| Lac; gums, resins | '13 | -0.01 | -0.01 | -0.01 | 0.00 | -0.02 | 0.11 | 0.04 | -0.03 | -0.01 | -0.01 | 0.00 | 0.04 | -0.01 | 0.01 | 0.00 | -0.01 | -0.01 | -0.02 | -0.01 | 0.00 | 0.01 |
| Vegetable plaiting materials | '14 | 0.00 | 0.00 | 0.00 | -0.01 | 0.00 | -0.04 | 0.00 | 0.00 | 0.00 | 0.00 | 0.00 | 0.00 | -0.01 | 0.00 | 0.00 | 0.00 | 0.00 | 0.03 | 0.00 | 0.00 | 0.05 |
| Animal or vegetable fats and oils | '15 | -0.09 | 1.62 | -0.11 | 0.09 | -0.17 | -2.83 | 0.60 | -0.14 | 0.04 | 0.00 | 0.01 | 0.41 | -0.02 | -0.06 | -0.05 | 0.05 | 0.26 | 0.02 | -0.07 | 0.05 | -0.07 |
| Preparations of meat, fish, molluscs | '16 | 0.03 | 0.03 | -0.02 | 0.06 | 0.08 | 0.06 | 0.12 | 0.19 | 0.06 | 0.01 | -0.06 | 0.16 | -0.14 | -0.10 | 0.02 | 2.07 | 0.00 | 0.03 | -0.23 | -0.04 | 0.29 |
| Sugars and sugar confectionery | '17 | -0.12 | -0.17 | 0.03 | 0.06 | -0.16 | 0.36 | -0.05 | 0.15 | -0.14 | -0.48 | -0.05 | 0.06 | -0.07 | -0.03 | -0.06 | 0.67 | -0.31 | 0.54 | -0.07 | -0.02 | 0.06 |
| Cocoa and cocoa preparations | '18 | -0.03 | 0.02 | -0.04 | 0.16 | -0.10 | -0.01 | -0.06 | 0.07 | -0.13 | -0.06 | 0.02 | -0.02 | -0.04 | 0.10 | -0.02 | -0.01 | -0.01 | 0.07 | -0.04 | -0.02 | -0.01 |
| Preparations of cereals, flour | '19 | 0.04 | -0.03 | -0.01 | 0.16 | -0.23 | -0.06 | -0.07 | 0.10 | -0.16 | -0.05 | -0.01 | 0.05 | -0.07 | 0.02 | -0.10 | 0.06 | 0.24 | 0.18 | 0.02 | 0.02 | 0.37 |
| Preparations of vegetables, fruit, nuts | '20 | -0.07 | -0.02 | 0.01 | 0.25 | -0.14 | 0.01 | 0.26 | 0.28 | 0.07 | -0.08 | -0.03 | 0.41 | -0.21 | -0.08 | -0.03 | 0.53 | 0.17 | 0.86 | -0.15 | 0.03 | 0.28 |
| Miscellaneous edible preparations | '21 | -0.09 | -0.02 | -0.06 | 0.22 | -0.17 | -0.01 | -0.19 | 0.00 | -0.11 | -0.24 | 0.01 | 0.02 | -0.14 | 0.09 | -0.09 | 0.18 | -0.08 | 0.05 | -0.01 | 0.13 | 0.12 |
| Beverages, spirits and vinegar | '22 | -0.07 | 0.03 | 0.44 | 0.26 | -0.29 | 0.09 | -0.05 | -0.03 | 0.65 | 0.04 | -0.06 | 0.41 | -0.11 | -0.49 | -0.11 | 0.03 | 0.15 | 0.04 | 0.10 | -0.26 | 0.02 |
| Residues and waste from food industries | '23 | -0.21 | -0.13 | -0.11 | 0.14 | -0.15 | -0.07 | -0.43 | -0.29 | -0.24 | -0.11 | 0.00 | -0.18 | -0.16 | -0.07 | -0.13 | -0.25 | -0.27 | -0.12 | -0.09 | 0.26 | -0.53 |
| Tobacco and manufactured tobacco substitutes | '24 | -0.06 | -0.01 | 0.01 | 0.56 | -0.09 | 0.04 | -0.18 | -0.01 | 0.03 | -0.43 | 0.01 | -0.29 | -0.07 | 0.09 | -0.27 | -0.03 | -0.08 | 0.39 | 0.13 | 0.23 | -0.16 |
| Salt; sulphur; earths and stone; lime and cement | '25 | -0.11 | -0.09 | 0.01 | -0.09 | -0.10 | 0.00 | -0.23 | -0.10 | -0.16 | 0.04 | -0.09 | 0.02 | -0.13 | -0.09 | -0.18 | 0.28 | -0.05 | 0.77 | 0.04 | 0.02 | -0.23 |
| Ores, slag and ash | '26 | -0.80 | -0.11 | -0.04 | -0.09 | -0.20 | -0.17 | -0.37 | -0.22 | 0.19 | -0.58 | 0.01 | -0.22 | 0.10 | 0.00 | -0.24 | -0.16 | 0.03 | 0.01 | -0.23 | 0.01 | 0.22 |
| Mineral fuels, mineral oils and products of their distillation | '27 | -9.81 | 2.18 | 2.47 | -1.78 | 29.94 | -11.1 | -4.86 | -2.26 | -4.10 | -3.36 | -2.49 | -4.23 | -2.90 | -2.19 | -4.94 | -4.66 | -0.22 | -9.23 | 1.93 | -4.53 | 8.58 |
| Inorganic chemicals | '28 | -0.36 | -0.24 | -0.12 | 0.01 | -0.52 | -0.35 | -0.29 | 0.05 | -0.07 | 0.36 | -0.13 | -0.12 | -0.26 | -0.02 | -0.29 | -0.32 | 1.26 | -0.07 | 0.14 | 0.13 | -0.44 |
| Organic chemicals | '29 | -0.25 | -0.36 | -0.77 | 0.74 | -0.22 | -3.49 | -0.71 | -0.25 | -0.23 | 0.07 | 1.03 | -0.34 | -0.78 | 1.62 | -1.26 | -0.90 | -0.51 | -1.68 | 0.42 | -0.07 | -0.61 |
| Pharmaceutical products | '30 | -0.19 | -0.21 | -0.10 | 0.04 | -0.90 | -0.44 | -0.41 | -1.63 | -0.81 | -1.20 | 0.01 | -0.40 | 1.64 | 2.98 | -0.37 | -0.24 | -0.90 | -1.09 | 1.06 | 0.17 | -1.01 |
| Fertilisers | '31 | -0.01 | -0.11 | -0.09 | 0.16 | -0.14 | -0.89 | -0.25 | 0.12 | -0.02 | 0.68 | 0.00 | -0.06 | -0.05 | -0.02 | -0.04 | -0.39 | 2.10 | -0.29 | -0.04 | 0.07 | -1.26 |
| Tanning or dyeing extracts; paints and varnishes | '32 | -0.16 | -0.11 | -0.11 | 0.19 | -0.36 | -0.58 | -0.22 | -0.47 | -0.34 | -0.42 | 0.12 | 0.17 | -0.07 | 0.56 | -0.07 | -0.51 | -0.20 | -0.43 | 0.19 | 0.17 | -0.49 |
| Essential oils; perfumery, cosmetics | '33 | -0.17 | -0.16 | -0.07 | -0.02 | -0.40 | -0.12 | -0.21 | -0.02 | -0.32 | -0.36 | -0.02 | 0.06 | -0.11 | 0.40 | -0.17 | 0.09 | 0.06 | -0.14 | 0.17 | 0.18 | -0.07 |
| Soap, washing preparations, waxes, candles | '34 | -0.06 | 0.02 | 0.04 | 0.11 | -0.17 | -0.25 | -0.09 | 0.01 | -0.12 | -0.17 | -0.02 | 0.23 | -0.09 | -0.03 | -0.10 | -0.13 | 0.00 | 0.21 | 0.04 | 0.10 | 0.03 |
| Albuminoidal substances | '35 | -0.04 | -0.03 | -0.10 | 0.09 | -0.05 | -0.02 | -0.10 | -0.10 | -0.07 | -0.14 | 0.00 | -0.07 | -0.01 | 0.04 | 0.03 | 0.11 | -0.08 | -0.09 | 0.02 | 0.07 | -0.12 |
| Explosives; pyrotechnic products; matches | '36 | 0.00 | 0.00 | -0.01 | 0.00 | -0.04 | 0.10 | 0.00 | 0.00 | -0.02 | -0.01 | -0.01 | 0.01 | 0.01 | 0.01 | 0.00 | 0.00 | 0.01 | 0.01 | 0.00 | 0.01 | -0.01 |
| Photographic or cinematographic goods | '37 | -0.19 | -0.04 | -0.07 | 0.07 | -0.11 | -0.12 | -0.10 | -0.11 | -0.10 | -0.07 | 0.01 | -0.05 | -0.11 | -0.04 | -0.21 | -0.09 | -0.08 | -0.13 | 0.09 | 0.08 | -0.06 |
| Miscellaneous chemical products | '38 | -0.51 | 0.13 | -0.40 | 0.23 | -0.20 | -0.99 | -0.38 | -0.60 | -0.15 | -0.51 | -0.04 | -0.15 | -0.11 | 0.29 | -0.75 | -0.53 | -0.37 | -0.47 | 0.29 | 0.54 | -0.93 |
| Plastics and articles thereof | '39 | 1.29 | -0.43 | -2.12 | 1.25 | -1.22 | -1.28 | -1.02 | -1.35 | -0.44 | -1.25 | 0.32 | 0.29 | -0.25 | -0.13 | 1.47 | 0.19 | -1.33 | -1.06 | -0.11 | 1.02 | -1.83 |
| Rubber and articles thereof | '40 | 0.38 | 0.53 | -0.54 | -0.09 | -0.38 | -0.66 | -0.13 | 0.26 | -0.11 | -0.22 | -0.04 | 0.30 | -0.29 | -0.22 | 0.17 | 1.72 | -0.13 | 0.33 | 0.01 | 0.06 | 0.49 |
| Raw hides and skins (other than furskins) and leather | '41 | -0.04 | -0.05 | -0.17 | 0.03 | -0.03 | 0.96 | -0.09 | -0.17 | -0.43 | -1.35 | 0.01 | -0.01 | 0.03 | 0.01 | 0.07 | -0.16 | -0.63 | -0.40 | 0.04 | 0.14 | -0.65 |
| Articles of leather; saddlery; travel goods, handbags | '42 | 0.04 | -0.01 | -0.05 | -0.06 | -0.16 | 2.10 | 0.52 | 0.04 | -0.14 | 0.30 | -0.08 | -0.06 | -0.14 | -0.17 | 0.00 | 0.47 | 0.30 | 0.48 | -0.15 | -0.27 | 0.84 |
| Furskins and artificial fur; manufactures thereof | '43 | -0.04 | 0.00 | 0.00 | 0.01 | 0.02 | 0.00 | 0.00 | 0.04 | 0.02 | 0.01 | 0.00 | 0.06 | 0.01 | -0.01 | 0.00 | 0.00 | 0.00 | 0.05 | 0.00 | 0.00 | 0.02 |
| Wood and articles of wood | '44 | -0.55 | 1.42 | -0.13 | -0.38 | -0.85 | -0.10 | -0.06 | 1.13 | 0.10 | 1.95 | -0.02 | -0.27 | 0.99 | -0.17 | -0.28 | 0.17 | -0.52 | -0.02 | -0.47 | -0.29 | 0.13 |
| Cork and articles of cork | '45 | 0.00 | 0.00 | 0.00 | 0.00 | 0.00 | 0.00 | 0.00 | 0.00 | 1.46 | -0.01 | 0.00 | 0.08 | 0.00 | -0.01 | 0.00 | 0.00 | 0.09 | 0.00 | 0.00 | -0.01 | 0.00 |
| Manufactures of straw, basketware | '46 | -0.01 | 0.00 | 0.00 | -0.01 | -0.01 | 0.00 | 0.14 | 0.03 | 0.00 | 0.02 | 0.00 | -0.01 | -0.01 | -0.01 | 0.00 | 0.00 | 0.01 | 0.00 | -0.01 | -0.01 | 0.34 |
| Pulp of wood or of other fibrous cellulosic material | '47 | -0.44 | -0.04 | -0.15 | -0.10 | 0.08 | -0.16 | -0.07 | -0.12 | 0.76 | 0.03 | 0.02 | 0.03 | 0.89 | -0.10 | -0.25 | -0.14 | -0.10 | -0.18 | -0.14 | 0.15 | -0.25 |
| Paper and paperboard; articles of paper pulp, of paper or of paperboard | '48 | 0.33 | -0.48 | -0.72 | -0.01 | -0.44 | -0.74 | -0.43 | 0.13 | 0.39 | -0.61 | -0.13 | -0.04 | 3.74 | -0.06 | -0.16 | 0.08 | -0.41 | -0.36 | -0.57 | 0.10 | -0.62 |
| Printed books, newspapers, pictures and products of the printing industry | '49 | -0.01 | -0.04 | -0.14 | 0.04 | -0.38 | -0.03 | -0.09 | -0.07 | -0.17 | -0.08 | 0.15 | 0.38 | -0.12 | -0.38 | -0.06 | -0.01 | -0.12 | -0.05 | 0.25 | 0.16 | -0.01 |
| Silk | '50 | -0.01 | -0.01 | 0.00 | 0.00 | 0.00 | -0.01 | 0.00 | -0.01 | -0.01 | -0.01 | -0.01 | -0.01 | 0.00 | 0.00 | 0.00 | 0.01 | -0.01 | -0.01 | 0.00 | -0.01 | 0.12 |
| Wool, fine or coarse animal hair; horsehair yarn and woven fabric | '51 | -0.09 | 0.00 | -0.03 | 0.00 | -0.02 | -0.03 | -0.06 | -0.11 | -0.03 | -0.70 | 0.00 | 0.02 | -0.01 | -0.02 | 0.00 | -0.07 | -0.27 | -0.06 | 0.02 | -0.01 | -0.01 |
| Cotton | '52 | -0.22 | -0.14 | -0.38 | 0.02 | -0.05 | 10.36 | -0.42 | -0.50 | -0.44 | -1.54 | -0.02 | 0.16 | -0.03 | 0.07 | 0.03 | -0.30 | -3.60 | 0.27 | -0.06 | 0.20 | -0.55 |
| Other vegetable textile fibres; paper yarn and woven fabrics of paper yarn | '53 | -0.02 | 0.00 | 0.00 | 0.00 | 0.00 | -0.11 | 0.00 | -0.01 | -0.04 | -0.07 | 0.00 | -0.01 | 0.00 | 0.00 | 0.00 | -0.02 | -0.05 | -0.06 | 0.00 | 0.00 | 0.02 |
| Man-made filaments; strip and the like of man-made textile materials | '54 | 1.06 | 0.15 | -0.21 | -0.04 | -0.04 | 2.31 | -0.25 | -0.33 | -0.30 | -1.13 | 0.05 | 0.02 | -0.06 | 0.06 | 1.21 | 0.00 | -0.69 | 0.10 | 0.00 | 0.06 | -0.41 |
| Man-made staple fibres | '55 | 0.13 | -0.02 | -0.12 | -0.02 | -0.05 | -0.09 | -0.29 | -0.33 | 0.00 | -1.29 | -0.02 | 0.08 | -0.01 | -0.01 | 0.47 | 0.33 | -1.44 | 0.41 | -0.01 | 0.06 | -1.68 |
| Wadding, felt and nonwovens; special yarns; twine, cordage, ropes | '56 | 0.10 | -0.01 | -0.08 | 0.02 | -0.06 | 0.20 | 0.02 | -0.10 | 0.15 | -0.26 | -0.02 | -0.03 | 0.00 | 0.00 | 0.09 | 0.04 | -0.12 | -0.01 | -0.01 | 0.03 | -0.14 |
| Carpets and other textile floor coverings | '57 | 0.00 | -0.01 | -0.03 | 0.10 | -0.08 | 0.99 | 0.00 | -0.03 | 0.04 | 0.00 | -0.02 | -0.02 | -0.06 | -0.04 | -0.01 | 0.04 | 0.01 | 0.38 | -0.08 | -0.01 | 0.03 |
| Special woven fabrics; tufted textile fabrics; lace; tapestries | '58 | 0.23 | -0.02 | -0.14 | 0.01 | -0.02 | 0.03 | -0.06 | -0.13 | -0.03 | -0.37 | -0.01 | 0.00 | -0.01 | 0.06 | 0.20 | 0.02 | -0.43 | 0.34 | -0.01 | 0.03 | -0.27 |
| Impregnated, coated, covered or laminated textile fabrics | '59 | 0.41 | -0.03 | -0.16 | 0.02 | -0.05 | -0.10 | -0.13 | -0.18 | 0.30 | -0.37 | -0.02 | 0.02 | 0.01 | 0.08 | 0.62 | -0.03 | -0.21 | 0.15 | 0.05 | 0.06 | -0.61 |
| Knitted or crocheted fabrics | '60 | 0.80 | -0.06 | -0.14 | 0.01 | -0.01 | 0.20 | -0.36 | -0.19 | -0.11 | -0.40 | -0.04 | 0.06 | 0.01 | 0.01 | 0.89 | -0.12 | -0.39 | 0.31 | 0.00 | 0.02 | -0.16 |
| Articles of apparel and clothing accessories, knitted or crocheted | '61 | 0.51 | 0.37 | 0.49 | -0.30 | -0.73 | 5.95 | 1.24 | 0.36 | 2.88 | 2.42 | 0.05 | -0.19 | -0.53 | -0.63 | 0.37 | 1.40 | 3.40 | 5.95 | -0.73 | -0.92 | 1.24 |
| Articles of apparel and clothing accessories, not knitted or crocheted | '62 | 0.23 | 0.23 | 1.03 | -0.40 | -0.92 | 4.81 | 2.40 | 1.83 | 1.42 | 8.38 | -0.08 | -0.13 | -0.66 | -0.82 | 0.09 | 1.25 | 13.57 | 4.25 | -0.78 | -1.23 | 5.12 |
| Other made-up textile articles; sets; worn clothing | '63 | 0.13 | -0.04 | 0.17 | -0.04 | -0.21 | 10.66 | 0.08 | 0.33 | 1.50 | 0.19 | -0.03 | 0.01 | -0.14 | -0.13 | 0.09 | 0.15 | 0.55 | 1.72 | -0.10 | -0.15 | 0.61 |
| Footwear, gaiters and the like | '64 | 0.12 | 0.00 | 0.03 | -0.15 | -0.34 | 0.36 | 0.03 | 0.14 | 2.61 | 3.46 | -0.05 | 0.69 | -0.26 | -0.34 | 0.09 | 0.69 | 1.86 | 0.11 | -0.38 | -0.62 | 5.96 |
| Headgear and parts thereof | '65 | 0.07 | 0.01 | 0.00 | -0.01 | -0.03 | 0.00 | 0.03 | 0.01 | 0.00 | 0.02 | 0.00 | -0.01 | -0.01 | -0.03 | 0.08 | 0.03 | 0.03 | 0.00 | -0.02 | -0.05 | 0.10 |
| Umbrellas, sun umbrellas, walking sticks | '66 | -0.01 | 0.00 | 0.00 | 0.00 | -0.01 | 0.00 | -0.01 | 0.01 | -0.01 | 0.00 | 0.00 | -0.01 | -0.01 | -0.01 | 0.02 | 0.00 | 0.01 | 0.00 | -0.01 | -0.01 | 0.00 |
| Prepared feathers and down; artificial flowers; articles of human hair | '67 | 0.01 | 0.00 | -0.01 | -0.01 | -0.01 | 0.00 | 0.02 | -0.01 | -0.01 | 0.00 | 0.00 | -0.01 | -0.01 | -0.01 | 0.00 | 0.06 | 0.01 | -0.01 | -0.01 | -0.05 | 0.05 |
| Articles of stone, plaster, cement, mica or similar materials | '68 | -0.07 | -0.01 | 0.04 | -0.03 | -0.14 | 0.05 | -0.04 | -0.01 | 0.21 | -0.19 | -0.07 | 0.28 | -0.03 | -0.08 | -0.04 | -0.02 | -0.02 | 0.23 | 0.03 | -0.07 | -0.02 |
| Ceramic products | '69 | -0.10 | 0.01 | 0.09 | -0.05 | -0.22 | -0.12 | 0.01 | -0.04 | 0.61 | 0.03 | -0.06 | 0.87 | -0.07 | -0.15 | -0.06 | 0.30 | 0.22 | 0.46 | 0.00 | -0.09 | 0.53 |
| Glass and glassware | '70 | -0.10 | -0.03 | 0.03 | -0.01 | -0.18 | -0.13 | 0.00 | 0.20 | 0.25 | 0.16 | -0.07 | 0.08 | -0.09 | -0.14 | -0.01 | 0.01 | -0.14 | 0.53 | -0.02 | 0.08 | -0.12 |
| Natural or cultured pearls, precious stones, imitation jewellery, coin | '71 | -0.22 | -0.45 | 0.01 | -0.05 | -0.03 | -0.97 | 0.13 | 0.22 | -0.26 | 0.50 | -0.16 | -0.14 | 0.08 | -1.44 | -0.42 | -0.01 | -0.09 | -0.50 | -0.07 | -0.13 | 0.18 |
| Iron and steel | '72 | -0.10 | -0.97 | -0.62 | 0.10 | -0.18 | -1.72 | -1.24 | -0.04 | -0.84 | 1.81 | -0.29 | -0.27 | 0.43 | -0.46 | -0.10 | -1.67 | -1.01 | 1.29 | 0.13 | -0.11 | -3.06 |
| Articles of iron or steel | '73 | 0.33 | -0.54 | -0.61 | -0.14 | -1.20 | -0.40 | -0.31 | 1.04 | 0.03 | 0.54 | -0.32 | 0.38 | -0.06 | 0.14 | 1.09 | -0.43 | -0.34 | 0.61 | 0.02 | -0.03 | -0.52 |
| Copper and articles thereof | '74 | -0.21 | -0.28 | -0.21 | -0.08 | -0.06 | -0.16 | 0.25 | 0.82 | -0.22 | -0.04 | -0.07 | 0.00 | 0.06 | -0.06 | -0.23 | -0.36 | -0.26 | -0.05 | -0.03 | -0.06 | -0.28 |
| Nickel and articles thereof | '75 | -0.15 | -0.01 | -0.03 | 0.07 | -0.59 | -0.05 | -0.01 | -0.02 | -0.01 | -0.04 | -0.03 | -0.07 | -0.17 | -0.02 | -0.15 | -0.01 | 0.00 | -0.02 | -0.01 | 0.00 | 0.01 |
| Aluminium and articles thereof | '76 | -0.46 | -0.27 | -0.42 | 0.04 | 1.11 | -0.41 | -0.21 | -0.06 | -0.15 | 0.85 | -0.26 | 0.06 | -0.03 | 0.03 | -0.26 | -0.46 | -0.15 | 0.03 | -0.04 | -0.03 | -0.42 |
| Lead and articles thereof | '78 | -0.02 | -0.02 | -0.01 | -0.01 | -0.01 | -0.03 | -0.01 | 0.01 | -0.02 | 0.03 | 0.00 | -0.02 | 0.02 | 0.00 | -0.03 | -0.03 | -0.02 | -0.03 | -0.01 | 0.00 | -0.04 |
| Zinc and articles thereof | '79 | 0.04 | -0.06 | 0.04 | 0.02 | 0.10 | -0.07 | -0.06 | 0.11 | -0.04 | 0.14 | -0.06 | 0.06 | -0.03 | -0.02 | -0.11 | 0.00 | -0.03 | -0.08 | 0.00 | -0.04 | -0.11 |
| Tin and articles thereof | '80 | -0.02 | 0.06 | -0.01 | -0.01 | -0.01 | -0.01 | 0.00 | -0.01 | 0.00 | -0.01 | 0.05 | -0.01 | 0.00 | 0.00 | -0.02 | 0.07 | 0.00 | -0.01 | 0.00 | -0.01 | 0.04 |
| Other base metals; cermets; articles thereof | '81 | -0.08 | -0.01 | -0.09 | 0.00 | -0.01 | -0.01 | 0.11 | -0.01 | -0.04 | -0.02 | 0.00 | -0.01 | -0.06 | -0.01 | -0.06 | 0.00 | 0.00 | -0.01 | -0.02 | 0.03 | 0.00 |
| Tools, implements, cutlery, spoons and forks, of base metal | '82 | 0.08 | -0.12 | 0.00 | -0.07 | -0.21 | 0.07 | -0.03 | -0.06 | -0.02 | -0.11 | -0.04 | -0.01 | 0.12 | 0.26 | 0.45 | -0.28 | -0.15 | -0.09 | 0.06 | 0.01 | -0.01 |
| Miscellaneous articles of base metal | '83 | 0.04 | -0.02 | -0.02 | -0.04 | -0.13 | -0.05 | -0.03 | -0.16 | 0.05 | -0.24 | -0.02 | 0.07 | -0.08 | -0.05 | 0.31 | -0.02 | -0.14 | 0.00 | -0.05 | 0.01 | -0.09 |
| Machinery, mechanical appliances, nuclear reactors, boilers | '84 | 1.89 | 2.99 | -0.77 | 0.77 | -5.30 | -6.29 | 4.34 | -2.66 | -2.80 | -3.15 | 3.48 | -1.78 | -0.30 | 2.55 | 4.93 | 1.36 | -5.78 | -4.63 | 1.55 | 3.05 | -4.15 |
| Electrical machinery and equipment; sound recorders and reproducers, tv | '85 | 2.07 | -2.45 | 0.69 | -1.70 | -3.50 | -2.54 | 4.51 | -0.22 | 0.96 | -1.06 | 2.09 | -0.81 | 0.54 | -0.06 | -0.43 | -0.64 | 1.21 | -0.59 | 1.11 | 1.78 | -1.41 |
| Railway or tramway locomotives; railway or tramway track | '86 | -0.03 | -0.06 | 0.09 | 0.00 | -0.10 | -0.33 | -0.03 | 0.23 | -0.04 | 0.22 | -0.05 | 0.10 | -0.02 | 0.03 | -0.04 | -0.02 | -0.05 | -0.09 | -0.03 | 0.04 | -0.02 |
| Vehicles other than railway or tramway rolling stock | '87 | 4.49 | -0.95 | 3.78 | -1.17 | -3.89 | -2.28 | -0.46 | 0.57 | 0.14 | -1.33 | -0.66 | 4.22 | 1.14 | -3.72 | 0.62 | 0.72 | -2.43 | 1.70 | -2.57 | -2.91 | -3.03 |
| Aircraft, spacecraft, and parts thereof | '88 | -0.11 | -0.29 | 0.06 | -0.11 | -1.52 | -0.55 | 0.04 | 0.03 | -0.15 | 0.15 | -1.32 | 0.02 | -0.26 | -0.30 | -0.85 | -1.10 | -0.62 | 0.57 | -0.04 | 2.21 | -0.07 |
| Ships, boats and floating structures | '89 | 3.11 | -0.05 | -0.02 | 0.18 | -0.63 | -0.07 | 0.06 | 1.96 | 0.07 | 0.96 | 0.19 | 0.09 | 0.16 | -0.03 | 0.05 | -0.06 | -0.05 | -0.29 | 0.07 | 0.08 | -0.25 |
| Optical, photographic, cinematographic, medical or surgical instruments | '90 | -1.44 | -0.46 | 0.20 | 0.48 | -0.81 | -0.04 | 0.18 | -0.73 | -0.63 | -1.08 | -0.10 | -0.62 | -0.18 | 1.42 | -1.64 | 0.04 | -0.61 | -1.02 | 0.31 | 1.57 | -0.54 |
| Clocks and watches and parts thereof | '91 | 0.00 | -0.09 | -0.02 | -0.04 | -0.06 | -0.03 | 0.13 | -0.03 | -0.11 | -0.01 | -0.07 | -0.10 | -0.05 | 3.26 | -0.05 | 0.06 | 0.01 | -0.05 | -0.08 | -0.10 | -0.01 |
| Musical instruments; parts and accessories of such articles | '92 | 0.05 | 0.05 | 0.01 | -0.01 | -0.03 | 0.01 | -0.01 | -0.01 | -0.01 | 0.03 | -0.01 | 0.00 | -0.01 | -0.02 | 0.07 | 0.00 | -0.01 | 0.00 | -0.02 | -0.03 | -0.01 |
| Arms and ammunition; parts and accessories thereof | '93 | -0.02 | 0.00 | 0.00 | 0.00 | -0.03 | 0.06 | 0.01 | -0.01 | 0.03 | 0.11 | 0.00 | 0.01 | 0.00 | 0.01 | 0.00 | 0.00 | -0.01 | -0.14 | 0.03 | 0.12 | -0.01 |
| Furniture; bedding, mattresses, stuffed furnishings; lamps | '94 | -0.04 | 0.74 | 0.88 | -0.36 | -1.04 | 0.09 | 0.43 | 3.22 | 0.28 | 1.91 | -0.16 | 0.49 | 0.07 | -0.81 | 0.52 | 0.74 | 0.04 | 0.20 | -0.34 | -0.60 | 1.15 |
| Toys, games and sports requisites | '95 | -0.01 | 0.03 | 0.04 | 0.05 | -0.35 | 1.17 | 0.12 | -0.06 | -0.22 | 0.19 | -0.08 | -0.06 | -0.21 | -0.15 | 0.59 | 0.36 | 0.03 | -0.06 | -0.32 | -0.63 | 0.21 |
| Miscellaneous manufactured articles | '96 | 0.13 | -0.03 | -0.01 | -0.02 | -0.11 | -0.15 | -0.04 | -0.11 | -0.07 | -0.32 | -0.02 | -0.01 | -0.05 | 0.01 | 0.19 | 0.02 | -0.15 | -0.08 | -0.04 | -0.05 | -0.05 |
| Works of art, collectors' pieces and antiques | '97 | 0.00 | 0.00 | 0.00 | -0.01 | 0.01 | 0.00 | 0.00 | 0.00 | 0.01 | 0.01 | 0.00 | -0.07 | 0.00 | -0.18 | -0.01 | 0.00 | 0.00 | -0.01 | 0.11 | 0.05 | 0.00 |
| Commodities not elsewhere specified | '99 | 0.00 | -0.48 | -0.22 | 5.07 | 1.98 | -0.06 | 0.01 | -0.07 | 0.13 | 0.11 | 1.49 | 0.53 | 0.52 |  | -0.60 | 1.55 | -0.23 | -0.16 | -0.59 | -0.51 | -0.07 |

**Table S 6. Lafay Index. 2019**

| **Product label** | **Code** | **Bang** | **Belg** | **Brazil** | **Bulg** | **Camb** | **Chile** | **Chin** | **Col** | **Czech** | **Eth** | **Fin** | **Fran** | **Germ** | **Gre** | **Hon** | **Hung** | **India** | **Indo** | **Irel** | **Italy** | **Jap** |
| --- | --- | --- | --- | --- | --- | --- | --- | --- | --- | --- | --- | --- | --- | --- | --- | --- | --- | --- | --- | --- | --- | --- |
| Dairy products | '04 | -0.36 | -0.01 | -0.08 | -0.03 | -0.08 | -0.11 | -0.15 | -0.15 | 0.02 | -0.01 | 0.04 | 0.30 | -0.02 | 0.30 | 4.91 | 0.00 | 0.07 | -0.23 | 0.52 | -0.09 | -0.14 |
| Products of animal origin | '05 | -0.04 | 0.00 | 0.11 | -0.01 | 0.00 | -0.02 | 0.03 | 0.04 | -0.01 | 0.01 | -0.02 | -0.01 | -0.01 | -0.01 | 2.52 | 0.01 | 0.01 | -0.05 | 0.00 | -0.01 | -0.05 |
| Lac; gums, resins | '13 | -0.02 | -0.01 | 0.01 | -0.04 | -0.01 | 0.08 | 0.02 | -0.02 | 0.00 | 0.10 | -0.01 | 0.03 | -0.01 | 0.01 | 0.85 | -0.01 | 0.12 | 0.02 | -0.03 | 0.00 | -0.02 |
| Vegetable plaiting materials | '14 | -0.01 | 0.00 | 0.00 | 0.00 | 0.00 | 0.03 | 0.00 | 0.00 | 0.00 | 0.00 | 0.00 | 0.00 | 0.00 | -0.01 | 0.83 | 0.01 | 0.00 | 0.06 | 0.00 | 0.00 | -0.01 |
| Animal or vegetable fats and oils | '15 | -1.12 | -0.04 | 0.04 | 0.41 | 0.06 | -0.26 | -0.23 | 0.23 | 0.00 | -0.06 | -0.24 | -0.07 | -0.07 | 0.43 | 0.71 | 0.15 | -0.81 | 5.39 | -0.18 | -0.21 | -0.09 |
| Preparations of meat, fish, molluscs | '16 | 0.01 | 0.00 | 0.29 | 0.06 | -0.04 | 0.19 | 0.18 | -0.23 | -0.02 | -0.01 | -0.18 | -0.08 | -0.02 | -0.05 | 0.44 | 0.00 | 0.07 | 0.36 | -0.02 | -0.06 | -0.45 |
| Sugars and sugar confectionery | '17 | -0.62 | 0.04 | 1.59 | -0.03 | 0.11 | -0.19 | 0.00 | 0.52 | 0.02 | 0.11 | -0.07 | 0.10 | 0.01 | -0.09 | 0.47 | 0.01 | 0.27 | -0.43 | -0.14 | -0.08 | -0.04 |
| Cocoa and cocoa preparations | '18 | -0.01 | 0.12 | 0.00 | 0.01 | -0.01 | -0.10 | -0.01 | 0.09 | -0.06 | -0.01 | -0.09 | -0.09 | 0.00 | -0.10 | 0.45 | -0.06 | 0.00 | 0.13 | -0.12 | 0.07 | -0.07 |
| Preparations of cereals, flour | '19 | -0.04 | 0.23 | 0.00 | 0.25 | -0.37 | -0.05 | -0.14 | -0.01 | -0.03 | 0.06 | -0.27 | 0.14 | 0.05 | 0.12 | 0.37 | -0.10 | 0.08 | 0.18 | 0.19 | 0.39 | -0.04 |
| Preparations of vegetables, fruit, nuts | '20 | 0.01 | 0.28 | 0.50 | 0.04 | 0.04 | 0.36 | 0.12 | -0.01 | -0.07 | 0.15 | -0.23 | -0.16 | -0.12 | 1.40 | 0.38 | 0.11 | 0.09 | 0.01 | -0.23 | 0.23 | -0.25 |
| Miscellaneous edible preparations | '21 | -0.09 | 0.07 | 0.23 | -0.12 | -0.25 | -0.10 | -0.01 | 0.05 | -0.04 | -0.08 | -0.22 | 0.08 | 0.06 | 0.14 | 0.33 | 0.05 | 0.11 | 0.15 | -0.18 | 0.14 | -0.01 |
| Beverages, spirits and vinegar | '22 | 0.02 | 0.11 | -0.03 | -0.12 | -0.79 | 1.43 | -0.10 | -0.42 | -0.05 | -0.01 | -0.31 | 1.43 | -0.13 | 0.04 | 0.25 | 0.17 | -0.04 | 0.01 | 0.01 | 0.81 | -0.20 |
| Residues and waste from food industries | '23 | -0.46 | 0.04 | 1.79 | 0.31 | -0.48 | -0.38 | -0.04 | -0.82 | 0.00 | 0.01 | -0.20 | 0.04 | -0.02 | -0.32 | 0.33 | 0.20 | 0.16 | -0.57 | -0.37 | -0.14 | -0.19 |
| Tobacco and manufactured tobacco substitutes | '24 | 0.11 | -0.01 | 0.62 | -0.08 | -0.51 | 0.05 | -0.02 | -0.03 | 0.04 | -0.02 | -0.08 | -0.11 | 0.01 | 0.47 | 0.30 | -0.03 | 0.15 | 0.16 | -0.04 | -0.09 | -0.40 |
| Salt; sulphur; earths and stone; lime and cement | '25 | -1.01 | -0.02 | -0.03 | 0.01 | -0.38 | -0.07 | -0.14 | -0.15 | -0.02 | 0.01 | -0.16 | -0.01 | -0.01 | 0.78 | 0.30 | -0.11 | 0.04 | -0.16 | 0.02 | -0.05 | -0.07 |
| Ores, slag and ash | '26 | -0.05 | -0.19 | 7.18 | -0.63 | -0.03 | 18.26 | -4.08 | 0.05 | -0.18 | 0.20 | -1.01 | -0.16 | -0.38 | 0.13 | 0.31 | -0.09 | 0.20 | 0.78 | -0.07 | -0.14 | -1.65 |
| Mineral fuels, mineral oils and products of their distillation | '27 | -5.79 | -1.94 | 1.97 | -2.03 | -5.73 | -8.59 | -7.70 | 26.48 | -1.85 | -0.31 | -3.52 | -3.93 | -3.43 | 2.42 | -0.21 | -2.77 | -8.32 | 3.29 | -2.72 | -5.02 | -10.6 |
| Inorganic chemicals | '28 | -0.35 | 0.04 | 0.55 | 0.33 | -0.09 | 1.14 | 0.08 | -0.24 | -0.16 | -0.03 | 0.03 | 0.00 | 0.03 | 0.11 | 0.27 | 0.02 | -0.40 | -0.25 | 0.09 | -0.08 | -0.06 |
| Organic chemicals | '29 | -0.80 | -0.10 | -2.59 | -0.25 | -0.07 | -0.43 | -0.31 | -1.93 | -0.07 | -0.09 | -0.10 | -0.21 | -0.83 | -1.70 | 0.27 | 0.01 | 0.83 | -0.96 | 7.31 | -1.04 | 0.15 |
| Pharmaceutical products | '30 | -0.14 | 0.58 | -1.78 | -0.39 | -0.53 | -1.18 | -0.66 | -2.15 | -0.78 | -0.75 | -1.08 | 1.28 | 0.66 | 0.39 | 0.18 | 0.45 | 2.33 | -0.11 | 11.39 | 0.23 | -1.56 |
| Fertilisers | '31 | -0.83 | 0.11 | -2.63 | -0.01 | -0.52 | 0.03 | 0.06 | -0.56 | -0.07 | -0.12 | 0.18 | -0.13 | -0.01 | 0.02 | 0.26 | -0.12 | -0.71 | -0.29 | -0.22 | -0.05 | -0.05 |
| Tanning or dyeing extracts; paints and varnishes | '32 | -0.73 | 0.12 | -0.25 | -0.25 | -0.19 | -0.23 | 0.03 | -0.28 | -0.15 | -0.09 | -0.05 | -0.01 | 0.17 | 0.06 | 0.23 | -0.17 | 0.34 | -0.30 | -0.05 | 0.02 | 0.24 |
| Essential oils; perfumery, cosmetics | '33 | -0.18 | -0.03 | 0.03 | 0.09 | -0.23 | -0.55 | -0.29 | 0.08 | -0.13 | -0.09 | -0.20 | 1.36 | 0.06 | -0.15 | 0.02 | -0.05 | 0.24 | -0.16 | 1.89 | 0.22 | 0.16 |
| Soap, washing preparations, waxes, candles | '34 | -0.20 | 0.17 | -0.07 | -0.09 | -0.17 | -0.26 | -0.03 | 0.01 | -0.01 | -0.07 | -0.15 | 0.10 | 0.09 | -0.05 | 0.23 | -0.02 | 0.01 | 0.13 | -0.18 | 0.08 | 0.14 |
| Albuminoidal substances | '35 | -0.11 | 0.04 | 0.02 | 0.07 | -0.10 | -0.10 | -0.03 | -0.08 | -0.07 | -0.04 | 0.08 | 0.10 | 0.03 | -0.07 | 0.21 | -0.05 | -0.02 | -0.14 | 0.10 | 0.01 | -0.02 |
| Explosives; pyrotechnic products; matches | '36 | 0.00 | 0.00 | 0.01 | 0.01 | 0.00 | 0.00 | 0.02 | -0.04 | 0.06 | 0.00 | -0.01 | 0.01 | 0.00 | 0.00 | 0.22 | -0.02 | 0.02 | -0.03 | 0.00 | -0.01 | -0.01 |
| Photographic or cinematographic goods | '37 | -0.04 | 0.06 | -0.02 | -0.01 | 0.00 | -0.02 | -0.05 | -0.03 | -0.01 | -0.01 | -0.01 | -0.01 | 0.00 | -0.03 | 0.17 | -0.01 | -0.02 | -0.02 | -0.12 | -0.02 | 0.35 |
| Miscellaneous chemical products | '38 | -0.63 | 0.26 | -1.28 | -0.17 | -0.43 | -0.62 | -0.10 | -0.32 | -0.28 | -0.26 | 0.06 | 0.48 | 0.23 | -0.09 | 0.13 | -0.20 | 0.23 | 0.47 | 0.69 | -0.13 | 0.46 |
| Plastics and articles thereof | '39 | -2.15 | 1.21 | -1.24 | -0.62 | -1.02 | -1.47 | -0.10 | -0.30 | -0.95 | -0.46 | -0.10 | -0.02 | 0.30 | 0.07 | -0.13 | -0.46 | -0.31 | -1.96 | -1.02 | -0.20 | 0.70 |
| Rubber and articles thereof | '40 | -0.31 | 0.01 | -0.38 | -0.01 | 0.30 | -0.57 | 0.06 | -0.81 | 0.23 | -0.22 | -0.19 | 0.02 | -0.07 | -0.29 | 0.15 | 0.25 | 0.18 | 1.23 | -0.19 | -0.06 | 0.44 |
| Raw hides and skins (other than furskins) and leather | '41 | 0.00 | 0.01 | 0.33 | -0.08 | -0.70 | 0.02 | -0.08 | 0.06 | -0.03 | 1.29 | 0.01 | 0.00 | 0.00 | 0.03 | 0.13 | -0.09 | 0.03 | -0.13 | 0.02 | 0.10 | 0.00 |
| Articles of leather; saddlery; travel goods, handbags | '42 | 0.36 | 0.00 | -0.12 | 0.02 | 3.68 | 0.13 | 0.50 | -0.09 | -0.04 | 0.26 | -0.11 | 0.46 | -0.11 | -0.14 | -0.02 | 0.06 | 0.35 | 0.07 | -0.08 | 0.74 | -0.47 |
| Furskins and artificial fur; manufactures thereof | '43 | 0.00 | 0.00 | 0.01 | 0.02 | 0.34 | 0.00 | 0.08 | 0.01 | 0.00 | 0.00 | 0.13 | 0.00 | 0.00 | 0.17 | 0.14 | 0.00 | 0.00 | 0.00 | 0.00 | 0.02 | -0.01 |
| Wood and articles of wood | '44 | -0.04 | -0.02 | 0.83 | 0.20 | 0.01 | 1.95 | -0.28 | -0.16 | 0.32 | -0.05 | 1.38 | -0.11 | -0.04 | -0.21 | 0.14 | -0.03 | -0.14 | 1.02 | -0.15 | -0.28 | -0.78 |
| Cork and articles of cork | '45 | 0.00 | 0.00 | 0.00 | 0.00 | 0.00 | -0.02 | 0.00 | 0.00 | 0.00 | 0.00 | 0.00 | -0.02 | 0.00 | -0.01 | 0.15 | 0.00 | 0.00 | 0.00 | 0.00 | -0.02 | 0.00 |
| Manufactures of straw, basketware | '46 | 0.03 | 0.00 | 0.00 | 0.00 | 0.00 | 0.00 | 0.03 | 0.00 | 0.00 | 0.00 | 0.00 | -0.01 | 0.00 | -0.01 | 0.15 | 0.00 | 0.01 | 0.03 | 0.00 | 0.00 | -0.02 |
| Pulp of wood or of other fibrous cellulosic material | '47 | -0.24 | -0.01 | 2.18 | 0.12 | 0.00 | 2.50 | -0.48 | -0.17 | 0.04 | -0.01 | 1.70 | -0.04 | -0.13 | -0.02 | 0.14 | -0.05 | -0.25 | 0.38 | -0.02 | -0.24 | -0.05 |
| Paper and paperboard; articles of paper pulp, of paper or of paperboard | '48 | -0.57 | -0.06 | 0.35 | -0.26 | -1.10 | -0.24 | 0.31 | -0.24 | -0.18 | -0.18 | 5.37 | -0.11 | 0.13 | -0.53 | 0.07 | -0.20 | 0.04 | 0.86 | -0.47 | 0.05 | -0.05 |
| Printed books, newspapers, pictures and products of the printing industry | '49 | -0.07 | 0.00 | -0.03 | 0.04 | -0.08 | -0.07 | 0.02 | -0.02 | 0.05 | -0.07 | -0.06 | -0.01 | 0.03 | 0.02 | 0.08 | 0.02 | 0.02 | -0.04 | -0.10 | 0.05 | -0.05 |
| Silk | '50 | -0.01 | 0.00 | 0.01 | -0.02 | -0.01 | 0.00 | 0.02 | 0.00 | 0.00 | -0.10 | 0.00 | 0.00 | 0.00 | 0.00 | 0.12 | 0.00 | -0.01 | 0.00 | 0.00 | -0.01 | -0.01 |
| Wool, fine or coarse animal hair; horsehair yarn and woven fabric | '51 | -0.07 | 0.00 | 0.01 | -0.09 | -0.09 | 0.03 | -0.03 | -0.01 | 0.01 | -0.01 | -0.01 | -0.01 | -0.01 | 0.00 | 0.07 | 0.00 | 0.00 | -0.04 | -0.01 | 0.04 | -0.02 |
| Cotton | '52 | -5.96 | 0.00 | 0.80 | -0.17 | -1.55 | -0.03 | 0.05 | -0.38 | -0.01 | 0.12 | 0.00 | -0.01 | -0.01 | 0.80 | 0.04 | -0.02 | 0.80 | -0.37 | 0.00 | 0.01 | -0.01 |
| Other vegetable textile fibres; paper yarn and woven fabrics of paper yarn | '53 | 0.52 | 0.03 | 0.01 | -0.01 | 0.00 | 0.00 | 0.00 | 0.00 | 0.00 | 0.00 | 0.00 | 0.06 | 0.00 | 0.00 | 0.08 | 0.00 | 0.03 | -0.02 | 0.00 | 0.00 | 0.00 |
| Man-made filaments; strip and the like of man-made textile materials | '54 | -1.39 | 0.00 | -0.37 | -0.11 | -0.30 | -0.06 | 0.38 | -0.19 | -0.02 | -0.14 | -0.04 | -0.02 | -0.02 | 0.04 | 0.05 | -0.03 | 0.27 | -0.26 | -0.01 | 0.02 | 0.09 |
| Man-made staple fibres | '55 | -1.64 | 0.04 | -0.24 | -0.08 | -2.42 | -0.06 | 0.19 | -0.25 | -0.03 | -0.06 | -0.04 | 0.00 | -0.03 | -0.03 | 0.06 | -0.04 | 0.18 | 0.33 | 0.03 | -0.02 | 0.05 |
| Wadding, felt and nonwovens; special yarns; twine, cordage, ropes | '56 | -0.12 | 0.00 | -0.04 | 0.02 | -0.19 | -0.12 | 0.09 | -0.08 | -0.02 | -0.02 | 0.01 | 0.01 | 0.03 | 0.12 | 0.07 | 0.03 | 0.03 | -0.12 | -0.04 | 0.08 | -0.01 |
| Carpets and other textile floor coverings | '57 | 0.02 | 0.16 | -0.02 | -0.01 | -0.01 | -0.04 | 0.06 | -0.02 | -0.03 | -0.02 | -0.06 | -0.02 | -0.03 | -0.02 | 0.06 | -0.02 | 0.26 | -0.01 | -0.03 | 0.00 | -0.04 |
| Special woven fabrics; tufted textile fabrics; lace; tapestries | '58 | -0.45 | 0.01 | -0.02 | -0.05 | -0.61 | -0.01 | 0.10 | -0.01 | -0.02 | -0.01 | 0.00 | 0.02 | 0.01 | 0.00 | 0.04 | -0.03 | 0.04 | -0.10 | -0.01 | 0.02 | 0.00 |
| Impregnated, coated, covered or laminated textile fabrics | '59 | -0.34 | 0.04 | -0.06 | -0.08 | -0.39 | -0.08 | 0.12 | -0.01 | -0.01 | -0.03 | -0.01 | 0.00 | 0.04 | -0.03 | 0.04 | -0.05 | -0.04 | -0.18 | -0.02 | 0.05 | 0.03 |
| Knitted or crocheted fabrics | '60 | -1.28 | 0.00 | -0.09 | -0.27 | -6.41 | -0.02 | 0.34 | -0.07 | -0.01 | -0.05 | 0.00 | 0.01 | 0.01 | 0.04 | 0.00 | -0.03 | 0.01 | -0.46 | -0.01 | 0.03 | 0.04 |
| Articles of apparel and clothing accessories, knitted or crocheted | '61 | 22.43 | 0.01 | -0.22 | 0.71 | 20.33 | -0.95 | 1.35 | -0.08 | -0.19 | 3.03 | -0.46 | -0.46 | -0.44 | -0.17 | -0.14 | -0.22 | 1.20 | 1.04 | -0.52 | 0.06 | -1.00 |
| Articles of apparel and clothing accessories, not knitted or crocheted | '62 | 21.01 | -0.11 | -0.22 | 0.96 | 7.55 | -0.93 | 1.24 | 0.04 | -0.12 | 2.34 | -0.44 | -0.37 | -0.38 | -0.40 | -0.14 | -0.15 | 1.26 | 1.25 | -0.46 | 0.40 | -1.05 |
| Other made-up textile articles; sets; worn clothing | '63 | 1.05 | -0.01 | -0.06 | 0.01 | 0.16 | -0.28 | 0.55 | -0.01 | 0.01 | -0.01 | -0.12 | -0.13 | -0.09 | -0.07 | 0.04 | -0.03 | 0.76 | 0.01 | -0.11 | -0.07 | -0.27 |
| Footwear, gaiters and the like | '64 | 1.04 | 0.17 | 0.21 | 0.02 | 3.87 | -0.73 | 0.82 | -0.37 | -0.15 | 0.63 | -0.18 | -0.27 | -0.25 | -0.24 | -0.08 | -0.08 | 0.37 | 1.10 | -0.24 | 0.41 | -0.40 |
| Headgear and parts thereof | '65 | 0.34 | 0.00 | -0.02 | 0.00 | 0.07 | -0.04 | 0.09 | -0.04 | -0.01 | 0.02 | -0.04 | -0.01 | -0.02 | -0.02 | 0.04 | -0.01 | 0.01 | 0.01 | -0.01 | 0.01 | -0.03 |
| Umbrellas, sun umbrellas, walking sticks | '66 | -0.02 | 0.00 | -0.01 | -0.01 | 0.07 | -0.01 | 0.06 | -0.01 | 0.00 | -0.01 | 0.00 | -0.01 | -0.01 | -0.01 | 0.04 | 0.00 | 0.00 | -0.01 | 0.00 | 0.00 | -0.02 |
| Prepared feathers and down; artificial flowers; articles of human hair | '67 | 0.08 | 0.00 | -0.01 | 0.00 | 0.03 | -0.01 | 0.16 | -0.02 | 0.00 | 0.01 | -0.01 | -0.01 | -0.01 | -0.01 | 0.03 | -0.01 | 0.04 | 0.09 | -0.01 | -0.01 | -0.02 |
| Articles of stone, plaster, cement, mica or similar materials | '68 | -0.11 | 0.04 | 0.18 | 0.00 | -0.18 | -0.10 | 0.19 | -0.04 | 0.03 | -0.03 | 0.01 | -0.06 | 0.02 | 0.26 | 0.02 | 0.00 | 0.18 | -0.11 | -0.06 | 0.15 | 0.07 |
| Ceramic products | '69 | -0.04 | -0.01 | 0.06 | 0.12 | -0.48 | -0.27 | 0.48 | -0.08 | 0.04 | -0.03 | -0.09 | -0.10 | 0.01 | -0.11 | 0.02 | 0.07 | 0.23 | -0.07 | -0.06 | 0.40 | 0.03 |
| Glass and glassware | '70 | -0.14 | 0.08 | -0.10 | 0.52 | -0.19 | -0.13 | 0.17 | -0.02 | 0.19 | -0.05 | -0.06 | 0.01 | 0.00 | -0.16 | -0.05 | 0.06 | 0.02 | -0.04 | -0.13 | 0.02 | 0.07 |
| Natural or cultured pearls, precious stones, imitation jewellery, coin | '71 | -1.07 | 0.21 | 1.02 | 0.10 | 1.12 | 1.20 | -1.10 | 2.74 | -0.08 | 1.68 | 0.41 | 0.20 | -0.25 | 0.00 | -1.94 | 0.01 | -0.10 | 1.46 | -0.18 | 0.09 | -0.08 |
| Iron and steel | '72 | -2.71 | 0.52 | 2.62 | -0.78 | -1.15 | -0.79 | 0.21 | -0.75 | -0.69 | -0.82 | 1.23 | 0.33 | -0.27 | -0.21 | -0.03 | -0.45 | 0.37 | -0.93 | -0.29 | -0.87 | 1.40 |
| Articles of iron or steel | '73 | -1.03 | -0.02 | -0.27 | -0.16 | -0.94 | -0.91 | 1.16 | -0.74 | 0.29 | -0.33 | 0.23 | -0.16 | 0.07 | 0.37 | -0.03 | -0.54 | 0.65 | -0.74 | -0.46 | 1.04 | 0.21 |
| Copper and articles thereof | '74 | -0.20 | -0.04 | -0.14 | 3.03 | -0.03 | 13.75 | -0.89 | -0.04 | -0.25 | -0.05 | 0.51 | -0.01 | -0.04 | 0.35 | -0.03 | -0.23 | -0.38 | 0.17 | -0.06 | -0.37 | 0.36 |
| Nickel and articles thereof | '75 | 0.00 | -0.03 | -0.04 | -0.01 | 0.00 | 0.00 | -0.12 | -0.01 | -0.03 | 0.00 | 0.02 | -0.04 | -0.02 | 0.00 | 0.02 | -0.02 | -0.06 | 0.22 | -0.02 | -0.05 | -0.14 |
| Aluminium and articles thereof | '76 | -0.38 | -0.01 | -0.33 | -0.02 | -0.77 | -0.23 | 0.38 | 0.10 | -0.28 | -0.14 | -0.06 | -0.07 | -0.16 | 1.74 | -0.01 | -0.28 | 0.37 | -0.42 | -0.17 | -0.07 | -0.43 |
| Lead and articles thereof | '78 | -0.04 | 0.03 | -0.03 | 0.22 | 0.00 | 0.02 | -0.01 | -0.02 | -0.06 | 0.00 | -0.01 | 0.01 | -0.01 | -0.03 | 0.03 | -0.01 | 0.00 | -0.04 | -0.01 | -0.01 | 0.00 |
| Zinc and articles thereof | '79 | -0.14 | 0.04 | 0.03 | 0.28 | -0.01 | -0.02 | -0.05 | -0.05 | -0.03 | 0.00 | 0.47 | -0.01 | -0.04 | -0.03 | 0.01 | -0.03 | 0.02 | -0.13 | 0.00 | -0.06 | 0.02 |
| Tin and articles thereof | '80 | -0.01 | 0.01 | 0.05 | -0.01 | 0.00 | 0.00 | 0.00 | -0.01 | -0.01 | 0.00 | 0.00 | 0.00 | -0.02 | 0.00 | 0.01 | -0.02 | -0.02 | 0.39 | 0.00 | -0.01 | -0.03 |
| Other base metals; cermets; articles thereof | '81 | 0.00 | -0.02 | -0.01 | 0.00 | -0.01 | 0.05 | -0.01 | 0.00 | -0.02 | 0.00 | -0.06 | -0.01 | -0.03 | -0.02 | 0.01 | -0.01 | -0.04 | -0.02 | -0.04 | -0.04 | -0.03 |
| Tools, implements, cutlery, spoons and forks, of base metal | '82 | -0.08 | -0.02 | -0.08 | -0.08 | -0.08 | -0.23 | 0.25 | -0.14 | -0.02 | -0.04 | -0.12 | -0.06 | 0.08 | -0.09 | -0.01 | -0.13 | 0.02 | -0.17 | -0.06 | 0.00 | 0.17 |
| Miscellaneous articles of base metal | '83 | -0.19 | -0.07 | -0.81 | 0.01 | -0.33 | -0.17 | 0.35 | -0.17 | 0.04 | -0.06 | -0.06 | -0.10 | 0.06 | -0.02 | -0.02 | -0.10 | 0.01 | -0.22 | -0.05 | 0.15 | -0.04 |
| Machinery, mechanical appliances, nuclear reactors, boilers | '84 | -5.48 | -0.46 | -2.50 | -0.64 | -3.03 | -6.39 | 3.63 | -5.39 | 1.07 | -0.39 | -0.04 | -0.51 | 2.28 | -1.01 | -2.40 | 0.44 | -1.10 | -6.60 | -2.60 | 4.65 | 5.13 |
| Electrical machinery and equipment; sound recorders and reproducers, tv | '85 | -3.11 | -0.93 | -5.42 | 0.72 | -0.63 | -4.65 | 1.04 | -5.05 | -1.01 | -0.84 | -1.22 | -0.55 | -0.91 | -1.18 | -11.2 | 0.71 | -2.75 | -3.43 | 0.19 | -0.96 | 0.42 |
| Railway or tramway locomotives; railway or tramway track | '86 | -0.09 | 0.00 | -0.02 | 0.07 | -0.04 | -0.14 | 0.17 | -0.02 | 0.11 | 0.00 | -0.06 | 0.02 | -0.01 | -0.02 | 0.00 | 0.07 | 0.00 | -0.03 | -0.01 | 0.01 | 0.03 |
| Vehicles other than railway or tramway rolling stock | '87 | -1.56 | -0.52 | -0.83 | -1.66 | -4.25 | -5.70 | -0.39 | -3.96 | 4.98 | -0.87 | -1.10 | -1.03 | 2.70 | -2.07 | -0.13 | 3.99 | 2.23 | 0.32 | -1.95 | -1.33 | 9.53 |
| Aircraft, spacecraft, and parts thereof | '88 | -0.68 | 0.06 | 0.73 | -0.02 | -0.04 | -0.06 | -0.41 | -1.19 | 0.03 | -2.39 | -0.26 | 3.33 | 0.75 | -0.05 | -0.07 | -0.29 | -0.10 | -0.12 | -10.1 | 0.18 | -0.28 |
| Ships, boats and floating structures | '89 | -0.55 | 0.00 | -0.49 | -0.01 | -0.03 | -0.04 | 0.44 | 0.00 | 0.00 | 0.00 | 1.42 | 0.20 | 0.11 | -0.29 | -0.01 | 0.00 | 0.47 | -0.21 | -0.03 | 0.29 | 1.00 |
| Optical, photographic, cinematographic, medical or surgical instruments | '90 | -0.54 | -0.04 | -1.25 | 0.10 | -0.22 | -1.06 | -1.01 | -1.42 | -0.13 | -0.32 | 0.90 | -0.03 | 0.90 | -0.41 | -0.64 | 0.41 | -0.42 | -0.69 | 2.70 | -0.22 | 0.85 |
| Clocks and watches and parts thereof | '91 | -0.02 | 0.00 | -0.05 | -0.06 | 0.01 | -0.03 | 0.00 | -0.06 | -0.03 | 0.00 | -0.03 | 0.03 | -0.04 | -0.06 | -0.29 | -0.02 | -0.02 | -0.07 | -0.03 | -0.03 | -0.19 |
| Musical instruments; parts and accessories of such articles | '92 | 0.00 | 0.00 | -0.02 | 0.00 | 0.00 | -0.02 | 0.02 | -0.02 | 0.00 | 0.00 | -0.03 | -0.01 | -0.01 | -0.01 | 0.00 | 0.00 | 0.00 | 0.14 | -0.01 | 0.00 | 0.01 |
| Arms and ammunition; parts and accessories thereof | '93 | -0.04 | 0.00 | 0.09 | 0.00 | 0.03 | -0.01 | 0.00 | 0.00 | 0.07 | 0.00 | 0.05 | 0.00 | 0.01 | 0.01 | 0.00 | -0.02 | 0.01 | -0.04 | -0.01 | 0.09 | 0.00 |
| Furniture; bedding, mattresses, stuffed furnishings; lamps | '94 | -0.10 | -0.17 | -0.06 | 0.56 | 1.06 | -0.60 | 1.92 | -0.22 | 0.34 | -0.14 | -0.60 | -0.58 | -0.27 | -0.26 | -0.05 | 0.08 | 0.12 | 0.27 | -0.36 | 0.90 | -0.54 |
| Toys, games and sports requisites | '95 | 0.01 | -0.05 | -0.16 | 0.25 | 0.00 | -0.43 | 1.20 | -0.28 | 0.41 | -0.01 | -0.22 | -0.26 | -0.16 | 0.06 | -0.15 | 0.02 | 0.01 | 0.03 | -0.21 | -0.09 | -0.21 |
| Miscellaneous manufactured articles | '96 | -0.41 | 0.03 | -0.05 | -0.10 | -0.51 | -0.18 | 0.30 | -0.01 | 0.16 | -0.04 | -0.12 | -0.03 | 0.02 | -0.01 | -0.04 | 0.09 | 0.04 | -0.07 | -0.06 | 0.02 | 0.14 |
| Works of art, collectors' pieces and antiques | '97 | 0.00 | -0.01 | 0.11 | 0.00 | 0.00 | -0.01 | -0.01 | 0.01 | 0.00 | 0.02 | 0.00 | 0.09 | 0.00 | 0.02 | -0.18 | -0.01 | 0.01 | 0.00 | 0.00 | 0.02 | -0.01 |
| Commodities not elsewhere specified | '99 | -0.31 | 0.06 | 0.00 | 0.50 | 0.01 | 1.69 | -0.03 | -0.44 | 0.03 | 1.81 | -1.46 | 0.01 | 0.60 | -0.21 | -0.03 | -0.18 | -0.17 | -0.14 | -0.82 | 0.57 | 2.52 |

| **Product label** | **Code** | **Kor** | **Mal** | **Mex** | **Neth** | **Nor** | **Paki** | **Phil** | **Pol** | **Port** | **Rom** | **Sing** | **Spa** | **Swe** | **Switz** | **Taiwan** | **Thai** | **Tun** | **Turk** | **UK** | **US** | **Viet** |
| --- | --- | --- | --- | --- | --- | --- | --- | --- | --- | --- | --- | --- | --- | --- | --- | --- | --- | --- | --- | --- | --- | --- |
| Dairy products | '04 | -0.10 | -0.12 | -0.22 | 0.43 | -0.04 | -0.01 | -0.48 | 0.33 | -0.09 | -0.19 | -0.12 | -0.03 | -0.27 | 0.00 | -0.11 | -0.09 | -0.06 | 0.17 | -0.03 | 0.10 | -0.11 |
| Products of animal origin | '05 | -0.02 | 0.00 | -0.03 | 0.01 | -0.02 | 0.08 | -0.01 | 0.02 | 0.03 | -0.02 | 0.00 | 0.02 | 0.00 | -0.01 | -0.01 | 0.00 | 0.00 | 0.01 | 0.00 | 0.02 | -0.07 |
| Lac; gums, resins | '13 | -0.02 | -0.01 | 0.00 | -0.01 | -0.01 | 0.07 | 0.12 | -0.02 | -0.03 | -0.02 | 0.00 | 0.05 | -0.01 | 0.00 | -0.01 | -0.02 | -0.01 | -0.01 | -0.01 | -0.01 | -0.01 |
| Vegetable plaiting materials | '14 | -0.01 | 0.02 | 0.00 | 0.00 | 0.00 | -0.01 | 0.00 | -0.01 | 0.00 | 0.00 | 0.00 | 0.00 | 0.00 | 0.00 | 0.00 | -0.01 | 0.00 | 0.00 | 0.00 | 0.00 | 0.00 |
| Animal or vegetable fats and oils | '15 | -0.12 | 2.00 | -0.09 | -0.11 | -0.46 | -1.61 | 0.30 | -0.12 | 0.22 | 0.07 | -0.09 | 0.40 | -0.07 | -0.06 | -0.05 | 0.04 | 1.42 | -0.02 | -0.07 | -0.03 | -0.12 |
| Preparations of meat, fish, molluscs | '16 | -0.08 | 0.04 | -0.04 | -0.01 | -0.09 | 0.01 | 0.27 | 0.35 | 0.02 | -0.01 | -0.06 | 0.09 | -0.16 | -0.06 | -0.02 | 1.37 | 0.10 | 0.04 | -0.28 | -0.05 | 0.45 |
| Sugars and sugar confectionery | '17 | -0.08 | -0.15 | 0.11 | 0.05 | -0.10 | 0.70 | -0.16 | 0.07 | -0.06 | -0.15 | -0.03 | -0.02 | -0.06 | -0.02 | -0.04 | 0.68 | -0.20 | 0.15 | -0.05 | -0.03 | -0.05 |
| Cocoa and cocoa preparations | '18 | -0.04 | 0.05 | 0.02 | 0.01 | -0.11 | -0.03 | -0.07 | 0.11 | -0.13 | -0.12 | 0.02 | -0.03 | -0.05 | 0.03 | -0.02 | -0.03 | 0.00 | 0.05 | -0.09 | -0.04 | -0.01 |
| Preparations of cereals, flour | '19 | 0.03 | 0.11 | 0.17 | 0.25 | -0.33 | 0.02 | -0.01 | 0.37 | -0.07 | -0.16 | 0.14 | 0.12 | -0.07 | -0.03 | -0.01 | 0.21 | 0.26 | 0.53 | -0.09 | -0.04 | 0.01 |
| Preparations of vegetables, fruit, nuts | '20 | -0.08 | -0.06 | 0.10 | 0.23 | -0.15 | 0.08 | 0.23 | 0.12 | 0.11 | -0.17 | -0.04 | 0.40 | -0.22 | -0.09 | -0.04 | 0.38 | 0.00 | 0.53 | -0.21 | -0.02 | 0.14 |
| Miscellaneous edible preparations | '21 | -0.10 | 0.03 | -0.05 | 0.20 | -0.38 | 0.00 | -0.63 | 0.19 | -0.12 | -0.19 | 0.59 | 0.09 | -0.05 | 0.02 | -0.05 | 0.33 | -0.01 | 0.06 | 0.02 | 0.12 | -0.05 |
| Beverages, spirits and vinegar | '22 | -0.04 | -0.01 | 0.74 | 0.13 | -0.45 | 0.68 | -0.23 | -0.04 | 0.63 | -0.17 | 0.01 | 0.55 | -0.26 | -0.05 | -0.17 | 0.35 | 0.07 | 0.04 | 0.49 | -0.29 | 0.00 |
| Residues and waste from food industries | '23 | -0.21 | -0.17 | -0.16 | 0.15 | -0.29 | 0.03 | -0.69 | -0.15 | -0.13 | -0.14 | 0.02 | -0.13 | -0.10 | -0.06 | -0.10 | -0.09 | -0.02 | -0.33 | -0.07 | 0.26 | -0.62 |
| Tobacco and manufactured tobacco substitutes | '24 | 0.05 | -0.04 | 0.01 | 0.02 | -0.25 | 0.03 | 0.22 | 0.65 | 0.36 | 0.51 | 0.02 | -0.22 | 0.06 | 0.04 | -0.03 | -0.02 | -0.24 | 0.14 | -0.04 | 0.01 | 0.02 |
| Salt; sulphur; earths and stone; lime and cement | '25 | -0.05 | -0.03 | 0.02 | -0.05 | 0.07 | 0.80 | -0.28 | -0.12 | 0.14 | -0.04 | -0.08 | 0.10 | -0.11 | -0.06 | -0.13 | 0.14 | 0.22 | 0.73 | -0.01 | -0.01 | 0.29 |
| Ores, slag and ash | '26 | -1.52 | -0.33 | 0.44 | -0.16 | -0.34 | 0.20 | 0.67 | -0.23 | 0.40 | -0.16 | -0.01 | -0.28 | 0.82 | 0.00 | -0.48 | -0.04 | 0.02 | 0.06 | -0.11 | 0.17 | -0.24 |
| Mineral fuels, mineral oils and products of their distillation | '27 | -9.21 | -0.25 | -1.97 | -1.53 | 29.53 | -11.9 | -5.35 | -3.14 | -2.95 | -1.96 | -4.22 | -3.76 | -2.44 | -1.30 | -6.04 | -6.31 | -5.91 | -8.34 | 0.26 | 2.08 | -2.50 |
| Inorganic chemicals | '28 | -0.35 | -0.38 | -0.21 | 0.11 | -0.58 | -0.41 | -0.17 | -0.08 | -0.23 | -0.02 | -0.09 | -0.05 | -0.43 | -0.05 | -0.18 | -0.28 | 0.97 | 0.22 | 0.03 | 0.10 | -0.09 |
| Organic chemicals | '29 | 0.63 | -0.12 | -0.75 | 0.16 | 0.31 | -2.08 | -0.33 | -0.37 | -0.17 | -0.30 | 0.72 | -0.92 | -0.13 | 1.07 | -0.20 | 0.06 | -0.57 | -1.22 | 0.62 | 0.15 | -0.67 |
| Pharmaceutical products | '30 | -0.37 | -0.35 | -0.39 | 0.96 | -0.93 | -0.24 | -0.77 | -0.58 | -0.81 | -1.41 | 0.65 | -0.09 | 1.74 | 7.49 | -0.68 | -0.41 | -1.06 | -0.82 | 0.86 | -0.88 | -0.66 |
| Fertilisers | '31 | -0.03 | -0.07 | -0.13 | 0.13 | -0.04 | -0.54 | -0.27 | -0.06 | -0.05 | -0.26 | 0.00 | 0.01 | -0.08 | -0.02 | -0.03 | -0.32 | 0.75 | -0.07 | -0.04 | -0.01 | -0.16 |
| Tanning or dyeing extracts; paints and varnishes | '32 | -0.05 | -0.05 | -0.20 | 0.14 | -0.21 | -0.33 | -0.15 | -0.17 | -0.25 | -0.22 | 0.02 | 0.20 | 0.03 | 0.11 | -0.04 | -0.20 | -0.25 | -0.24 | 0.19 | 0.15 | -0.35 |
| Essential oils; perfumery, cosmetics | '33 | 0.40 | -0.20 | -0.13 | 0.02 | -0.34 | -0.11 | -0.40 | 0.21 | -0.33 | -0.20 | 0.63 | 0.37 | -0.16 | 0.33 | -0.19 | 0.11 | 0.00 | -0.01 | 0.14 | 0.14 | -0.11 |
| Soap, washing preparations, waxes, candles | '34 | 0.01 | 0.05 | -0.02 | 0.07 | -0.19 | -0.09 | -0.07 | 0.21 | -0.17 | -0.15 | 0.03 | 0.07 | -0.05 | -0.04 | -0.09 | 0.01 | -0.16 | 0.04 | 0.05 | 0.14 | -0.05 |
| Albuminoidal substances | '35 | -0.01 | -0.04 | -0.09 | 0.10 | -0.03 | -0.03 | -0.13 | -0.04 | 0.01 | -0.08 | -0.01 | -0.05 | -0.01 | 0.03 | -0.01 | 0.12 | -0.13 | -0.05 | -0.01 | 0.06 | -0.14 |
| Explosives; pyrotechnic products; matches | '36 | 0.00 | -0.01 | -0.01 | 0.00 | -0.05 | 0.02 | 0.01 | 0.00 | -0.01 | -0.04 | 0.00 | 0.01 | 0.00 | 0.00 | 0.00 | -0.01 | 0.00 | -0.01 | 0.00 | 0.00 | 0.00 |
| Photographic or cinematographic goods | '37 | -0.01 | 0.01 | -0.02 | 0.05 | -0.02 | -0.02 | -0.02 | -0.02 | -0.02 | -0.02 | -0.03 | -0.02 | -0.01 | -0.01 | -0.16 | -0.03 | -0.03 | -0.03 | -0.02 | 0.04 | -0.03 |
| Miscellaneous chemical products | '38 | -0.44 | -0.06 | -0.55 | 0.11 | -0.24 | -0.63 | -0.46 | -0.11 | -0.61 | -0.60 | 0.26 | 0.01 | 0.06 | -0.13 | -0.55 | -0.60 | -0.40 | -0.31 | 0.29 | 0.68 | -0.43 |
| Plastics and articles thereof | '39 | 1.83 | -0.23 | -1.83 | 0.65 | -1.23 | -1.20 | -0.94 | -0.42 | 0.13 | -1.37 | 0.77 | 0.28 | -0.08 | -0.36 | 1.72 | 0.89 | -1.28 | -1.06 | -0.11 | 0.83 | -2.32 |
| Rubber and articles thereof | '40 | 0.39 | 0.59 | -0.43 | -0.07 | -0.41 | -0.27 | -0.01 | 0.26 | 0.42 | 0.81 | 0.08 | 0.13 | -0.29 | -0.18 | 0.19 | 2.73 | -0.30 | 0.22 | -0.10 | -0.19 | 0.35 |
| Raw hides and skins (other than furskins) and leather | '41 | 0.01 | -0.01 | -0.06 | 0.01 | 0.00 | 0.44 | -0.05 | -0.03 | -0.16 | -0.17 | 0.00 | 0.04 | 0.00 | 0.00 | 0.02 | 0.02 | -0.42 | 0.01 | 0.02 | 0.04 | -0.26 |
| Articles of leather; saddlery; travel goods, handbags | '42 | -0.28 | -0.12 | -0.07 | -0.01 | -0.17 | 1.32 | 0.45 | -0.08 | -0.10 | -0.10 | -0.07 | -0.06 | -0.11 | -0.14 | -0.11 | -0.05 | 0.26 | 0.05 | -0.14 | -0.21 | 0.68 |
| Furskins and artificial fur; manufactures thereof | '43 | -0.02 | 0.00 | 0.00 | 0.00 | 0.02 | 0.00 | 0.00 | 0.03 | 0.00 | 0.00 | 0.00 | 0.01 | 0.00 | -0.01 | 0.00 | -0.02 | 0.00 | 0.04 | 0.00 | 0.00 | -0.03 |
| Wood and articles of wood | '44 | -0.31 | 0.44 | -0.14 | -0.25 | -0.54 | -0.09 | 0.15 | 0.45 | 0.05 | 0.78 | -0.04 | 0.06 | 0.57 | -0.27 | -0.19 | 0.39 | -0.29 | 0.17 | -0.48 | -0.13 | 0.29 |
| Cork and articles of cork | '45 | 0.00 | 0.00 | 0.00 | 0.00 | 0.00 | 0.00 | 0.00 | 0.00 | 0.78 | 0.00 | 0.00 | 0.03 | 0.00 | 0.00 | 0.00 | 0.00 | 0.02 | 0.00 | 0.00 | 0.00 | 0.00 |
| Manufactures of straw, basketware | '46 | 0.00 | 0.00 | 0.00 | 0.00 | -0.01 | 0.00 | 0.02 | 0.00 | -0.01 | 0.00 | 0.00 | 0.00 | 0.00 | 0.00 | 0.00 | 0.00 | -0.01 | 0.00 | -0.01 | -0.01 | 0.05 |
| Pulp of wood or of other fibrous cellulosic material | '47 | -0.17 | -0.06 | -0.11 | -0.02 | 0.12 | -0.15 | 0.05 | -0.10 | 0.49 | -0.04 | 0.03 | -0.02 | 0.78 | -0.01 | -0.10 | -0.12 | -0.18 | -0.03 | 0.01 | 0.20 | -0.13 |
| Paper and paperboard; articles of paper pulp, of paper or of paperboard | '48 | 0.07 | -0.26 | -0.51 | -0.05 | -0.29 | -0.35 | -0.51 | -0.02 | 0.95 | -0.35 | -0.02 | 0.13 | 2.34 | -0.22 | -0.05 | -0.06 | -0.30 | -0.10 | -0.22 | 0.13 | -0.29 |
| Printed books, newspapers, pictures and products of the printing industry | '49 | -0.01 | 0.02 | -0.04 | 0.06 | -0.19 | -0.02 | -0.06 | 0.29 | -0.05 | 0.01 | 0.03 | 0.04 | -0.04 | -0.19 | 0.01 | -0.03 | -0.03 | 0.00 | 0.21 | 0.03 | -0.01 |
| Silk | '50 | 0.00 | 0.00 | 0.00 | 0.00 | 0.00 | -0.01 | 0.00 | 0.00 | -0.01 | 0.01 | 0.00 | 0.00 | 0.00 | 0.00 | 0.00 | 0.00 | -0.07 | -0.01 | 0.00 | 0.00 | 0.01 |
| Wool, fine or coarse animal hair; horsehair yarn and woven fabric | '51 | -0.03 | 0.00 | 0.00 | -0.01 | -0.03 | 0.00 | 0.00 | -0.02 | -0.04 | -0.04 | 0.00 | 0.01 | -0.01 | -0.01 | 0.00 | -0.02 | -0.05 | -0.03 | 0.02 | 0.00 | -0.07 |
| Cotton | '52 | -0.07 | -0.06 | -0.05 | 0.01 | -0.01 | 6.14 | -0.07 | -0.06 | -0.22 | -0.14 | 0.00 | 0.06 | -0.01 | 0.00 | 0.00 | -0.06 | -1.33 | -0.16 | 0.00 | 0.23 | -0.28 |
| Other vegetable textile fibres; paper yarn and woven fabrics of paper yarn | '53 | -0.01 | 0.00 | -0.01 | 0.00 | 0.00 | -0.02 | 0.01 | 0.00 | -0.04 | -0.01 | 0.00 | 0.00 | 0.00 | 0.00 | 0.00 | 0.00 | 0.03 | -0.06 | 0.00 | 0.00 | -0.01 |
| Man-made filaments; strip and the like of man-made textile materials | '54 | 0.15 | 0.00 | -0.10 | 0.02 | -0.02 | -0.48 | -0.09 | -0.12 | -0.18 | -0.24 | 0.02 | 0.02 | -0.01 | 0.00 | 0.36 | -0.02 | -0.44 | -0.09 | 0.00 | 0.01 | -0.48 |
| Man-made staple fibres | '55 | 0.08 | 0.03 | -0.07 | -0.01 | -0.01 | 0.13 | -0.10 | -0.07 | 0.05 | -0.03 | 0.00 | 0.01 | -0.02 | 0.00 | 0.09 | 0.19 | -0.59 | -0.05 | 0.00 | 0.03 | -0.49 |
| Wadding, felt and nonwovens; special yarns; twine, cordage, ropes | '56 | 0.00 | 0.00 | -0.07 | 0.01 | -0.09 | -0.09 | -0.04 | -0.06 | 0.15 | -0.12 | -0.01 | 0.03 | -0.01 | -0.03 | 0.05 | 0.06 | -0.14 | 0.16 | -0.01 | 0.03 | -0.11 |
| Carpets and other textile floor coverings | '57 | -0.01 | -0.02 | -0.01 | 0.08 | -0.06 | 0.13 | -0.01 | 0.00 | 0.02 | -0.04 | -0.01 | -0.01 | -0.05 | -0.02 | -0.01 | 0.02 | -0.02 | 0.76 | -0.05 | -0.03 | 0.02 |
| Special woven fabrics; tufted textile fabrics; lace; tapestries | '58 | 0.02 | -0.01 | -0.03 | 0.00 | -0.01 | 0.02 | -0.04 | -0.04 | 0.06 | -0.04 | 0.00 | 0.01 | 0.00 | 0.00 | 0.09 | 0.01 | -0.15 | 0.07 | 0.00 | 0.00 | -0.20 |
| Impregnated, coated, covered or laminated textile fabrics | '59 | 0.07 | -0.02 | -0.09 | 0.02 | -0.02 | -0.07 | -0.07 | -0.03 | 0.15 | -0.19 | -0.01 | 0.02 | 0.00 | 0.02 | 0.12 | -0.08 | -0.27 | 0.00 | 0.01 | 0.01 | -0.16 |
| Knitted or crocheted fabrics | '60 | 0.24 | 0.03 | -0.07 | 0.00 | -0.01 | 0.00 | -0.13 | -0.02 | 0.03 | -0.11 | 0.00 | 0.04 | 0.01 | 0.00 | 0.34 | -0.02 | -0.33 | 0.36 | 0.00 | 0.01 | -0.90 |
| Articles of apparel and clothing accessories, knitted or crocheted | '61 | -0.33 | 0.00 | -0.05 | -0.20 | -0.63 | 6.36 | 0.24 | -0.20 | 1.07 | -0.05 | -0.08 | -0.29 | -0.44 | -0.42 | -0.14 | 0.24 | 1.58 | 2.65 | -0.53 | -0.87 | 3.05 |
| Articles of apparel and clothing accessories, not knitted or crocheted | '62 | -0.58 | -0.09 | 0.05 | -0.24 | -0.72 | 5.91 | 0.11 | -0.14 | 0.05 | 0.79 | -0.08 | -0.09 | -0.43 | -0.50 | -0.14 | 0.02 | 5.09 | 1.76 | -0.39 | -0.71 | 3.09 |
| Other made-up textile articles; sets; worn clothing | '63 | -0.05 | -0.07 | 0.04 | -0.07 | -0.22 | 8.37 | -0.03 | 0.04 | 0.42 | 0.13 | -0.02 | -0.06 | -0.11 | -0.11 | 0.01 | 0.01 | 0.51 | 0.58 | -0.07 | -0.27 | 0.34 |
| Footwear, gaiters and the like | '64 | -0.29 | -0.12 | -0.09 | -0.13 | -0.41 | 0.22 | -0.12 | -0.15 | 1.02 | 0.32 | -0.04 | -0.02 | -0.25 | -0.26 | -0.11 | -0.01 | 1.08 | 0.16 | -0.24 | -0.51 | 3.72 |
| Headgear and parts thereof | '65 | -0.01 | 0.00 | 0.00 | 0.01 | -0.04 | 0.00 | 0.00 | 0.00 | 0.02 | -0.01 | -0.01 | -0.01 | -0.03 | -0.03 | 0.01 | 0.00 | 0.00 | 0.01 | -0.01 | -0.04 | 0.09 |
| Umbrellas, sun umbrellas, walking sticks | '66 | -0.01 | 0.00 | 0.00 | 0.00 | -0.01 | 0.00 | -0.01 | 0.00 | 0.00 | -0.01 | 0.00 | -0.01 | -0.01 | 0.00 | 0.00 | 0.00 | 0.00 | 0.00 | -0.01 | -0.01 | 0.00 |
| Prepared feathers and down; artificial flowers; articles of human hair | '67 | -0.01 | 0.00 | -0.01 | 0.00 | -0.01 | 0.00 | 0.03 | -0.02 | -0.01 | -0.01 | 0.00 | -0.01 | -0.01 | -0.01 | 0.00 | 0.00 | 0.00 | -0.01 | -0.02 | -0.05 | 0.00 |
| Articles of stone, plaster, cement, mica or similar materials | '68 | -0.08 | 0.06 | 0.01 | -0.03 | -0.24 | -0.01 | -0.06 | 0.14 | 0.29 | -0.12 | -0.08 | 0.25 | -0.14 | -0.10 | -0.06 | 0.04 | -0.03 | 0.34 | -0.03 | -0.04 | 0.00 |
| Ceramic products | '69 | -0.13 | -0.04 | 0.04 | -0.03 | -0.15 | -0.09 | -0.33 | 0.07 | 0.47 | -0.10 | -0.03 | 0.56 | -0.09 | -0.09 | -0.09 | 0.04 | 0.01 | 0.28 | -0.08 | -0.07 | 0.02 |
| Glass and glassware | '70 | -0.07 | 0.04 | 0.02 | -0.05 | -0.20 | -0.06 | -0.10 | 0.16 | 0.17 | -0.16 | -0.10 | 0.01 | -0.16 | -0.11 | 0.01 | -0.03 | -0.04 | 0.16 | -0.04 | 0.01 | -0.10 |
| Natural or cultured pearls, precious stones, imitation jewellery, coin | '71 | -0.14 | -0.43 | 0.71 | -0.01 | 0.16 | 0.00 | 1.01 | 0.10 | 0.10 | 0.18 | -0.28 | 0.09 | 0.30 | -3.03 | -0.29 | 0.73 | -0.04 | -1.17 | -1.98 | 0.71 | 0.33 |
| Iron and steel | '72 | 0.52 | -0.74 | -0.93 | 0.12 | 0.12 | -2.60 | -1.64 | -0.94 | -0.48 | -0.20 | -0.16 | -0.10 | 0.66 | -0.27 | -0.11 | -2.33 | -0.99 | -0.72 | 0.27 | -0.04 | -1.48 |
| Articles of iron or steel | '73 | 0.13 | -0.24 | -0.37 | -0.06 | -2.22 | -0.52 | -0.52 | 0.59 | 0.53 | -0.05 | -0.19 | 0.63 | -0.25 | -0.25 | 0.90 | -0.55 | 0.20 | 1.38 | -0.20 | -0.23 | -0.17 |
| Copper and articles thereof | '74 | -0.12 | -0.27 | -0.12 | 0.00 | -0.06 | 0.59 | 0.66 | 0.39 | -0.15 | -0.25 | -0.01 | 0.23 | 0.09 | -0.07 | -0.23 | -0.46 | -0.66 | -0.29 | 0.04 | 0.04 | -0.40 |
| Nickel and articles thereof | '75 | -0.10 | -0.02 | -0.02 | 0.02 | -0.33 | -0.01 | 0.27 | -0.04 | -0.01 | -0.03 | -0.02 | -0.06 | -0.10 | -0.01 | -0.11 | -0.02 | -0.03 | -0.06 | 0.04 | 0.04 | -0.02 |
| Aluminium and articles thereof | '76 | -0.32 | -0.12 | -0.63 | -0.10 | 1.46 | -0.21 | -0.10 | -0.17 | 0.06 | 0.22 | -0.08 | 0.11 | -0.03 | -0.20 | -0.14 | -0.36 | -0.25 | 0.07 | -0.07 | -0.10 | -0.49 |
| Lead and articles thereof | '78 | 0.04 | 0.02 | 0.02 | 0.00 | -0.02 | 0.00 | -0.04 | -0.01 | -0.02 | 0.00 | 0.00 | -0.04 | 0.05 | 0.00 | -0.03 | -0.05 | 0.01 | -0.06 | 0.02 | -0.02 | -0.08 |
| Zinc and articles thereof | '79 | 0.13 | -0.05 | 0.05 | 0.00 | 0.30 | -0.03 | -0.01 | -0.01 | -0.05 | -0.05 | -0.01 | 0.24 | 0.01 | -0.01 | -0.10 | -0.09 | -0.04 | -0.19 | -0.01 | -0.04 | -0.10 |
| Tin and articles thereof | '80 | -0.02 | 0.06 | -0.01 | 0.00 | 0.00 | -0.01 | 0.00 | 0.00 | 0.00 | -0.01 | -0.02 | 0.00 | 0.00 | 0.00 | -0.01 | 0.02 | 0.00 | -0.02 | -0.01 | -0.01 | -0.02 |
| Other base metals; cermets; articles thereof | '81 | -0.09 | -0.03 | -0.03 | 0.00 | 0.06 | -0.01 | -0.02 | -0.02 | -0.02 | -0.03 | -0.01 | -0.02 | -0.06 | -0.02 | -0.07 | -0.01 | -0.02 | -0.04 | 0.03 | 0.04 | -0.01 |
| Tools, implements, cutlery, spoons and forks, of base metal | '82 | 0.11 | -0.07 | -0.15 | -0.03 | -0.19 | 0.16 | -0.10 | 0.03 | -0.01 | -0.18 | 0.01 | 0.00 | 0.17 | 0.11 | 0.34 | -0.15 | -0.13 | -0.06 | -0.03 | -0.07 | 0.01 |
| Miscellaneous articles of base metal | '83 | 0.07 | -0.04 | -0.03 | -0.06 | -0.15 | -0.06 | -0.03 | 0.02 | 0.02 | -0.10 | -0.01 | 0.01 | -0.09 | -0.07 | 0.20 | 0.02 | -0.18 | 0.12 | -0.07 | -0.10 | -0.11 |
| Machinery, mechanical appliances, nuclear reactors, boilers | '84 | 1.24 | -0.65 | 0.19 | 0.92 | -4.58 | -3.58 | 1.89 | 0.65 | -1.76 | -0.10 | -0.26 | -0.97 | 1.12 | 0.07 | -1.10 | 2.25 | -2.71 | -0.49 | 1.68 | -1.10 | -2.19 |
| Electrical machinery and equipment; sound recorders and reproducers, tv | '85 | 4.96 | 3.20 | -2.00 | -1.37 | -3.65 | -3.64 | 12.41 | -0.80 | -0.63 | 1.51 | 2.41 | -1.15 | -1.85 | -0.66 | 8.11 | -2.06 | 6.16 | -1.18 | -1.49 | -1.60 | 3.88 |
| Railway or tramway locomotives; railway or tramway track | '86 | 0.01 | -0.02 | 0.30 | -0.04 | -0.13 | -0.06 | -0.03 | 0.20 | -0.01 | 0.06 | -0.03 | 0.35 | -0.09 | 0.04 | -0.02 | -0.09 | -0.34 | -0.01 | -0.12 | 0.07 | -0.01 |
| Vehicles other than railway or tramway rolling stock | '87 | 4.09 | -1.17 | 8.95 | -0.74 | -5.73 | -1.18 | -3.05 | 0.80 | 1.24 | 3.81 | -0.29 | 2.81 | 1.29 | -2.50 | -0.04 | 3.94 | -1.10 | 5.55 | -0.07 | -2.01 | -0.71 |
| Aircraft, spacecraft, and parts thereof | '88 | -0.15 | -0.33 | 0.07 | -0.13 | -0.55 | 0.04 | -0.70 | -0.16 | -1.38 | 0.15 | -0.61 | 0.65 | -0.11 | -0.14 | -0.41 | -0.28 | 0.97 | -0.56 | 1.18 | 3.58 | -0.01 |
| Ships, boats and floating structures | '89 | 1.62 | 0.01 | 0.09 | 0.14 | -0.25 | -0.35 | 0.33 | 0.13 | 0.04 | 0.31 | 0.07 | 0.00 | -0.03 | -0.06 | 0.01 | 0.14 | 0.14 | 0.04 | 0.12 | 0.03 | 0.07 |
| Optical, photographic, cinematographic, medical or surgical instruments | '90 | 0.04 | 0.70 | 0.37 | 0.56 | -0.78 | 0.30 | 0.50 | -0.16 | 0.28 | 0.62 | 1.07 | -0.70 | -0.07 | 1.24 | 0.18 | -0.22 | 1.09 | -0.84 | 0.73 | 0.92 | -0.78 |
| Clocks and watches and parts thereof | '91 | -0.10 | -0.08 | -0.03 | -0.03 | -0.05 | -0.01 | 0.07 | -0.03 | -0.02 | -0.02 | -0.06 | -0.04 | -0.03 | 2.79 | -0.07 | 0.05 | 0.08 | -0.05 | -0.03 | -0.05 | -0.02 |
| Musical instruments; parts and accessories of such articles | '92 | -0.01 | 0.03 | 0.00 | 0.00 | -0.03 | 0.01 | -0.01 | -0.01 | -0.02 | 0.00 | -0.01 | -0.01 | 0.00 | -0.01 | 0.02 | -0.01 | 0.01 | 0.00 | -0.01 | -0.01 | 0.00 |
| Arms and ammunition; parts and accessories thereof | '93 | -0.02 | 0.00 | 0.00 | -0.02 | 0.03 | 0.03 | -0.07 | -0.02 | 0.02 | 0.03 | 0.00 | 0.06 | 0.02 | 0.01 | 0.01 | -0.12 | -0.03 | 0.06 | 0.05 | 0.14 | 0.00 |
| Furniture; bedding, mattresses, stuffed furnishings; lamps | '94 | -0.19 | 0.38 | 0.74 | -0.31 | -1.56 | 0.08 | -0.03 | 2.22 | 0.89 | 1.31 | -0.14 | 0.00 | -0.26 | -0.70 | 0.15 | 0.01 | 0.42 | 0.88 | -0.41 | -1.02 | 1.77 |
| Toys, games and sports requisites | '95 | -0.19 | -0.03 | -0.11 | -0.04 | -0.33 | 0.43 | -0.09 | -0.12 | -0.22 | -0.22 | -0.03 | -0.10 | -0.19 | -0.18 | 0.23 | 0.08 | 0.16 | -0.03 | -0.20 | -0.48 | 0.34 |
| Miscellaneous manufactured articles | '96 | -0.05 | -0.01 | 0.03 | 0.02 | -0.15 | -0.09 | -0.12 | 0.13 | -0.09 | -0.14 | -0.01 | -0.03 | 0.07 | -0.02 | 0.00 | 0.04 | 0.21 | 0.17 | -0.10 | -0.06 | -0.11 |
| Works of art, collectors' pieces and antiques | '97 | -0.03 | -0.01 | 0.00 | 0.00 | -0.04 | -0.02 | 0.00 | 0.00 | 0.00 | 0.00 | 0.00 | 0.01 | -0.01 | -0.15 | -0.02 | -0.01 | 0.00 | 0.00 | 1.10 | 0.17 | 0.00 |
| Commodities not elsewhere specified | '99 | 0.00 | -0.09 | -1.24 | 0.22 | 2.02 | -0.12 | -0.40 | -0.76 | 0.07 | 0.19 | 2.32 | 1.06 | -0.37 |  | -0.40 | 0.00 | 0.00 | 0.05 | 0.45 | -0.74 | 0.17 |

**Table S 7. Correlation Lafay RSACA**

| Product label | Code | Corr Lafay RSACA 2001 | Corr Lafay RSACA 2019 |
| --- | --- | --- | --- |
| Dairy products | '04 | 0.87 | 0.61 |
| Products of animal origin | '05 | 0.90 | 0.47 |
| Lac; gums, resins | '13 | 0.68 | 0.68 |
| Vegetable plaiting materials | '14 | 0.42 | 0.43 |
| Animal or vegetable fats and oils | '15 | 0.75 | 0.74 |
| Preparations of meat, fish, molluscs | '16 | 0.72 | 0.79 |
| Sugars and sugar confectionery | '17 | 0.79 | 0.85 |
| Cocoa and cocoa preparations | '18 | 0.88 | 0.93 |
| Preparations of cereals, flour | '19 | 0.94 | 0.94 |
| Preparations of vegetables, fruit, nuts | '20 | 0.74 | 0.73 |
| Miscellaneous edible preparations | '21 | 0.87 | 0.88 |
| Beverages, spirits and vinegar | '22 | 0.82 | 0.84 |
| Residues and waste from food industries | '23 | 0.81 | 0.82 |
| Tobacco and manufactured tobacco substitutes | '24 | 0.68 | 0.79 |
| Salt; sulphur; earths and stone; lime and cement | '25 | 0.92 | 0.87 |
| Ores, slag and ash | '26 | 0.60 | 0.60 |
| Mineral fuels, mineral oils and products of their distillation | '27 | 0.89 | 0.87 |
| Inorganic chemicals | '28 | 0.78 | 0.77 |
| Organic chemicals | '29 | 0.78 | 0.66 |
| Pharmaceutical products | '30 | 0.81 | 0.61 |
| Fertilisers | '31 | 0.84 | 0.70 |
| Tanning or dyeing extracts; paints and varnishes | '32 | 0.88 | 0.91 |
| Essential oils; perfumery, cosmetics | '33 | 0.76 | 0.78 |
| Soap, washing preparations, waxes, candles | '34 | 0.91 | 0.89 |
| Albuminoidal substances | '35 | 0.91 | 0.93 |
| Explosives; pyrotechnic products; matches | '36 | 0.88 | 0.75 |
| Photographic or cinematographic goods | '37 | 0.91 | 0.80 |
| Miscellaneous chemical products | '38 | 0.92 | 0.88 |
| Plastics and articles thereof | '39 | 0.88 | 0.92 |
| Rubber and articles thereof | '40 | 0.83 | 0.73 |
| Raw hides and skins (other than furskins) and leather | '41 | 0.74 | 0.69 |
| Articles of leather; saddlery; travel goods, handbags | '42 | 0.87 | 0.71 |
| Furskins and artificial fur; manufactures thereof | '43 | 0.19 | 0.33 |
| Wood and articles of wood | '44 | 0.89 | 0.83 |
| Cork and articles of cork | '45 | 0.23 | 0.27 |
| Manufactures of straw, basketware | '46 | 0.68 | 0.72 |
| Pulp of wood or of other fibrous cellulosic material | '47 | 0.72 | 0.72 |
| Paper and paperboard; articles of paper pulp, of paper or of paperboard | '48 | 0.71 | 0.70 |
| Printed books, newspapers, pictures and products of the printing industry | '49 | 0.68 | 0.78 |
| Silk | '50 | 0.59 | 0.66 |
| Wool, fine or coarse animal hair; horsehair yarn and woven fabric | '51 | 0.64 | 0.78 |
| Cotton | '52 | 0.48 | 0.50 |
| Other vegetable textile fibres; paper yarn and woven fabrics of paper yarn | '53 | 0.55 | 0.31 |
| Man-made filaments; strip and the like of man-made textile materials | '54 | 0.75 | 0.84 |
| Man-made staple fibres | '55 | 0.58 | 0.68 |
| Wadding, felt and nonwovens; special yarns; twine, cordage, ropes | '56 | 0.92 | 0.87 |
| Carpets and other textile floor coverings | '57 | 0.76 | 0.69 |
| Special woven fabrics; tufted textile fabrics; lace; tapestries | '58 | 0.87 | 0.81 |
| Impregnated, coated, covered or laminated textile fabrics | '59 | 0.86 | 0.79 |
| Knitted or crocheted fabrics | '60 | 0.49 | 0.41 |
| Articles of apparel and clothing accessories, knitted or crocheted | '61 | 0.54 | 0.67 |
| Articles of apparel and clothing accessories, not knitted or crocheted | '62 | 0.47 | 0.66 |
| Other made-up textile articles; sets; worn clothing | '63 | 0.38 | 0.39 |
| Footwear, gaiters and the like | '64 | 0.58 | 0.73 |
| Headgear and parts thereof | '65 | 0.70 | 0.69 |
| Umbrellas, sun umbrellas, walking sticks | '66 | 0.76 | 0.80 |
| Prepared feathers and down; artificial flowers; articles of human hair | '67 | 0.74 | 0.81 |
| Articles of stone, plaster, cement, mica or similar materials | '68 | 0.90 | 0.92 |
| Ceramic products | '69 | 0.86 | 0.93 |
| Glass and glassware | '70 | 0.74 | 0.85 |
| Natural or cultured pearls, precious stones, imitation jewellery, coin | '71 | 0.81 | 0.81 |
| Iron and steel | '72 | 0.90 | 0.88 |
| Articles of iron or steel | '73 | 0.87 | 0.89 |
| Copper and articles thereof | '74 | 0.70 | 0.71 |
| Nickel and articles thereof | '75 | 0.45 | 0.70 |
| Aluminium and articles thereof | '76 | 0.83 | 0.77 |
| Lead and articles thereof | '78 | 0.53 | 0.60 |
| Zinc and articles thereof | '79 | 0.76 | 0.91 |
| Tin and articles thereof | '80 | 0.84 | 0.66 |
| Other base metals; cermets; articles thereof | '81 | 0.91 | 0.83 |
| Tools, implements, cutlery, spoons and forks, of base metal | '82 | 0.91 | 0.93 |
| Miscellaneous articles of base metal | '83 | 0.88 | 0.70 |
| Machinery, mechanical appliances, nuclear reactors, boilers | '84 | 0.93 | 0.92 |
| Electrical machinery and equipment; sound recorders and reproducers, tv | '85 | 0.84 | 0.85 |
| Railway or tramway locomotives; railway or tramway track | '86 | 0.88 | 0.88 |
| Vehicles other than railway or tramway rolling stock | '87 | 0.90 | 0.90 |
| Aircraft, spacecraft, and parts thereof | '88 | 0.86 | 0.56 |
| Ships, boats and floating structures | '89 | 0.80 | 0.85 |
| Optical, photographic, cinematographic, medical or surgical instruments | '90 | 0.88 | 0.87 |
| Clocks and watches and parts thereof | '91 | 0.43 | 0.37 |
| Musical instruments; parts and accessories of such articles | '92 | 0.81 | 0.61 |
| Arms and ammunition; parts and accessories thereof | '93 | 0.86 | 0.70 |
| Furniture; bedding, mattresses, stuffed furnishings; lamps | '94 | 0.82 | 0.91 |
| Toys, games and sports requisites | '95 | 0.86 | 0.89 |
| Miscellaneous manufactured articles | '96 | 0.87 | 0.90 |
| Works of art, collectors' pieces and antiques | '97 | 0.57 | 0.40 |
| Commodities not elsewhere specified | '99 | 0.54 | 0.85 |
| Parts and accessories suitable for calculating machines and calculators | 8473 | 0.84 | 0.48 |
| Electronic integrated circuits; parts thereof | 8542 | 0.65 | 0.46 |
| Parts and accessories for tractors, motor vehicles for the transport of persons, … | 8708 | 0.86 | 0.82 |
| Parts of aircraft and spacecraft of heading 8801 or 8802, n.e.s. | 8803 | 0.78 | 0.62 |
| Parts of telephone sets, telephones for cellular networks or for other wireless networks and ... | 851770 | 0.71 | 0.40 |

**Table S 8. Correlation among Revealed Symmetric Augmented Comparative Advantages 2001**

| Correlation | BANG | BELG | BRAZIL | BULG | CAMB | CHILE | CHIN | COL | CZECH | ETH | FIN | FRAN | GERM | GRE | HON_K | HUNG | INDIA | INDO | IREL | ITALY | JAP |
| --- | --- | --- | --- | --- | --- | --- | --- | --- | --- | --- | --- | --- | --- | --- | --- | --- | --- | --- | --- | --- | --- |
| BANG | 1 |  |  |  |  |  |  |  |  |  |  |  |  |  |  |  |  |  |  |  |  |
| BELG | -0.15 | 1.00 |  |  |  |  |  |  |  |  |  |  |  |  |  |  |  |  |  |  |  |
| BRAZIL | 0.13 | 0.01 | 1.00 |  |  |  |  |  |  |  |  |  |  |  |  |  |  |  |  |  |  |
| BULG | 0.09 | 0.00 | 0.07 | 1.00 |  |  |  |  |  |  |  |  |  |  |  |  |  |  |  |  |  |
| CAMB | 0.41 | -0.11 | 0.05 | 0.29 | 1.00 |  |  |  |  |  |  |  |  |  |  |  |  |  |  |  |  |
| CHILE | 0.06 | -0.14 | 0.32 | 0.20 | 0.22 | 1.00 |  |  |  |  |  |  |  |  |  |  |  |  |  |  |  |
| CHIN | 0.40 | 0.02 | 0.03 | 0.32 | 0.23 | -0.23 | 1.00 |  |  |  |  |  |  |  |  |  |  |  |  |  |  |
| COL | 0.29 | -0.03 | 0.15 | -0.03 | -0.03 | -0.10 | 0.13 | 1.00 |  |  |  |  |  |  |  |  |  |  |  |  |  |
| CZECH | 0.02 | 0.02 | 0.12 | 0.16 | 0.08 | -0.02 | 0.31 | -0.04 | 1.00 |  |  |  |  |  |  |  |  |  |  |  |  |
| ETH | 0.26 | -0.26 | 0.26 | -0.04 | 0.18 | 0.37 | -0.04 | 0.14 | -0.32 | 1.00 |  |  |  |  |  |  |  |  |  |  |  |
| FIN | -0.32 | 0.01 | -0.15 | 0.06 | -0.03 | 0.03 | -0.30 | -0.07 | 0.12 | -0.17 | 1.00 |  |  |  |  |  |  |  |  |  |  |
| FRAN | -0.31 | 0.13 | -0.04 | -0.20 | -0.16 | 0.08 | -0.14 | -0.11 | 0.23 | -0.06 | 0.13 | 1.00 |  |  |  |  |  |  |  |  |  |
| GERM | -0.38 | 0.19 | -0.25 | -0.36 | -0.37 | -0.15 | -0.27 | -0.08 | 0.17 | -0.25 | 0.22 | 0.52 | 1.00 |  |  |  |  |  |  |  |  |
| GRE | 0.00 | 0.14 | 0.16 | 0.12 | -0.17 | 0.22 | -0.14 | 0.30 | -0.20 | 0.27 | -0.17 | -0.04 | 0.03 | 1.00 |  |  |  |  |  |  |  |
| HON_K | 0.30 | -0.10 | -0.13 | 0.00 | 0.21 | -0.14 | 0.17 | 0.08 | 0.04 | 0.02 | 0.13 | -0.38 | -0.18 | -0.07 | 1.00 |  |  |  |  |  |  |
| HUNG | 0.30 | -0.14 | 0.30 | 0.37 | 0.34 | 0.22 | 0.41 | 0.11 | 0.32 | 0.12 | -0.20 | 0.05 | -0.15 | -0.02 | -0.12 | 1.00 |  |  |  |  |  |
| INDIA | 0.34 | 0.11 | 0.31 | -0.01 | -0.02 | -0.11 | 0.39 | 0.26 | 0.15 | 0.12 | -0.49 | 0.09 | -0.09 | 0.13 | 0.11 | 0.32 | 1.00 |  |  |  |  |
| INDO | 0.46 | -0.09 | 0.21 | 0.22 | 0.36 | 0.07 | 0.33 | 0.30 | 0.05 | 0.12 | -0.28 | -0.50 | -0.54 | 0.08 | 0.18 | 0.34 | 0.22 | 1.00 |  |  |  |
| IREL | -0.06 | -0.12 | -0.08 | -0.14 | -0.11 | 0.25 | -0.16 | -0.13 | -0.12 | 0.18 | -0.06 | 0.29 | 0.10 | -0.07 | -0.08 | 0.02 | 0.07 | -0.23 | 1.00 |  |  |
| ITALY | 0.12 | 0.09 | -0.07 | -0.14 | -0.04 | -0.26 | 0.36 | 0.20 | 0.39 | -0.24 | -0.10 | 0.33 | 0.29 | 0.05 | -0.04 | 0.15 | 0.27 | 0.06 | -0.14 | 1.00 |  |
| JAP | -0.40 | 0.13 | -0.39 | -0.35 | -0.29 | -0.35 | -0.30 | -0.24 | 0.09 | -0.33 | 0.34 | 0.38 | 0.67 | -0.23 | -0.09 | -0.33 | -0.25 | -0.50 | -0.02 | 0.25 | 1.00 |
| KOR | 0.09 | 0.15 | -0.23 | -0.10 | -0.09 | -0.35 | 0.27 | 0.13 | 0.27 | -0.27 | 0.08 | 0.13 | 0.33 | -0.14 | 0.18 | -0.01 | 0.24 | -0.06 | -0.13 | 0.49 | 0.44 |
| MAL | 0.22 | 0.00 | 0.07 | 0.12 | 0.31 | -0.09 | 0.23 | 0.21 | 0.25 | -0.15 | -0.16 | -0.25 | -0.19 | -0.01 | 0.11 | 0.28 | 0.01 | 0.59 | -0.14 | 0.26 | -0.20 |
| MEX | 0.27 | -0.25 | 0.10 | 0.39 | 0.10 | -0.09 | 0.41 | 0.17 | 0.34 | -0.07 | -0.21 | 0.00 | -0.16 | 0.00 | -0.01 | 0.38 | 0.20 | 0.28 | -0.21 | 0.29 | -0.14 |
| NETH | -0.23 | 0.22 | -0.05 | -0.09 | -0.32 | -0.02 | -0.20 | -0.05 | -0.07 | -0.12 | 0.11 | 0.18 | 0.33 | 0.11 | -0.25 | -0.05 | -0.12 | -0.44 | 0.21 | -0.06 | 0.16 |
| NOR | -0.22 | -0.26 | -0.03 | 0.05 | -0.10 | 0.18 | -0.34 | -0.02 | -0.20 | 0.25 | 0.42 | -0.04 | -0.12 | 0.06 | -0.07 | -0.16 | -0.39 | -0.23 | 0.22 | -0.37 | -0.02 |
| PAKI | 0.38 | 0.02 | 0.25 | 0.16 | 0.10 | -0.01 | 0.50 | 0.25 | 0.24 | 0.23 | -0.29 | -0.01 | -0.25 | 0.18 | 0.24 | 0.34 | 0.61 | 0.29 | 0.02 | 0.27 | -0.39 |
| PHIL | 0.51 | -0.14 | 0.13 | 0.22 | 0.34 | 0.03 | 0.52 | 0.14 | 0.20 | 0.13 | -0.16 | -0.24 | -0.39 | -0.06 | 0.22 | 0.49 | 0.25 | 0.56 | -0.21 | 0.18 | -0.35 |
| POL | 0.24 | -0.07 | 0.14 | 0.58 | 0.32 | 0.20 | 0.39 | 0.23 | 0.27 | -0.03 | 0.12 | -0.07 | -0.19 | -0.05 | -0.08 | 0.60 | 0.04 | 0.25 | -0.18 | 0.02 | -0.29 |
| PORT | 0.19 | -0.10 | 0.33 | 0.20 | 0.14 | 0.09 | 0.04 | 0.11 | 0.28 | 0.01 | -0.05 | -0.28 | -0.30 | 0.20 | 0.07 | 0.27 | 0.16 | 0.39 | -0.20 | 0.18 | -0.21 |
| ROM | 0.20 | -0.02 | -0.01 | 0.65 | 0.49 | 0.08 | 0.33 | -0.08 | 0.31 | 0.04 | 0.11 | -0.19 | -0.31 | -0.13 | 0.10 | 0.46 | -0.11 | 0.29 | -0.27 | -0.01 | -0.24 |
| SING | 0.02 | -0.01 | -0.13 | -0.13 | 0.25 | 0.12 | -0.30 | -0.06 | -0.23 | 0.20 | 0.01 | -0.19 | -0.03 | 0.07 | 0.40 | -0.11 | -0.09 | 0.05 | 0.29 | -0.16 | 0.01 |
| SPA | -0.21 | -0.04 | 0.12 | 0.07 | -0.12 | 0.02 | 0.11 | -0.05 | 0.32 | -0.09 | 0.11 | 0.20 | 0.18 | 0.11 | -0.23 | 0.19 | 0.10 | -0.06 | -0.06 | 0.42 | 0.13 |
| SWE | -0.27 | -0.01 | -0.13 | -0.15 | -0.18 | 0.06 | -0.33 | -0.03 | 0.15 | -0.10 | 0.42 | 0.15 | 0.27 | -0.11 | 0.06 | -0.32 | -0.35 | -0.24 | 0.05 | 0.03 | 0.30 |
| SWITZ | -0.31 | 0.11 | -0.22 | -0.44 | -0.35 | -0.04 | -0.37 | -0.16 | -0.14 | -0.10 | 0.06 | 0.31 | 0.50 | -0.01 | 0.01 | -0.38 | 0.00 | -0.43 | 0.41 | -0.01 | 0.36 |
| TAIW | 0.22 | 0.06 | -0.18 | -0.26 | -0.03 | -0.34 | 0.35 | 0.12 | 0.26 | -0.13 | -0.05 | -0.01 | 0.15 | -0.09 | 0.36 | 0.01 | 0.29 | 0.11 | -0.12 | 0.52 | 0.23 |
| THAI | 0.28 | 0.04 | 0.37 | 0.17 | 0.12 | -0.10 | 0.57 | 0.28 | 0.18 | 0.02 | -0.22 | -0.14 | -0.37 | -0.04 | 0.11 | 0.41 | 0.44 | 0.56 | -0.09 | 0.25 | -0.35 |
| TUN | 0.43 | -0.10 | 0.03 | 0.44 | 0.21 | 0.00 | 0.45 | 0.15 | 0.02 | -0.01 | -0.24 | -0.45 | -0.54 | 0.04 | 0.14 | 0.37 | 0.16 | 0.48 | -0.16 | 0.03 | -0.53 |
| TURK | 0.22 | 0.10 | 0.41 | 0.17 | 0.06 | 0.07 | 0.33 | 0.47 | 0.22 | 0.08 | -0.29 | 0.01 | -0.13 | 0.34 | -0.09 | 0.47 | 0.54 | 0.39 | -0.27 | 0.33 | -0.24 |
| UK | -0.37 | 0.10 | -0.31 | -0.23 | -0.37 | -0.19 | -0.33 | 0.05 | -0.05 | -0.26 | 0.18 | 0.40 | 0.58 | 0.09 | -0.16 | -0.34 | -0.21 | -0.59 | 0.28 | 0.15 | 0.50 |
| US | -0.42 | 0.21 | 0.02 | -0.33 | -0.29 | 0.21 | -0.59 | -0.23 | -0.13 | -0.01 | 0.06 | 0.33 | 0.57 | 0.19 | -0.26 | -0.22 | -0.15 | -0.54 | 0.25 | -0.13 | 0.33 |
| VIET | 0.54 | -0.19 | 0.30 | 0.18 | 0.35 | 0.06 | 0.59 | 0.27 | 0.13 | 0.17 | -0.44 | -0.28 | -0.58 | 0.01 | 0.06 | 0.48 | 0.35 | 0.69 | -0.11 | 0.19 | -0.57 |

| Correlation | KOR | MAL | MEX | NETH | NOR | PAKI | PHIL | POL | PORT | ROM | SING | SPA | SWE | SWITZ | TAIW | THAI | TUN | TURK | UK | US | VIET |
| --- | --- | --- | --- | --- | --- | --- | --- | --- | --- | --- | --- | --- | --- | --- | --- | --- | --- | --- | --- | --- | --- |
| KOR | 1.00 |  |  |  |  |  |  |  |  |  |  |  |  |  |  |  |  |  |  |  |  |
| MAL | 0.04 | 1.00 |  |  |  |  |  |  |  |  |  |  |  |  |  |  |  |  |  |  |  |
| MEX | 0.12 | 0.22 | 1.00 |  |  |  |  |  |  |  |  |  |  |  |  |  |  |  |  |  |  |
| NETH | 0.02 | -0.23 | -0.25 | 1.00 |  |  |  |  |  |  |  |  |  |  |  |  |  |  |  |  |  |
| NOR | -0.17 | -0.20 | -0.14 | 0.01 | 1.00 |  |  |  |  |  |  |  |  |  |  |  |  |  |  |  |  |
| PAKI | 0.25 | 0.22 | 0.20 | -0.08 | -0.24 | 1.00 |  |  |  |  |  |  |  |  |  |  |  |  |  |  |  |
| PHIL | 0.07 | 0.41 | 0.32 | -0.30 | -0.22 | 0.45 | 1.00 |  |  |  |  |  |  |  |  |  |  |  |  |  |  |
| POL | 0.06 | 0.10 | 0.46 | -0.12 | -0.03 | 0.11 | 0.34 | 1.00 |  |  |  |  |  |  |  |  |  |  |  |  |  |
| PORT | 0.05 | 0.24 | 0.26 | -0.19 | -0.07 | 0.22 | 0.28 | 0.13 | 1.00 |  |  |  |  |  |  |  |  |  |  |  |  |
| ROM | 0.03 | 0.20 | 0.37 | -0.31 | 0.07 | 0.08 | 0.44 | 0.58 | 0.25 | 1.00 |  |  |  |  |  |  |  |  |  |  |  |
| SING | -0.12 | 0.21 | -0.22 | -0.01 | 0.01 | -0.09 | -0.03 | -0.26 | 0.02 | -0.08 | 1.00 |  |  |  |  |  |  |  |  |  |  |
| SPA | 0.20 | 0.01 | 0.22 | 0.08 | -0.05 | 0.12 | 0.04 | 0.11 | 0.23 | 0.00 | -0.20 | 1.00 |  |  |  |  |  |  |  |  |  |
| SWE | 0.08 | -0.05 | -0.19 | -0.11 | 0.10 | -0.26 | -0.20 | -0.18 | 0.06 | -0.06 | 0.04 | 0.01 | 1.00 |  |  |  |  |  |  |  |  |
| SWITZ | 0.04 | -0.35 | -0.42 | 0.28 | -0.04 | -0.17 | -0.44 | -0.42 | -0.38 | -0.54 | 0.14 | -0.17 | 0.25 | 1.00 |  |  |  |  |  |  |  |
| TAIW | 0.69 | 0.21 | -0.01 | -0.21 | -0.18 | 0.38 | 0.14 | -0.04 | 0.09 | -0.04 | -0.03 | 0.17 | 0.03 | -0.02 | 1.00 |  |  |  |  |  |  |
| THAI | 0.20 | 0.41 | 0.27 | -0.19 | -0.27 | 0.53 | 0.54 | 0.29 | 0.26 | 0.10 | -0.11 | 0.13 | -0.33 | -0.44 | 0.25 | 1.00 |  |  |  |  |  |
| TUN | -0.09 | 0.24 | 0.30 | -0.02 | -0.25 | 0.24 | 0.50 | 0.38 | 0.26 | 0.43 | -0.05 | 0.00 | -0.31 | -0.44 | -0.07 | 0.33 | 1.00 |  |  |  |  |
| TURK | 0.26 | 0.17 | 0.31 | -0.10 | -0.35 | 0.52 | 0.29 | 0.38 | 0.43 | 0.09 | -0.32 | 0.31 | -0.32 | -0.30 | 0.22 | 0.50 | 0.26 | 1.00 |  |  |  |
| UK | 0.05 | -0.23 | -0.08 | 0.39 | 0.13 | -0.32 | -0.52 | -0.30 | -0.33 | -0.36 | 0.06 | -0.02 | 0.16 | 0.42 | -0.13 | -0.48 | -0.37 | -0.27 | 1.00 |  |  |
| US | -0.17 | -0.28 | -0.47 | 0.46 | 0.01 | -0.25 | -0.48 | -0.41 | -0.11 | -0.39 | 0.19 | 0.05 | 0.21 | 0.56 | -0.24 | -0.48 | -0.42 | -0.17 | 0.46 | 1.00 |  |
| VIET | -0.09 | 0.42 | 0.42 | -0.29 | -0.24 | 0.43 | 0.65 | 0.38 | 0.28 | 0.22 | -0.07 | 0.01 | -0.42 | -0.49 | 0.07 | 0.65 | 0.55 | 0.43 | -0.52 | -0.60 | 1 |

**Table S 9. Correlation among Revealed Symmetric Augmented Comparative Advantages 2019**

| Correlation | BANG | BELG | BRAZIL | BULG | CAMB | CHILE | CHIN | COL | CZECH | ETH | FIN | FRAN | GERM | GRE | HON_K | HUNG | INDIA | INDO | IREL | ITALY | JAP |
| --- | --- | --- | --- | --- | --- | --- | --- | --- | --- | --- | --- | --- | --- | --- | --- | --- | --- | --- | --- | --- | --- |
| BANG | 1.00 |  |  |  |  |  |  |  |  |  |  |  |  |  |  |  |  |  |  |  |  |
| BELG | -0.27 | 1.00 |  |  |  |  |  |  |  |  |  |  |  |  |  |  |  |  |  |  |  |
| BRAZIL | 0.01 | -0.03 | 1.00 |  |  |  |  |  |  |  |  |  |  |  |  |  |  |  |  |  |  |
| BULG | 0.18 | -0.03 | 0.09 | 1.00 |  |  |  |  |  |  |  |  |  |  |  |  |  |  |  |  |  |
| CAMB | 0.62 | -0.25 | -0.04 | 0.30 | 1.00 |  |  |  |  |  |  |  |  |  |  |  |  |  |  |  |  |
| CHILE | -0.06 | -0.04 | 0.44 | 0.14 | 0.01 | 1.00 |  |  |  |  |  |  |  |  |  |  |  |  |  |  |  |
| CHIN | 0.25 | -0.17 | -0.38 | -0.25 | 0.10 | -0.52 | 1.00 |  |  |  |  |  |  |  |  |  |  |  |  |  |  |
| COL | 0.09 | -0.03 | 0.23 | -0.02 | 0.08 | 0.07 | -0.19 | 1.00 |  |  |  |  |  |  |  |  |  |  |  |  |  |
| CZECH | -0.15 | -0.16 | 0.16 | 0.19 | -0.02 | -0.03 | 0.10 | -0.21 | 1.00 |  |  |  |  |  |  |  |  |  |  |  |  |
| ETH | 0.47 | -0.18 | 0.30 | 0.11 | 0.54 | 0.21 | -0.07 | 0.30 | -0.14 | 1.00 |  |  |  |  |  |  |  |  |  |  |  |
| FIN | -0.42 | 0.08 | 0.14 | 0.17 | -0.16 | 0.23 | -0.32 | 0.01 | 0.02 | -0.18 | 1.00 |  |  |  |  |  |  |  |  |  |  |
| FRAN | -0.28 | 0.35 | 0.09 | -0.02 | -0.18 | 0.11 | -0.33 | 0.00 | 0.10 | 0.01 | 0.25 | 1.00 |  |  |  |  |  |  |  |  |  |
| GERM | -0.41 | 0.25 | -0.06 | -0.07 | -0.43 | -0.20 | -0.09 | 0.04 | 0.30 | -0.34 | 0.24 | 0.47 | 1.00 |  |  |  |  |  |  |  |  |
| GRE | -0.20 | 0.09 | 0.24 | -0.02 | -0.30 | 0.19 | -0.23 | 0.16 | 0.09 | 0.12 | 0.03 | 0.10 | 0.23 | 1.00 |  |  |  |  |  |  |  |
| HON_K | -0.10 | 0.23 | 0.14 | -0.06 | -0.14 | 0.27 | -0.12 | -0.12 | -0.18 | -0.11 | -0.17 | -0.04 | -0.22 | -0.07 | 1.00 |  |  |  |  |  |  |
| HUNG | 0.04 | -0.08 | 0.14 | 0.24 | 0.05 | 0.08 | -0.06 | 0.08 | 0.30 | 0.05 | -0.16 | 0.07 | 0.08 | -0.09 | -0.09 | 1.00 |  |  |  |  |  |
| INDIA | 0.42 | 0.00 | 0.04 | -0.05 | 0.14 | -0.30 | 0.41 | 0.01 | 0.07 | 0.28 | -0.39 | 0.01 | 0.00 | -0.01 | 0.02 | 0.04 | 1.00 |  |  |  |  |
| INDO | 0.49 | -0.25 | 0.17 | 0.12 | 0.38 | 0.19 | -0.05 | 0.09 | -0.07 | 0.27 | -0.31 | -0.31 | -0.41 | -0.10 | 0.07 | 0.25 | 0.04 | 1.00 |  |  |  |
| IREL | -0.25 | 0.08 | 0.12 | -0.01 | -0.27 | 0.18 | -0.35 | 0.00 | -0.07 | -0.01 | 0.29 | 0.45 | 0.21 | 0.19 | -0.06 | -0.08 | -0.04 | -0.25 | 1.00 |  |  |
| ITALY | 0.04 | 0.12 | -0.12 | -0.10 | -0.17 | -0.26 | 0.27 | 0.11 | 0.17 | -0.02 | -0.10 | 0.28 | 0.50 | 0.29 | -0.34 | 0.16 | 0.27 | -0.19 | 0.06 | 1.00 |  |
| JAP | -0.57 | 0.26 | -0.25 | -0.05 | -0.41 | -0.23 | -0.13 | -0.06 | 0.13 | -0.40 | 0.43 | 0.36 | 0.60 | 0.00 | -0.28 | -0.03 | -0.27 | -0.44 | 0.20 | 0.20 | 1.00 |
| KOR | -0.37 | 0.29 | -0.32 | -0.09 | -0.33 | -0.27 | 0.04 | -0.08 | 0.03 | -0.31 | 0.33 | 0.27 | 0.53 | -0.03 | -0.20 | -0.17 | 0.02 | -0.45 | 0.21 | 0.26 | 0.70 |
| MAL | -0.03 | 0.01 | -0.09 | 0.15 | 0.03 | 0.12 | -0.10 | 0.01 | 0.10 | -0.14 | -0.09 | -0.11 | -0.04 | -0.05 | 0.06 | 0.36 | -0.27 | 0.38 | -0.01 | -0.01 | 0.04 |
| MEX | 0.23 | -0.07 | 0.16 | 0.25 | 0.21 | 0.14 | -0.08 | 0.14 | 0.23 | 0.29 | -0.10 | 0.02 | -0.04 | 0.00 | -0.15 | 0.30 | 0.18 | 0.19 | -0.07 | 0.25 | -0.08 |
| NETH | -0.19 | 0.61 | -0.13 | 0.03 | -0.27 | -0.01 | -0.24 | 0.02 | -0.22 | -0.14 | 0.08 | 0.27 | 0.31 | 0.18 | 0.25 | -0.02 | -0.05 | -0.23 | 0.14 | 0.02 | 0.24 |
| NOR | -0.40 | -0.02 | 0.18 | 0.10 | -0.19 | 0.30 | -0.48 | 0.12 | -0.25 | -0.02 | 0.64 | 0.10 | -0.15 | 0.10 | -0.02 | -0.21 | -0.44 | -0.20 | 0.36 | -0.38 | 0.18 |
| PAKI | 0.53 | -0.04 | 0.12 | 0.09 | 0.40 | 0.07 | 0.17 | 0.08 | 0.01 | 0.58 | -0.36 | 0.02 | -0.17 | 0.12 | -0.04 | -0.08 | 0.55 | 0.10 | -0.08 | 0.15 | -0.44 |
| PHIL | 0.51 | -0.34 | 0.11 | 0.14 | 0.53 | 0.35 | 0.02 | 0.05 | 0.01 | 0.40 | -0.10 | -0.13 | -0.34 | -0.09 | -0.05 | 0.07 | 0.07 | 0.51 | -0.13 | -0.18 | -0.36 |
| POL | 0.17 | 0.15 | 0.18 | 0.30 | 0.03 | -0.11 | 0.06 | 0.22 | 0.26 | -0.06 | -0.05 | 0.04 | 0.27 | 0.06 | -0.10 | 0.19 | 0.11 | 0.01 | 0.03 | 0.28 | 0.03 |
| PORT | 0.09 | 0.03 | 0.10 | 0.23 | 0.02 | 0.02 | -0.01 | 0.05 | 0.18 | 0.09 | 0.03 | -0.16 | 0.00 | 0.16 | -0.11 | 0.12 | 0.02 | 0.05 | -0.07 | 0.18 | -0.04 |
| ROM | 0.12 | -0.19 | 0.11 | 0.24 | 0.27 | 0.05 | 0.01 | 0.13 | 0.36 | 0.08 | 0.07 | -0.07 | 0.15 | -0.01 | -0.37 | 0.30 | -0.02 | 0.23 | -0.05 | 0.22 | -0.01 |
| SING | -0.06 | 0.16 | -0.06 | -0.06 | -0.14 | 0.12 | -0.24 | 0.03 | 0.00 | -0.16 | 0.16 | 0.26 | 0.22 | -0.01 | 0.01 | -0.05 | 0.02 | -0.11 | 0.31 | 0.10 | 0.20 |
| SPA | -0.26 | 0.06 | 0.17 | 0.18 | -0.28 | -0.04 | -0.04 | 0.00 | 0.19 | -0.06 | 0.23 | 0.21 | 0.19 | 0.31 | -0.04 | -0.01 | 0.05 | -0.25 | 0.12 | 0.35 | 0.29 |
| SWE | -0.27 | 0.13 | 0.21 | 0.01 | -0.12 | 0.30 | -0.43 | 0.10 | 0.08 | -0.13 | 0.40 | 0.18 | 0.14 | 0.09 | -0.05 | -0.11 | -0.19 | -0.09 | 0.25 | -0.13 | 0.23 |
| SWITZ | -0.39 | 0.25 | 0.06 | -0.26 | -0.32 | 0.15 | -0.44 | 0.04 | -0.04 | -0.15 | 0.26 | 0.44 | 0.36 | 0.19 | -0.05 | -0.14 | -0.16 | -0.33 | 0.57 | 0.06 | 0.26 |
| TAIW | -0.20 | 0.03 | -0.35 | -0.32 | -0.21 | -0.35 | 0.43 | -0.04 | 0.04 | -0.22 | -0.02 | -0.05 | 0.28 | 0.04 | -0.23 | -0.22 | 0.03 | -0.31 | 0.02 | 0.29 | 0.37 |
| THAI | 0.24 | 0.06 | 0.19 | 0.01 | 0.06 | -0.11 | 0.13 | 0.09 | 0.08 | 0.23 | -0.31 | 0.06 | 0.09 | 0.05 | -0.04 | 0.25 | 0.29 | 0.24 | 0.01 | 0.26 | -0.11 |
| TUN | 0.40 | -0.08 | -0.08 | 0.32 | 0.36 | -0.01 | 0.10 | 0.01 | -0.02 | 0.24 | -0.27 | -0.09 | -0.18 | 0.00 | -0.08 | 0.15 | 0.06 | 0.25 | -0.23 | 0.07 | -0.34 |
| TURK | 0.31 | 0.05 | 0.03 | 0.12 | 0.11 | -0.15 | 0.34 | 0.20 | 0.22 | 0.22 | -0.34 | -0.16 | 0.08 | 0.27 | -0.06 | 0.20 | 0.48 | 0.14 | -0.19 | 0.44 | -0.20 |
| UK | -0.38 | 0.21 | -0.06 | -0.06 | -0.29 | 0.05 | -0.43 | 0.08 | 0.05 | -0.16 | 0.38 | 0.53 | 0.48 | 0.33 | -0.19 | -0.18 | -0.19 | -0.46 | 0.43 | 0.21 | 0.50 |
| US | -0.56 | 0.21 | 0.29 | -0.02 | -0.50 | 0.25 | -0.56 | 0.08 | 0.15 | -0.17 | 0.32 | 0.38 | 0.52 | 0.41 | 0.02 | 0.04 | -0.28 | -0.27 | 0.37 | 0.03 | 0.42 |
| VIET | 0.69 | -0.28 | -0.03 | 0.10 | 0.54 | -0.12 | 0.50 | -0.02 | 0.11 | 0.38 | -0.42 | -0.30 | -0.25 | -0.09 | -0.16 | 0.14 | 0.40 | 0.50 | -0.32 | 0.21 | -0.46 |

| Correlation | KOR | MAL | MEX | NETH | NOR | PAKI | PHIL | POL | PORT | ROM | SING | SPA | SWE | SWITZ | TAIW | THAI | TUN | TURK | UK | US | VIET |
| --- | --- | --- | --- | --- | --- | --- | --- | --- | --- | --- | --- | --- | --- | --- | --- | --- | --- | --- | --- | --- | --- |
| KOR | 1.00 |  |  |  |  |  |  |  |  |  |  |  |  |  |  |  |  |  |  |  |  |
| MAL | -0.05 | 1.00 |  |  |  |  |  |  |  |  |  |  |  |  |  |  |  |  |  |  |  |
| MEX | 0.01 | 0.16 | 1.00 |  |  |  |  |  |  |  |  |  |  |  |  |  |  |  |  |  |  |
| NETH | 0.26 | -0.09 | -0.18 | 1.00 |  |  |  |  |  |  |  |  |  |  |  |  |  |  |  |  |  |
| NOR | 0.06 | -0.05 | -0.24 | 0.03 | 1.00 |  |  |  |  |  |  |  |  |  |  |  |  |  |  |  |  |
| PAKI | -0.25 | -0.18 | 0.26 | -0.08 | -0.34 | 1.00 |  |  |  |  |  |  |  |  |  |  |  |  |  |  |  |
| PHIL | -0.30 | 0.11 | 0.28 | -0.24 | -0.14 | 0.20 | 1.00 |  |  |  |  |  |  |  |  |  |  |  |  |  |  |
| POL | 0.11 | 0.03 | 0.19 | 0.18 | -0.27 | 0.08 | 0.05 | 1.00 |  |  |  |  |  |  |  |  |  |  |  |  |  |
| PORT | 0.06 | 0.04 | 0.03 | -0.05 | -0.09 | 0.13 | -0.02 | 0.19 | 1.00 |  |  |  |  |  |  |  |  |  |  |  |  |
| ROM | 0.06 | 0.28 | 0.32 | -0.36 | -0.09 | -0.02 | 0.18 | 0.16 | 0.30 | 1.00 |  |  |  |  |  |  |  |  |  |  |  |
| SING | 0.29 | -0.01 | -0.09 | 0.34 | 0.05 | -0.11 | -0.03 | 0.04 | -0.09 | -0.02 | 1.00 |  |  |  |  |  |  |  |  |  |  |
| SPA | 0.21 | -0.16 | 0.08 | -0.13 | 0.18 | 0.01 | -0.24 | 0.23 | 0.10 | -0.13 | -0.12 | 1.00 |  |  |  |  |  |  |  |  |  |
| SWE | 0.26 | 0.11 | 0.04 | 0.06 | 0.25 | -0.16 | -0.04 | 0.00 | 0.18 | 0.09 | 0.43 | -0.07 | 1.00 |  |  |  |  |  |  |  |  |
| SWITZ | 0.29 | -0.21 | -0.12 | 0.26 | 0.23 | -0.18 | -0.17 | -0.13 | -0.13 | -0.14 | 0.48 | -0.02 | 0.38 | 1.00 |  |  |  |  |  |  |  |
| TAIW | 0.52 | 0.08 | -0.32 | 0.03 | -0.14 | -0.04 | -0.22 | -0.02 | 0.10 | -0.02 | 0.17 | 0.01 | 0.07 | 0.04 | 1.00 |  |  |  |  |  |  |
| THAI | -0.01 | 0.09 | 0.10 | 0.02 | -0.30 | 0.34 | 0.12 | 0.33 | 0.31 | 0.11 | -0.04 | 0.05 | -0.25 | -0.21 | 0.14 | 1.00 |  |  |  |  |  |
| TUN | -0.26 | 0.11 | 0.25 | 0.07 | -0.31 | 0.25 | 0.22 | 0.24 | 0.09 | 0.17 | -0.08 | -0.12 | -0.24 | -0.32 | -0.26 | 0.17 | 1.00 |  |  |  |  |
| TURK | -0.07 | 0.01 | 0.19 | -0.02 | -0.47 | 0.44 | -0.03 | 0.34 | 0.50 | 0.18 | -0.25 | 0.16 | -0.29 | -0.35 | 0.14 | 0.49 | 0.22 | 1.00 |  |  |  |
| UK | 0.37 | -0.15 | -0.11 | 0.37 | 0.34 | -0.14 | -0.31 | -0.11 | -0.02 | 0.05 | 0.42 | 0.08 | 0.32 | 0.46 | 0.09 | -0.22 | -0.20 | -0.28 | 1.00 |  |  |
| US | 0.17 | -0.06 | -0.22 | 0.33 | 0.37 | -0.25 | -0.31 | -0.04 | 0.03 | -0.01 | 0.23 | 0.18 | 0.28 | 0.49 | 0.00 | -0.12 | -0.43 | -0.18 | 0.56 | 1.00 |  |
| VIET | -0.25 | 0.08 | 0.33 | -0.30 | -0.46 | 0.49 | 0.36 | 0.16 | 0.15 | 0.33 | -0.28 | -0.15 | -0.40 | -0.52 | -0.08 | 0.34 | 0.44 | 0.53 | -0.46 | -0.53 | 1 |

Figure S 1. Frequency distribution of Revealed Symmetric Augmented Comparative Advantages 2001 and 2019 for selected countries

Italy Test for null hypothesis of truncated normal distribution 2001:

Chi-square(2) = 0.829 with p-value 0.66057

Japan Test for null hypothesis of truncated normal distribution 2001:

Chi-square(2) = 1.723 with p-value 0.42254

China Test for null hypothesis of truncated normal distribution 2001:

Chi-square(2) = 1.350 with p-value 0.50918

US Test for null hypothesis of truncated normal distribution 2001:

Chi-square(2) = 4.031 with p-value 0.13324

Italy Test for null hypothesis of truncated normal distribution 2019:

Chi-square(2) = 0.152 with p-value 0.92663

Japan Test for null hypothesis of truncated normal distribution 2019:

Chi-square(2) = 0.526 with p-value 0.76885

China Test for null hypothesis of truncated normal distribution 2019:

Chi-square(2) = 0.971 with p-value 0.61533

US Test for null hypothesis of truncated normal distribution 2019:

Chi-square(2) = 2.464 with p-value 0.29170
